# Supplementary material for: Ritter-type amination of C(sp3)-H bonds enabled by electrochemistry with SO42−
Source: Nat Commun. 2022 Jul 16;13:4138. doi: 10.1038/s41467-022-31813-3 (PMC9288499; doi:10.1038/s41467-022-31813-3)
Supplement: Supplementary file 1 — Supplementary Information [file 41467_2022_31813_MOESM1_ESM.pdf]

Supplementary Information

Ritter-Type Amination Reaction Enabled by Electrochemistry with SO_4^{2-}

Ling Zhang,[1&] Youtian Fu,[1&] Yi Shen,[1] Chengyu Liu,[1] Maolin Sun,[2] Ruihua Cheng,[2] Weiping Zhu,[3] Xuhong Qian[3], Yueyue Ma,*[2]Jinxing Ye*[12]

1. Supplementary Methods 1
1.1 General Considerations 1
1.2 Optimization Tables 1
1.3 General Procedures for the Electrolysis 2
1.3.1 General procedure for the making of electrolytic cell 2
1.3.2 General procedure for electrolysis 2
1.3.3 Synthetic method for alkanes 3
1.3.4 Synthetic method for aromatic hydrocarbon 20-62 3
1.3.5 Synthetic method for other nitriles 63-68 4
1.4 Large scale general procedure for electrolysis: 4
1.4.1 Large scale general procedure for 1,3-Dimethyladamantane 5
1.4.2 Large scale general procedure for cyclooctane 5
1.4.3 Large scale general procedure for ethylbenzene 5
1.5 Transformations of the product 1, 3 and 25 6
1.6 Mechanism research experiment 7
2. Characterization Data for the Electrolysis Products 9
3. Supplementary References 107

1. Supplementary Methods

1.1 General Considerations

A1. Solvents. The acetonitrile used in the experiment is chromatographic grade. Other solvents were from commercial sources and used without purification unless otherwise noted.

A2. Analytical methods.

^{1}H NMR spectra and ^{13}C NMR spectra were recorded on a Bruker AV-400/500 spectrometer (400/500 MHz and 100/125 MHz). Chemical shifts (δ) for protons are reported in parts per million (ppm) downfield from tetramethylsilane and are referenced to residual solvent peak. Chemical shifts (δ) for carbon are reported in parts per million (ppm) downfield from tetramethylsilane and are referenced to the carbon resonances of the solvent. Data are reported as follows: chemical shift, multiplicity (br = broad, s = singlet, d = doublet, dd = doublet of doublets, t = triplet, dt = doublet of triplets, q = quartet, quint = quintet, m = multiplet), coupling constants (J) in Hertz (Hz), integration; "app" is used to denote the apparent splitting of a signal.

GC analysis was performed on an Echrom A 90 gas chromatograph equipped with a flame ionization detector using a fused silica capillary column.

High resolution mass spectrometry (HRMS)was carried out using MicroMass GCT CA 055 instrument and recorded on a MicroMass LCTTM spectrometer.

1.2 Optimization Tables

Other factors were screened in Supplementary Table 1., such as temperature, electrolyte and solvent.

Supplementary Table 1. Additional optimization for the reaction.

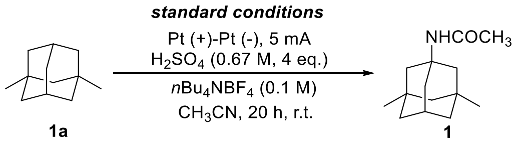

Entry	Variation from the standard conditions	Yield[a] (%)
1	None	93
2	$LiClO_4$ as electrolyte	73
3	$LiPF_4$ as electrolyte	81
4	0.08 M *n*Bu_4NBF_4 as electrolyte	89
5	0.06 M *n*Bu_4NBF_4 as electrolyte	85
6	0.04 M *n*Bu_4NBF_4 as electrolyte	83
7	40°C	66
8	10°C	81
9	CH_3CN/MeOH (1/1)	27
10	CH_3CN/EtOH (1/1)	23
11	CH_3CN/DCM (1/1)	20
12	CH_3CN/DCE (1/1)	31
13	CH_3CN/DMF (1/1)	37
14	CH_3CN/H_2O (1/1)	20
15	N_2 atmosphere	91

Standard conditions: 1,3-dimethyladamantane (82 mg, 0.5 mmol), 98% H_2SO_4 (108 μL, 0.67 M, 4 eq.), *n*Bu_4NBF_4 (0.1 M), CH_3CN 3 mL, Pt anode, Pt cathode, constant current=5 mA, under r.t. for 20 h; [a]Isolated yield.

1.3 General Procedures for the Electrolysis

1.3.1 General procedure for the making of electrolytic cell

The cathode and anode are assembled by commercially available PTFE screws, nuts and Pt sheets.

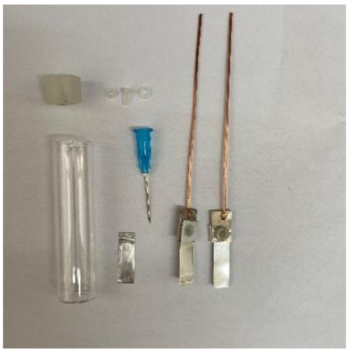

Supplementary Figure 1. All the materials used to make the electrolytic cell

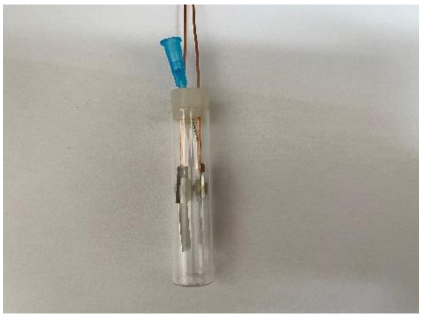

Supplementary Figure 2. The assemble of electrolytic cell

Two Pt elecrodes (10 mm ×35 mm) with the copper wires were cross the silica gel plug. Then it was placed into the cube (diameter 13 mm, length 70 mm) and the distance between two Pt sheets was almost 3 mm (Supplementary Figure 2).

1.3.2 General procedure for electrolysis

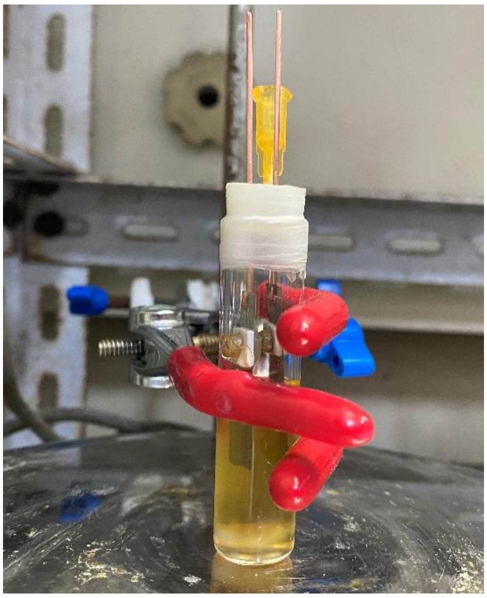

Supplementary Figure 3. The electrolysis under air

1.3.3 Synthetic method for alkanes

A. Tertiary alkane substrate 1-8

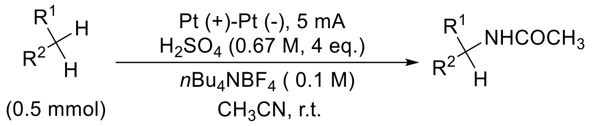

An oven-dried undivided cell was equipped with a stir bar, 0.5 mmol substrate, H_2SO_4 (108 μL, 2 mmol, 0.67 M, 4 eq.), *n*Bu_4NBF_4 (98 mg, 0.3 mmol, 0.1 M), CH_3CN (3 mL). Air has little effect on this reaction. Then the assembled electrodes were placed into the solution. The silica gel plug was sealed with film. The mixture was electrolyzed at a constant current of 5 mA until the tertiary alkane was completely consumed (usually 20 h) (Supplementary Figure 3). The Pt electrodes were washed by water, ethanol and DCM in turn. After the reaction is over, drop $NaHCO_3$ saturated solution into the reaction system slowly until no bubbles. The aqueous layer was separated and extracted with EtOAc (3×10 mL), and the combined organic layers were washed with brine and dried over anhydrous Na_2SO_4. Following concentration in vacuo, the crude product was purified by column chromatography on silica gel to give pure product.

B. Secondary alkane substrate 9-19

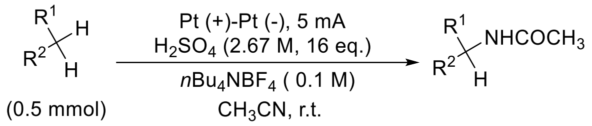

An oven-dried undivided cell was equipped with a stir bar, 0.5 mmol substrate, H_2SO_4 (432 μL, 8 mmol, 2.67 M, 16 eq.), *n*Bu_4NBF_4 (98 mg, 0.3 mmol, 0.1 M), CH_3CN (3 mL). Air has little effect on this reaction. Then the assembled electrodes were placed into the solution. The silica gel plug was sealed with film. The mixture was electrolyzed at a constant current of 5 mA until the secondary alkane was completely consumed (usually 24 h) (Supplementary Figure 3). The Pt electrodes were washed by water, ethanol and DCM in turn. After the reaction is over, drop $NaHCO_3$ saturated solution into the reaction system slowly until no bubbles. The aqueous layer was separated and extracted with EtOAc (3×10 mL), and the combined organic layers were washed with brine and dried over anhydrous Na_2SO_4. Following concentration in vacuo, the crude product was purified by column chromatography on silica gel to give pure product.

1.3.4 Synthetic method for aromatic hydrocarbon 20-62

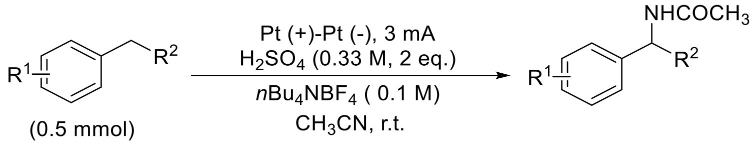

An oven-dried undivided cell was equipped with a stir bar, 0.5 mmol substrate, H_2SO_4 (54 μL, 1 mmol, 0.33 M, 2 eq.), *n*Bu_4NBF_4 (98 mg, 0.3 mmol, 0.1 M), CH_3CN (3 mL). Air has little effect on this reaction. Then the assembled electrodes were placed into the solution. The silica gel plug was sealed with film. The mixture was electrolyzed at a constant current of 3 mA until the aromatic hydrocarbon was completely consumed (usually 12 h) (Supplementary Figure 3). The Pt electrodes were washed by water,

ethanol and DCM in turn. After the reaction is over, drop $NaHCO_3$ saturated solution into the reaction system slowly until no bubbles. The aqueous layer was separated and extracted with EtOAc (3×10 mL), and the combined organic layers were washed with brine and dried over anhydrous Na_2SO_4. Following concentration in vacuo, the crude product was purified by column chromatography on silica gel to give pure product.

1.3.5 Synthetic method for other nitriles 63-68

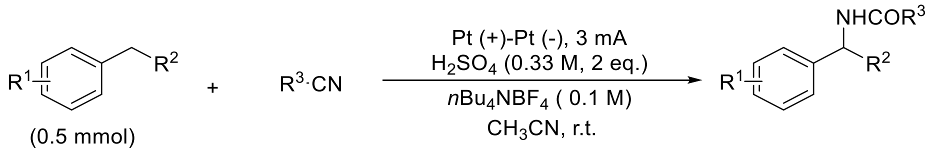

An oven-dried undivided cell was equipped with a stir bar, 0.5 mmol substrate, H_2SO_4 (54 μL, 1 mmol, 0.33 M, 2 eq.), *n*Bu_4NBF_4 (98 mg, 0.3 mmol, 0.1 M), solvent (3 mL). Air has little effect on this reaction. Then the assembled electrodes were placed into the solution. The silica gel plug was sealed with film. The mixture was electrolyzed at a constant current of 5 mA until the substrate was completely consumed (usually 12 h) (Supplementary Figure 3). The Pt electrodes were washed by water, ethanol and DCM in turn. After the reaction is over, drop $NaHCO_3$ saturated solution into the reaction system slowly until no bubbles. The aqueous layer was separated and extracted with EtOAc (3×10 mL), and the combined organic layers were washed with brine and dried over anhydrous Na_2SO_4. Following concentration in vacuo, the crude product was purified by column chromatography on silica gel to give pure product. (For the synthesis of product **63**, the concentration of H_2SO_4 is 0.67 M.)

1.4 Large scale general procedure for electrolysis:

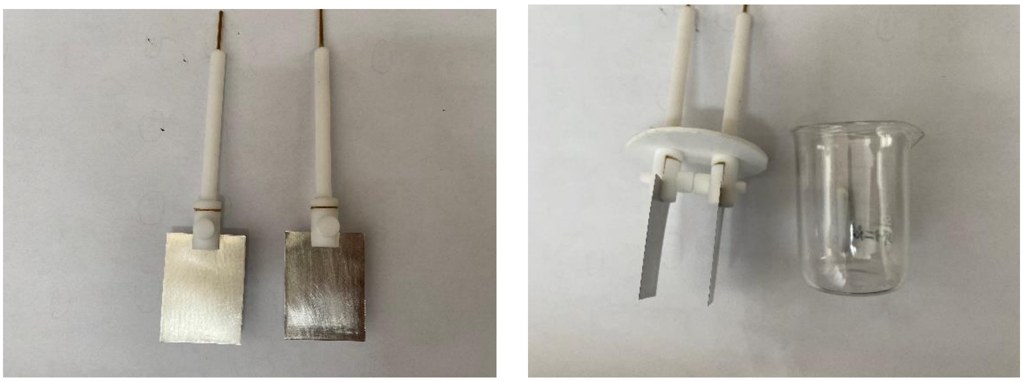

(a) **(b)**

Supplementary Figure 4. The setup for large scale electrolysis

Two Pt elecrodes (65 mm×55 mm×1mm) were assembled into sealed cap (Figure S4, a). Then it was placed into the bake (diameter 35 mm, length 60 mm) and the distance between two Pt sheets was almost 24 mm (Figure S4, b)

1.4.1 Large scale general procedure for 1,3-Dimethyladamantane

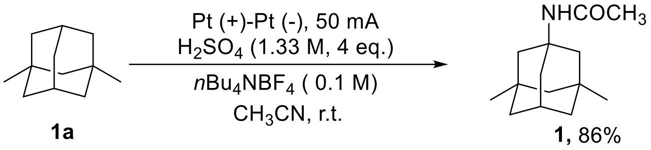

1a **1**, 86%

An oven-dried undivided cell was equipped with a stir bar, 1,3-dimethyladamantane (1.64 g, 10 mmol, 1 eq.), H_2SO_4 (2.16 mL, 40 mmol, 1.33 M, 4 eq), *n*Bu$_4$NBF$_4$ (987 mg, 3 mmol, 0.1 M), CH_3CN (30 mL). Then the assembled electrodes were placed into the solution. The silica gel plug was sealed with film. Air has little effect on this reaction. The mixture was electrolyzed at a constant current of 50 mA until the 1,3-dimethyladamantane was completely consumed. (Supplementary Figure 4). The Pt electrodes were washed by water, ethanol and DCM in turn. After the reaction is over, drop $NaHCO_3$ saturated solution into the reaction system slowly until no bubbles. The aqueous layer was separated and extracted with EtOAc (3×100 mL), and the combined organic layers were washed with brine and dried over anhydrous Na_2SO_4. Following concentration in vacuo, the crude product was purified by column chromatography on silica gel to give pure product **1** (1.9 g, 86%).

1.4.2 Large scale general procedure for cyclooctane

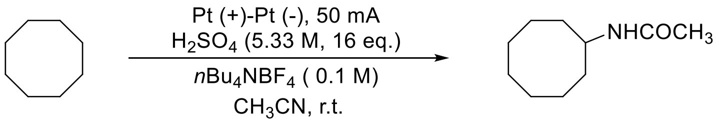

12a **12**, 58%

An oven-dried undivided cell was equipped with a stir bar, cyclooctane (1.12 g, 10 mmol, 1 eq.), H_2SO_4 (8.64 mL, 160 mmol, 5.33 M, 16 eq.), *n*Bu$_4$NBF$_4$ (987 mg, 3 mmol, 0.1 M), CH_3CN (30 mL). Then the assembled electrodes were placed into the solution. The silica gel plug was sealed with film. Air has little effect on this reaction. The mixture was electrolyzed at a constant current of 50 mA until the cyclooctane was completely consumed. (Supplementary Figure 4). The Pt electrodes were washed by water, ethanol and DCM in turn. After the reaction is over, drop $NaHCO_3$ saturated solution into the reaction system slowly until no bubbles. The aqueous layer was separated and extracted with EtOAc (3×100 mL), and the combined organic layers were washed with brine and dried over anhydrous Na_2SO_4. Following concentration in vacuo, the crude product was purified by column chromatography on silica gel to give pure product **12** (0.98 g, 58%).

1.4.3 Large scale general procedure for ethylbenzene

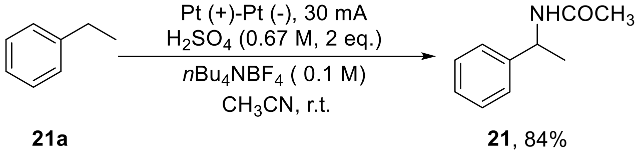

21a **21**, 84%

An oven-dried undivided cell was equipped with a stir bar, ethylbenzene (1.06 g, 10 mmol, 1 eq.), H_2SO_4 (1.08 mL, 20 mmol, 0.67 M, 2 eq.), *n*Bu$_4$NBF$_4$ (987 mg, 3 mmol, 0.1 M), CH_3CN (30 mL). Then the

assembled electrodes were placed into the solution. The silica gel plug was sealed with film. Air has little effect on this reaction. The mixture was electrolyzed at a constant current of 30 mA until the ethylbenzene was completely consumed. (Supplementary Figure 4). The Pt electrodes were washed by water, ethanol and DCM in turn. After the reaction is over, drop $NaHCO_3$ saturated solution into the reaction system slowly until no bubbles. The aqueous layer was separated and extracted with EtOAc (3 ×100 mL), and the combined organic layers were washed with brine and dried over anhydrous Na_2SO_4. Following concentration in vacuo, the crude product was purified by column chromatography on silica gel to give pure product **21** (1.36 g, 84%).

1.5 Transformations of the product 1, 3 and 25

A. Transformations of the product 1[1]

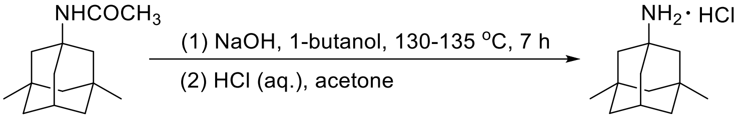

1 **1b**, 85%

A mixture of **1** (664 mg, 3 mmol) in 1-butanol (10 mL) and pulverized NaOH (960 mg, 24 mmol) was heated at 130-135 ℃ for 7 h, quenched with water, and extracted with toluene (50 mL*3). The toluene layer was washed with water and evaporated to give an oil of memantine free base. The free base was added 5 M aq. HCl (3 mL), stirred at 55–60 ℃ for 1 h, and then cooled to room temperature. The resulting aqueous layer was added acetone (20 mL), stirred at 50 ℃ for 1 h, and then at 0–5 ℃ for additional 1 h. The obtained colorless precipitate was filtered off and dried under vacuum to give white solid **1b**, which was filtered and dried under vacuum. (550 mg, 85%).

B. Transformations of the product 3[2]

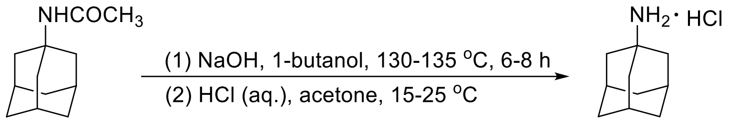

3 **3b**, 82%

A mixture of **3** (579 mg, 3 mmol) in 1-butanol (10 mL) and pulverized sodium hydroxide (960 mg, 24 mmol) was heated at 130-135 ℃ for 6-8 h, quenched with water, and extracted with toluene (50 mL*3). The toluene layer was washed with water and evaporated to give an oil of amantadine free base. The free base was added 5 M aq. HCl (3 mL), stirred at 55–60 ℃ for 1 h, and then cooled to room temperature. The resulting aqueous layer was added acetone (20 mL), stirred at 50 ℃ for 1 h, and then at 0–5 ℃ for additional 1 h. The obtained colorless precipitate was filtered off and dried under vacuum to give white solid **3b**, which was filtered and dried under vacuum. (461 mg, 82 %).

C. Transformations of the product 25[3]

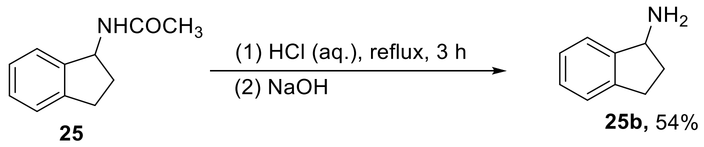

Amide product **25** (525 mg, 3 mmol) was dissolved in 10 mL of HCl (3 M) and reflux for 3 hours. Reaction was basified to pH 12 and extracted with ethyl acetate (3 × 30 mL), the combined organic layers were dried by Na_2SO_4. The organic solvent was removed under reduced pressure. The residue was purified by column silica gel column chromatography to give colorless oil **25b** (335 mg, 84%).

1.6 Mechanism research experiment

A. Control experiment

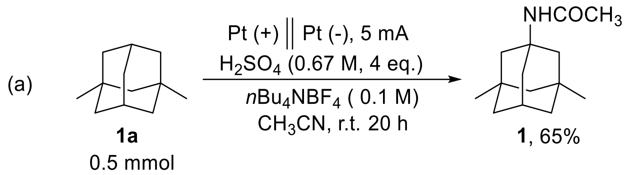

The anodic chamber and cathodic chamber are separated by anion exchange membrane. The anodic chamber is equipped with **1a** (0.5 mmol), H_2SO_4 (2 mmol, 0.67 M, 4 eq.), *n*Bu_4NBF_4 (98 mg, 0.3 mmol, 0.1 M) and CH_3CN (3 mL), the cathodic chamber was added *n*Bu_4NBF_4 (98 mg, 0.3 mmol, 0.1 M) and CH_3CN (3 mL). The mixture is electrolyzed at a constant current of 5 mA for 20 h. (Supplementary Figure 5.) Then $NaHCO_3$ saturated solution is slowly added dropwise until there are no bubbles in the anodic chamber. The aqueous layer was separated and extracted with EtOAc (3×10 mL), and the combined organic layers were washed with brine and dried over anhydrous Na_2SO_4. Following concentration in vacuo, the crude product was purified by column chromatography on silica gel to give pure product **1** (71.8 mg, 65%).

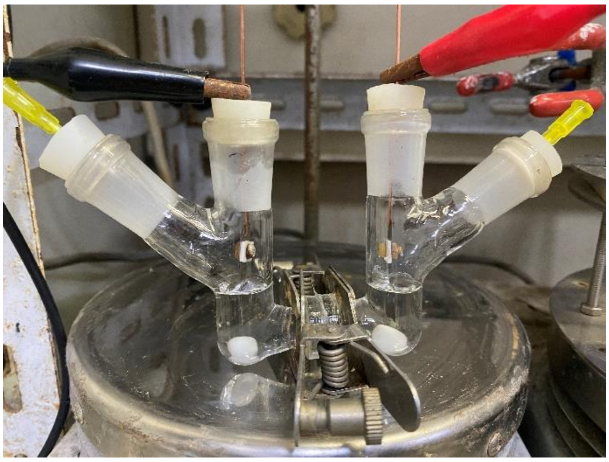

Supplementary Figure 5. The divided cell experiment set-up.

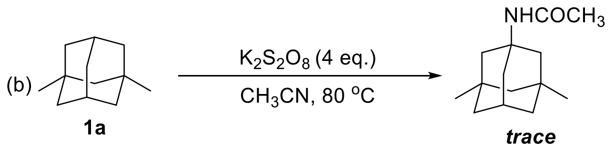

A mixture of **1a** (82 mg, 0.5 mmol) and $K_2S_2O_8$ (540 mg, 2 mmol) in acetonitrile (3 mL) was heated at 80 ℃ for 20 h. Gas chromatography monitoring of the reaction showed that trace amount of product was generated.

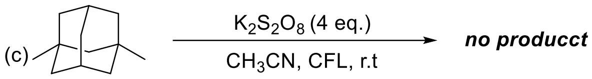

A mixture of **1a** (82 mg, 0.5 mmol) and $K_2S_2O_8$ (540 mg, 2 mmol) in acetonitrile (3 mL) was irradiated by CFL at r.t. for 20 h. Gas chromatography monitoring of the reaction showed that no product was generated.

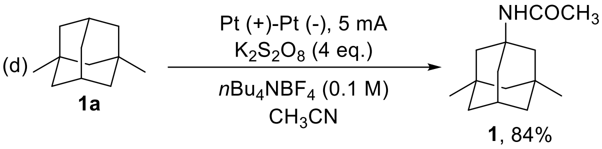

An oven-dried undivided cell was equipped with a stir bar, **1a** (82 mg, 0.5 mmol), $K_2S_2O_8$ (540 mg, 2 mmol), nBu$_4$NBF$_4$ (98 mg, 0.3 mmol, 0.1 M), 3 mL CH_3CN. Then the assembled electrodes were placed into the solution. The silica gel plug was sealed with film. Air has little effect on this reaction. The mixture was electrolyzed at a constant current of 5 mA for 20 h. Detect the conversion of the reaction by GC. Then, the Pt electrodes were washed by water, ethanol and DCM in turn. After the reaction is over, drop $NaHCO_3$ saturated solution into the reaction system slowly until no bubbles. The aqueous layer was separated and extracted with EtOAc (3×10 mL), and the combined organic layers were washed with brine and dried over anhydrous Na_2SO_4. Following concentration in vacuo, the crude product was purified by column chromatography on silica gel to give pure product **1** (92.8 mg, 84%).

B. The mechanism research of carbon radical

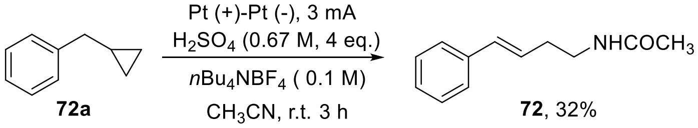

72a was prepared according to the method reported by Melchiorre.[4] An oven-dried undivided cell was equipped with a stir bar, (cyclopropylmethyl) benzene (66 mg, 0.5 mmol, 1 eq.), H_2SO_4 (108 µL, 2 mmol, 0.67 M, 4 eq.), nBu$_4$NBF$_4$ (98 mg, 0.3 mmol, 0.1 M), and CH_3CN (3 mL). Then put the assembled electrode into the solution. The silicone plug is sealed with a film. The mixture is electrolyzed at a constant current of 3 mA for 3 h. Then $NaHCO_3$ saturated solution is slowly added dropwise until there are no bubbles in the reaction system. The aqueous layer was separated and extracted with EtOAc (3×10 mL), and the combined organic layers were washed with brine and dried over anhydrous Na_2SO_4. Following concentration in vacuo, the crude product was purified by column chromatography on silica gel to give pure product **72** (30 mg, 32%).

C. UV absorption spectra of the iodometric persulfate measurement system

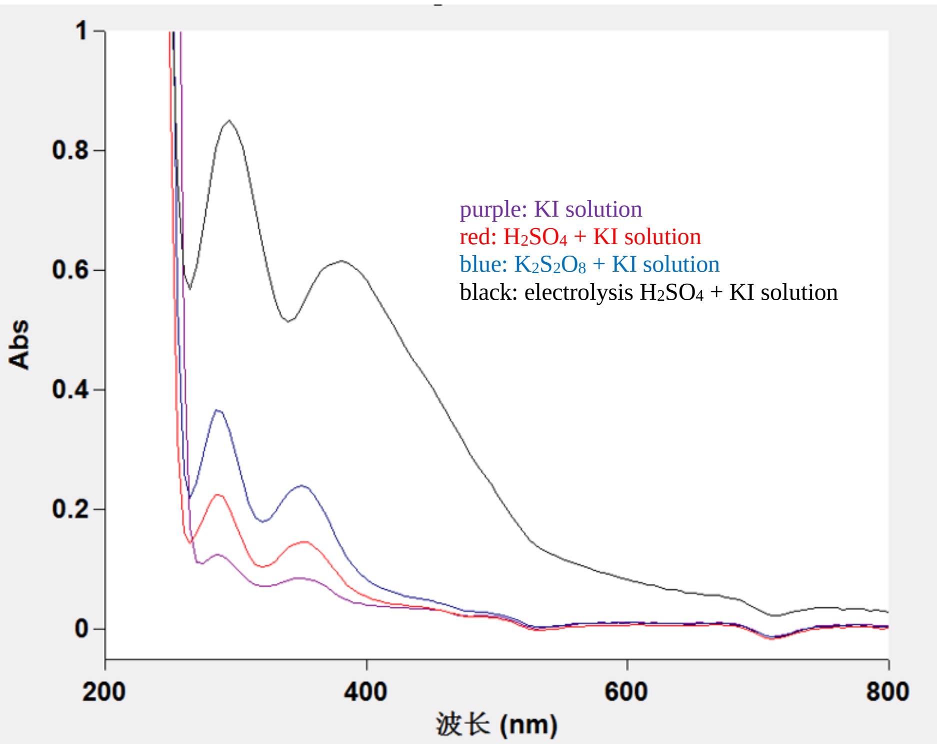

Supplementary Figure 6. UV absorption spectra of the iodometric persulfate measurement system

Prepare KI standard solution (0.2 g $NaHCO_3$, 4 g KI and 40 mL water) and a series of solutions: (a) $K_2S_2O_8$ aqueous solution (0.02 M), (b) H_2SO_4 (2 mmol, 0.67 M in acetonitrile) electrolyzed for 2 h under standard conditions, (c) H_2SO_4 solution (0.67 M in acetonitrile). Add the prepared different solutions to the KI standard solution and scan the absorption spectrum to obtain Supplementary Figure 6.

Base on the work of Kurakalva,[5] the formation of persulfate by electrolysis of sulfuric acid was determinded by spectrophotometric method. The $K_2S_2O_8$ and KI standard solutions have absorptions at 288 nm and 352 nm, which is coincidence with the absorption of electrolyzed sulfuric acid.

2. Characterization Data for the Electrolysis Products

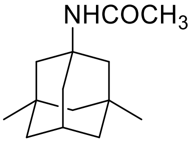

1

***N*-(3,5-dimethyladamantan-1-yl)acetamide**

White solid, 93% yield, 4.4 F mol^{-1}. ^{1}H NMR (400 MHz, Chloroform-*d*) δ 5.26 (d, *J* = 23.4 Hz, 1H), 2.11 (hept, *J* = 3.2 Hz, 1H), 1.88 (s, 3H), 1.83 – 1.78 (m, 2H), 1.67 – 1.56 (m, 4H), 1.36 (dt, *J* = 12.3, 2.7 Hz, 2H), 1.30 – 1.23 (m, 2H), 1.20 – 1.07 (m, 2H), 0.83 (s, 6H).^{13}C NMR (101 MHz, Chloroform-*d*) δ 169.54, 53.34, 50.57, 47.46 (×2), 42.64 (×2), 40.02, 32.26 (×2), 30.05 (×2), 30.03, 24.51. HRMS (EI): exact mass calculated for $C_{14}H_{23}NO$ $[M]^+$ require m/z = 221.1780, found m/z = 221.1778.

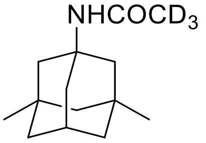

2

***N*-(3,5-dimethyladamantan-1-yl)acetamide-2,2,2-*d*₃**

White solid, 85% yield. 4.4 F mol^{-1}. ^{1}H NMR (400 MHz, Chloroform-*d*) δ 5.94 (s, 1H), 2.03 – 1.97 (m, *J* = 2.9 Hz, 1H), 1.73 (q, *J* = 3.3 Hz, 2H), 1.53 (q, *J* = 3.7, 2.6 Hz, 4H), 1.28 – 1.21 (m, 2H), 1.20 – 1.09 (m, 2H), 1.09 – 0.96 (m, 2H), 0.76 – 0.69 (m, 6H). ^{13}C NMR (101 MHz, Chloroform-d) δ 169.44, 53.44, 50.59, 47.57(×2), 42.65(×2), 40.13, 32.35(×2), 30.10, 30.06(×2), δ 23.95 (dt, J = 38.6, 19.4 Hz). HRMS (EI): exact mass calculated for $C_{14}H_{20}D_3NO$ $[M]^+$ require m/z = 224.1968, found m/z = 224.1966.

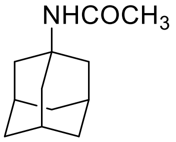

3

***N*-(adamantan-1-yl)acetamide**

White solid, 70% yield. 6.4 F mol^{-1}. ^{1}H NMR (400 MHz, Chloroform-*d*) δ 5.42 (s, 1H), 2.04 – 1.98 (m, 3H), 1.94 (d, *J* = 3.2 Hz, 6H), 1.85 (s, 3H), 1.62 (t, *J* = 3.2 Hz, 6H). ^{13}C NMR (101 MHz, Chloroform-*d*) δ 169.33, 51.79, 41.57 (×3), 36.34 (×3), 29.40 (×3), 24.64. HRMS (EI): exact mass calculated for $C_{12}H_{19}NO$ $[M]^+$ require m/z = 193.1467, found m/z = 193.1466.

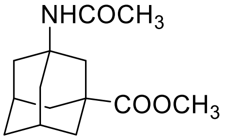

4

methyl-3-acetamidoadamantane-1-carboxylate

White solid, 68% yield. 5.2 F mol^{-1}. ^{1}H NMR (400 MHz, Chloroform-*d*) δ 5.71 (s, 1H), 3.55 (s, 3H), 2.16 – 2.03 (m, 4H), 1.96 – 1.89 (m, 2H), 1.89 – 1.83 (m, 2H), 1.83 (s, 3H), 1.76 – 1.73 (m, 4H), 1.56 (dd, *J* = 6.8, 3.3 Hz, 2H). ^{13}C NMR (101 MHz, Chloroform-*d*) δ 176.94, 169.60, 51.81, 51.64, 42.46, 42.24, 40.52 (×2), 37.82 (×2), 35.24, 28.96 (×2), 24.42. HRMS (EI): exact mass calculated for $C_{14}H_{21}NO_3$ $[M]^+$ require m/z = 251.1521, found m/z = 251.1520.

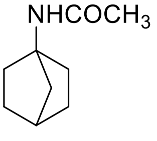

5

***N*-(bicyclo[2.2.1]heptan-1-yl)acetamide**

White solid, 82% yield. 5.2 F mol^{-1}. ^{1}H NMR (400 MHz, Chloroform-*d*) δ 6.28 (s, 1H), 3.60 (td, *J* = 7.8, 3.5 Hz, 1H), 2.17 (d, J = 4.2 Hz, 1H), 2.12 – 2.08 (m, 1H), 1.86 (s, 3H), 1.66 (ddd, *J* = 13.1, 8.0, 2.4 Hz, 1H), 1.49 – 1.24 (m, 3H), 1.18 – 0.97 (m, 4H). ^{13}C NMR (101 MHz, Chloroform-*d*) δ 169.54, 52.79, 42.25, 39.88, 35.59, 35.40, 28.16, 26.47, 23.17. HRMS (EI): exact mass calculated for $C_9H_{15}NO$ $[M]^+$ require m/z = 153.1154, found m/z = 153.1152.

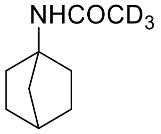

6

***N*-(bicyclo[2.2.1]heptan-1-yl)acetamide-2,2,2-*d*$_3$**

White solid, 78% yield. 5.2 F mol^{-1}. ^{1}H NMR (600 MHz, Chloroform-d) δ 6.16 (s, 1H), 3.62 (td, J = 7.9, 3.7 Hz, 1H), 2.19 (d, J = 4.3 Hz, 1H), 2.12 (d, J = 4.3 Hz, 1H), 1.68 (ddd, J = 12.9, 8.3, 2.8 Hz, 1H), 1.40 (dddd, J = 19.7, 12.1, 10.1, 5.9 Hz, 2H), 1.30 (d, J = 9.9 Hz, 1H), 1.19 – 1.08 (m, 3H), 1.04 (td, J = 9.2, 4.7 Hz, 1H). ^{13}C NMR (151 MHz, Chloroform-d) δ 169.49, 52.77, 42.28, 40.02, 35.62, 35.44, 28.16, 26.48, 22.46 (h, J = 19.9 Hz). HRMS (EI): exact mass calculated for $C_9H_{12}D_3NO$ [M]$^+$ require m/z = 156.1342, found m/z = 156.1344.

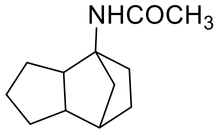

7

***N*-(octahydro-4*H*-4,7-methanoinden-4-yl)acetamide**

White solid, 83% yield. 4.4 F mol^{-1}. ^{1}H NMR (400 MHz, Chloroform-*d*) δ 6.01 (s, 1H), 2.42 – 2.37 (m, 1H), 2.14 – 2.00 (m, 3H), 1.86 (s, 3H), 1.82 – 1.69 (m, 3H), 1.69 – 1.63 (m, 1H), 1.58 – 1.46 (m, 1H), 1.46 – 1.12 (m, 6H). ^{13}C NMR (101 MHz, Chloroform-*d*) δ 168.99, 71.46, 54.70, 46.67, 41.50, 41.06, 34.04, 28.82, 26.13, 24.02, 23.57, 22.39. HRMS (EI): exact mass calculated for $C_{12}H_{19}NO$ [M]$^+$ require m/z = 193.1467, found m/z = 193.1468.

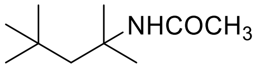

8

***N*-(2,4,4-trimethylpentan-2-yl)acetamide**

White solid, 44% yield. 6.0 F mol^{-1}. ^{1}H NMR (400 MHz, Chloroform-*d*) δ 5.28 (s, 1H), 1.88 (s, 3H), 1.73 (s, 2H), 1.38 (s, 6H), 0.99 (s, 9H).^{13}C NMR (101 MHz, Chloroform-*d*) δ 169.31, 55.11, 51.16, 31.64, 31.43 (×3), 29.32 (×2), 24.78. HRMS (EI): exact mass calculated for $C_{10}H_{21}NO$ [M]$^+$ require m/z = 171.1623, found m/z = 171.1625.

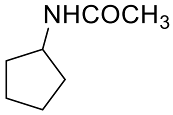

9

***N*-cyclopentylacetamide**

White solid, 65% yield. 9 F mol^{-1}. ^{1}H NMR (400 MHz, Chloroform-*d*) δ 6.04 (s, 1H), 4.12 (h, *J* = 7.0 Hz, 1H), 2.02-1.89 (m, 5H), 1.70 – 1.43 (m, 4H), 1.42 – 1.22 (m, 2H). ^{13}C NMR (101 MHz, Chloroform-*d*) δ 169.93, 51.17, 32.92 (×2), 23.66 (×2), 23.26. HRMS (EI): exact mass calculated for $C_7H_{13}NO$ [M]$^+$ require m/z = 127.0997, found m/z = 127.0998.

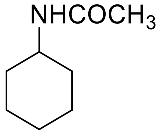

10

***N*-cyclohexylacetamide**

White solid, 57% yield. 9 F mol^{-1}. ^{1}H NMR (400 MHz, Chloroform-*d*) δ 5.60 (s, 1H), 3.79 – 3.64 (m, 1H), 1.92 (s, 3H), 1.88 (dd, *J* = 12.6, 3.8 Hz, 2H), 1.67 (dt, *J* = 13.3, 3.9 Hz, 2H), 1.58 (dt, *J* = 12.7, 3.7 Hz, 1H), 1.42 – 1.20 (m, 2H), 1.15 – 1.01 (m, 3H). ^{13}C NMR (101 MHz, Chloroform-*d*) δ 169.22, 48.25, 33.16 (×2), 25.51, 24.88 (×2), 23.52. HRMS (EI): exact mass calculated for $C_8H_{15}NO$ $[M]^+$ require m/z = 141.1154, found m/z = 141.1152.

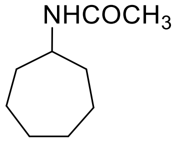

11

***N*-cycloheptylacetamide**

White solid, 50% yield. 9 F mol^{-1}.^{1}H NMR (400 MHz, Chloroform-*d*) δ 5.73 (s, 1H), 3.89 (qt, *J* = 8.5, 4.4 Hz, 1H), 1.91 (s, 3H), 1.89 (m, 2H), 1.63 – 1.52 (m, 4H), 1.51 – 1.30 (m, 6H). ^{13}C NMR (101 MHz, Chloroform-*d*) δ 168.90, 50.45, 35.10 (×2), 27.89 (×2), 24.03 (×2), 23.51. HRMS (EI): exact mass calculated for $C_9H_{17}NO$ $[M]^+$ require m/z = 155.1310, found m/z = 155.1308.

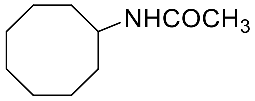

12

***N*-cyclooctylacetamide**

White solid, 71% yield. 9 F mol^{-1}. ^{1}H NMR (400 MHz, Chloroform-*d*) δ 5.84 – 5.65 (m, 1H), 3.94 (tp, *J* = 8.1, 4.1 Hz, 1H), 1.91 (s, 3H), 1.78 (td, *J* = 12.1, 10.9, 4.3 Hz, 2H), 1.62 – 1.44 (m, 12H). ^{13}C NMR (101 MHz, Chloroform-*d*) δ 167.89, 48.40, 31.11 (×2), 26.22 (×2), 24.34, 22.62 (×2), 22.54. HRMS (EI): exact mass calculated for $C_{10}H_{19}NO$ $[M]^+$ require m/z = 169.1467, found m/z = 169.1469.

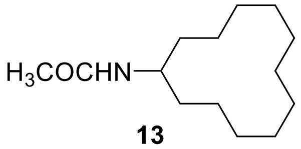

13

***N*-cyclododecylacetamide**

White solid, 32% yield. 9 F mol^{-1}. ^{1}H NMR (400 MHz, Chloroform-*d*) δ 5.52 (s, 1H), 4.02 (th, *J* = 7.3, 4.4, 3.8 Hz, 1H), 1.92 (s, 3H), 1.56 (q, *J* = 6.6 Hz, 2H), 1.47 – 1.16 (m, 20H). ^{13}C NMR (101 MHz, Chloroform-*d*) δ 169.33, 46.15, 30.13(×2), 24.00(×2), 23.75, 23.51, 23.44(×2), 23.33(×2), 21.38(×2). HRMS (EI): exact mass calculated for $C_{14}H_{27}NO$ $[M]^+$ require m/z = 225.2093, found m/z = 225.2092.

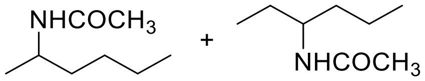

14a **14b**

***N*-(hexan-2-yl)acetamide and *N*-(hexan-3-yl)acetamide**

Light yellow oil, 54% yield. 9 F mol^{-1}. ^{1}H NMR (400 MHz, Chloroform-*d*, mixture of **14a** and **14b**) δ 5.33 (d, *J* = 37.7 Hz, 2H), 3.94 (dh, *J* = 9.2, 7.0 Hz, 2H), 1.96 (s, 6H), 1.59 – 1.20 (m, 12H), 1.11 (s, 3H),

0.94 – 0.83 (m, 9H). ^{13}C NMR (101 MHz, Chloroform-*d*, mixture of **14a** and **14b**) δ 168.73, 168.35, 49.40, 44.29, 35.89, 35.63, 27.16, 26.90, 22.54, 22.51, 21.57, 19.96, 18.09, 13.02,13.00, 9.12. HRMS (EI): exact mass calculated for $C_8H_{17}NO$ $[M]^+$ require m/z = 143.1310, found m/z = 143.1308

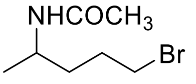

15

_N_-(5-bromopentan-2-yl)acetamide

Light yellow solid, 62% yield. 9 F mol^{-1}. ^{1}H NMR (400 MHz, Chloroform-*d*) δ 6.19 (d, *J* = 8.6 Hz, 1H), 3.91 (qd, *J* = 8.2, 6.3 Hz, 1H), 3.35 (t, *J* = 6.6 Hz, 2H), 1.90 (s, 3H), 1.81 (p, *J* = 7.2 Hz, 2H), 1.50 (m, 2H), 1.07 (d, *J* = 6.6 Hz, 3H).^{13}C NMR (101 MHz, Chloroform-*d*) δ 168.62, 43.51, 34.42, 32.68, 28.32, 22.44, 20.08. HRMS (EI): exact mass calculated for $C_7H_{14}BrNO$ $[M]^+$ require m/z = 207.0259, found m/z = 207.0261.

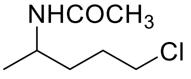

16

_N_-(5-chloropentan-2-yl)acetamide

White solid, 60% yield. 9 F mol^{-1}. ^{1}H NMR (400 MHz, Chloroform-*d*) δ 6.40 (d, *J* = 8.5 Hz, 1H), 3.88 (dq, *J* = 14.3, 7.8, 7.4 Hz, 1H), 3.44 (t, *J* = 6.5 Hz, 2H), 1.86 (s, 3H), 1.69 (p, *J* = 7.1 Hz, 2H), 1.47 (hept, *J* = 7.4 Hz, 2H), 1.04 (d, *J* = 6.7 Hz, 3H). ^{13}C NMR (101 MHz, Chloroform-*d*) δ 169.74, 44.84, 44.44, 33.99, 29.17, 23.22, 20.90. HRMS (EI): exact mass calculated for $C_7H_{14}ClNO$ $[M]^+$ require m/z = 163.0764, found m/z = 163.0762.

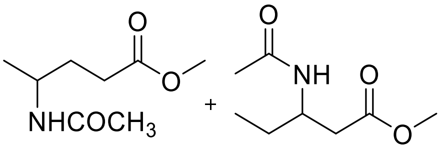

17a **17b**

methyl 4-acetamidopentanoate and methyl 3-acetamidopentanoate

White solid, 55% yield. 9 F mol^{-1}.^{1}H NMR (400 MHz, Chloroform-*d*, mixture of **17a** and **17b**) δ 6.29 (d, *J* = 8.8 Hz, 1H), 6.05 (d, *J* = 8.5 Hz, 2.3H), 4.08 (dddd, *J* = 12.5, 9.2, 7.2, 5.5 Hz, 1H), 3.91 (tdd, *J* = 8.7, 6.9, 3.7 Hz, 2.3H), 3.60 (d, *J* = 5.0 Hz, 10H), 2.46 (d, *J* = 5.6 Hz, 2H), 2.35 – 2.25 (m, 4.6H), 1.89 (d, *J* = 11.1 Hz, 10H), 1.79 – 1.58 (m, 4.6H), 1.48 (p, *J* = 7.3 Hz, 2H), 1.07 (d, *J* = 6.6 Hz, 6.7H), 0.84 (t, *J* = 7.4 Hz, 3H). ^{13}C NMR (101 MHz, Chloroform-*d*, mixture of **17a** and **17b**) δ 174.06, 172.21, 169.78, 51.59 (×2), 47.57, 44.87, 38.15, 31.51, 30.83, 27.04, 23.26, 23.21, 20.84, 10.52. HRMS (EI): exact mass calculated for $C_8H_{15}NO_3$ $[M]^+$ require m/z = 173.1052, found m/z = 173.1067

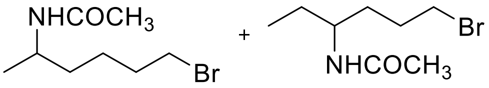

18a **18b**

_N_-(6-bromohexan-2-yl)acetamide and 6-bromo-_N_-(methoxymethyl)hexan-3-amine

White solid, 64% yield. 9 F mol^{-1}. **18a**:^{1}H NMR (400 MHz, Chloroform-*d*) δ 5.91 (d, *J* = 8.6 Hz, 1H), 3.91 (h, *J* = 7.7, 7.2 Hz, 1H), 3.34 (t, *J* = 6.7 Hz, 2H), 1.90 (s, 3H), 1.80 (dt, *J* = 14.5, 7.3 Hz, 2H), 1.39 (dd, *J* = 5.4, 2.5 Hz, 4H), 1.07 (d, *J* = 6.6 Hz, 3H). ^{13}C NMR (101 MHz, Chloroform-*d*) δ 169.59, 44.96, 35.81, 33.71, 32.39, 24.54, 23.40, 20.82.

18b:^{1}H NMR (400 MHz, Chloroform-*d*) δ 5.59 (d, *J* = 9.3 Hz, 1H), 3.96 – 3.76 (m, 1H), 3.40 (t, *J* = 6.6 Hz, 2H), 1.96 (s, 3H), 1.85 (p, *J* = 7.1 Hz, 2H), 1.65 (dtd, *J* = 16.0, 7.7, 4.7 Hz, 1H), 1.57 – 1.30 (m, 3H), 0.88 (t, *J* = 7.4 Hz, 3H). ^{13}C NMR (101 MHz, Chloroform-*d*) δ 169.98, 49.88, 33.85, 33.29, 29.21, 28.10, 23.41, 10.25. HRMS (EI): exact mass calculated for $C_8H_{16}BrNO$ $[M]^+$ require m/z = 221.0415, found 18a m/z = 221.0411, found 18b m/z = 221.0411.

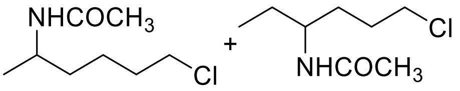

19a **19b**

6-bromo-*N*-(methoxymethyl)hexan-3-amine and *N*-(6-chlorohexan-3-yl)acetamide

Colorless oil, 63% yield. 9 F mol^{-1}. ^{1}H NMR (400 MHz, Chloroform-*d,* mixture of **19a** and **19b**) δ 6.04 – 5.58 (m, 2.31H), 3.99 – 3.86 (m, 1.31H), 3.80 (qt, J = 9.1, 5.0 Hz, 1H), 3.48 (dt, J = 10.2, 6.5 Hz, 4.63H), 1.93 (s, 3H), 1.90 (s, 3.86H), 1.80 -1.61 (m, 4.54H), 1.55-1.27 (m, 9.31H), 1.07 (d, J = 6.6 Hz, 3.87H), 0.85 (t, J = 7.4 Hz, 3H).^{13}C NMR (101 MHz, Chloroform-*d,* mixture of **19a** and **19b**) δ 170.03, 169.53, 49.92, 44.96(×2), 44.87, 35.97, 32.25, 31.99, 29.06, 28.05, 23.40, 23.33, 23.27, 20.81, 10.25. HRMS (EI): exact mass calculated for $C_8H_{16}ClNO$ $[M]^+$ require m/z = 177.0920, found m/z = 177.0998.

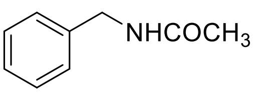

20

***N*-benzylacetamide**

White solid, 76% yield. 2.6 F mol^{-1}. ^{1}H NMR (400 MHz, Chloroform-*d*) δ 7.31 – 7.14 (m, 5H), 5.91 (s, 1H), 4.34 (d, *J* = 5.7 Hz, 2H), 1.93 (s, 3H). ^{13}C NMR (101 MHz, Chloroform-*d*) δ 170.40, 138.37, 128.60 (×2), 127.72 (×2), 127.37, 43.56, 23.02. HRMS (EI): exact mass calculated for $C_9H_{11}NO$ $[M]^+$ require m/z = 149.0841, found m/z = 149.0839.

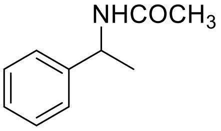

21

***N*-(1-phenylethyl)acetamide**

White solid, 71% yield. 2.6 F mol^{-1}. ^{1}H NMR (400 MHz, Chloroform-*d*) δ 7.33 – 7.17 (m, 5H), 6.44 – 6.25 (m, 1H), 5.05 (p, *J* = 7.2 Hz, 1H), 1.89 (s, 3H), 1.41 (d, *J* = 6.9 Hz, 3H). ^{13}C NMR (101 MHz, Chloroform-*d*) δ 169.85, 143.83, 128.48 (×2), 127.04, 126.17(×2), 48.80, 22.98, 22.05. HRMS (EI): exact mass calculated for $C_{10}H_{13}NO$ $[M]^+$ require m/z = 163.0997, found m/z = 163.0999.

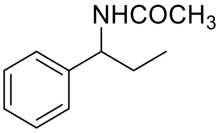

22

***N*-(1-phenylpropyl)acetamide**

White solid, 72% yield. 2.6 F mol^{-1}. ^{1}H NMR (400 MHz, Chloroform-*d*) δ 7.33 – 7.05 (m, 5H), 6.47 (d, *J* = 8.5 Hz, 1H), 4.76 (q, *J* = 7.7 Hz, 1H), 1.85 (s, 3H), 1.77 – 1.59 (m, 2H), 0.79 (t, *J* = 7.4 Hz, 3H). ^{13}C NMR (101 MHz, Chloroform-*d*) δ 169.78, 142.46, 128.51 (×2), 127.17, 126.68 (×2), 55.05, 29.21, 23.21,

10.86. HRMS (EI): exact mass calculated for $C_{11}H_{15}NO$ $[M]^+$ require m/z = 177.1154, found m/z = 177.1152.

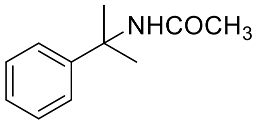

23

***N*-(2-phenylpropan-2-yl)acetamide**

White solid, 64% yield. 2.2 F mol^{-1}. ^1H NMR (400 MHz, Chloroform-*d*) δ 7.32 – 7.18 (m, 4H), 7.11 (t, *J* = 7.2 Hz, 1H), 6.12 (s, 1H), 1.81 (s, 3H), 1.56 (s, 6H). ^{13}C NMR (101 MHz, Chloroform-*d*) δ 169.40, 147.02, 128.32(×2), 126.52, 124.77(×2), 55.79, 29.21(×2), 24.15. HRMS (EI): exact mass calculated for $C_{11}H_{15}NO$ $[M]^+$ require m/z = 177.1154, found m/z = 177.1152.

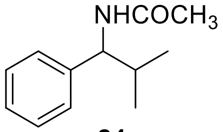

24

***N*-(2-methyl-1-phenylpropyl)acetamide**

White solid, 61% yield. 2.2 F mol^{-1}. ^1H NMR (400 MHz, Chloroform-*d*) δ 7.23 – 7.06 (m, 5H), 6.84 (d, *J* = 9.1 Hz, 1H), 4.63 (t, *J* = 8.6 Hz, 1H), 1.91 (dt, *J* = 14.1, 7.0 Hz, 1H), 1.85 (s, 3H), 0.87 (d, *J* = 6.7 Hz, 3H), 0.70 (d, *J* = 6.7 Hz, 3H). ^{13}C NMR (101 MHz, Chloroform-*d*) δ 169.83, 141.96, 128.31(×2), 127.13(×2), 126.96, 59.45, 33.39, 23.21, 19.84, 19.07. HRMS (EI): exact mass calculated for $C_{12}H_{17}NO$ $[M]^+$ require m/z = 191.1310, found m/z = 191.1307.

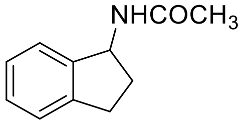

25

***N*-(2,3-dihydro-1*H*-inden-1-yl)acetamide**

White solid, 61% yield. 3.0 F mol^{-1}. ^1H NMR (400 MHz, Chloroform-*d*) δ 7.26 – 7.10 (m, 4H), 6.43 (d, *J* = 8.3 Hz, 1H), 5.34 (q, *J* = 7.8 Hz, 1H), 2.92 (ddd, *J* = 16.0, 8.7, 3.9 Hz, 1H), 2.79 (dt, *J* = 16.0, 8.1 Hz, 1H), 2.47 (dtd, *J* = 12.0, 7.8, 3.9 Hz, 1H), 1.93 (s, 3H), 1.75 (dq, *J* = 12.8, 8.1 Hz, 1H). ^{13}C NMR (101 MHz, Chloroform-*d*) δ 169.13, 142.23, 142.20, 126.76, 125.56, 123.64, 122.95, 53.54, 32.74, 29.14, 22.08. HRMS (EI): exact mass calculated for $C_{11}H_{13}NO$ $[M]^+$ require m/z = 175.0997, found m/z = 175.0995.

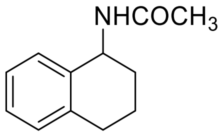

26

***N*-(1,2,3,4-tetrahydronaphthalen-1-yl)acetamide**

White solid, 73% yield. 2.6 F mol^{-1}. ^1H NMR (400 MHz, Chloroform-*d*) δ 7.22 – 7.15 (m, 1H), 7.14 – 7.07 (m, 2H), 7.07 – 7.00 (m, 1H), 6.45 (d, *J* = 8.6 Hz, 1H), 5.10 – 5.01 (m, 1H), 2.84 – 2.63 (m, 2H), 1.94 (td, *J* = 8.3, 7.7, 5.5 Hz, 1H), 1.90 (s, 3H), 1.86 – 1.70 (m, 3H). ^{13}C NMR (101 MHz, Chloroform-*d*) δ 169.64, 137.46, 136.78, 129.07, 128.64, 127.13, 126.11, 47.38, 30.10, 29.24, 23.20, 20.03. HRMS (EI): exact mass calculated for $C_{12}H_{15}NO$ $[M]^+$ require m/z = 189.1154, found m/z = 189.1155.

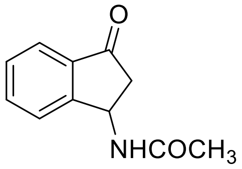

27

***N*-(3-oxo-2,3-dihydro-1*H*-inden-1-yl)acetamide**

Colorless oil, 74% yield. 3.0 F mol^{-1}. ^{1}H NMR (400 MHz, Chloroform-*d*) δ 7.64 – 7.49 (m, 3H), 7.42 – 7.33 (m, 2H), 5.45 (d, *J* = 3.8 Hz, 1H), 2.99 (dd, *J* = 19.1, 7.7 Hz, 1H), 2.42 (dd, *J* = 19.1, 3.7 Hz, 1H), 1.95 (s, 3H). ^{13}C NMR (101 MHz, Chloroform-*d*) δ 203.68, 170.57, 154.26, 136.32, 135.35, 128.98, 126.05, 123.01, 47.21, 44.32 (d, *J* = 2.8 Hz), 22.89 (d, *J* = 1.9 Hz). HRMS (EI): exact mass calculated for $C_{11}H_{11}NO_2$ $[M]^+$ require m/z = 189.0790, found m/z = 189.0792.

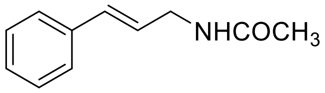

28

***N*-cinnamylacetamide**

Colorless oil, 41% yield. 2.6 F mol^{-1}. ^{1}H NMR (400 MHz, Chloroform-*d*) δ 7.32 – 7.13 (m, 5H), 6.45 (d, *J* = 16.1 Hz, 1H), 6.12 (dt, *J* = 15.9, 6.4 Hz, 1H), 5.70 – 5.49 (m, 1H), 3.97 (td, *J* = 6.1, 1.5 Hz, 2H), 1.96 (s, 3H). ^{13}C NMR (101 MHz, Chloroform-*d*) δ 170.23, 136.56, 131.99, 128.60 (×2), 127.71, 126.35 (×2), 125.61, 41.67, 23.20. HRMS (EI): exact mass calculated for $C_{11}H_{13}NO$ $[M]^+$ require m/z = 175.0997, found m/z = 175.0996.

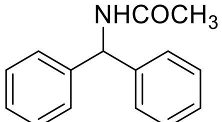

29

***N*-benzhydrylacetamide**

White solid, 86% yield. 2.2 F mol^{-1}. ^{1}H NMR (400 MHz, Chloroform-*d*) δ 7.25 – 7.02 (m, 11H), 6.06 (d, *J* = 8.2 Hz, 1H), 1.74 (s, 3H). ^{13}C NMR (101 MHz, Chloroform-*d*) δ 169.76, 141.77(×2), 128.61(×4), 127.57 (×4), 127.37(×2), 57.00, 22.97. HRMS (EI): exact mass calculated for $C_{15}H_{15}NO$ $[M]^+$ require m/z = 225.1154, found m/z = 225.1156.

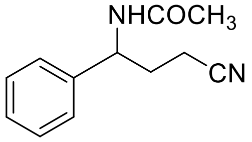

30

***N*-(3-cyano-1-phenylpropyl)acetamide**

White solid, 55% yield. 2.6 F mol^{-1}. ^{1}H NMR (400 MHz, Chloroform-*d*) δ 7.33 – 7.13 (m, 5H), 7.06 (d, *J* = 9.2 Hz, 1H), 4.91 (td, *J* = 8.3, 6.4 Hz, 1H), 2.28 – 2.14 (m, 2H), 2.14 – 1.92 (m, 2H), 1.87 (s, 3H). ^{13}C NMR (101 MHz, Chloroform-*d*) δ 170.33, 140.56, 128.95 (×2), 127.96, 126.52 (×2), 119.43, 52.65, 31.70, 23.10, 14.49. HRMS (EI): exact mass calculated for $C_{12}H_{14}N_2O$ $[M]^+$ require m/z = 202.1106, found m/z = 202.1108.

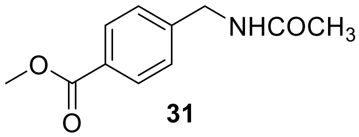

31

methyl 4-(acetamidomethyl)benzoate

White solid, 65% yield. 3.0 F mol^{-1}. ^{1}H NMR (400 MHz, Chloroform-*d*) δ 7.88 (d, *J* = 8.0 Hz, 2H), 7.23 (d, *J* = 8.0 Hz, 2H), 6.76 (s, 1H), 4.36 (d, *J* = 5.9 Hz, 2H), 3.84 (s, 3H), 1.95 (s, 3H). ^{13}C NMR (101 MHz, Chloroform-*d*) δ 170.47, 166.87, 143.75, 129.82 (×2), 129.03, 127.39 (×2), 52.10, 43.13, 23.02. HRMS (EI): exact mass calculated for $C_{11}H_{13}NO_3$ $[M]^+$ require m/z = 207.0895, found m/z = 207.0893.

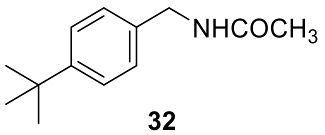

32

***N*-(4-(tert-butyl)benzyl)acetamide**

White solid, 63% yield. 3.0 F mol^{-1}. ^{1}H NMR (400 MHz, Chloroform-*d*) δ 7.33 (d, *J* = 8.3 Hz, 2H), 7.19 (d, *J* = 8.1 Hz, 2H), 6.43 (s, 1H), 4.33 (d, *J* = 5.7 Hz, 2H), 1.95 (s, 3H), 1.29 (d, *J* = 1.1 Hz, 9H). ^{13}C NMR (101 MHz, Chloroform-*d*) δ 169.27, 149.38, 134.20, 126.60 (×2), 124.51(×2), 42.32, 33.44, 30.30 (×3), 22.01. HRMS (EI): exact mass calculated for $C_{13}H_{19}NO$ $[M]^+$ require m/z = 205.1467, found m/z = 205.1465.

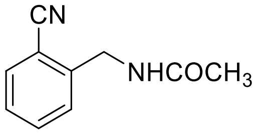

33

***N*-(2-cyanobenzyl)acetamide**

White solid, 67% yield. 2.6 F mol^{-1}. ^{1}H NMR (400 MHz, Chloroform-*d*) δ 7.63 – 7.51 (m, 2H), 7.47 (d, *J* = 7.8 Hz, 1H), 7.40 – 7.29 (m, 1H), 7.05 (d, *J* = 7.5 Hz, 1H), 4.54 (d, *J* = 6.1 Hz, 2H), 2.01 (s, 3H). ^{13}C NMR (101 MHz, Chloroform-*d*) δ 170.74, 142.31, 133.13, 132.80, 128.94, 127.82, 117.51, 111.26, 41.70, 22.89. HRMS (EI): exact mass calculated for $C_{10}H_{10}N_2O$ $[M]^+$ require m/z = 174.0793, found m/z = 174.0791.

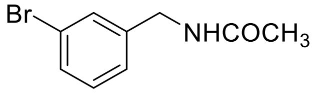

34

***N*-(3-bromobenzyl)acetamide**

Light yellow solid, 64% yield. 2.6 F mol^{-1}. ^{1}H NMR (400 MHz, Chloroform-*d*) δ 7.41 – 7.33 (m, 2H), 7.19 – 7.12 (m, 2H), 6.32 (s, 1H), 4.34 (d, *J* = 5.9 Hz, 2H), 1.99 (s, 3H). ^{13}C NMR (101 MHz, Chloroform-*d*) δ 170.31, 140.71, 130.63, 130.51, 130.23, 126.33, 122.66, 42.96, 23.13. HRMS (EI): exact mass calculated for $C_9H_{10}BrNO$ $[M]^+$ require m/z = 226.9946, found m/z = 226.9944.

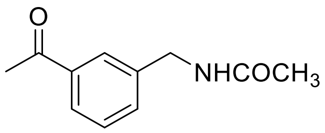

35

***N*-(3-acetylbenzyl)acetamide**

White solid, 60% yield. 2.2 F mol^{-1}. ^{1}H NMR (400 MHz, Chloroform-*d*) δ 7.82 (dd, *J* = 6.8, 1.5 Hz, 2H), 7.48 (d, *J* = 7.9 Hz, 1H), 7.41 (t, *J* = 8.0 Hz, 1H), 6.20 (s, 1H), 4.45 (d, *J* = 5.9 Hz, 2H), 2.57 (s, 3H), 2.02

(s, 3H). ^{13}C NMR (101 MHz, Chloroform-*d*) δ 198.15, 170.30, 139.04, 137.43, 132.57, 128.99, 127.56, 127.30, 43.33, 26.70, 23.19. HRMS (EI): exact mass calculated for $C_{11}H_{13}NO_2$ $[M]^+$ require m/z = 191.0946, found m/z = 191.0944.

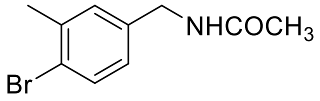

36

***N*-(4-bromo-3-methylbenzyl)acetamide**

Light yellow solid, 63% yield. 3.0 F mol^{-1}. ^{1}H NMR (400 MHz, Chloroform-*d*) δ 7.42 (d, *J* = 8.1 Hz, 1H), 7.09 (d, *J* = 2.2 Hz, 1H), 6.90 (dd, *J* = 8.2, 2.2 Hz, 1H), 6.36 (s, 1H), 4.26 (d, *J* = 5.8 Hz, 2H), 2.33 (s, 3H), 1.96 (s, 3H). ^{13}C NMR (101 MHz, Chloroform-*d*) δ 170.32, 138.09, 137.65, 132.46, 130.19, 126.65, 123.67, 42.92, 23.09, 22.86. HRMS (EI): exact mass calculated for $C_{10}H_{12}BrNO$ $[M]^+$ require m/z = 241.0102, found m/z = 241.0101.

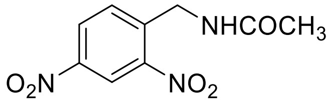

37

***N*-(2,4-dinitrobenzyl)acetamide**

White solid, 70% yield. 3.0 F mol^{-1}. ^{1}H NMR (400 MHz, Chloroform-*d*) δ 8.77 (s, 1H), 8.37 (d, *J* = 8.3 Hz, 1H), 7.83 (d, *J* = 8.4 Hz, 1H), 7.03 (t, *J* = 5.9 Hz, 1H), 4.70 (d, *J* = 6.0 Hz, 2H), 1.99 (s, 3H). ^{13}C NMR (101 MHz, Chloroform-*d*) δ 171.13, 148.00, 147.02, 140.70, 132.75, 127.73, 120.44, 40.99, 22.89. HRMS (EI): exact mass calculated for $C_9H_9N_3O_5$ $[M]^+$ require m/z = 239.0542, found m/z = 239.0540.

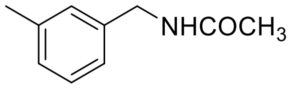

38

***N*-(3-methylbenzyl)acetamide**

White solid, 68% yield. 3.0 F mol^{-1}. ^{1}H NMR (400 MHz, Chloroform-*d*) δ 7.19 (t, *J* = 7.8 Hz, 1H), 7.09 – 7.00 (m, 3H), 6.41 (s, 1H), 4.31 (d, *J* = 5.7 Hz, 2H), 2.31 (s, 3H), 1.95 (s, 3H). ^{13}C NMR (101 MHz, Chloroform-*d*) δ 170.29, 138.28 (d, *J* = 6.3 Hz) (×2), 128.55 (×2), 128.17, 124.81, 43.61, 23.09, 21.36. HRMS (EI): exact mass calculated for $C_{10}H_{13}NO$ $[M]^+$ require m/z = 163.0097, found m/z = 163.0095.

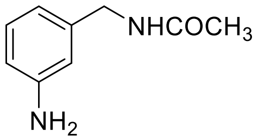

39

***N*-(4-aminobenzyl)acetamide**

Yellow oil, 25% yield. 2.6 F mol^{-1}. ^{1}H NMR (400 MHz, Chloroform-*d*) δ 7.07 (d, *J* = 8.3 Hz, 2H), 6.69 – 6.61 (m, 2H), 5.64 (s, 1H), 4.29 (d, *J* = 5.5 Hz, 2H), 1.99 (s, 3H). ^{13}C NMR (101 MHz, Chloroform-*d*) δ 169.79, 145.94, 129.26(×2), 127.98, 115.23(×2), 43.48, 23.34. HRMS (EI): exact mass calculated for $C_9H_{12}N_2O$ $[M]^+$ require m/z = 164.0950, found m/z = 164.0952.

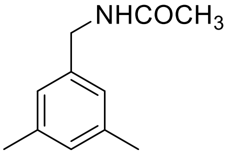

40

***N*-(3,5-dimethylbenzyl)acetamide**

Colorless oil, 64% yield. 2.6 F mol^{-1}. ^{1}H NMR (400 MHz, Chloroform-*d*) δ 6.87 (d, *J* = 10.5 Hz, 3H), 6.36 (s, 1H), 4.28 (d, *J* = 5.7 Hz, 2H), 2.28 (s, 6H), 1.96 (s, 3H). ^{13}C NMR (101 MHz, Chloroform-*d*) δ 170.19, 138.22 (×2), 138.17, 129.13, 125.63(×2), 43.60, 22.90, 21.23 (×2). HRMS (EI): exact mass calculated for $C_{11}H_{15}NO$ $[M]^+$ require m/z = 177.1154, found m/z = 177.1152.

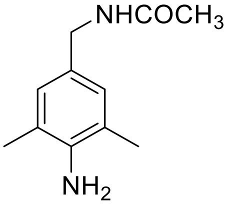

41

***N*-(4-amino-3,5-dimethylbenzyl)acetamide**

Yellow oil, 54% yield. 3.0 F mol^{-1}. ^{1}H NMR (400 MHz, Chloroform-*d*) δ 6.83 (s, 2H), 6.08 (s, 1H), 4.21 (d, *J* = 5.4 Hz, 2H), 3.64 (s, 2H), 2.14 (s, 6H), 1.94 (s, 3H). ^{13}C NMR (101 MHz, Chloroform-*d*) δ 170.05, 142.18, 128.07(×2), 127.37, 121.85(×2), 43.44, 23.15, 17.59(×2). HRMS (EI): exact mass calculated for $C_{11}H_{16}N_2O$ $[M]^+$ require m/z = 192.1263, found m/z = 192.1261.

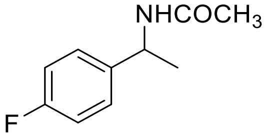

42

***N*-(1-(4-fluorophenyl)ethyl)acetamide**

White solid, 64% yield. 3.0 F mol^{-1}. ^{1}H NMR (400 MHz, Chloroform-*d*) δ 7.30 – 7.21 (m, 2H), 7.03 – 6.92 (m, 2H), 6.57 (d, *J* = 8.0 Hz, 1H), 5.05 (p, *J* = 7.1 Hz, 1H), 1.93 (s, 3H), 1.43 (d, *J* = 7.0 Hz, 3H). ^{13}C NMR (101 MHz, Chloroform-*d*) δ 169.53, 161.84 (d, *J* = 245.0 Hz), 139.37 (d, *J* = 3.1 Hz), 127.76 (d, *J* = 8.0 Hz), 115.26 (d, *J* = 21.3 Hz), 48.14, 23.14, 21.90. HRMS (EI): exact mass calculated for $C_{10}H_{12}FNO$ $[M]^+$ require m/z = 181.0903, found m/z = 181.0901.

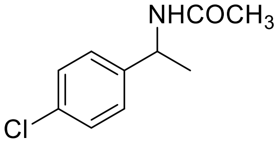

43

***N*-(1-(4-chlorophenyl)ethyl)acetamide**

White solid, 71% yield. 2.2 F mol^{-1}. ^{1}H NMR (400 MHz, Chloroform-*d*) δ 7.18 – 7.06 (m, 4H), 6.86 (d, *J* = 7.8 Hz, 1H), 4.91 (p, *J* = 7.1 Hz, 1H), 1.82 (s, 3H), 1.30 (d, *J* = 7.0 Hz, 3H).^{13}C NMR (101 MHz, Chloroform-*d*) δ 168.63, 141.18, 131.69, 127.55 (×2), 126.51(×2), 47.20, 22.06, 20.83. HRMS (EI): exact mass calculated for $C_{10}H_{12}ClNO$ $[M]^+$ require m/z = 197.0607, found m/z = 197.0609.

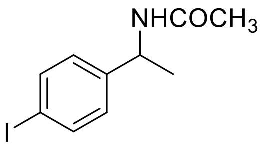

44

***N*-(1-(4-iodophenyl)ethyl)acetamide**

White solid, 77% yield. 2.2 F mol^{-1}. ^{1}H NMR (400 MHz, Chloroform-*d*) δ 7.58 (d, *J* = 8.4 Hz, 2H), 6.99 (d, *J* = 8.1 Hz, 2H), 6.81 (d, *J* = 7.9 Hz, 1H), 4.94 (p, *J* = 7.1 Hz, 1H), 1.88 (s, 3H), 1.36 (d, *J* = 7.0 Hz, 3H). ^{13}C NMR (101 MHz, Chloroform-*d*) δ 169.66, 143.42, 137.54 (×2), 128.21 (×2), 92.48, 48.40, 23.19, 21.88. HRMS (EI): exact mass calculated for $C_{10}H_{12}INO$ $[M]^+$ require m/z = 288.9964, found m/z = 288.9962.

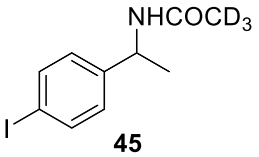

45

N*-(1-(4-iodophenyl)ethyl)acetamide-2,2,2-*d₃

White solid, 75% yield. 4.4 F mol^{-1}. ^{1}H NMR (600 MHz, Chloroform-d) δ 7.61 (ddd, J = 8.4, 5.3, 1.9 Hz, 2H), 7.03 (td, J = 6.4, 5.3, 2.8 Hz, 2H), 6.87 (d, J = 7.2 Hz, 1H), 5.00 – 4.93 (m, 1H), 1.42 – 1.34 (m, 3H). ^{13}C NMR (151 MHz, Chloroform-d) δ 169.64, 143.41, 137.55(×2), 128.21(×2), 92.46, 48.36, 22.47 (dp, J = 39.2, 19.7 Hz), 21.85. HRMS (EI): exact mass calculated for $C_{10}H_{9}D_{3}INO$ $[M]^+$ require m/z = 292.0152, found m/z = 292.0155.

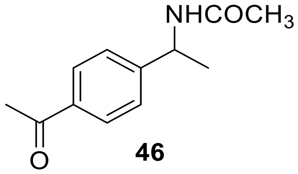

46

***N*-(1-(4-acetylphenyl)ethyl)acetamide**

White solid, 73% yield. 3.0 F mol^{-1}. ^{1}H NMR (400 MHz, Chloroform-*d*) δ 7.85 (dt, *J* = 8.4, 2.2 Hz, 2H), 7.43 – 7.30 (m, 2H), 6.60 (s, 1H), 5.16 – 5.03 (m, 1H), 2.54 (t, *J* = 1.9 Hz, 3H), 1.95 (t, *J* = 2.1 Hz, 3H), 1.42 (dt, *J* = 7.1, 2.3 Hz, 3H). ^{13}C NMR (101 MHz, Chloroform-*d*) δ 196.98, 168.73, 148.33, 134.85, 127.66 (×2), 125.27 (×2), 47.66, 25.63, 22.09, 20.89. HRMS (EI): exact mass calculated for $C_{12}H_{15}NO_2$ $[M]^+$ require m/z = 205.1103, found m/z = 205.1105.

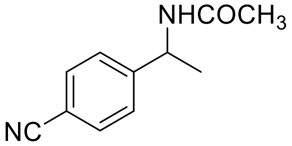

47

***N*-(1-(4-cyanophenyl)ethyl)acetamide**

White solid, 85% yield. 2.2 F mol^{-1}. ^{1}H NMR (400 MHz, Chloroform-*d*) δ 7.56 (d, *J* = 8.2 Hz, 2H), 7.38 (d, *J* = 8.2 Hz, 2H), 6.52 (d, *J* = 7.6 Hz, 1H), 5.05 (p, *J* = 7.1 Hz, 1H), 1.94 (s, 3H), 1.41 (d, *J* = 7.1 Hz, 3H). ^{13}C NMR (101 MHz, Chloroform-*d*) δ 169.70, 149.31, 132.41 (×2), 126.88 (×2), 118.84, 110.73, 48.74, 23.11, 21.80. HRMS (EI): exact mass calculated for $C_{11}H_{12}N_{2}O$ $[M]^+$ require m/z = 188.0950, found m/z = 188.0952.

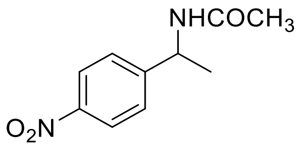

48

***N*-(1-(4-nitrophenyl)ethyl)acetamide**

White solid, 87% yield. 2.2 F mol^{-1}. ^{1}H NMR (400 MHz, Chloroform-*d*) δ 8.10 (d, *J* = 8.5 Hz, 2H), 7.42 (d, *J* = 8.5 Hz, 2H), 6.68 (d, *J* = 7.4 Hz, 1H), 5.09 (p, *J* = 7.1 Hz, 1H), 1.96 (s, 3H), 1.43 (d, *J* = 7.1 Hz, 3H). ^{13}C NMR (101 MHz, Chloroform-*d*) δ 169.89, 151.43, 146.86, 126.93 (×2), 123.79 (×2), 48.64, 23.06, 21.84. HRMS (EI): exact mass calculated for $C_{10}H_{12}N_2O_3$ $[M]^+$ require m/z = 208.0848, found m/z = 208.0846.

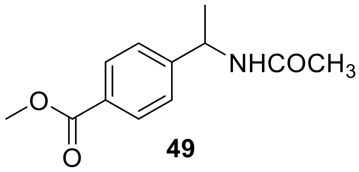

49

methyl 4-(acetamidomethyl)benzoate

White solid, 73% yield. 2.6 F mol^{-1}. ^{1}H NMR (400 MHz, Chloroform-d) δ 8.03 – 7.91 (m, 2H), 7.38 – 7.31 (m, 2H), 6.91 (d, J = 7.8 Hz, 1H), 5.10 (p, J = 7.2 Hz, 1H), 3.89 (s, 3H), 1.96 (s, 3H), 1.43 (d, J = 7.0 Hz, 3H). ^{13}C NMR (101 MHz, Chloroform-d) δ 169.68, 166.90, 148.99, 129.82 (×2), 128.82, 126.06 (×2), 52.05, 48.59, 23.06, 21.83. HRMS (EI): exact mass calculated for $C_{12}H_{15}NO_3$ $[M]^+$ require m/z = 221.1052, found m/z = 221.1052.

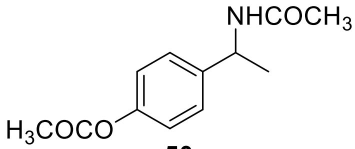

50

4-(1-acetamidoethyl)phenyl acetate

Colorless oil, 71% yield. 3.0 F mol^{-1}. ^{1}H NMR (400 MHz, Chloroform-*d*) δ 7.26 – 7.19 (m, 2H), 6.98 – 6.90 (m, 2H), 6.25 (d, *J* = 8.0 Hz, 1H), 5.01 (p, *J* = 7.1 Hz, 1H), 2.21 (s, 3H), 1.84 (s, 3H), 1.35 (d, *J* = 7.0 Hz, 3H). ^{13}C NMR (101 MHz, Chloroform-*d*) δ 168.66, 168.51, 148.66, 139.88 (×2), 126.32 (×2), 120.62, 47.19, 22.18, 20.61, 20.09. HRMS (EI): exact mass calculated for $C_{12}H_{15}NO_3$ $[M]^+$ require m/z = 221.1052, found m/z = 221.1054.

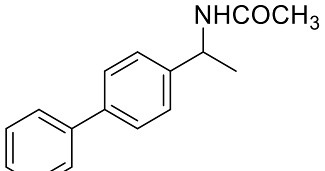

51

***N*-(1-([1,1'-biphenyl]-4-yl)ethyl)acetamide**

White solid, 74% yield. 2.6 F mol^{-1}. ^{1}H NMR (400 MHz, Chloroform-*d*) δ 7.61 – 7.52 (m, 4H), 7.47 – 7.31 (m, 5H), 6.00 (s, 1H), 5.17 (p, *J* = 7.1 Hz, 1H), 1.99 (s, 3H), 1.52 (d, *J* = 6.9 Hz, 3H). ^{13}C NMR (101 MHz, Chloroform-*d*) δ 168.30, 141.27, 139.67, 139.22, 127.74 (×2), 126.32 (×2), 126.26, 126.01(×2), 125.60 (×2), 47.48, 22.34, 20.72. HRMS (EI): exact mass calculated for $C_{16}H_{17}NO$ $[M]^+$ require m/z = 239.1310, found m/z = 239.1312.

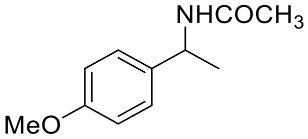

52

***N*-(1-(4-methoxyphenyl)ethyl)acetamide**

Colorless oil, 62% yield. 3.0 F mol^{-1}. ^{1}H NMR (400 MHz, Chloroform-d) δ 7.27 – 7.07 (m, 2H), 6.82 – 6.74 (m, 2H), 5.83 (d, J = 7.4 Hz, 1H), 4.99 (p, J = 7.1 Hz, 1H), 3.71 (s, 3H), 1.88 (s, 3H), 1.38 (d, J = 6.9 Hz, 3H). ^{13}C NMR (101 MHz, Chloroform-d) δ 168.14, 157.77, 134.31, 126.38(×2), 112.95(×2), 54.27, 47.17, 22.39, 20.59. HRMS (EI): exact mass calculated for $C_{11}H_{15}NO_2$ $[M]^+$ require m/z = 193.1103, found m/z = 193.1105.

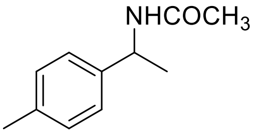

53

***N*-(1-(p-tolyl)ethyl)acetamide**

White solid, 73% yield. 2.6 F mol^{-1}. ^{1}H NMR (400 MHz, Chloroform-*d*) δ 7.24 – 7.07 (m, 4H), 6.35 – 6.14 (m, 1H), 5.06 (p, *J* = 7.1 Hz, 1H), 2.32 (s, 3H), 1.94 (s, 3H), 1.45 (d, *J* = 6.9 Hz, 3H). ^{13}C NMR (101 MHz, Chloroform-*d*) δ 169.37, 140.34, 136.94, 129.29(×2), 126.15(×2), 48.55, 23.32, 21.77, 21.05. HRMS (EI): exact mass calculated for $C_{11}H_{15}NO$ $[M]^+$ require m/z = 177.1154, found m/z = 177.1156.

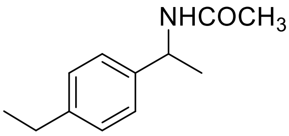

54

***N*-(1-(4-ethylphenyl)ethyl)acetamide**

White solid, 65% yield. 2.6 F mol^{-1}. ^{1}H NMR (400 MHz, Chloroform-*d*) δ 7.23 (d, *J* = 8.2 Hz, 2H), 7.16 (d, *J* = 8.2 Hz, 2H), 5.98 (s, 1H), 5.08 (p, *J* = 7.1 Hz, 1H), 2.62 (q, *J* = 7.6 Hz, 2H), 1.95 (s, 3H), 1.46 (d, *J* = 6.9 Hz, 3H), 1.22 (t, *J* = 7.6 Hz, 3H). ^{13}C NMR (101 MHz, Chloroform-*d*) δ 169.37, 143.27, 140.59, 128.08 (×2), 126.22 (×2), 48.54, 28.47, 23.30, 21.78, 15.60. HRMS (EI): exact mass calculated for $C_{12}H_{17}NO$ $[M]^+$ require m/z = 191.1310, found m/z = 191.1312.

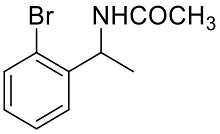

55

***N*-(1-(2-bromophenyl)ethyl)acetamide**

Light yellow solid, 74% yield. 2.6 F mol^{-1}. ^{1}H NMR (400 MHz, Chloroform-*d*) δ 7.54 (dd, *J* = 8.0, 1.2 Hz, 1H), 7.36 – 7.25 (m, 2H), 7.10 (ddd, *J* = 7.9, 6.9, 2.2 Hz, 1H), 6.07 (d, *J* = 7.5 Hz, 1H), 5.34 (p, *J* = 7.1 Hz, 1H), 1.98 (s, 3H), 1.46 (d, *J* = 6.9 Hz, 3H). ^{13}C NMR (101 MHz, Chloroform-*d*) δ 168.72, 141.86, 131.96, 127.41, 126.70, 125.81, 121.69, 47.92, 21.97, 20.07. HRMS (EI): exact mass calculated for $C_{10}H_{12}BrNO$ $[M]^+$ require m/z = 241.0102, found m/z = 241.0104.

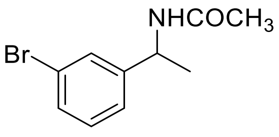

56

***N*-(1-(3-bromophenyl)ethyl)acetamide**

Light yellow solid, 69% yield. 2.6 F mol^{-1}. ^{1}H NMR (400 MHz, Chloroform-*d*) δ 7.40 (s, 1H), 7.31 (d, *J* = 7.6 Hz, 1H), 7.14 (dt, *J* = 15.4, 7.9 Hz, 2H), 6.89 (d, *J* = 7.9 Hz, 1H), 4.97 (p, *J* = 7.2 Hz, 1H), 1.90 (s, 3H), 1.37 (d, *J* = 7.0 Hz, 3H). ^{13}C NMR (101 MHz, Chloroform-*d*) δ 169.73, 146.12, 130.18, 130.16, 129.15, 124.95, 122.60, 48.43, 23.12, 21.94. HRMS (EI): exact mass calculated for $C_{10}H_{12}BrNO$ $[M]^+$ require m/z = 241.0102, found m/z = 241.0104.

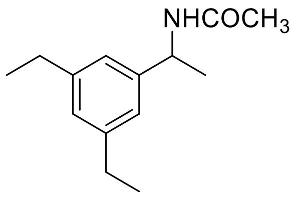

57

***N*-(1-(3,5-diethylphenyl)ethyl)acetamide**

White solid, 72% yield. 2.6 F mol^{-1}. ^{1}H NMR (400 MHz, Chloroform-*d*) δ 6.96 (dd, *J* = 8.6, 1.8 Hz, 3H), 6.23 (d, *J* = 8.1 Hz, 1H), 5.08 (p, *J* = 7.1 Hz, 1H), 2.62 (q, *J* = 7.6 Hz, 4H), 1.95 (s, 3H), 1.47 (d, *J* = 6.9 Hz, 3H), 1.23 (t, *J* = 7.6 Hz, 6H).^{13}C NMR (101 MHz, Chloroform-*d*) δ 169.25, 144.61 (×2), 143.31, 126.49, 123.20 (×2), 48.91, 28.87 (×2), 23.34, 21.90, 15.61(×2). HRMS (EI): exact mass calculated for $C_{14}H_{21}NO$ $[M]^+$ require m/z = 219.1623, found m/z = 216.1625.

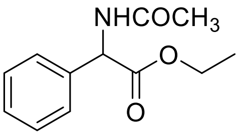

58

ethyl 2-acetamido-2-phenylacetate

Light yellow oil, 72% yield. 3.0 F mol^{-1}. ^{1}H NMR (400 MHz, Chloroform-*d*) δ 7.24 (dq, *J* = 11.6, 6.2, 4.8 Hz, 5H), 6.87 (d, *J* = 7.4 Hz, 1H), 5.48 (t, *J* = 7.2 Hz, 1H), 4.22 – 3.95 (m, 2H), 1.90 (d, *J* = 7.0 Hz, 3H), 1.10 (q, *J* = 7.1 Hz, 3H). ^{13}C NMR (101 MHz, Chloroform-*d*) δ 170.06, 168.70, 135.60, 127.85 (×2), 127.39, 126.25 (×2), 60.81, 55.46, 21.84, 12.94. HRMS (EI): exact mass calculated for $C_{12}H_{15}NO_3$ $[M]^+$ require m/z = 221.1052, found m/z = 221.1054.

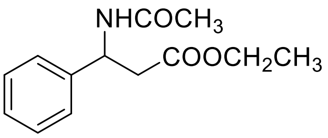

59

ethyl 3-acetamido-3-phenylpropanoate

Light yellow oil, 65% yield. 3.0 F mol^{-1}. ^{1}H NMR (400 MHz, Chloroform-*d*) δ 7.38 – 7.19 (m, 5H), 7.07 (d, *J* = 8.4 Hz, 1H), 5.40 (dt, *J* = 8.3, 6.4 Hz, 1H), 4.05 (q, *J* = 7.2 Hz, 2H), 2.88 (dd, *J* = 15.4, 6.5 Hz, 1H), 2.77 (dd, *J* = 15.5, 6.3 Hz, 1H), 1.95 (s, 3H), 1.15 (t, *J* = 7.2 Hz, 3H). ^{13}C NMR (101 MHz, Chloroform-*d*) δ 171.07, 169.55, 140.76, 128.59 (×2), 127.52, 126.40 (×2), 60.67, 49.78, 40.28, 23.16, 14.03. HRMS (EI): exact mass calculated for $C_{13}H_{17}NO_3$ $[M]^+$ require m/z = 235.1208, found m/z = 235.1210.

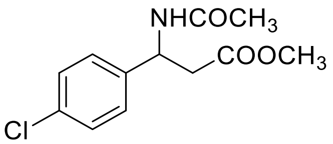

60

methyl 3-acetamido-3-(4-chlorophenyl)propanoate

Light yellow oil, 74% yield. 3.0 F mol^{-1}. ^{1}H NMR (400 MHz, Chloroform-*d*) δ 7.39 (d, *J* = 8.3 Hz, 1H), 7.23 – 7.14 (m, 4H), 5.30 (dt, *J* = 8.4, 6.6 Hz, 1H), 3.54 (s, 3H), 2.79 (dd, *J* = 15.8, 6.7 Hz, 1H), 2.69 (dd, *J* = 15.8, 6.4 Hz, 1H), 1.88 (s, 3H). ^{13}C NMR (101 MHz, Chloroform-*d*) δ 171.11, 169.90, 139.65, 133.15, 128.67(×2), 127.91(×2), 51.79, 49.37, 40.05, 22.97. HRMS (EI): exact mass calculated for $C_{12}H_{14}ClNO_3$ $[M]^+$ require m/z = 255.0662, found m/z = 255.0660.

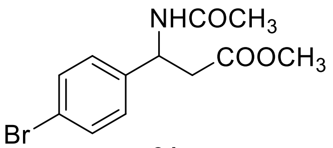

61

methyl 3-acetamido-3-(4-bromophenyl)propanoate

Light yellow oil, 68% yield. 2.2 F mol^{-1}. ^{1}H NMR (400 MHz, Chloroform-*d*) δ 7.40 (d, *J* = 8.3 Hz, 2H), 7.13 (d, *J* = 8.3 Hz, 2H), 7.01 (d, *J* = 8.4 Hz, 1H), 5.32 (dt, *J* = 8.5, 6.2 Hz, 1H), 3.58 (s, 3H), 2.84 (dd, *J* = 15.9, 6.2 Hz, 1H), 2.75 (dd, *J* = 15.9, 6.1 Hz, 1H), 1.95 (s, 3H). ^{13}C NMR (101 MHz, Chloroform-*d*) δ 171.40, 169.62, 139.81, 131.74 (×2), 128.12 (×2), 121.45, 51.92, 49.15, 39.66, 23.23. HRMS (EI): exact mass calculated for $C_{12}H_{14}BrNO_3$ $[M]^+$ require m/z = 299.0157, found m/z = 299.0155.

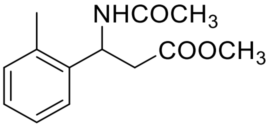

62

methyl 3-acetamido-3-(o-tolyl)propanoate

Light yellow oil, 62% yield. 2.6 F mol^{-1}. ^{1}H NMR (400 MHz, Chloroform-*d*) δ 7.14 (d, *J* = 8.2 Hz, 2H), 7.12 – 7.04 (m, 3H), 5.33 (dt, *J* = 8.4, 6.5 Hz, 1H), 3.56 (s, 3H), 2.85 (dd, *J* = 15.5, 6.6 Hz, 1H), 2.73 (dd, *J* = 15.5, 6.4 Hz, 1H), 2.27 (s, 3H), 1.90 (s, 3H). ^{13}C NMR (101 MHz, Chloroform-*d*) δ 171.51, 169.67, 137.83, 137.15, 129.28(×2), 126.33(×2), 51.70, 49.59, 40.14, 23.09, 21.01. HRMS (EI): exact mass calculated for $C_{13}H_{17}NO_3$ $[M]^+$ require m/z = 235.1208, found m/z = 235.1210.

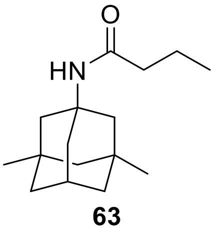

63

***N*-(3,5-dimethyladamantan-1-yl)butyramide**

White solid, 85% yield. 4.4 F mol^{-1}. ^{1}H NMR (400 MHz, Chloroform-*d*) δ 5.35 (s, 1H), 2.07 (hept, *J* = 3.2 Hz, 1H), 2.00 (t, *J* = 7.4 Hz, 2H), 1.77 (d, *J* = 3.2 Hz, 2H), 1.63 – 1.51 (m, 6H), 1.31 (dt, *J* = 12.2, 2.6 Hz, 2H), 1.26 – 1.19 (m, 2H), 1.15 – 1.02 (m, 2H), 0.86 (t, *J* = 7.4 Hz, 3H), 0.78 (s, 6H). ^{13}C NMR (101 MHz, Chloroform-*d*) δ 172.32, 53.26, 50.59, 47.58 (×2), 42.66 (×2), 40.16, 39.55, 32.30 (×2), 30.07 (×2), 19.18, 17.85, 13.63. HRMS (EI): exact mass calculated for $C_{16}H_{27}NO$ $[M]^+$ require m/z = 249.2093, found m/z = 249.2095.

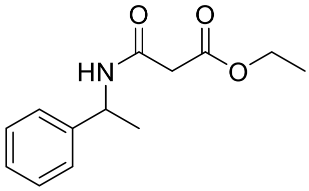

64

ethyl 3-oxo-3-((1-phenylethyl)amino)propanoate

White solid, 85% yield. 3.0 F mol^{-1}. ^{1}H NMR (400 MHz, Chloroform-*d*) δ 7.61 (d, *J* = 8.0 Hz, 1H), 7.30 (d, *J* = 4.4 Hz, 4H), 7.22 (dt, *J* = 8.7, 4.0 Hz, 1H), 5.10 (p, *J* = 7.1 Hz, 1H), 4.16 (q, *J* = 7.1 Hz, 2H), 3.25 (d, *J* = 2.2 Hz, 2H), 1.47 (d, *J* = 7.0 Hz, 3H), 1.25 (t, *J* = 7.2 Hz, 3H). ^{13}C NMR (101 MHz, Chloroform-d) δ 169.50, 164.30, 143.08, 128.65(×2), 127.32, 126.08(×2), 61.54, 48.95, 41.27, 22.06, 14.04. HRMS (EI): exact mass calculated for $C_{13}H_{17}NO_3$ $[M]^+$ require m/z = 235.1208, found m/z = 235.1210.

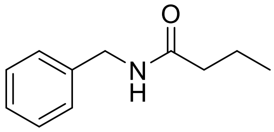

65

***N*-benzylbutyramide**

White solid, 69% yield. 2.6 F mol^{-1}. ^{1}H NMR (400 MHz, Chloroform-*d*) δ 7.38 – 7.03 (m, 5H), 6.34 (s, 1H), 4.28 (d, *J* = 5.8 Hz, 2H), 2.07 (t, *J* = 7.5 Hz, 2H), 1.69 – 1.41 (m, 2H), 0.84 (t, *J* = 7.4 Hz, 3H). ^{13}C NMR (101 MHz, Chloroform-*d*) δ 173.21, 138.60, 128.59(×2), 127.67(×2), 127.31, 43.38, 38.50, 19.21, 13.79. HRMS (EI): exact mass calculated for $C_{11}H_{15}NO$ $[M]^+$ require m/z = 177.1154, found m/z = 177.1156.

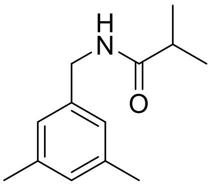

66

***N*-(3,5-dimethylbenzyl)isobutyramide**

White solid, 74% yield. 4.4 F mol^{-1}. ^{1}H NMR (400 MHz, Chloroform-*d*) δ 6.93 – 6.85 (m, 3H), 5.86 (s, 1H), 4.34 (d, *J* = 5.6 Hz, 2H), 2.38 (p, *J* = 6.9 Hz, 1H), 2.29 (d, *J* = 0.7 Hz, 6H), 1.18 (d, *J* = 6.9 Hz, 6H). ^{13}C NMR (101 MHz, Chloroform-*d*) δ 176.82, 138.39, 138.29, 129.06(×2), 125.60(×2), 43.42, 35.64, 21.24(×2), 19.65(×2). HRMS (EI): exact mass calculated for $C_{13}H_{19}NO$ $[M]^+$ require m/z = 205.1467, found m/z = 205.1467.

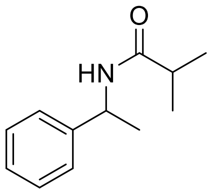

67

***N*-(1-phenylethyl)isobutyramide**

White solid, 72% yield. 4.4 F mol^{-1}.^{1}H NMR (400 MHz, Chloroform-*d*) δ 7.36 – 7.19 (m, 5H), 6.32 (d, *J* = 7.6 Hz, 1H), 5.18 – 5.06 (m, 1H), 2.38 (p, *J* = 6.9 Hz, 1H), 1.46 (d, *J* = 6.9 Hz, 3H), 1.14 (dd, *J* = 11.8, 6.9 Hz, 6H). ^{13}C NMR (101 MHz, Chloroform-*d*) δ 176.24, 143.73, 128.55(×2), 127.09, 126.09(×2),

48.35, 35.42, 21.92, 19.65, 19.58. HRMS (EI): exact mass calculated for $C_{12}H_{17}NO$ $[M]^+$ require m/z = 191.1310, found m/z = 191.1312.

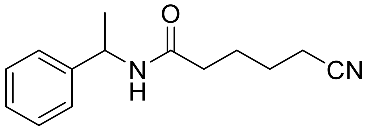

68

5-cyano-*N*-(1-phenylethyl)pentanamide

White solid, 76% yield. 4.4 F mol^{-1}. ^1H NMR (400 MHz, Chloroform-*d*) δ 7.38 – 7.22 (m, 5H), 5.94 (d, *J* = 7.6 Hz, 1H), 5.10 (p, *J* = 7.1 Hz, 1H), 2.33 (t, *J* = 7.0 Hz, 2H), 2.21 (td, *J* = 7.0, 1.5 Hz, 2H), 1.77 (dtd, *J* = 8.9, 7.8, 7.0, 5.7 Hz, 2H), 1.67 (dqd, *J* = 8.9, 6.8, 2.0 Hz, 2H), 1.48 (d, *J* = 6.9 Hz, 3H). ^{13}C NMR (101 MHz, Chloroform-*d*) δ 170.98, 143.18, 128.71(×2), 127.42, 126.14(×2), 119.61, 48.83, 35.45, 24.87, 24.59, 21.81, 16.99. HRMS (EI): exact mass calculated for $C_{14}H_{18}N_2O$ $[M]^+$ require m/z = 230.1419, found m/z = 230.1417.

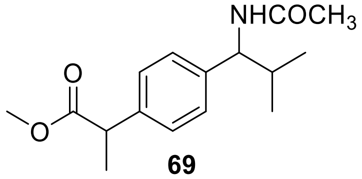

69

methyl 2-(4-(1-acetamido-2-methylpropyl)phenyl)propanoate

White solid, 77% yield. 4.4 F mol^{-1}. ^1H NMR (400 MHz, Chloroform-*d*) δ 7.13 (s, 5H), 4.63 (t, *J* = 8.6 Hz, 1H), 3.63 (q, *J* = 7.1 Hz, 1H), 3.54 (s, 3H), 1.96 – 1.87 (m, 1H), 1.85 (s, 3H), 1.39 (d, *J* = 7.3 Hz, 3H), 0.87 (d, *J* = 6.7 Hz, 3H), 0.71 (d, *J* = 6.8 Hz, 3H). ^{13}C NMR (101 MHz, Chloroform-*d*) δ 175.04, 169.88, 141.01, 138.88, 127.40 (×2), 127.25 (×2), 59.08, 51.87, 44.95, 33.24, 22.98, 19.81, 18.99, 18.48. HRMS (EI): exact mass calculated for $C_{16}H_{23}NO_3$ $[M]^+$ require m/z = 277.1678, found m/z = 277.1680.

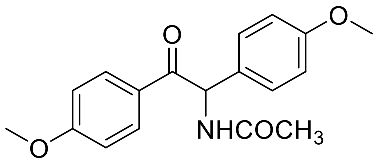

70

***N*-(1,2-bis(4-methoxyphenyl)-2-oxoethyl)acetamide**

White solid, 63% yield. 4.4 F mol^{-1}. ^1H NMR (400 MHz, Chloroform-*d*) δ 7.99 – 7.85 (m, 2H), 7.37 – 7.28 (m, 2H), 6.99 (d, *J* = 7.3 Hz, 1H), 6.90 – 6.77 (m, 4H), 6.47 (d, *J* = 7.3 Hz, 1H), 3.82 (s, 3H), 3.74 (s, 3H), 2.02 (s, 3H). ^{13}C NMR (101 MHz, Chloroform-*d*) δ 194.31, 169.13, 163.96, 159.42, 131.52 (×2), 130.04, 129.38 (×2), 127.16, 114.53 (×2), 113.94 (×2), 57.45, 55.49, 55.23, 23.34. HRMS (EI): exact mass calculated for $C_{18}H_{19}NO_4$ $[M]^+$ require m/z = 313.1314, found m/z = 313.1317.

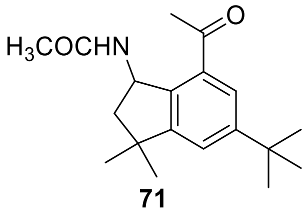

71

***N*-(7-acetyl-5-(tert-butyl)-3,3-dimethyl-2,3-dihydro-1*H*-inden-1-yl)acetamide**

White solid, 78% yield. 4.4 F mol^{-1}. ^1H NMR (400 MHz, Chloroform-*d*) δ 7.59 (d, *J* = 1.8 Hz, 1H), 7.37 (d, *J* = 1.9 Hz, 1H), 6.44 (d, *J* = 7.0 Hz, 1H), 5.57 (td, *J* = 7.4, 4.1 Hz, 1H), 2.51 (s, 3H), 2.32 (dd, *J* = 13.4, 7.7 Hz, 1H), 2.07 – 1.87 (m, 1H), 1.83 (s, 3H), 1.34 (d, *J* = 2.2 Hz, 12H), 1.29 (s, 3H). ^{13}C NMR (101 MHz, Chloroform-*d*) δ 201.02, 169.60, 154.83, 152.45, 137.05, 135.70, 124.78, 123.01, 52.94, 49.12, 42.43, 34.87, 31.36(×3), 30.81, 29.67, 28.84, 22.98. HRMS (EI): exact mass calculated for $C_{19}H_{27}NO_2$ $[M]^+$ require m/z = 301.2042, found m/z = 301.2044.

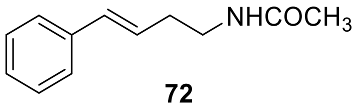

72

N-(4-phenylbut-3-en-1-yl)acetamide

White solid, 32% yield. 4.4 F mol^{-1}. ^1H NMR (600 MHz, Chloroform-d) δ 7.37 – 7.27 (m, 4H), 7.26 – 7.16 (m, 1H), 6.46 (dt, J = 15.8, 1.6 Hz, 1H), 6.15 (dt, J = 15.8, 7.1 Hz, 1H), 5.58 (s, 1H), 3.40 (td, J = 6.7, 5.7 Hz, 2H), 2.43 (qd, J = 6.8, 1.5 Hz, 2H), 1.97 (s, 3H). ^{13}C NMR (151 MHz, Chloroform-d) δ 170.16, 137.10, 132.42, 128.60 (×2), 127.39, 126.83, 126.09 (×2), 38.99, 33.04, 23.35. HRMS (EI): exact mass calculated for $C_{12}H_{15}NO$ $[M]^+$ require m/z = 189.1154, found m/z = 189.1152.

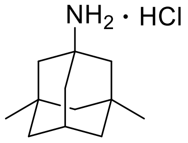

1b

3,5-dimethyladamantan-1-amine

White solid, 85%.^1H NMR (400 MHz, Deuterium Oxide) δ 2.14 (dt, *J* = 6.9, 3.4 Hz, 1H), 1.64 – 1.59 (m, 2H), 1.41 (q, *J* = 11.6 Hz, 4H), 1.30 – 1.25 (m, 4H), 1.11 (q, *J* = 12.8 Hz, 2H), 0.79 (s, 6H). ^{13}C NMR (101 MHz, Deuterium Oxide) δ 53.69, 48.95, 45.76 (×2), 41.08 (×2), 38.51, 31.85 (×2), 29.43 (×2), 28.94. HRMS (EI): exact mass calculated for $C_{12}H_{22}ClN$ $[M]^+$ require m/z = 215.1441, found m/z = 215.1443.

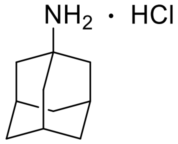

3b

adamantan-1-amine

White solid, 82%. ^1H NMR (400 MHz, Deuterium Oxide) δ 2.11 – 2.03 (m, 3H), 1.78 (d, *J* = 3.1 Hz, 6H), 1.69 – 1.60 (m, 3H), 1.61 – 1.52 (m, 3H). ^{13}C NMR (101 MHz, Deuterium Oxide) δ 52.31, 39.93 (×3), 34.81 (×3), 28.72 (×3). HRMS (EI): exact mass calculated for $C_{10}H_{18}ClN$ $[M]^+$ require m/z = 187.1128, found m/z = 187.1130.

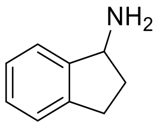

25b

2,3-dihydro-1*H*-inden-1-amine

Colorless oil, 54%. ^1H NMR (400 MHz, Chloroform-*d*) δ 7.36 – 7.28 (m, 1H), 7.25 – 7.14 (m, 3H), 4.33 (t, *J* = 7.5 Hz, 1H), 2.94 (ddd, *J* = 15.8, 8.6, 3.3 Hz, 1H), 2.78 (dt, *J* = 16.1, 8.3 Hz, 1H), 2.48 (dtd, *J* = 12.6, 7.5, 3.3 Hz, 1H), 1.67 (ddd, *J* = 16.7, 8.2, 4.1 Hz, 3H). ^{13}C NMR (101 MHz, Chloroform-*d*) δ 147.47, 143.11, 127.22 (×2), 126.52 (×2), 124.70 (×2), 123.35 (×2), 57.28, 37.37, 30.14. HRMS (EI): exact mass calculated for $C_9H_{11}N$ $[M]^+$ require m/z = 133.0891, found m/z = 133.0893.

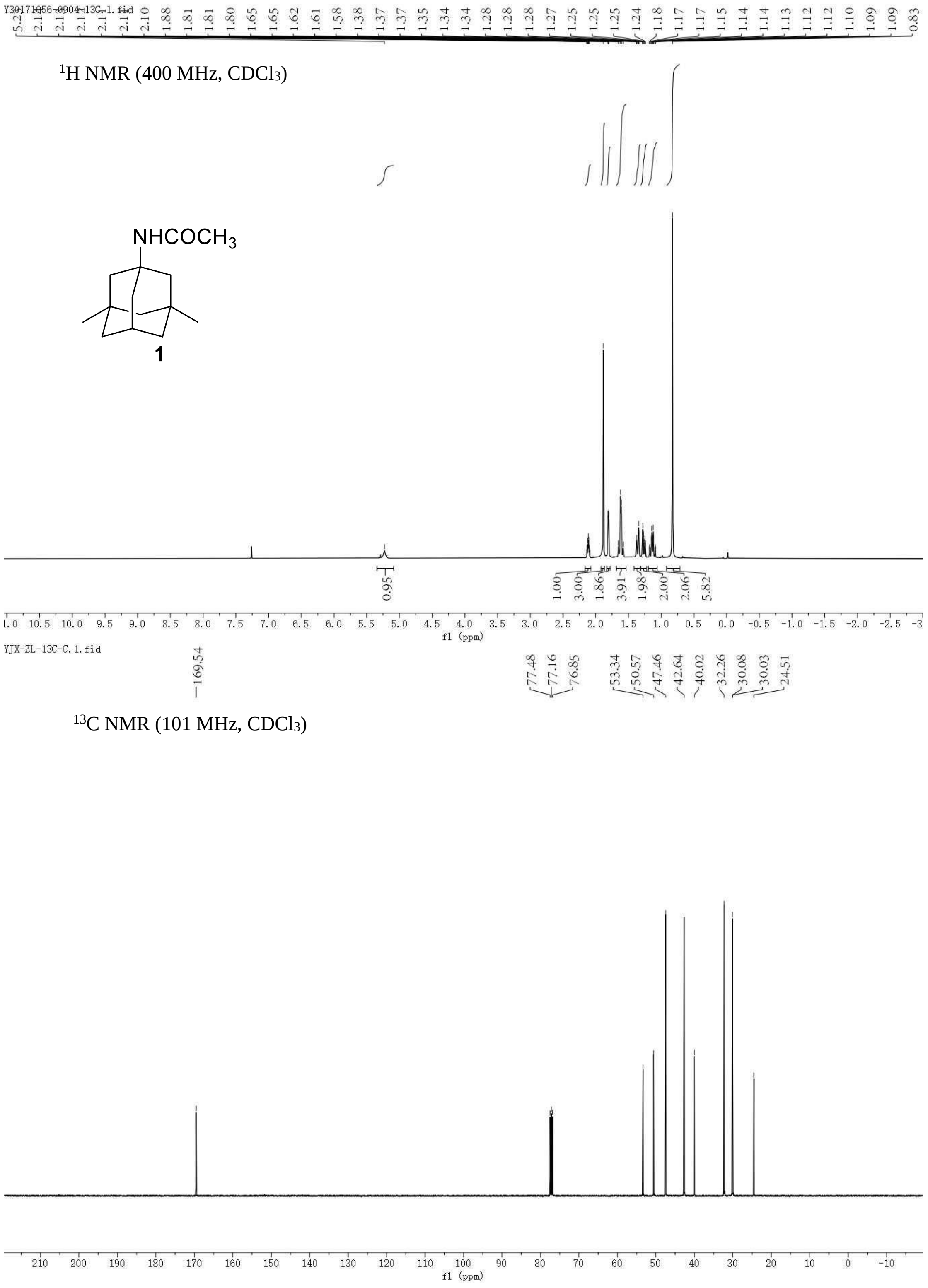

Supplementary Figure 7. ^{1}H NMR and ^{13}C NMR spectra of compound 1.

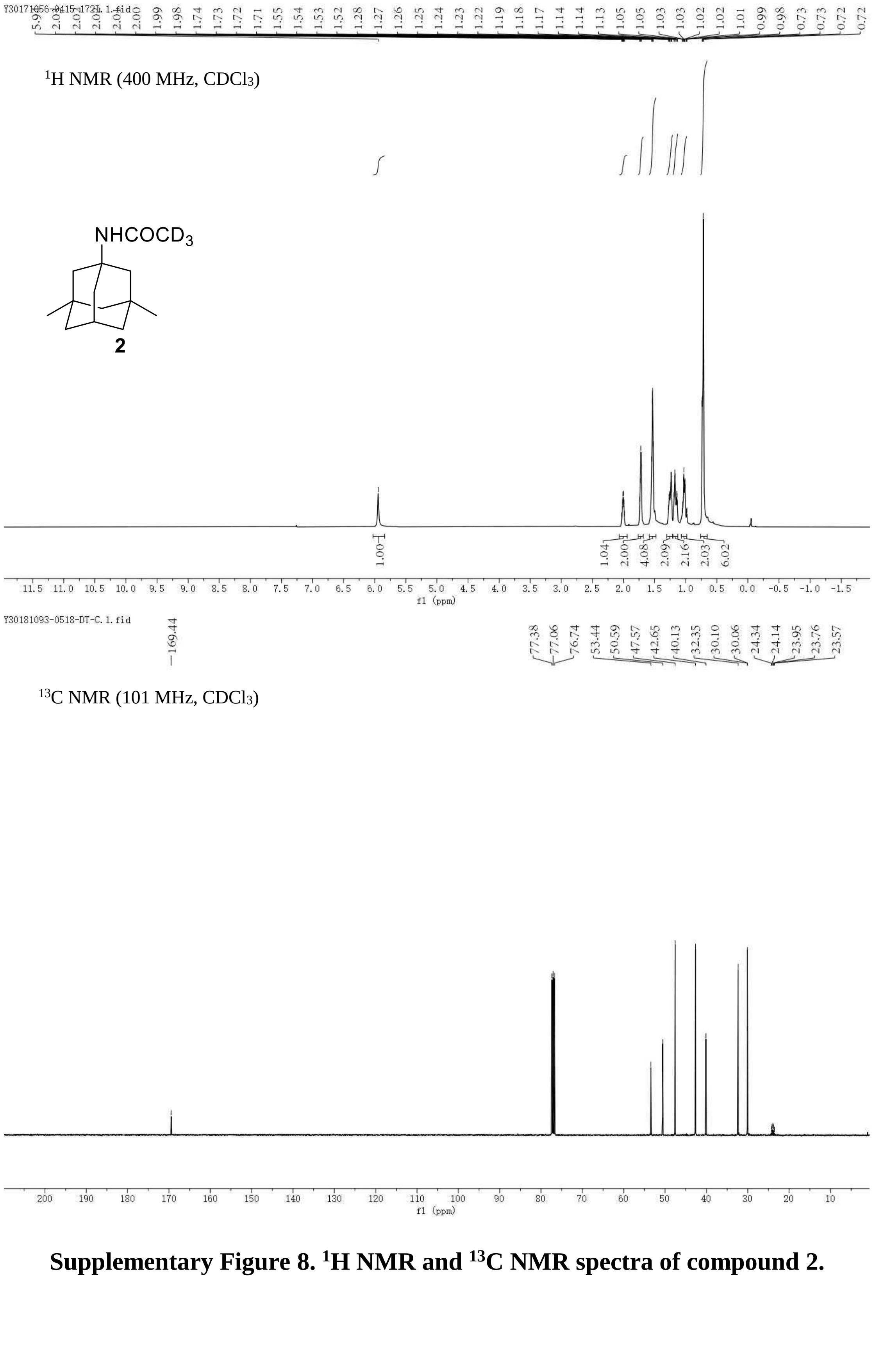

Supplementary Figure 8. ^{1}H NMR and ^{13}C NMR spectra of compound 2.

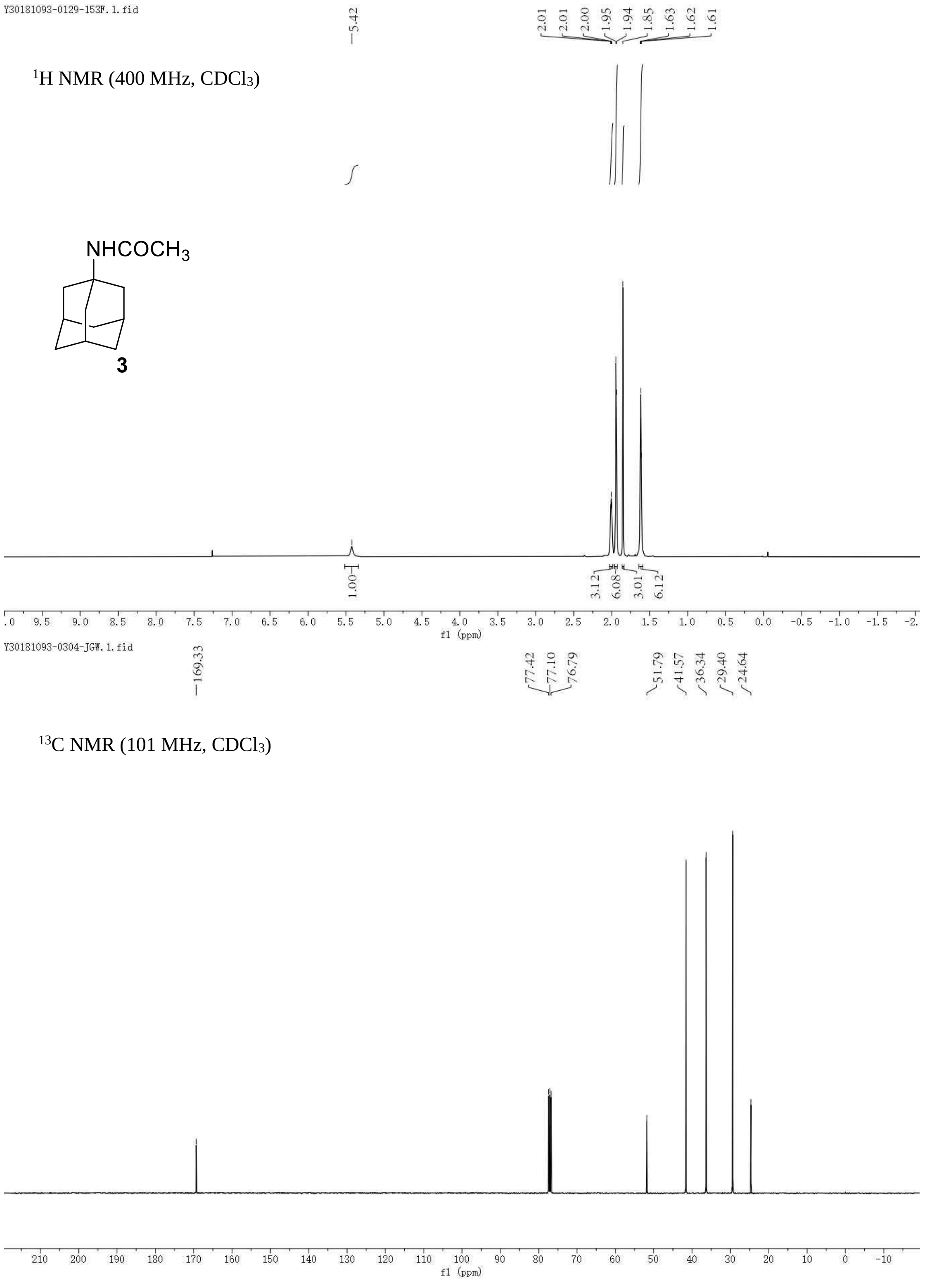

Supplementary Figure 9. ^{1}H NMR and ^{13}C NMR spectra of compound 3.

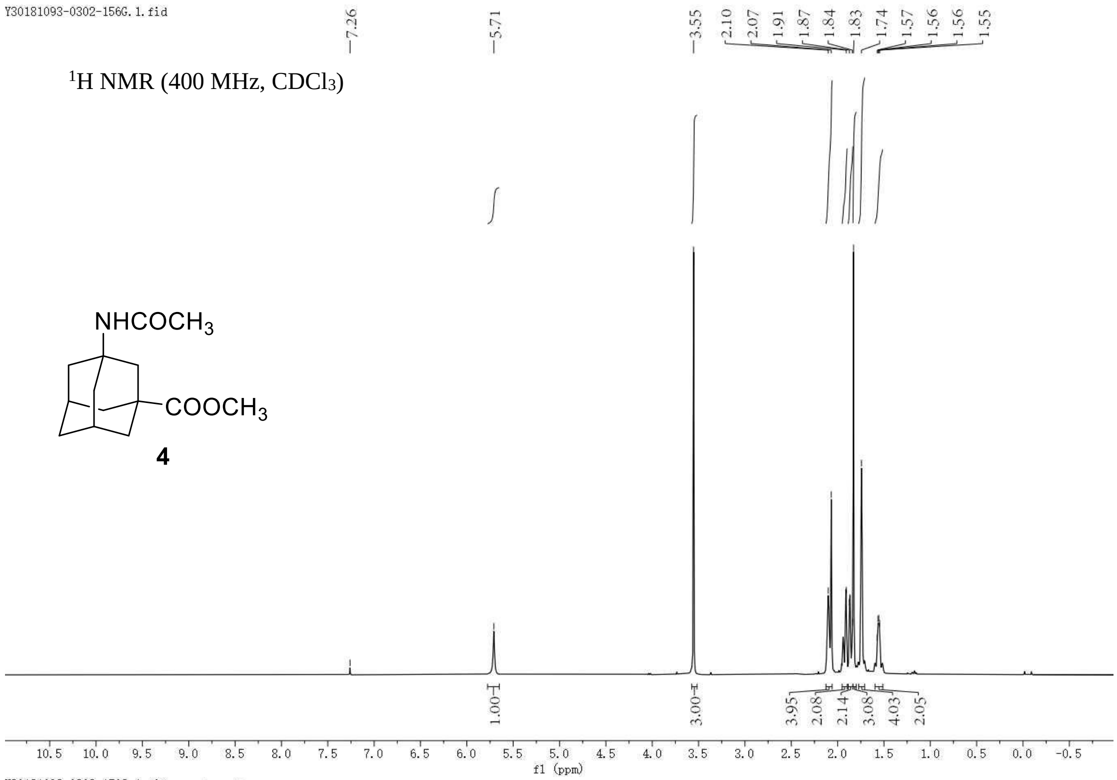

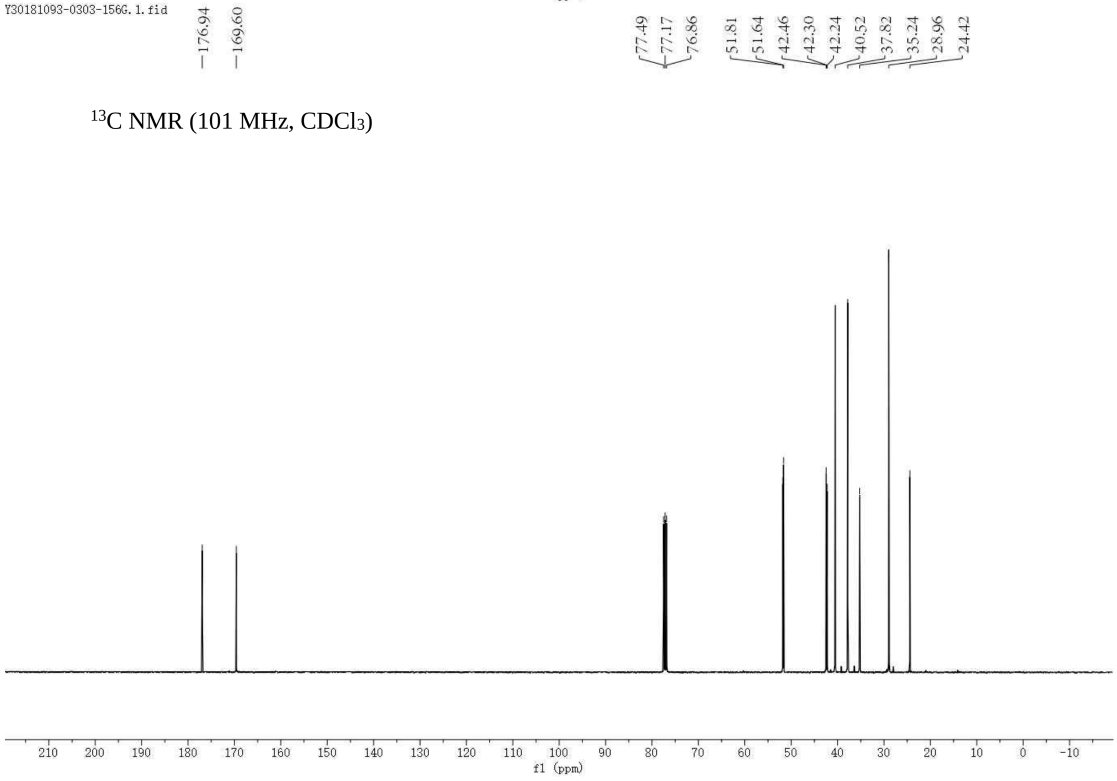

Supplementary Figure 10. ^{1}H NMR and ^{13}C NMR spectra of compound 4.

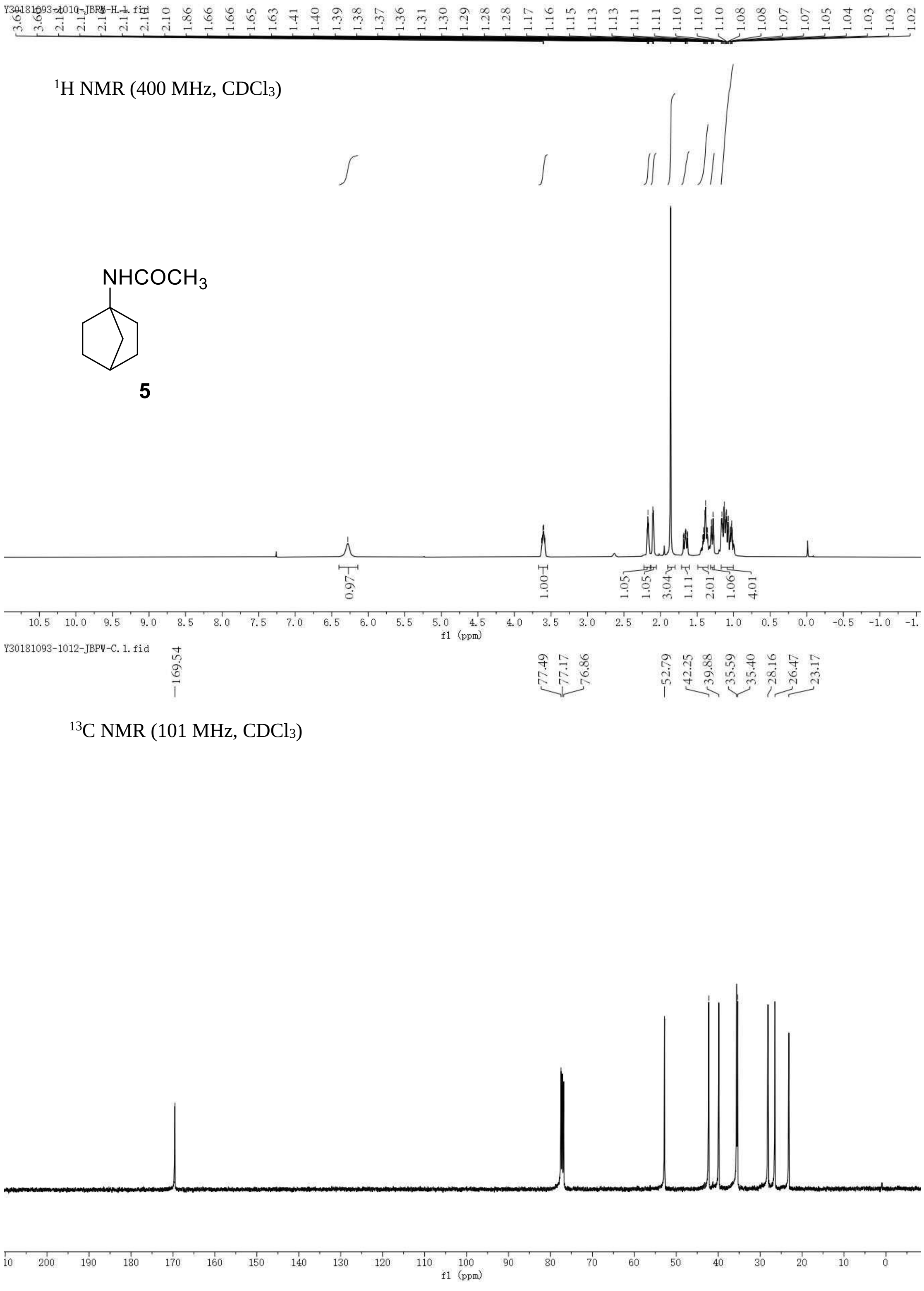

Supplementary Figure 11. ^{1}H NMR and ^{13}C NMR spectra of compound 5.

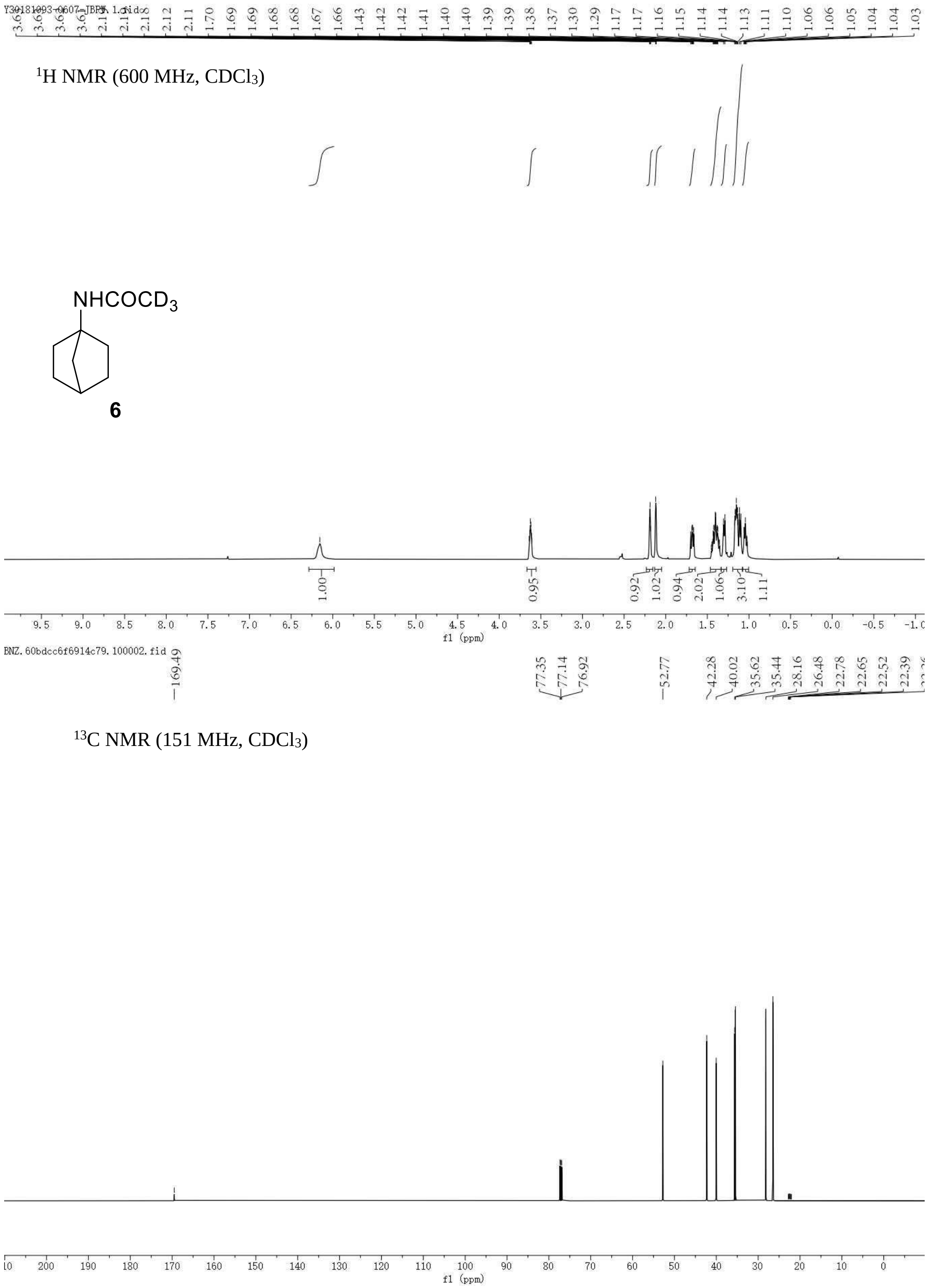

Supplementary Figure 12. ^{1}H NMR and ^{13}C NMR spectra of compound 6.

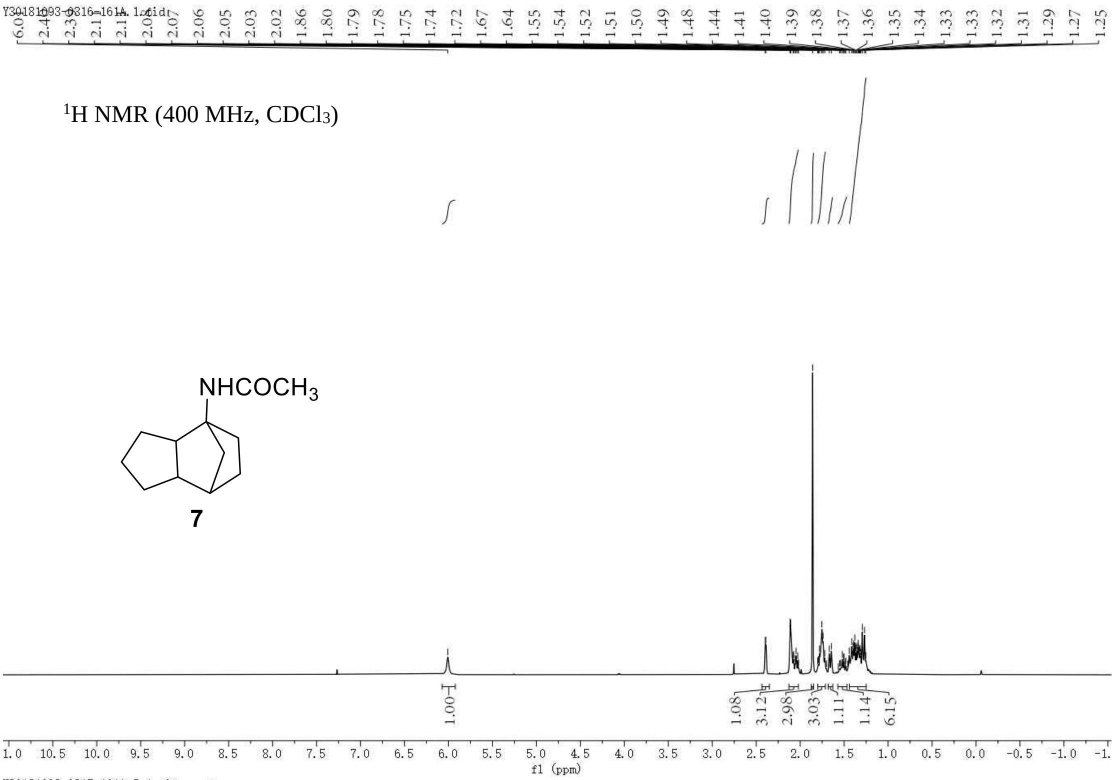

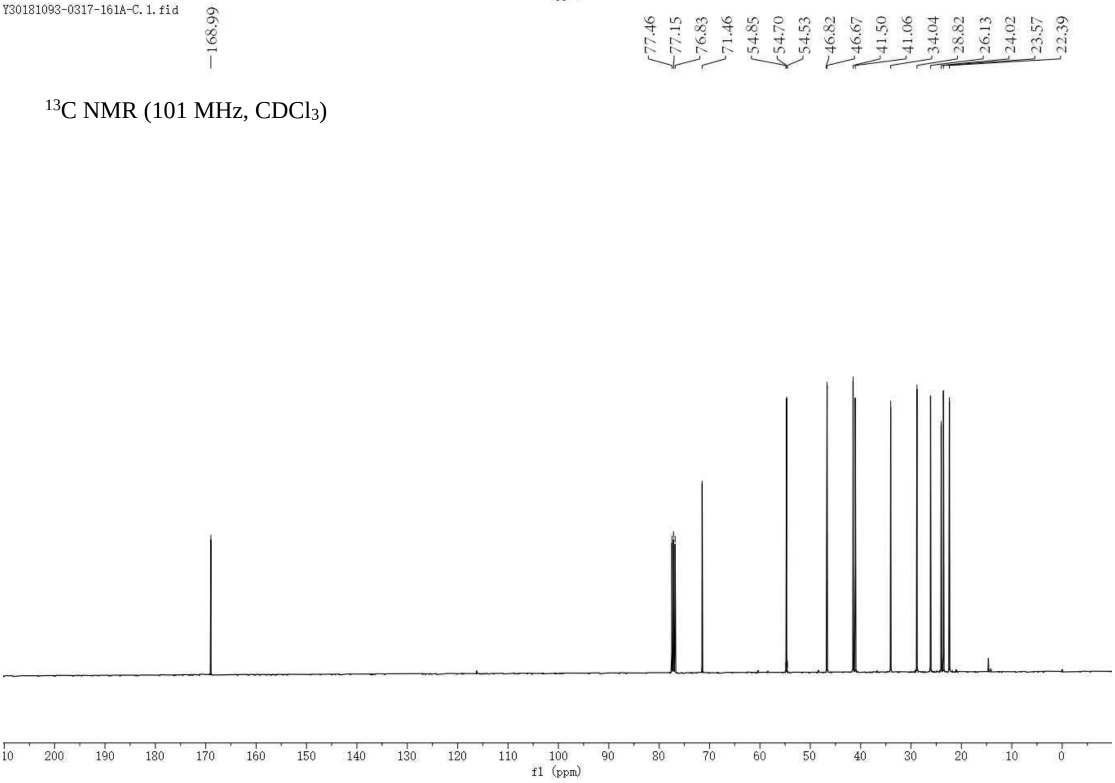

Supplementary Figure 13. ^{1}H NMR and ^{13}C NMR spectra of compound 7.

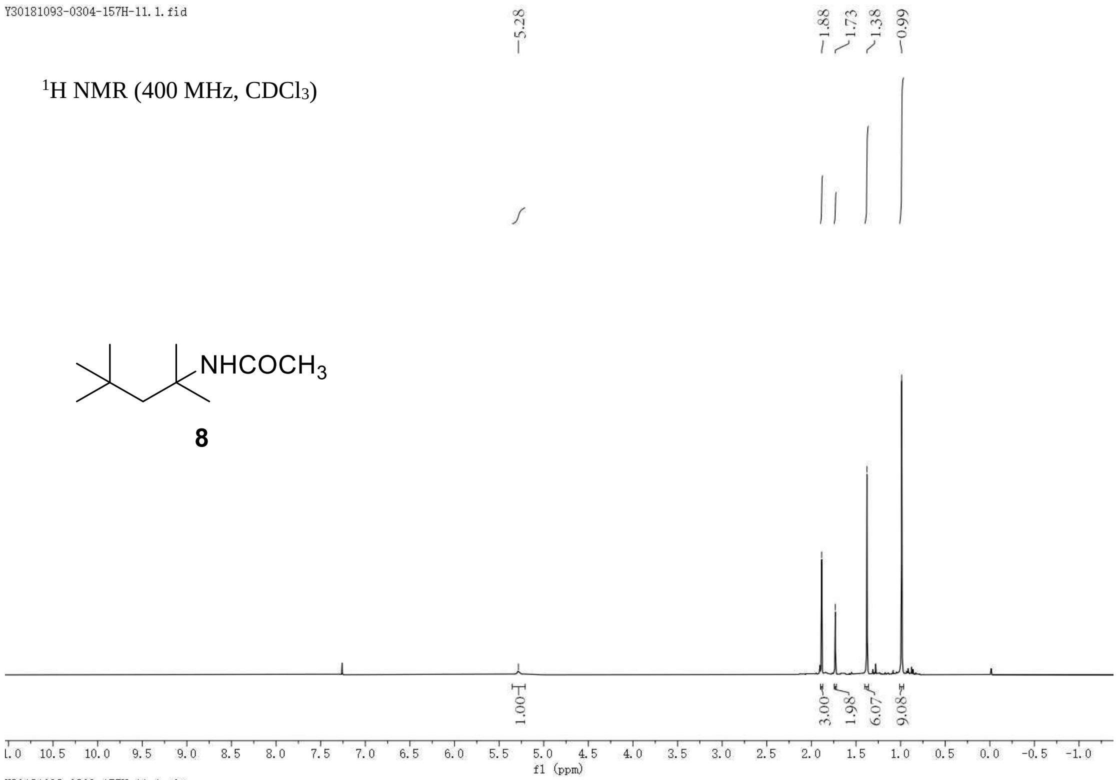

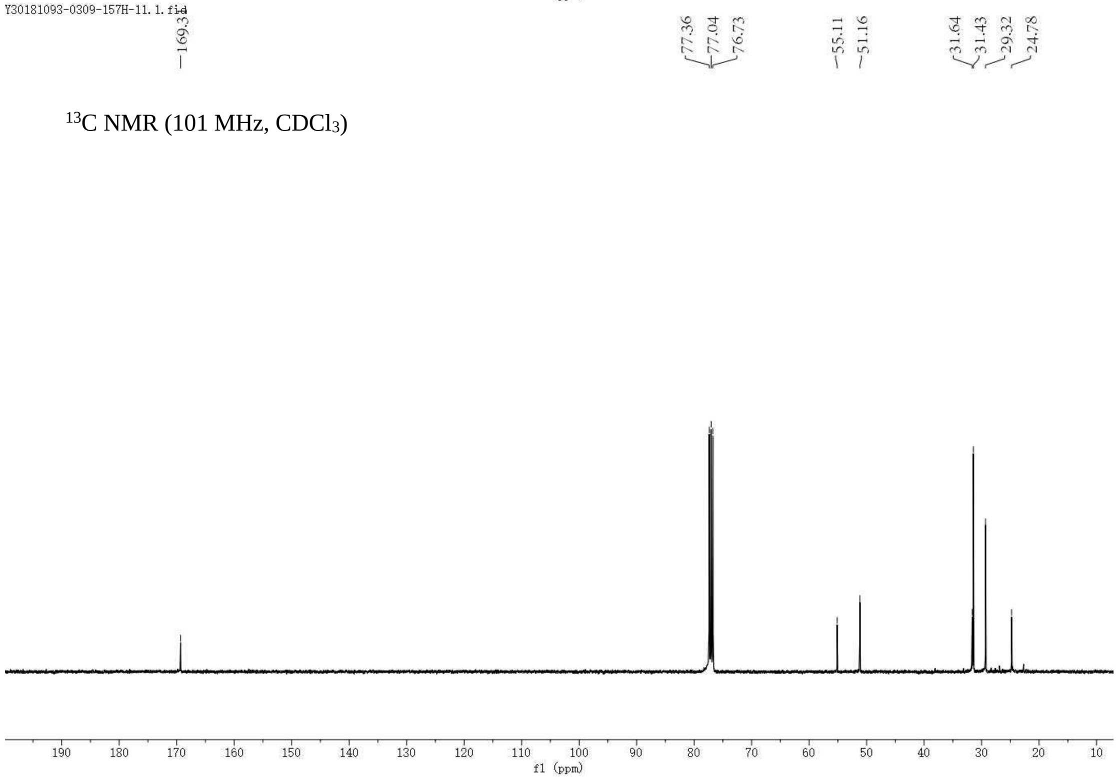

Supplementary Figure 14. ^{1}H NMR and ^{13}C NMR spectra of compound 8.

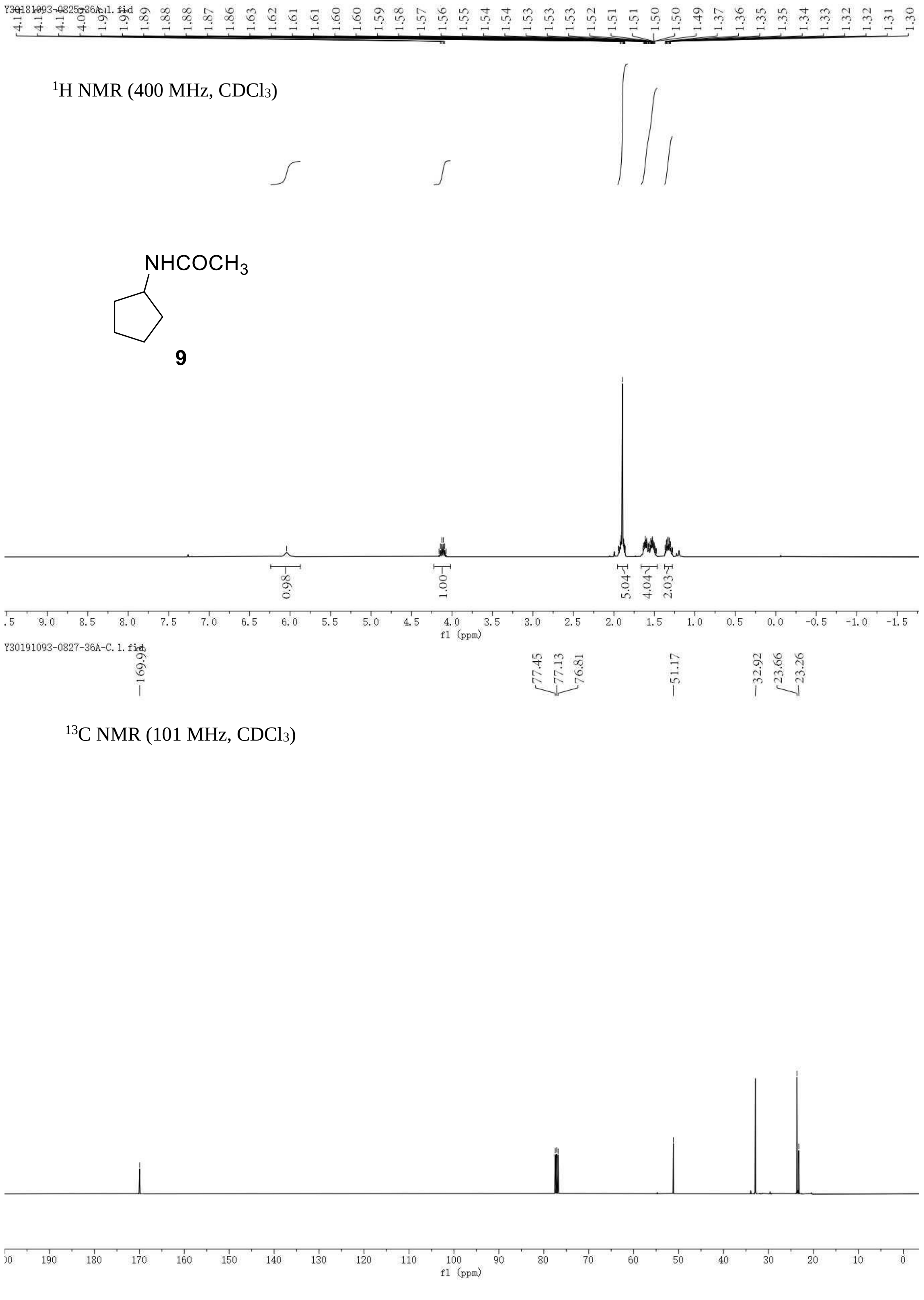

Supplementary Figure 15. ^{1}**H NMR and** ^{13}**C NMR spectra of compound 9.**

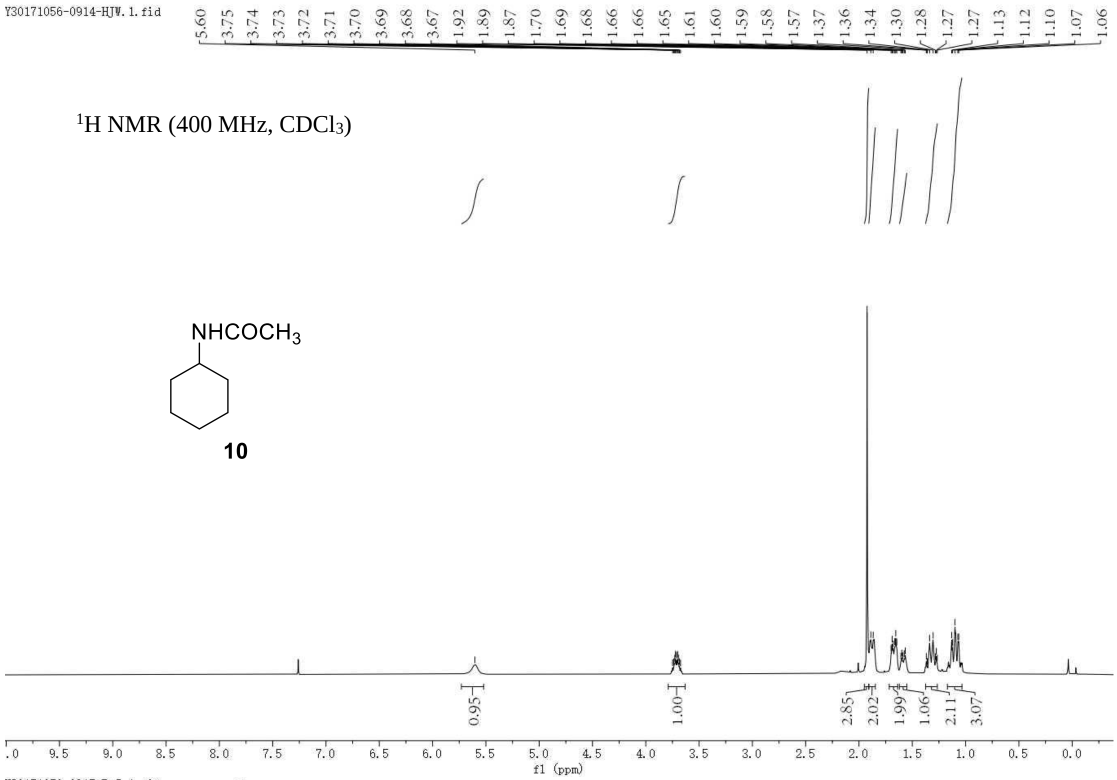

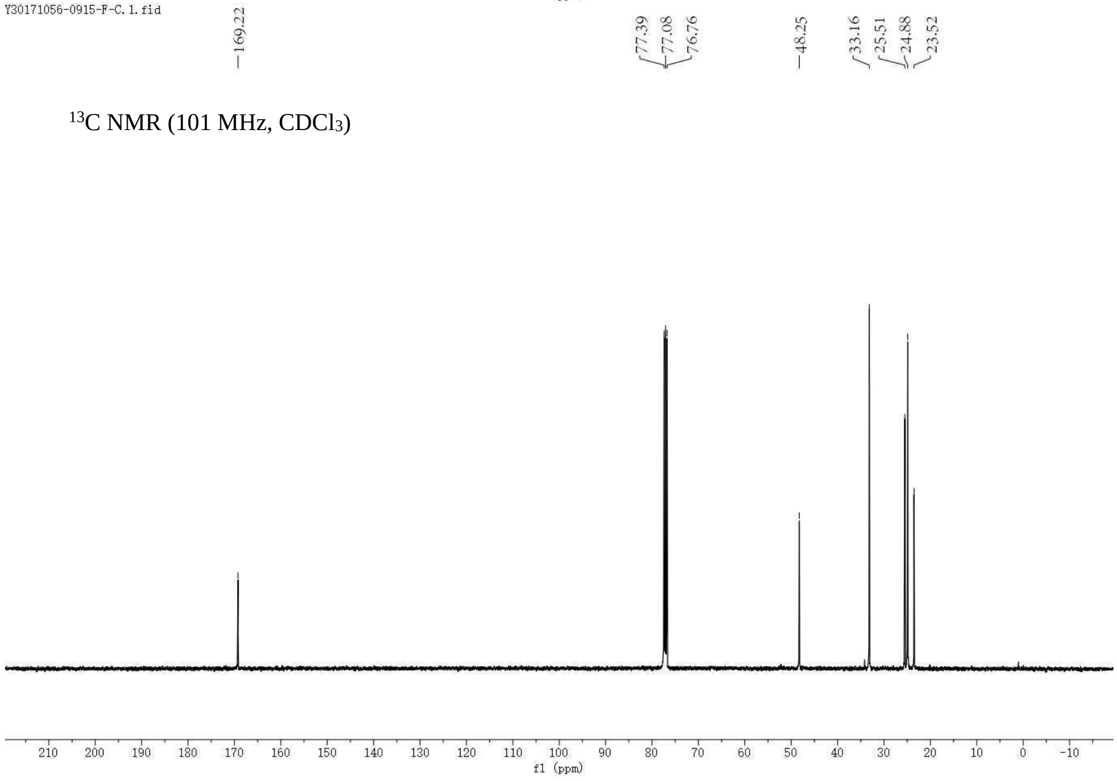

Supplementary Figure 16. ^{1}H NMR and ^{13}C NMR spectra of compound 10.

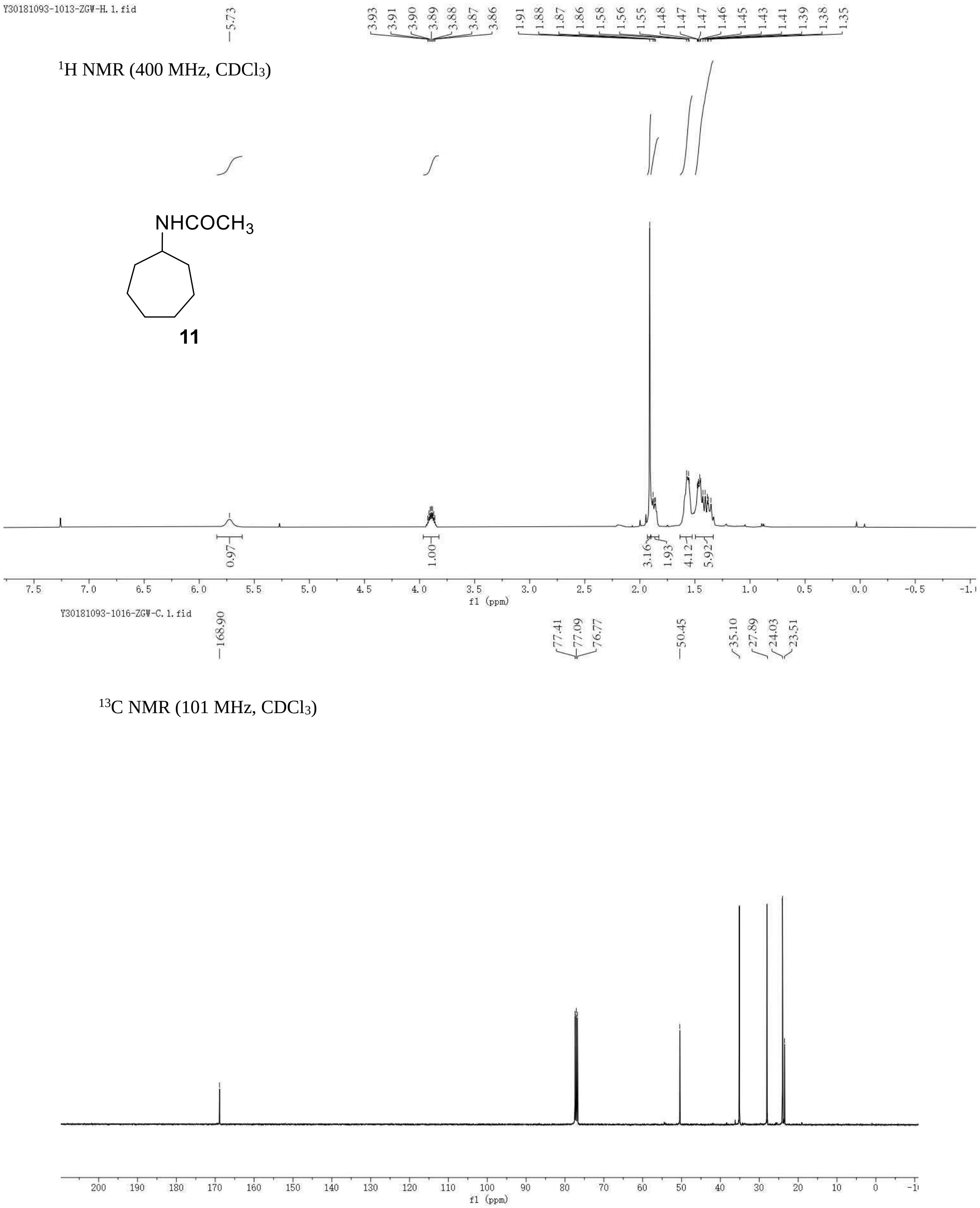

Supplementary Figure 17. ^{1}H NMR and ^{13}C NMR spectra of compound 11.

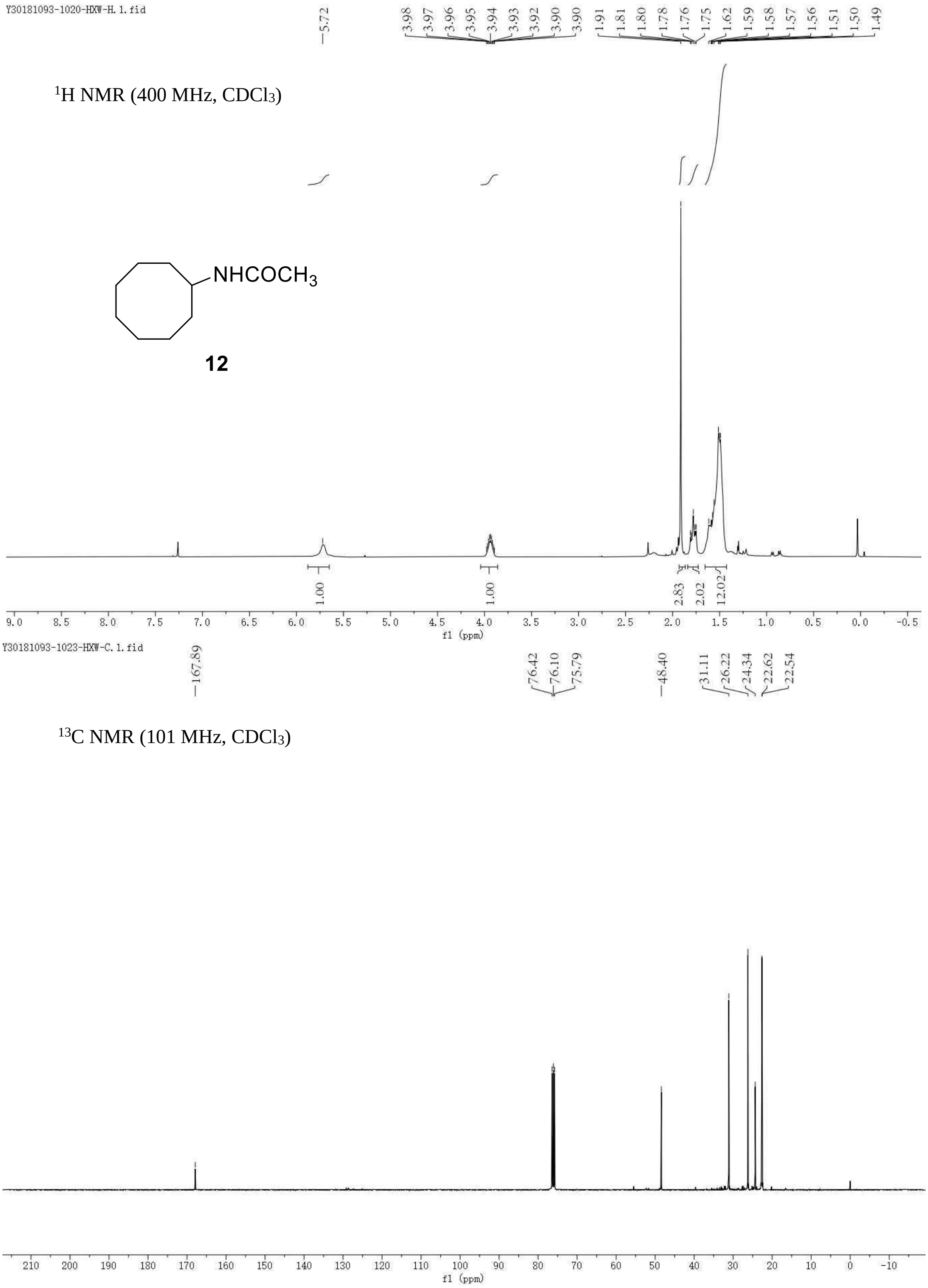

Supplementary Figure 18. ^{1}H NMR and ^{13}C NMR spectra of compound 12.

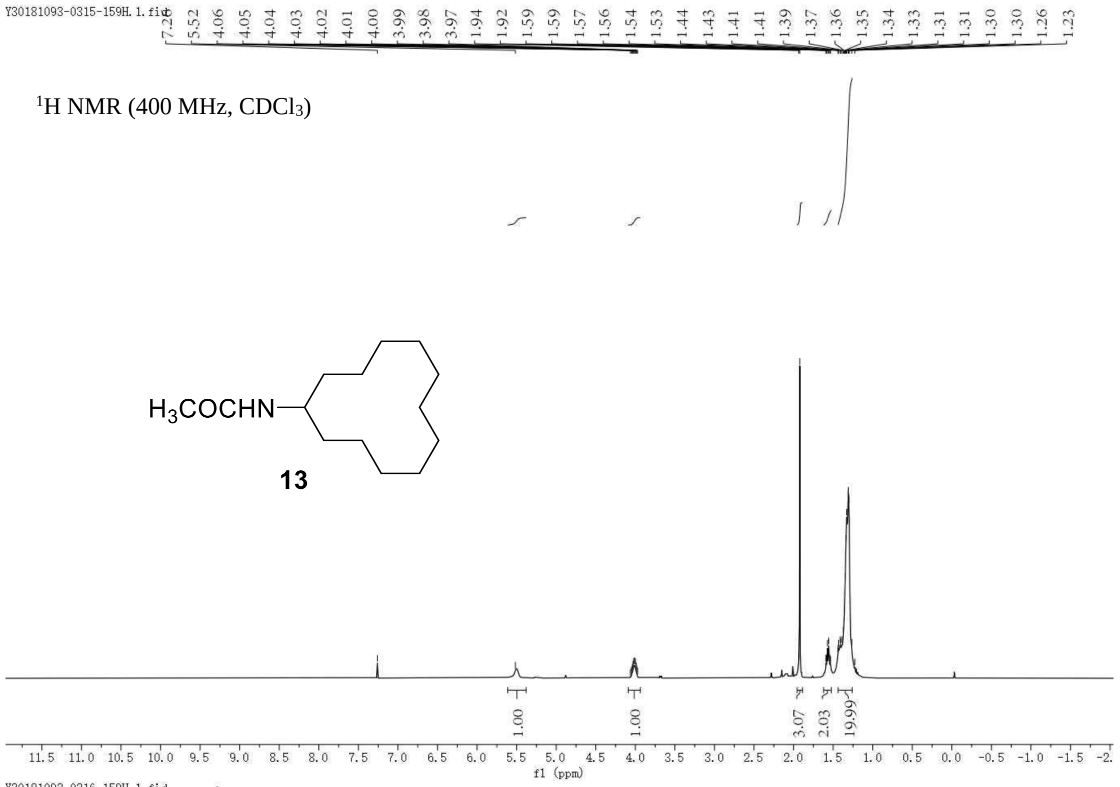

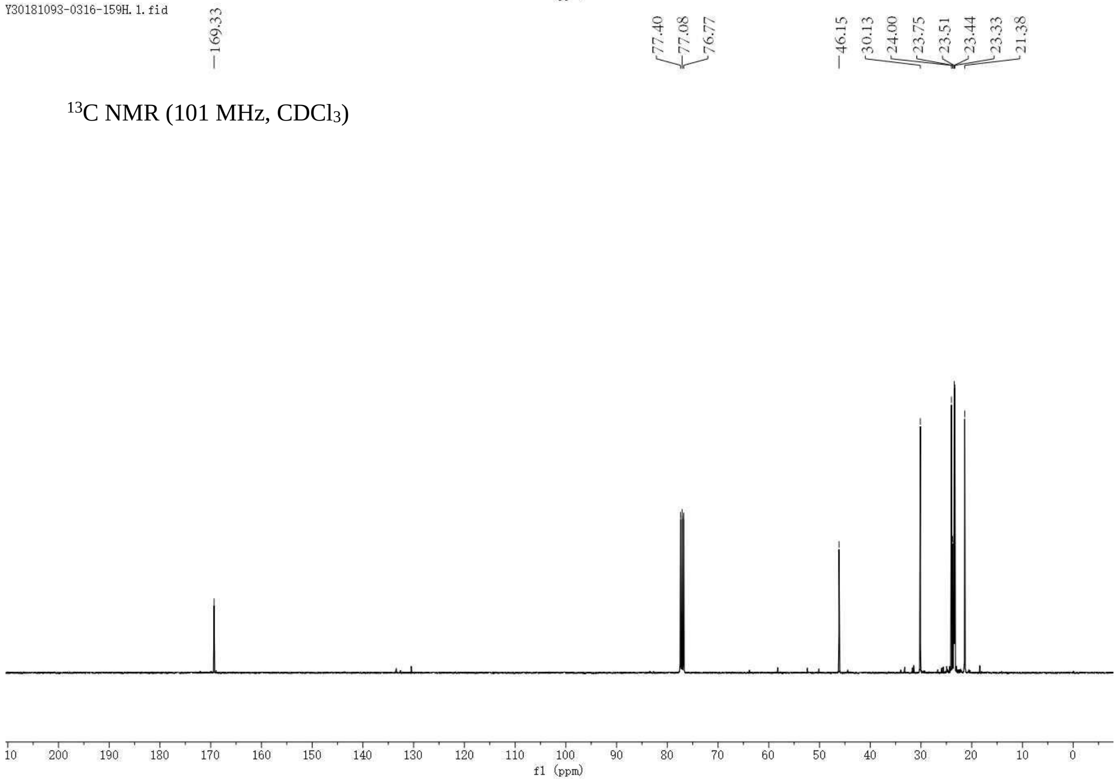

Supplementary Figure 19. ^{1}H NMR and ^{13}C NMR spectra of compound 13.

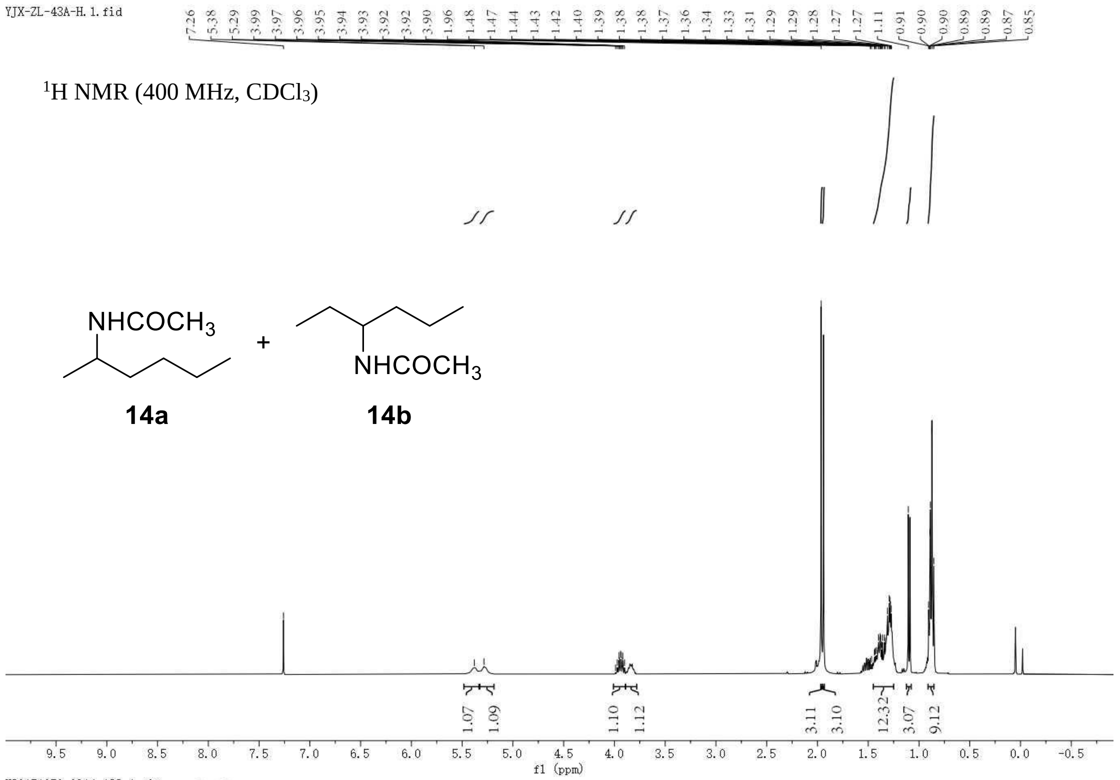

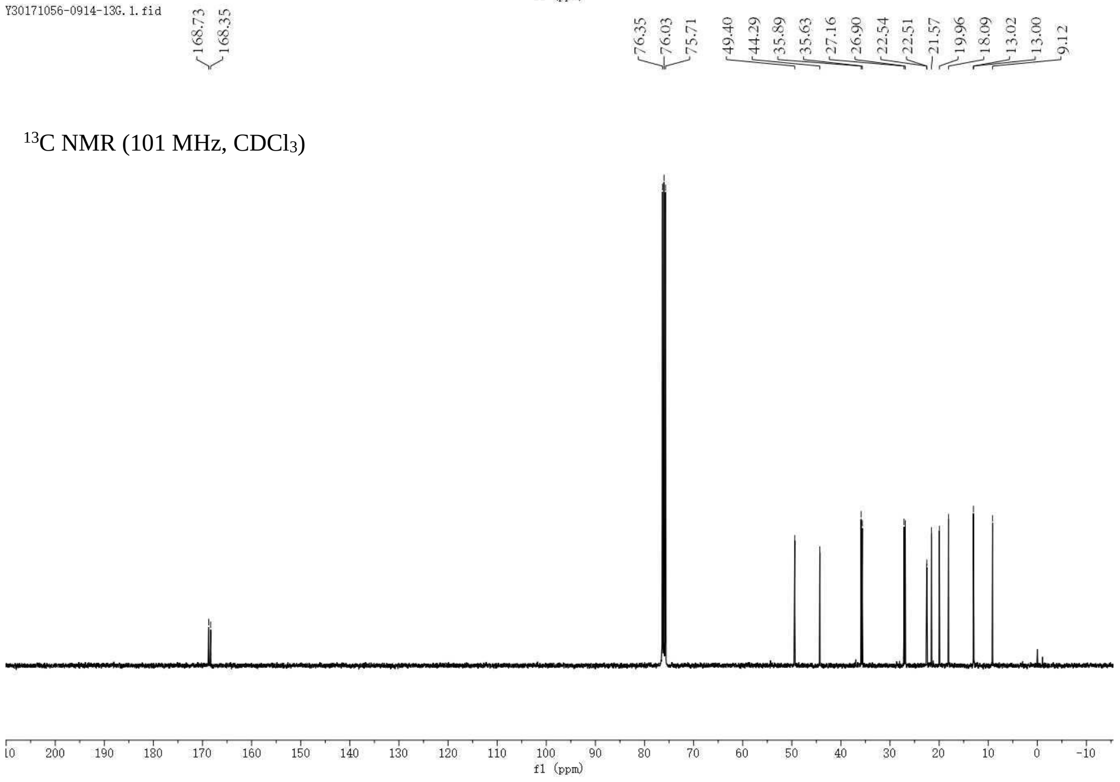

Supplementary Figure 20. ^{1}H NMR and ^{13}C NMR spectra of compound 14a and 14 b.

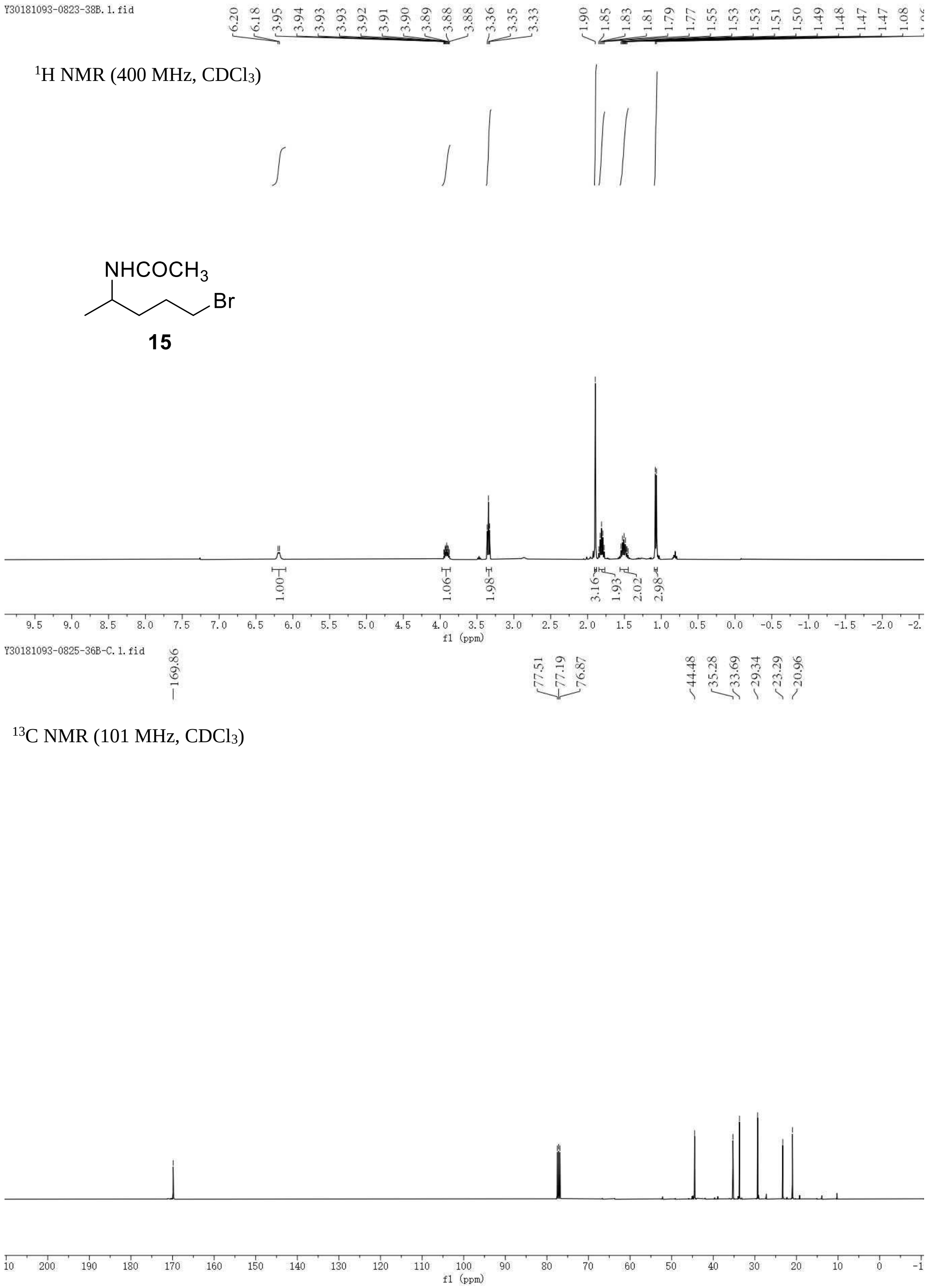

Supplementary Figure 21. ^{1}H NMR and ^{13}C NMR spectra of compound 15.

Y30181093-0303-157A.1.fid

^{1}H NMR (400 MHz, $CDCl_3$)

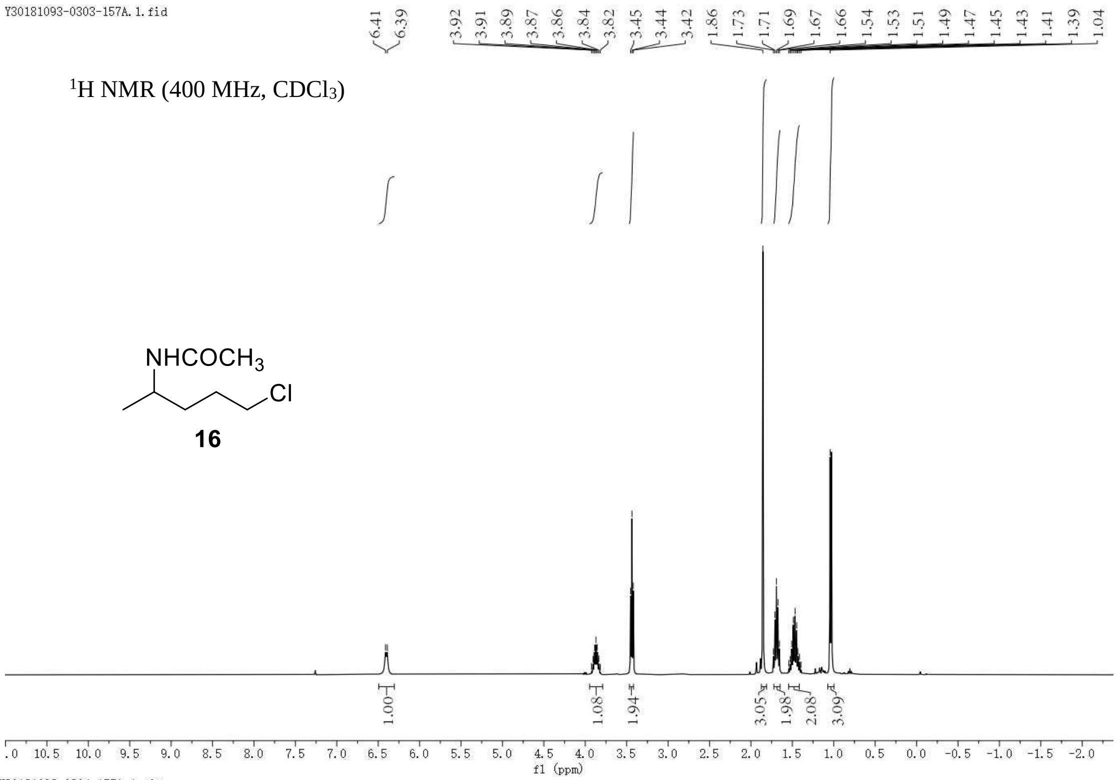

Y30181093-0304-157A.1.fid

^{13}C NMR (101 MHz, $CDCl_3$)

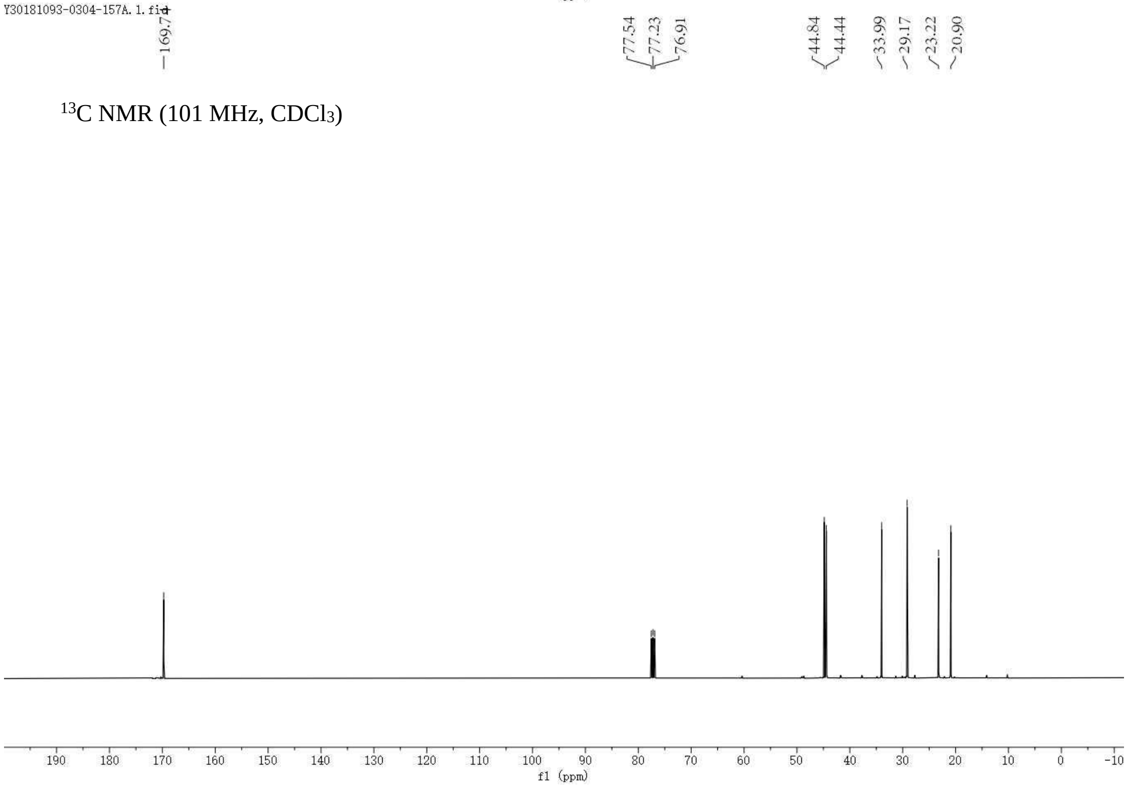

Supplementary Figure 22. ^{1}H NMR and ^{13}C NMR spectra of compound 16.

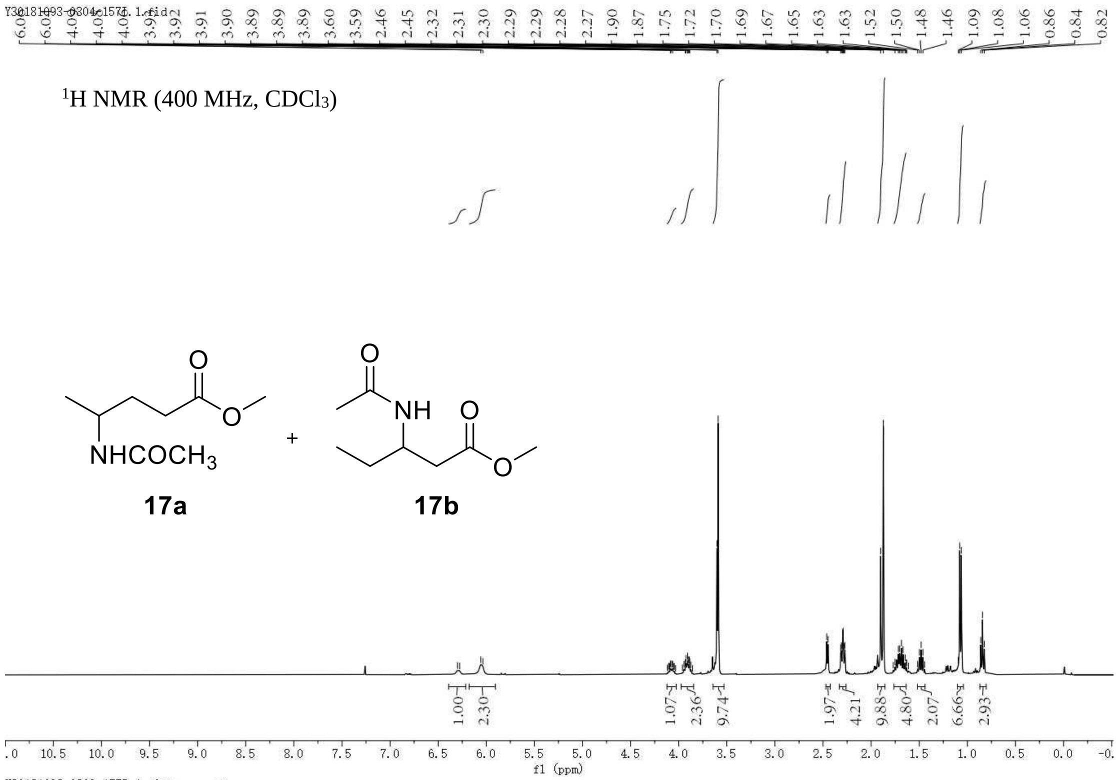

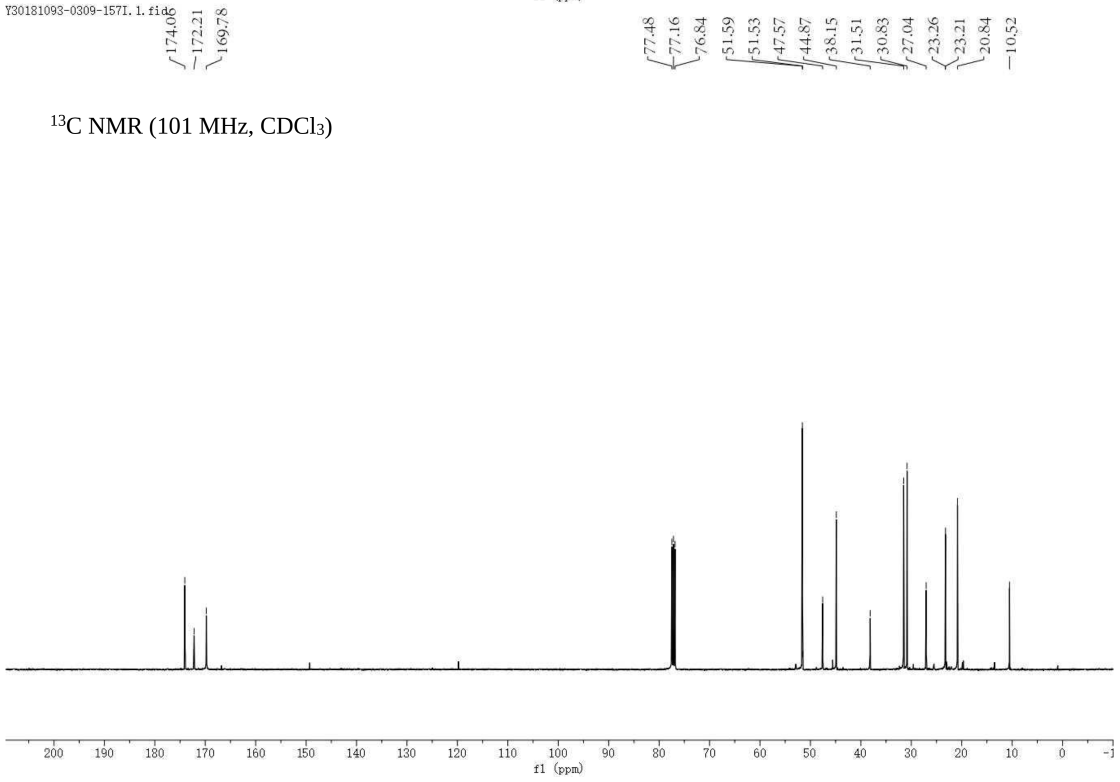

Supplementary Figure 23. ^{1}H NMR and ^{13}C NMR spectra of compound 17a and 17b.

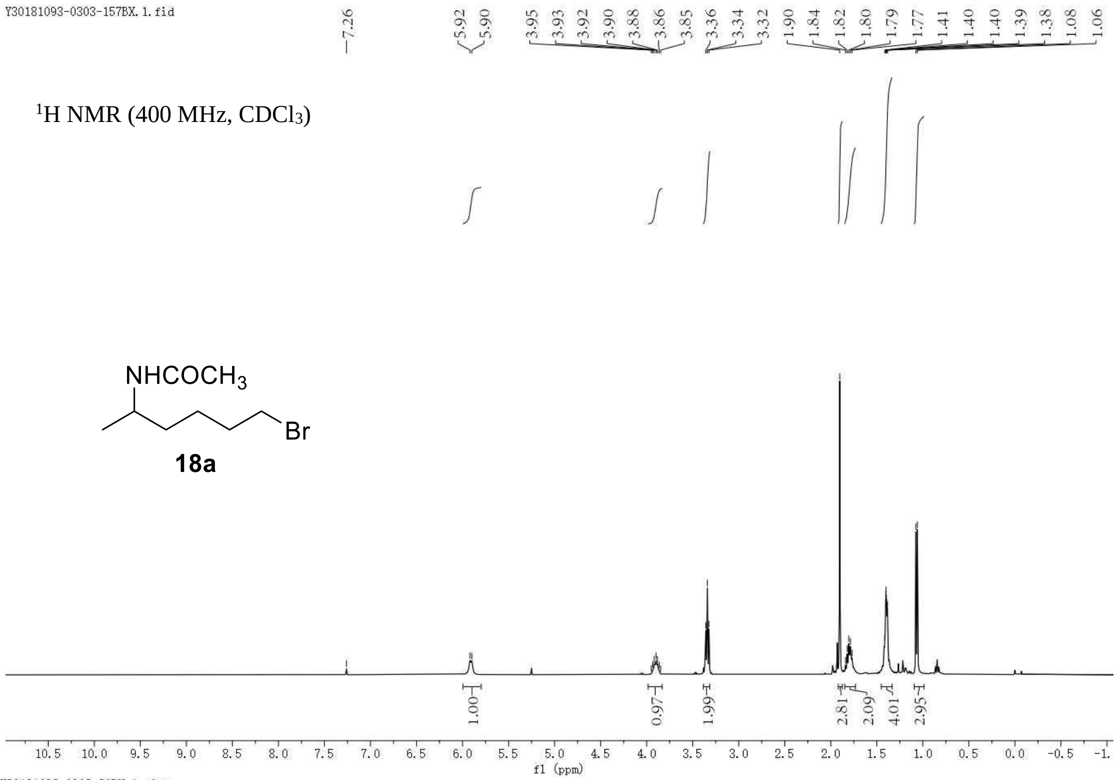

18a

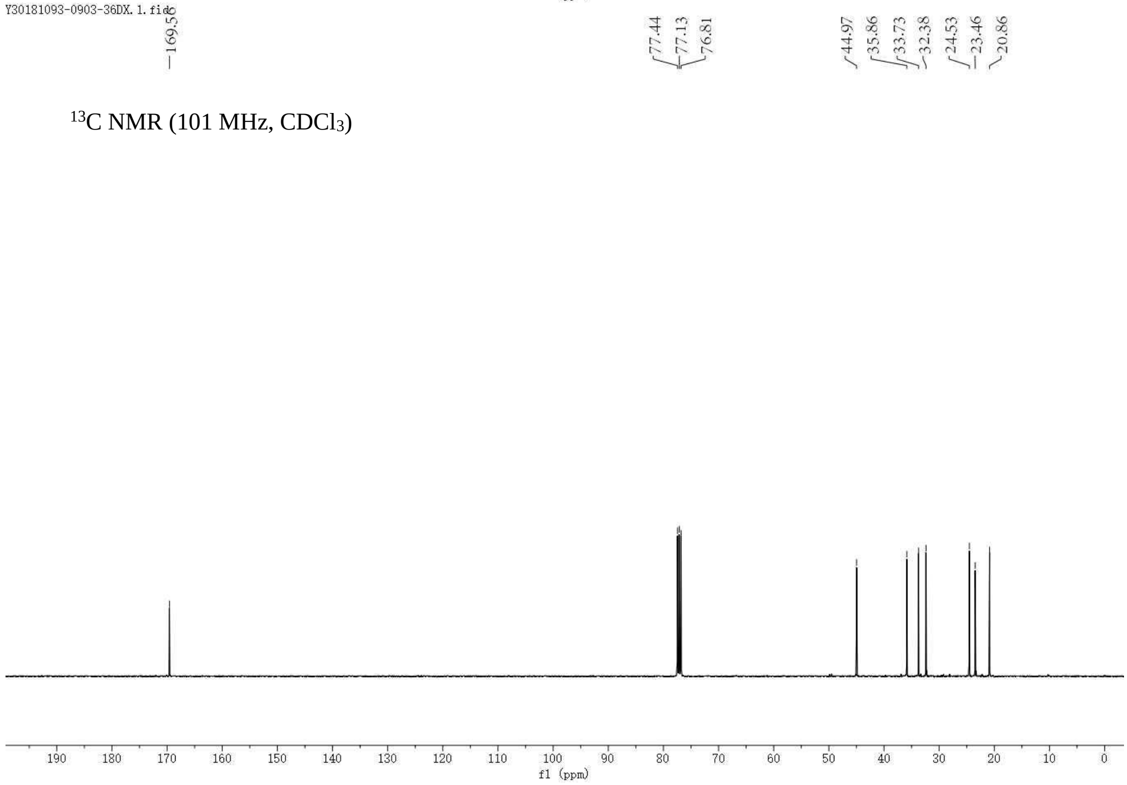

Supplementary Figure 24. ^{1}H NMR and ^{13}C NMR spectra of compound 18a.

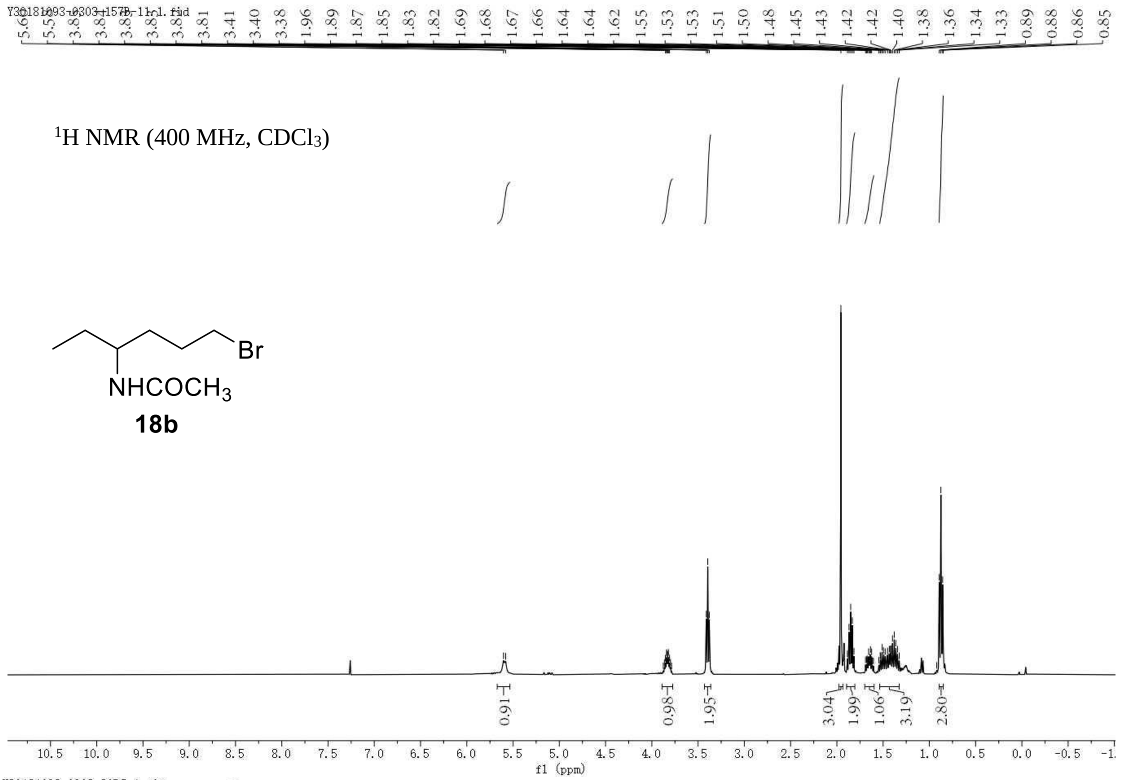

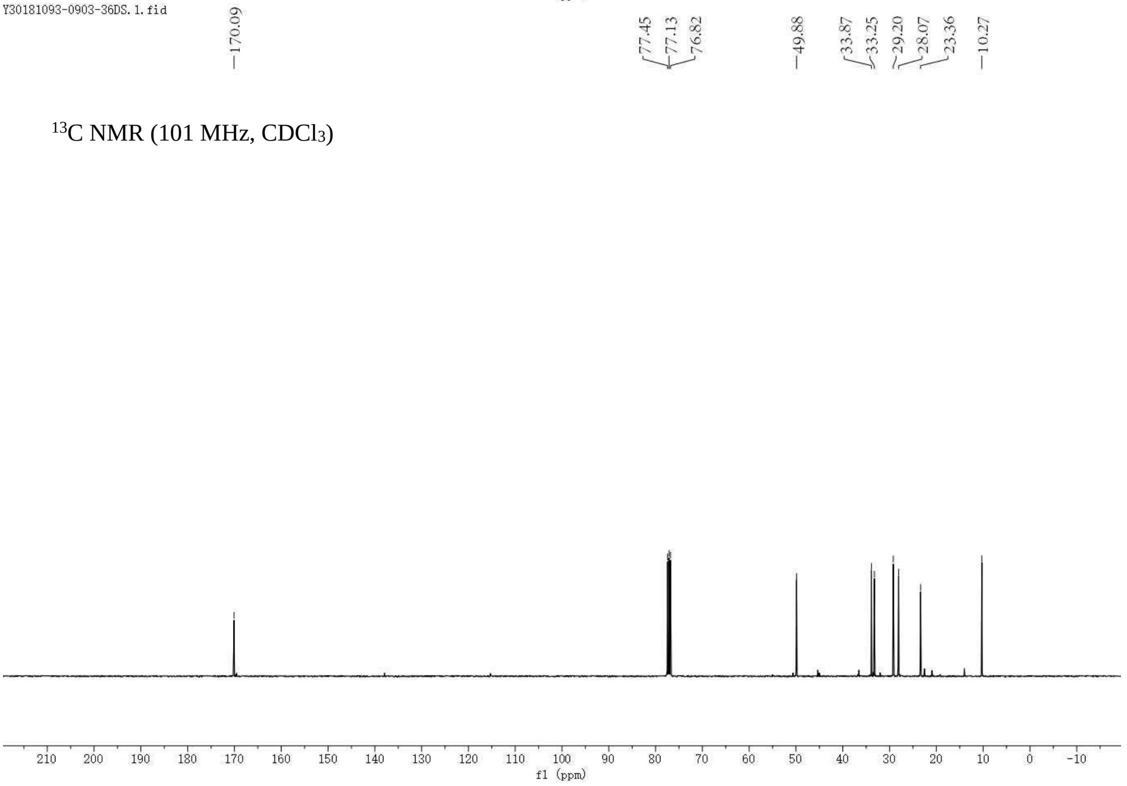

Supplementary Figure 25. ^{1}H NMR and ^{13}C NMR spectra of compound 18b.

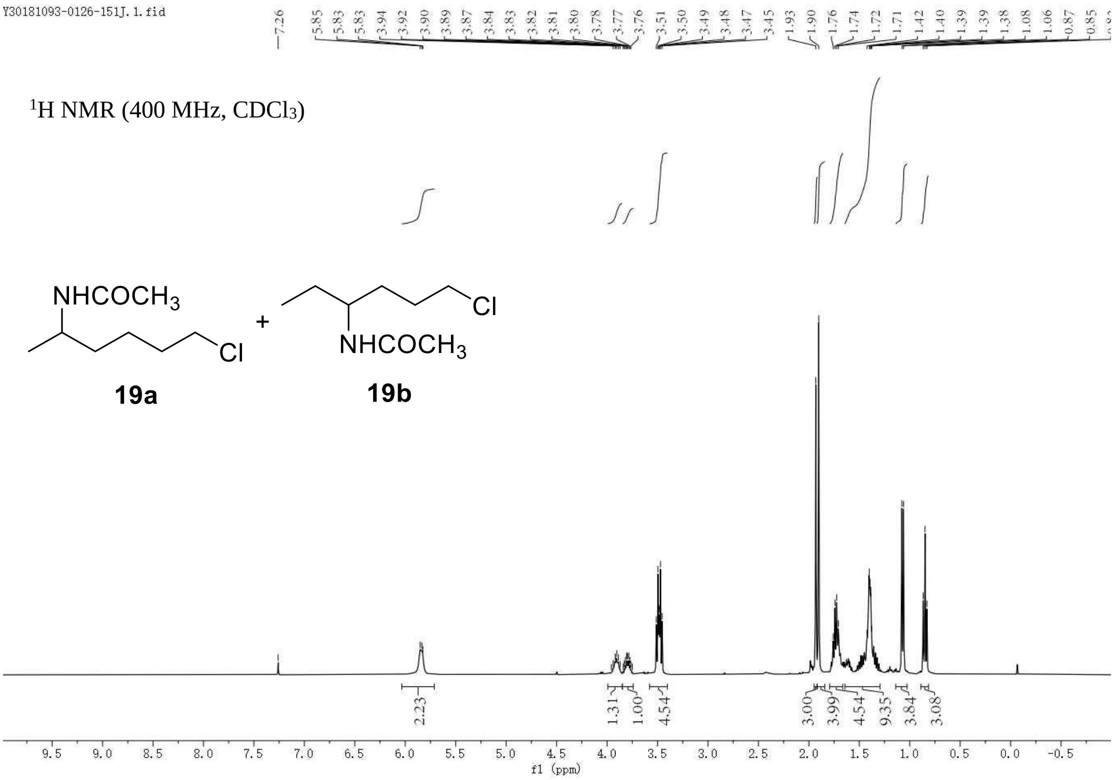
Y30181093-0126-151J.1.fid
1H NMR (400 MHz, CDCl3)
NHCOCH3
Cl
+
19a
19b
f1 (ppm)

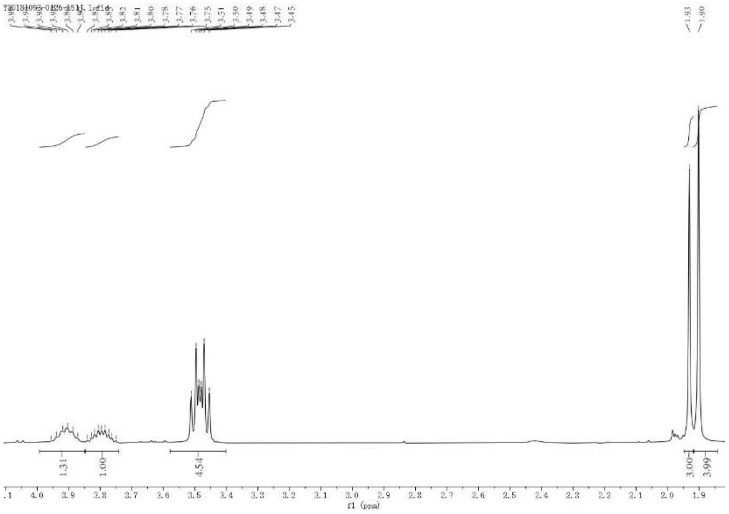
Y30181093-0126-151J.1.fid
f1 (ppm)

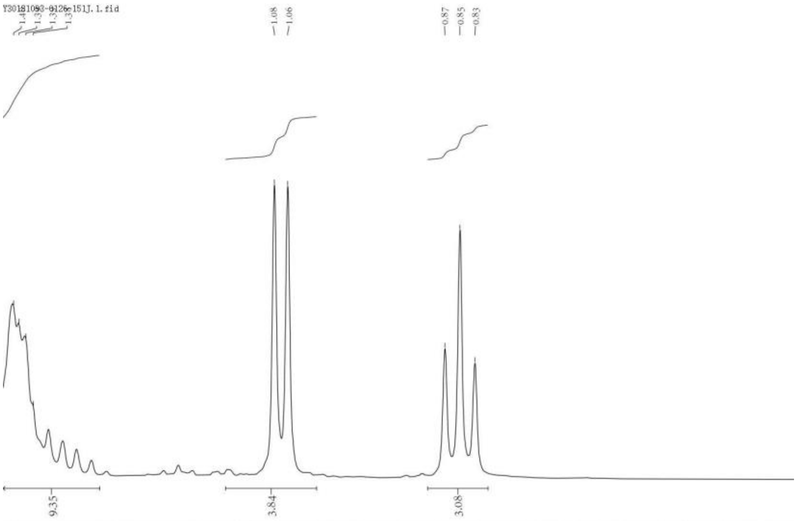
Y30181093-0126-151J.1.fid
f1 (ppm)

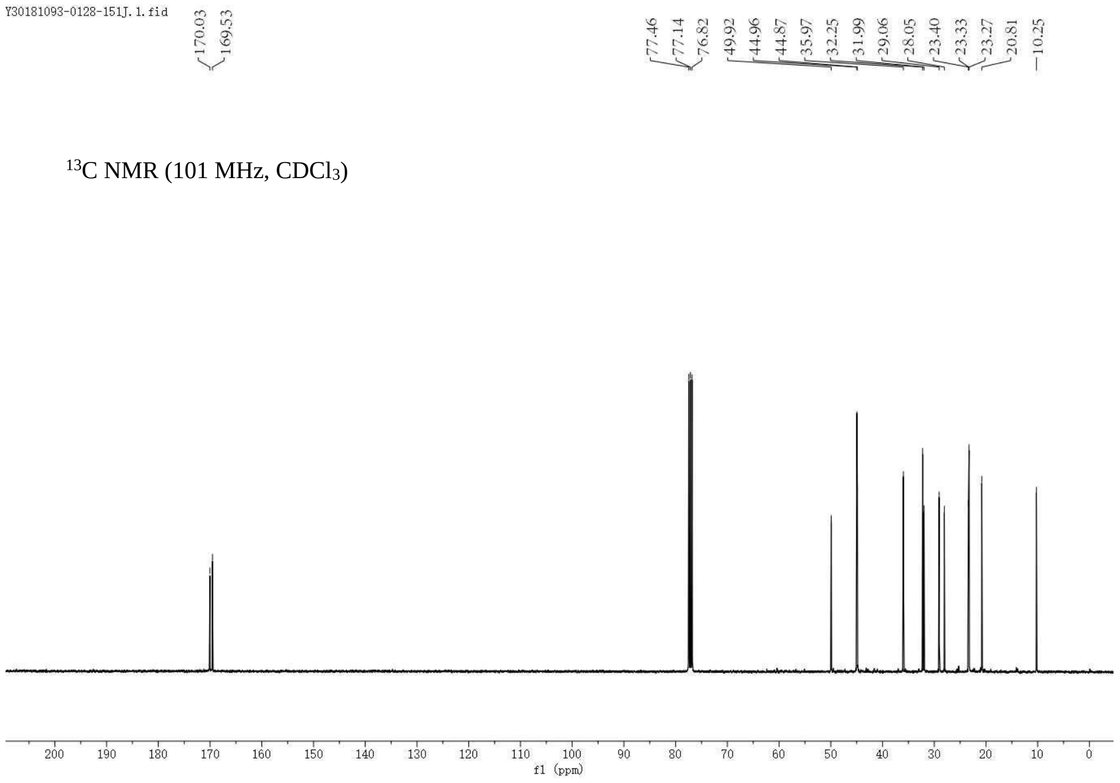

Supplementary Figure 26. ^{1}H NMR and ^{13}C NMR spectra of compound 19a and 19b.

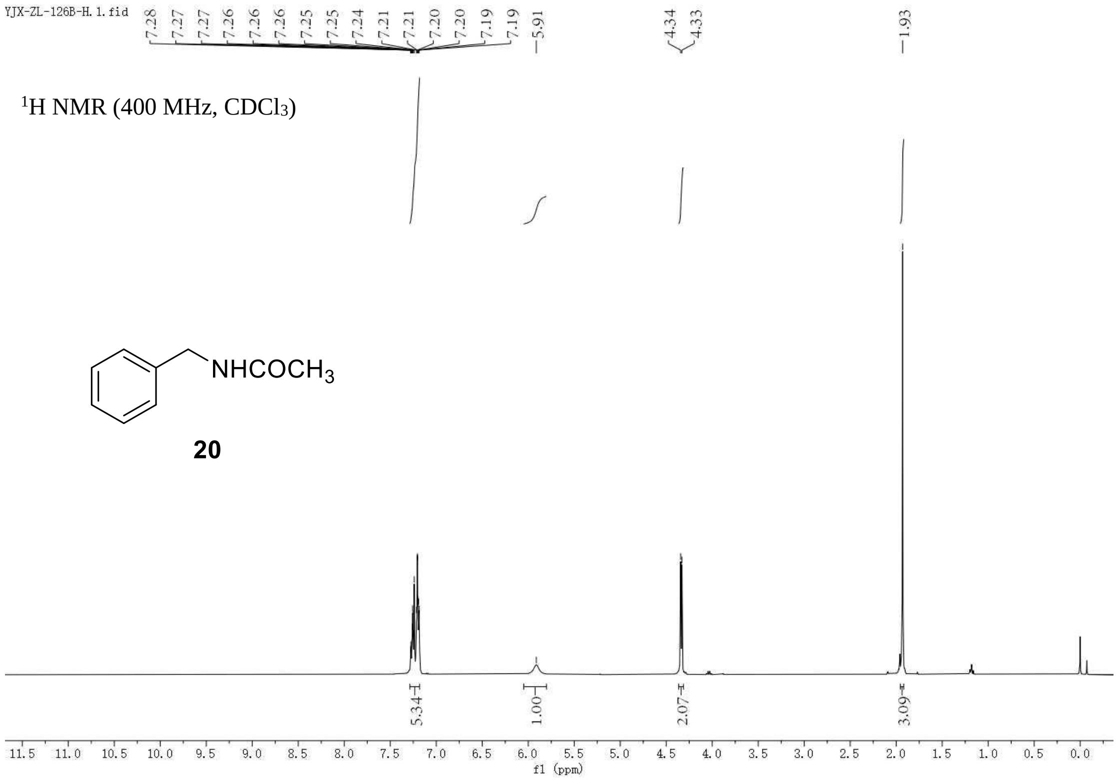

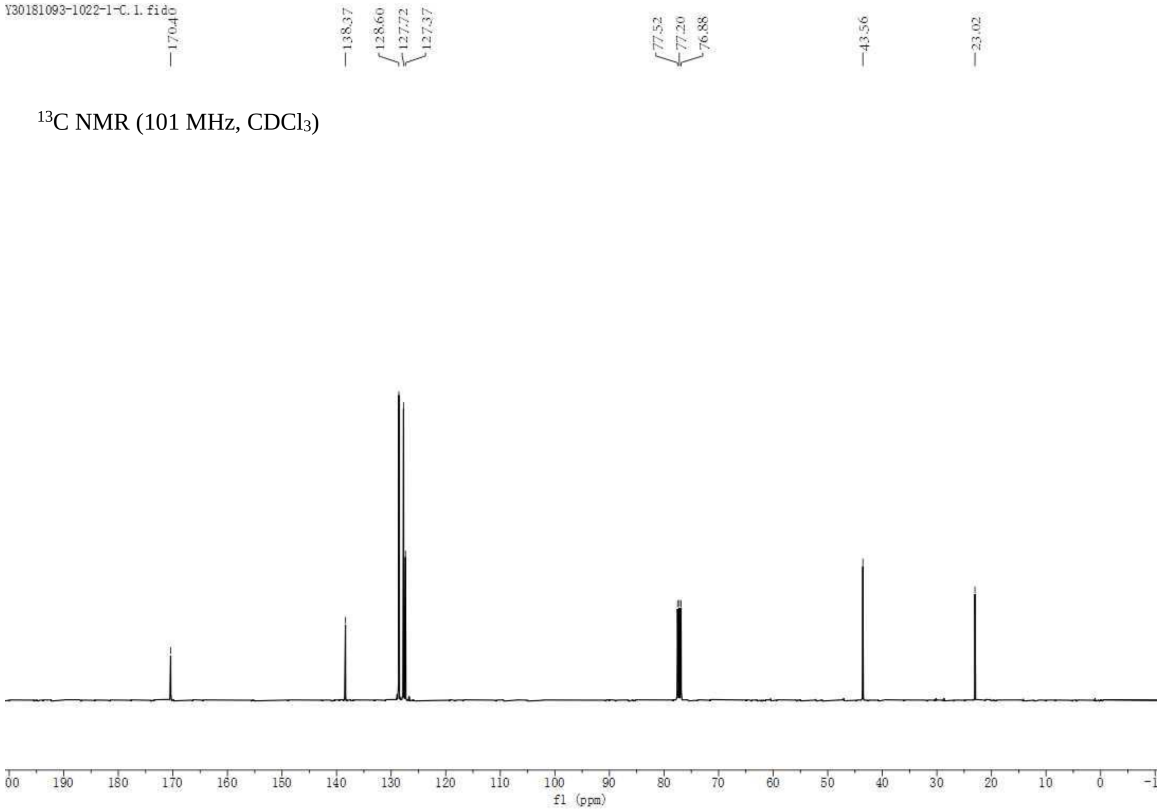

Supplementary Figure 27. ^{1}H NMR and ^{13}C NMR spectra of compound 20.

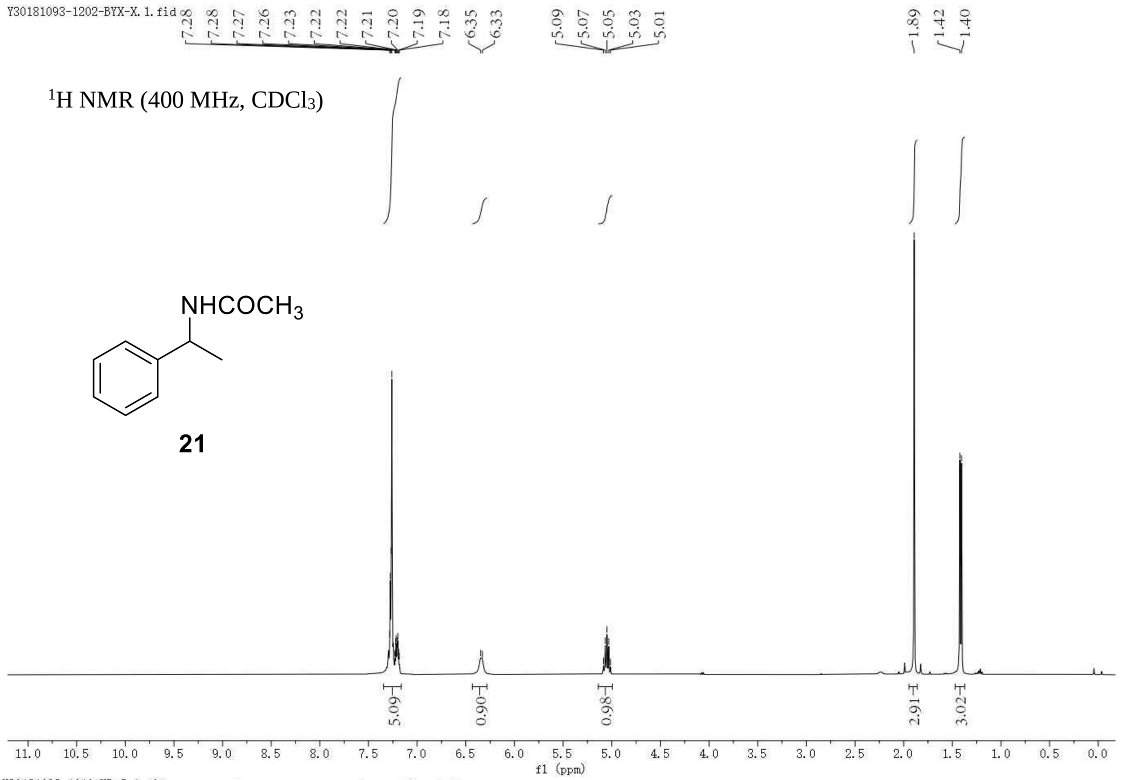

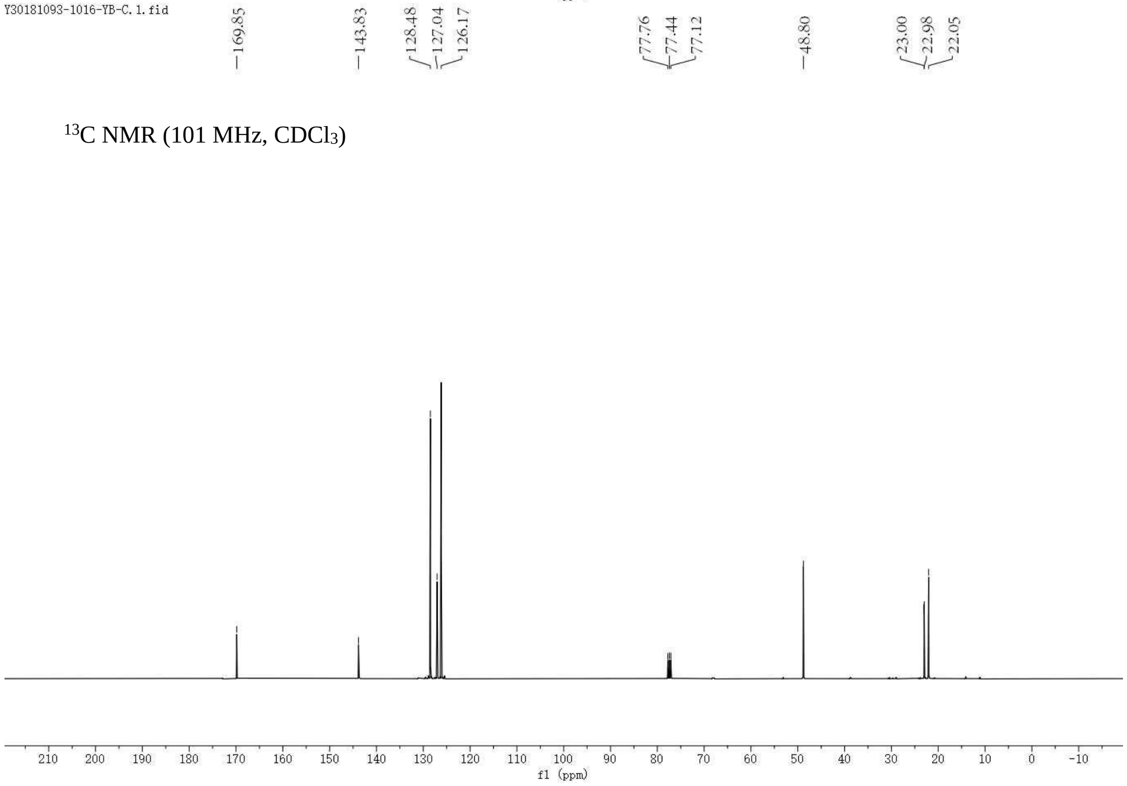

Supplementary Figure 28. ^{1}H NMR and ^{13}C NMR spectra of compound 21.

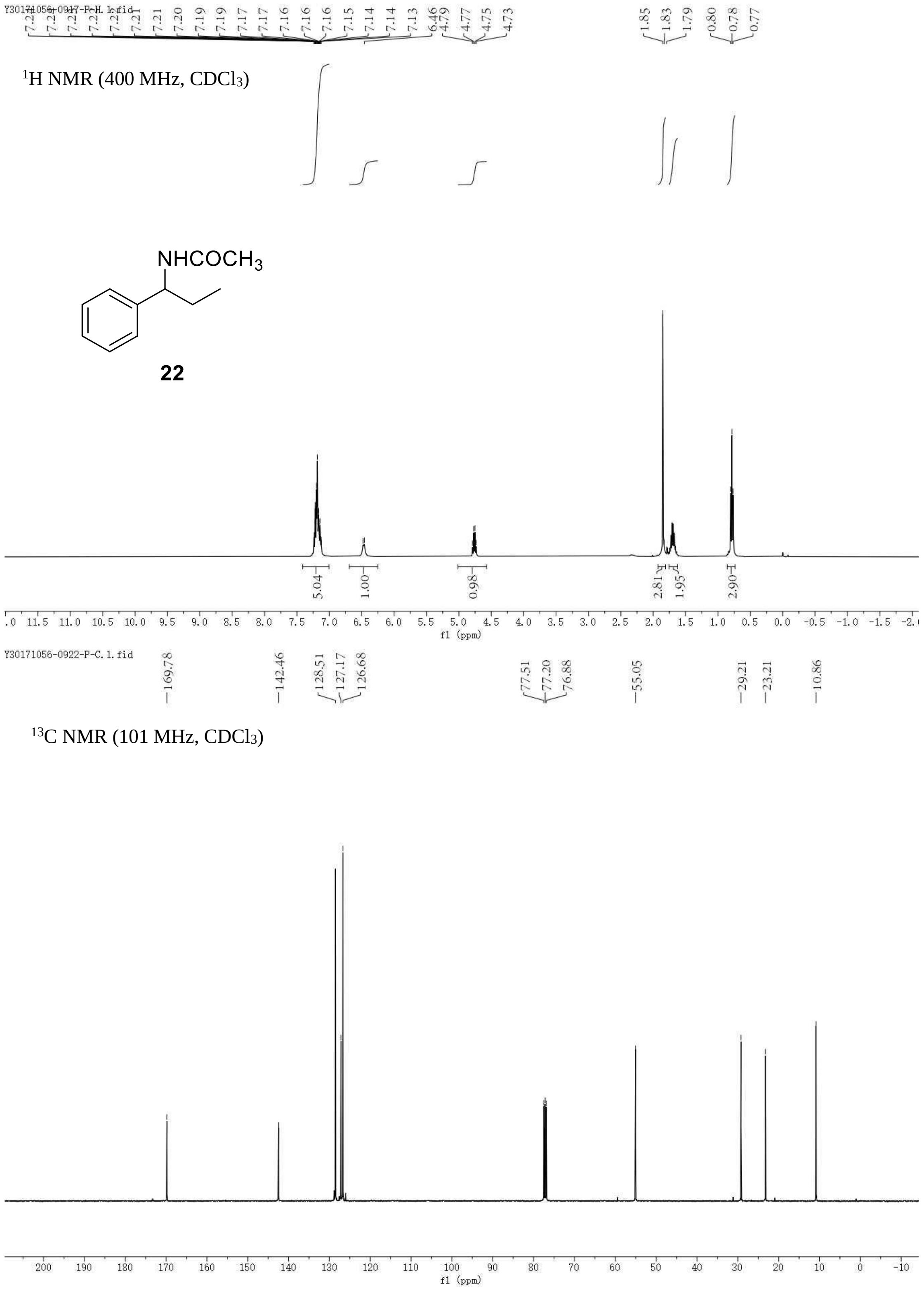

Supplementary Figure 29. ^{1}H NMR and ^{13}C NMR spectra of compound 22.

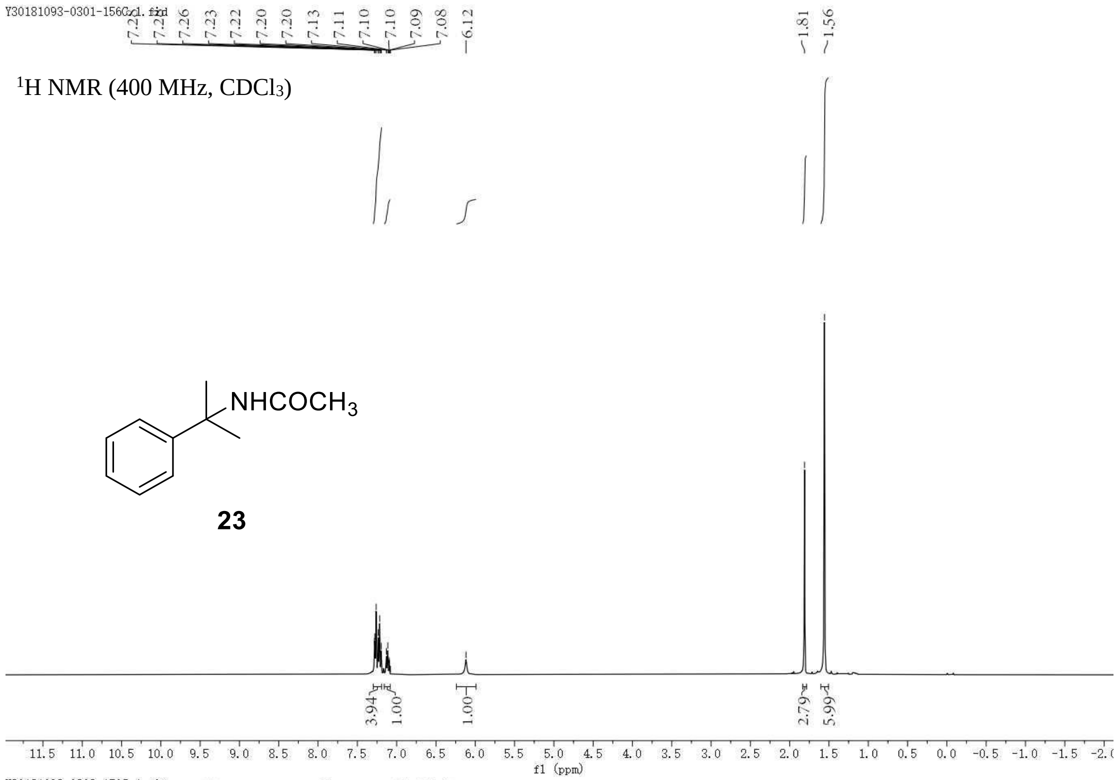

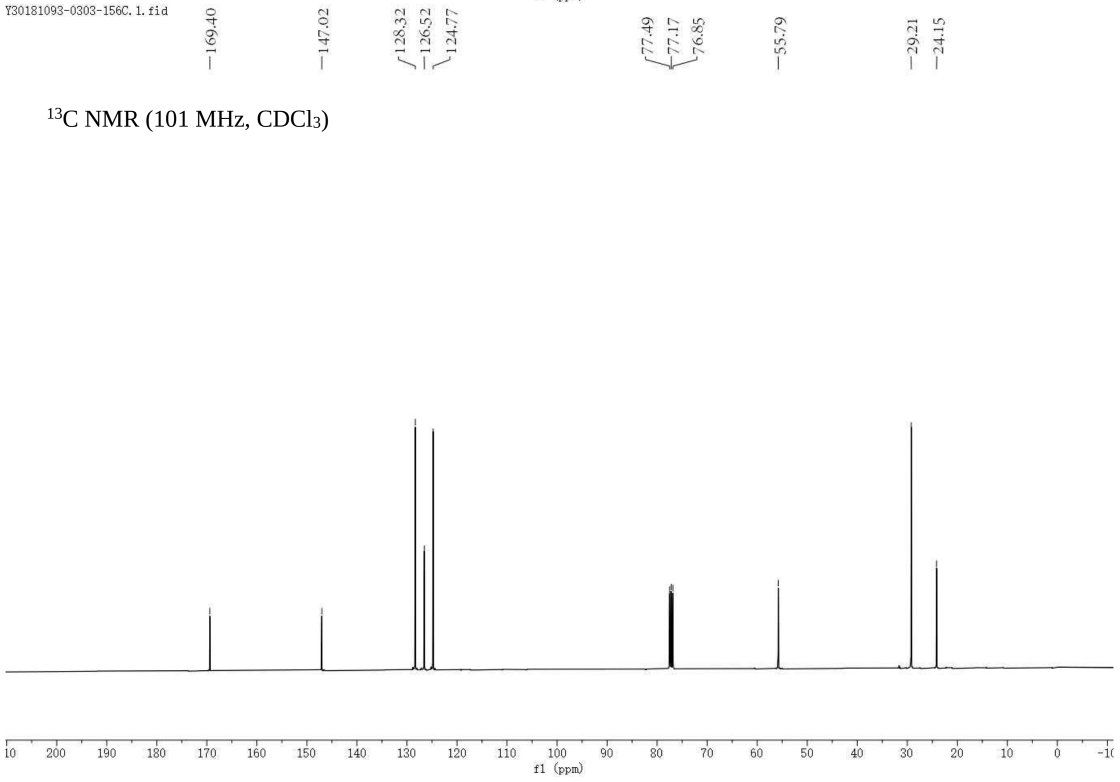

Supplementary Figure 30. ^{1}H NMR and ^{13}C NMR spectra of compound 23.

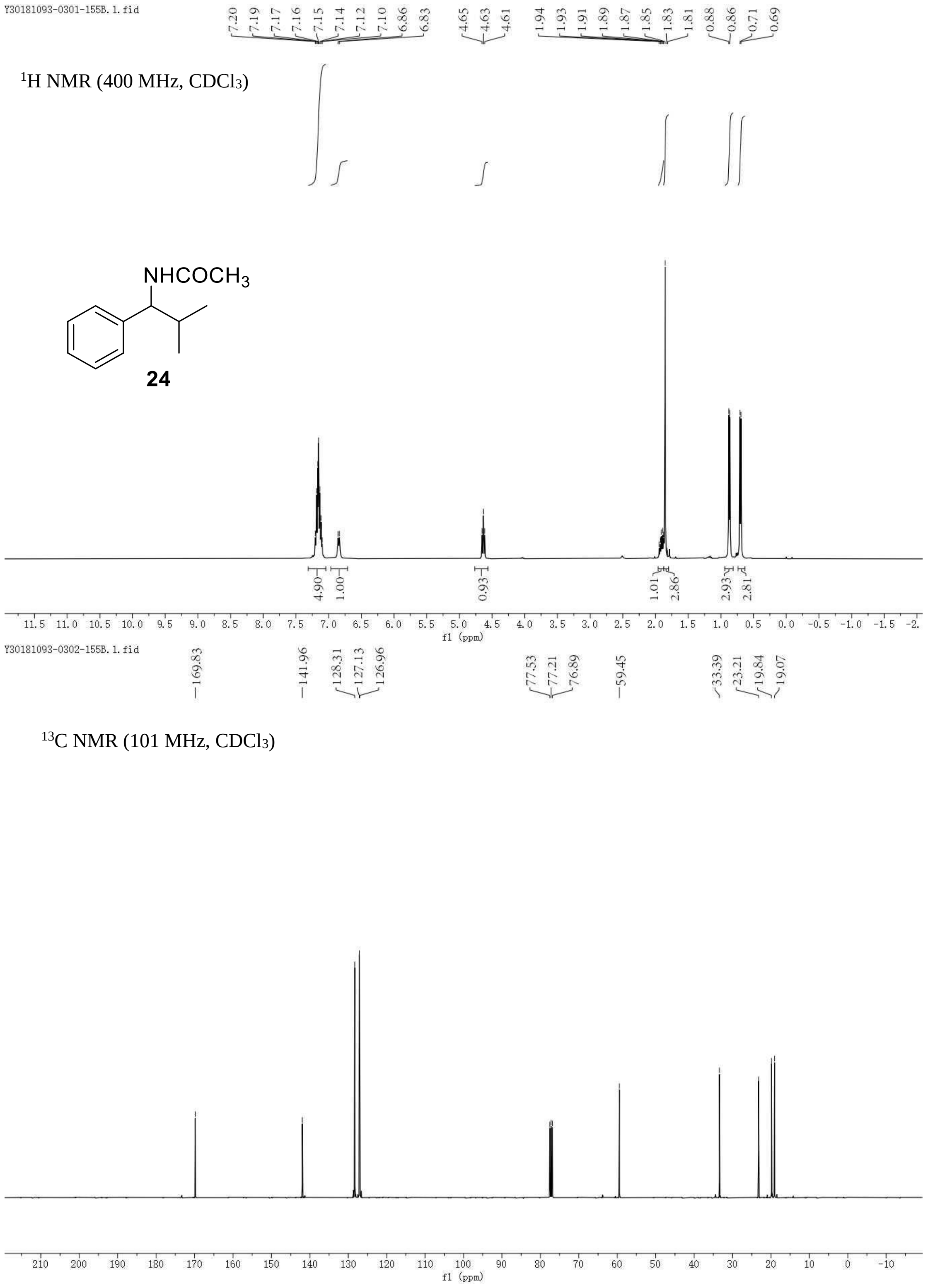

Supplementary Figure 31. ^{1}H NMR and ^{13}C NMR spectra of compound 24.

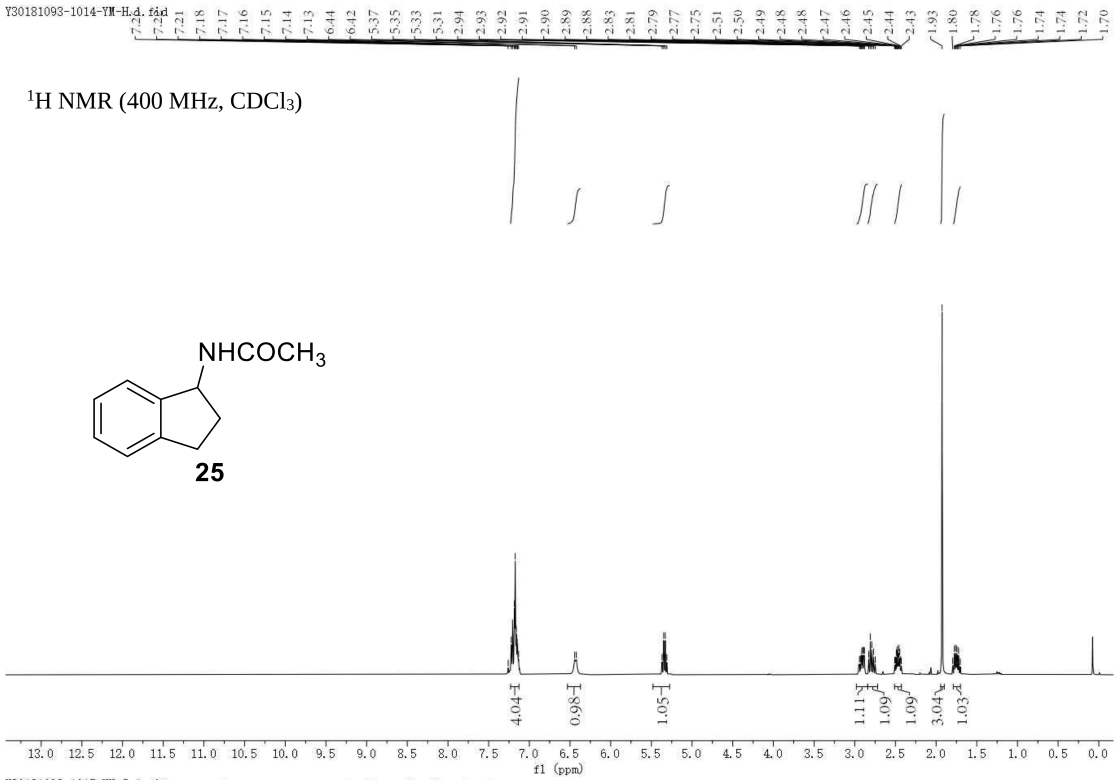

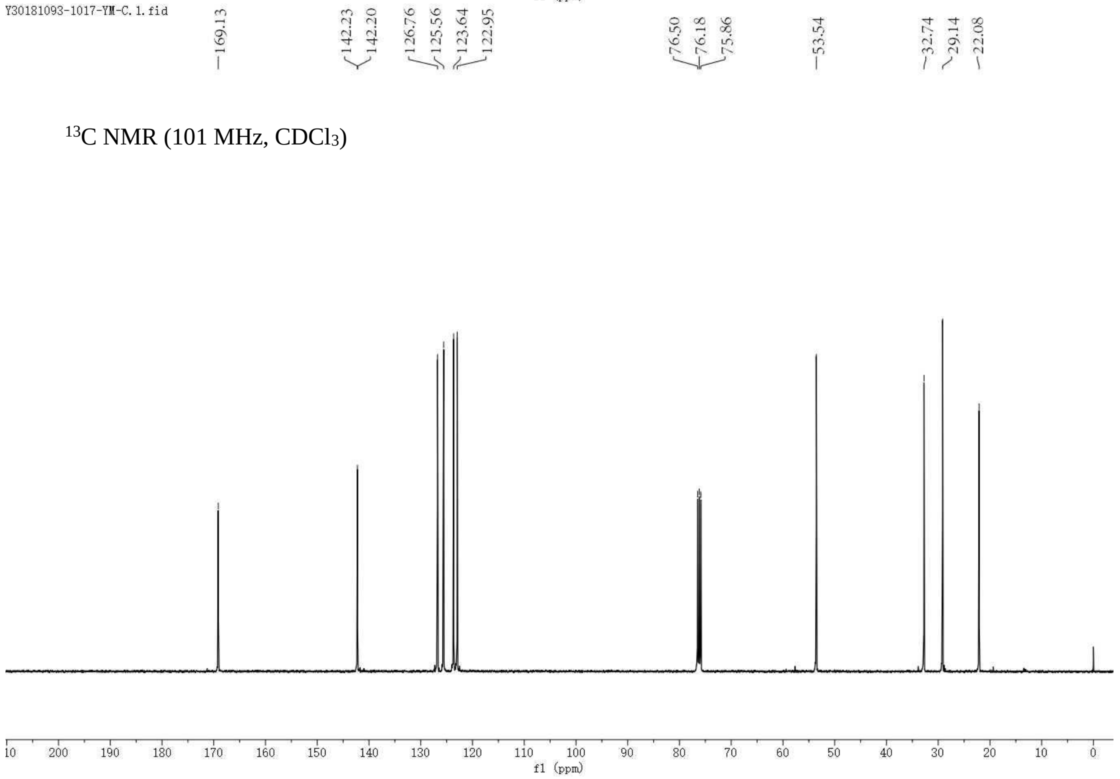

Supplementary Figure 32. ^{1}H NMR and ^{13}C NMR spectra of compound 25.

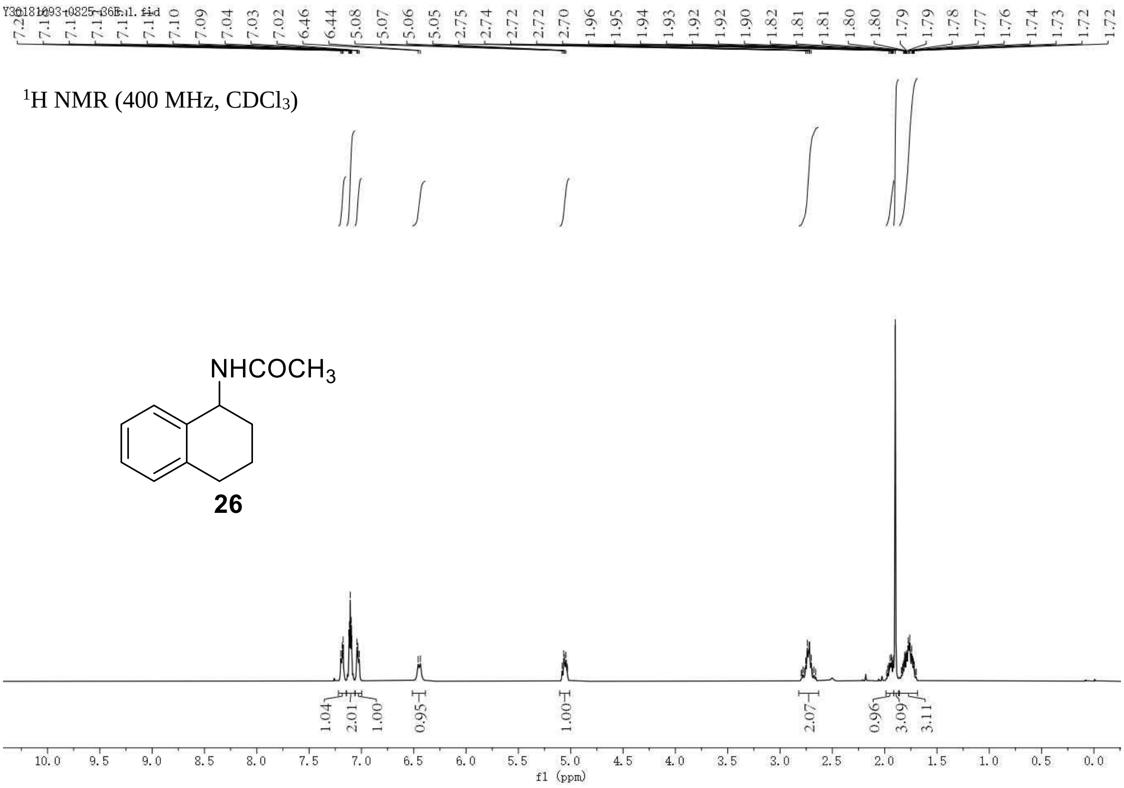

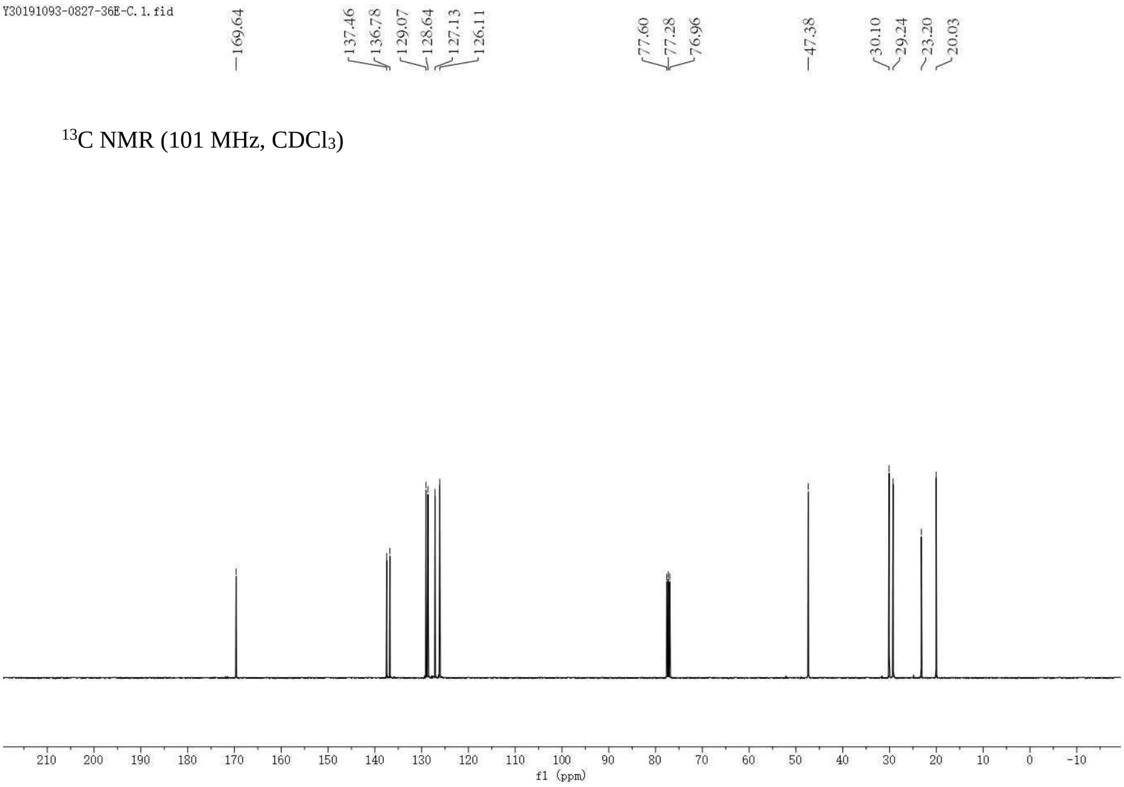

Supplementary Figure 33. ^{1}H NMR and ^{13}C NMR spectra of compound 26.

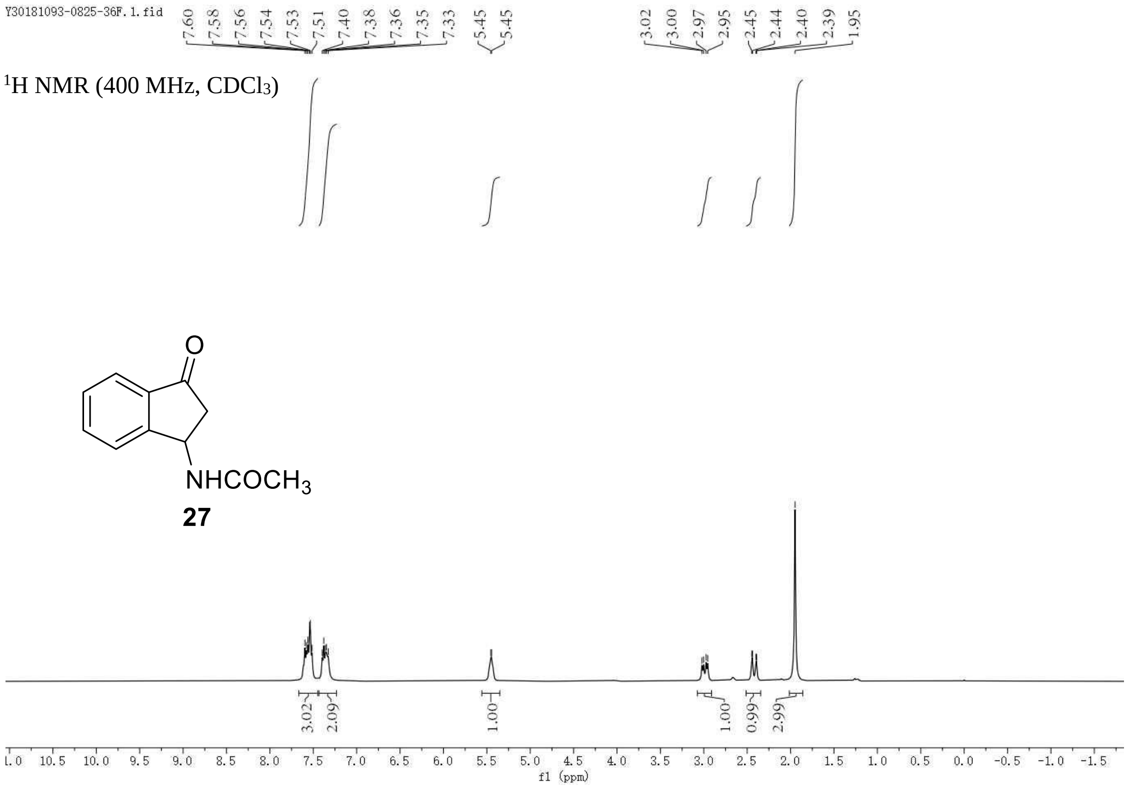

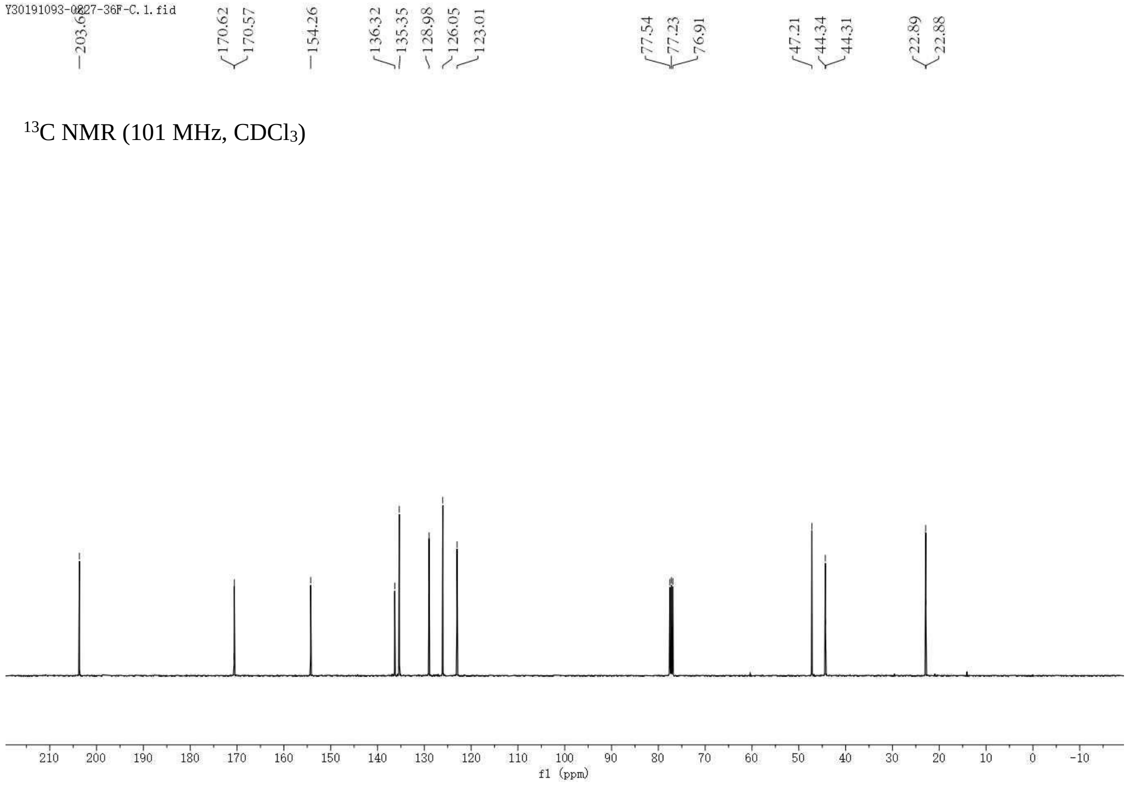

Supplementary Figure 34. ^{1}H NMR and ^{13}C NMR spectra of compound 27.

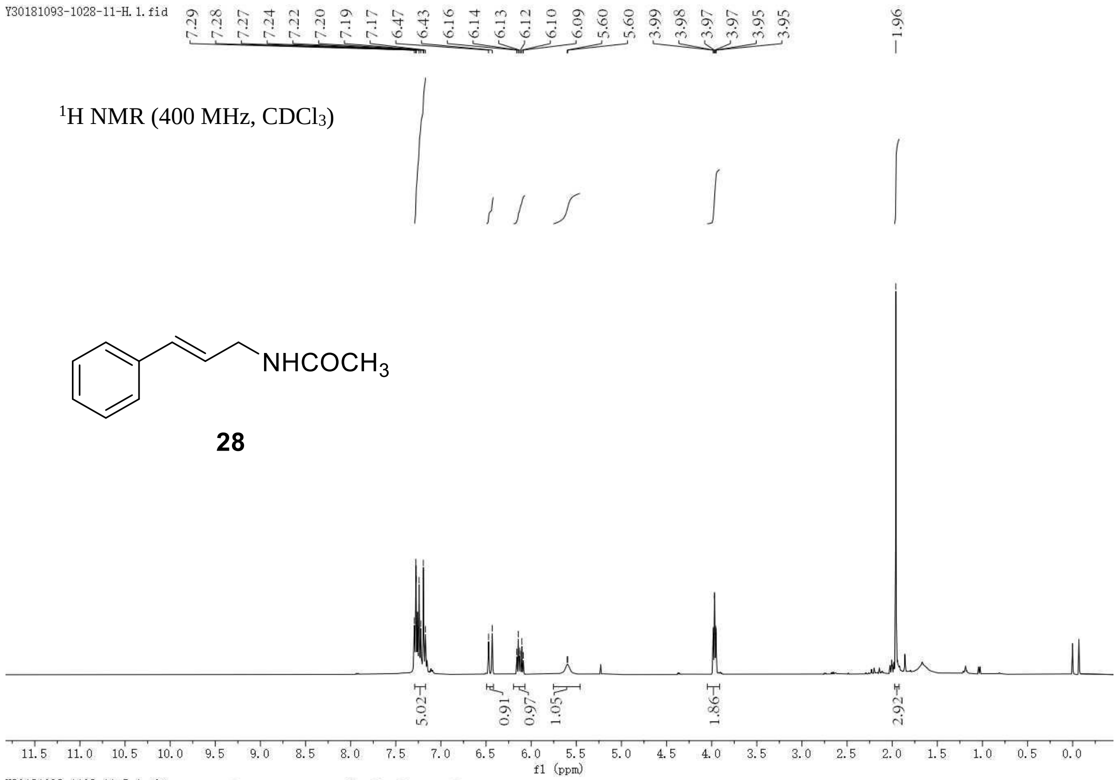

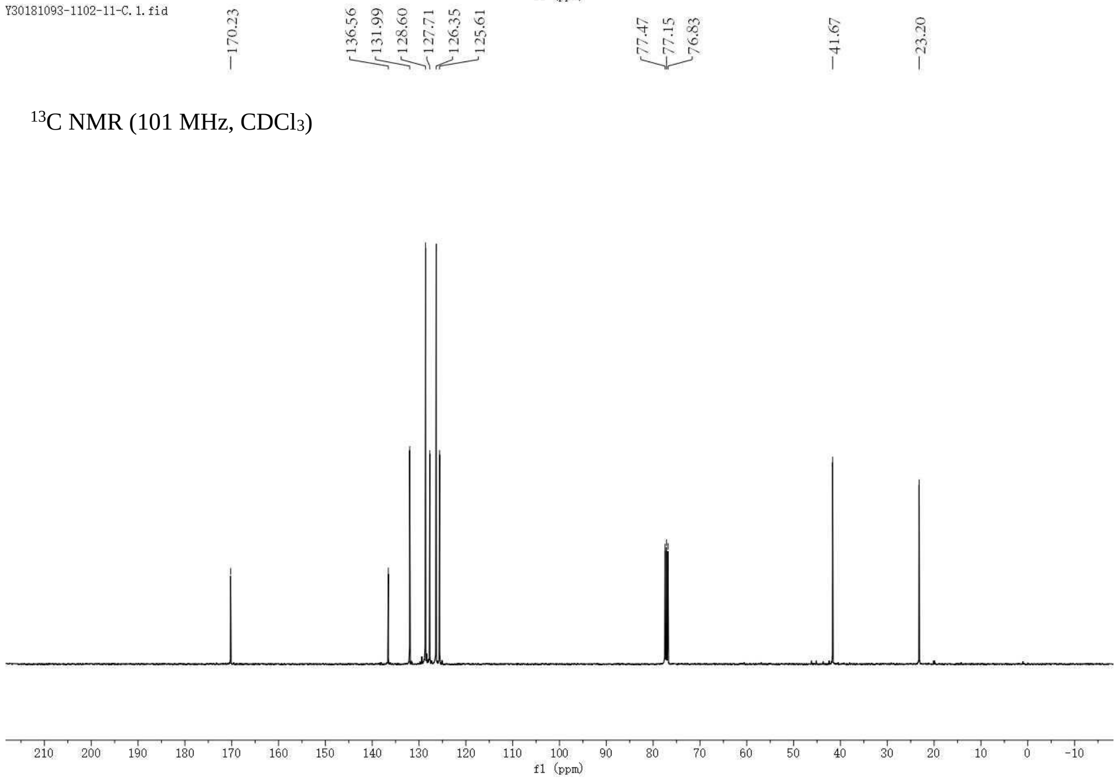

Supplementary Figure 35. ^{1}H NMR and ^{13}C NMR spectra of compound 28.

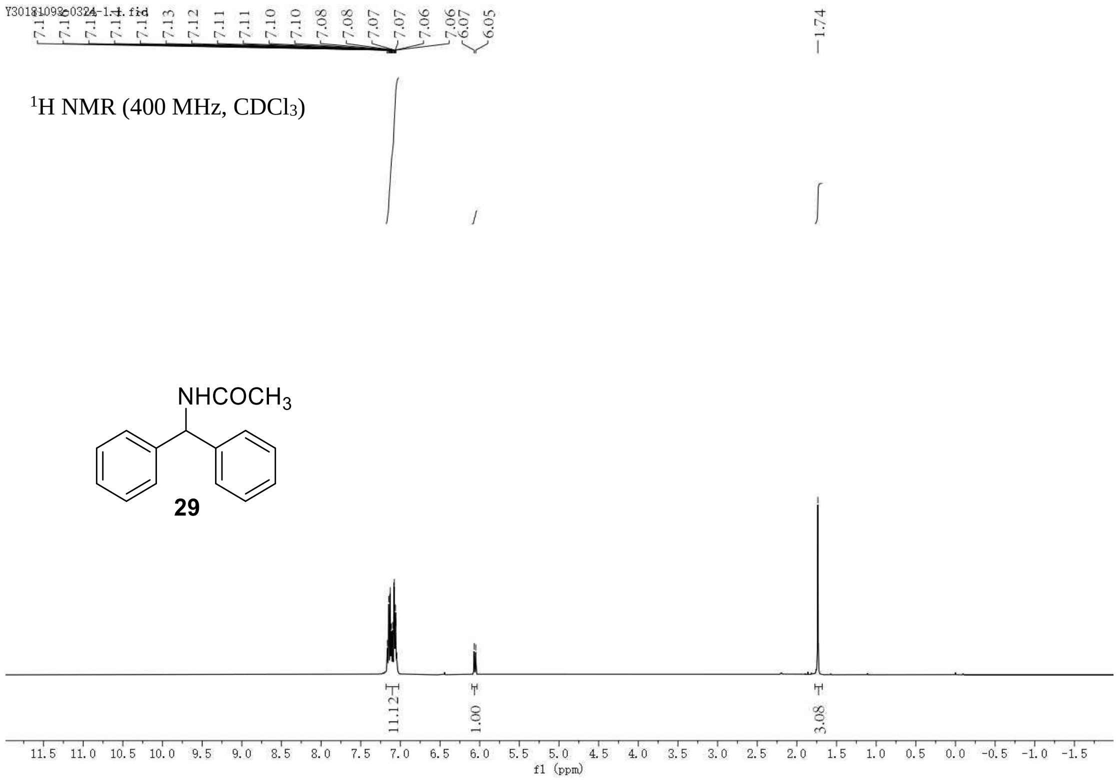

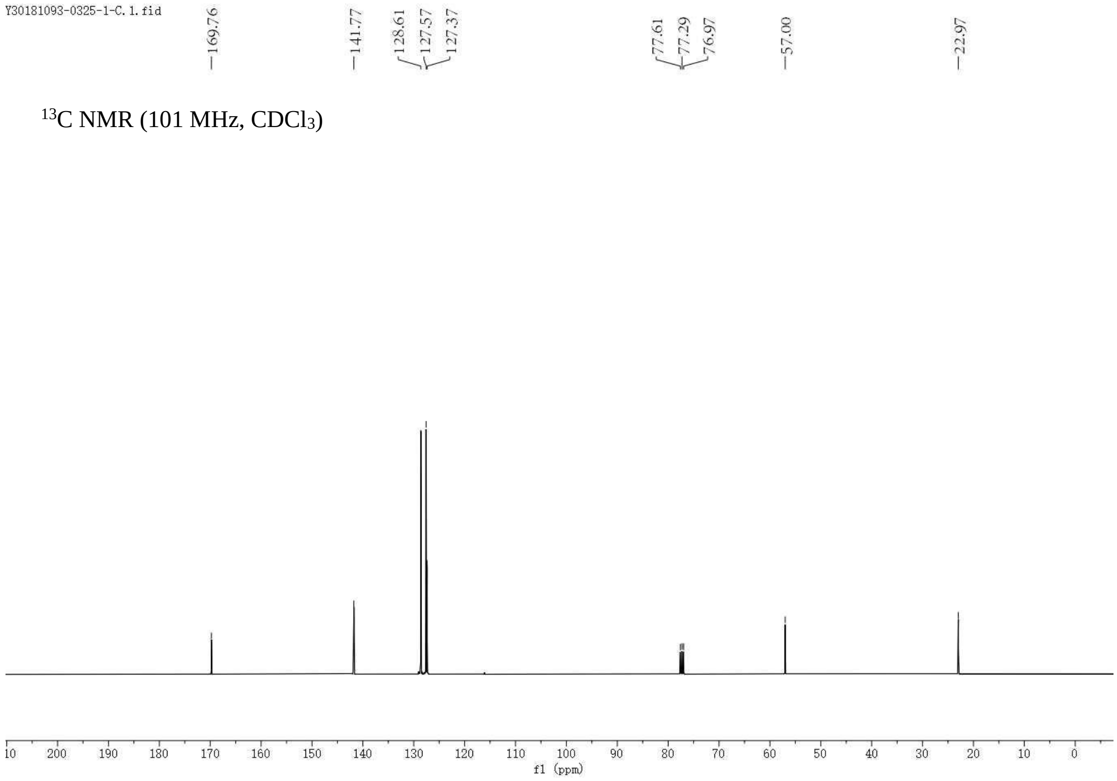

Supplementary Figure 36. ^{1}H NMR and ^{13}C NMR spectra of compound 29.

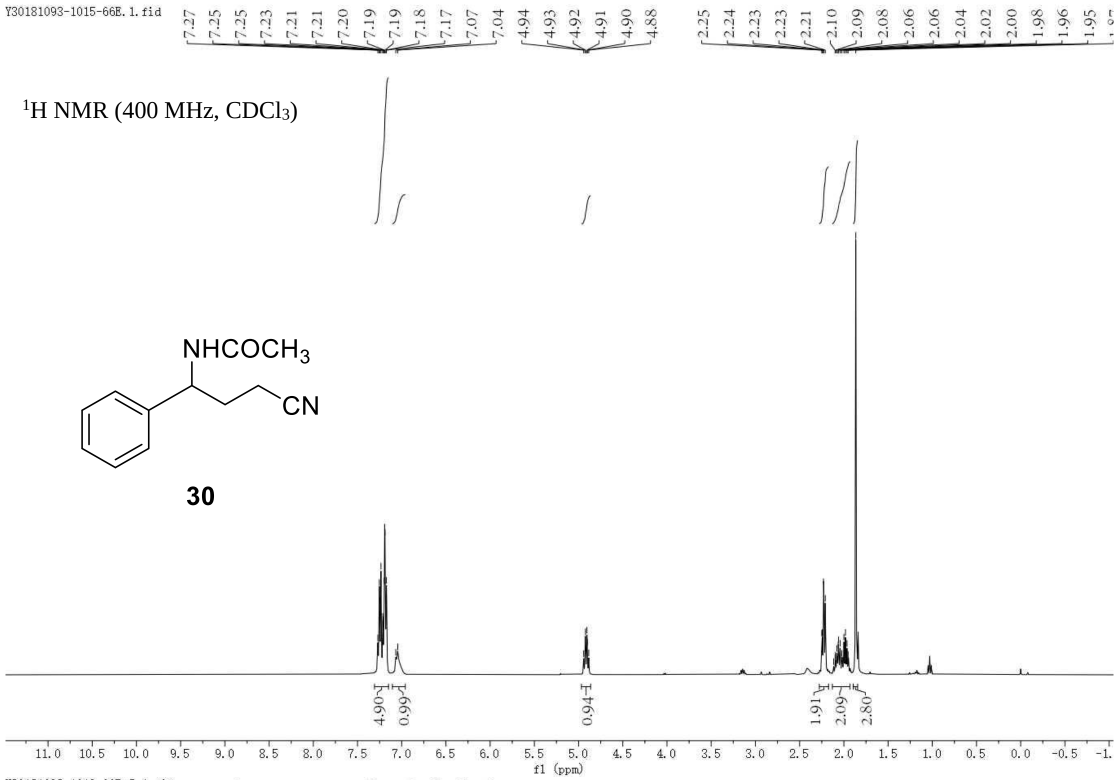

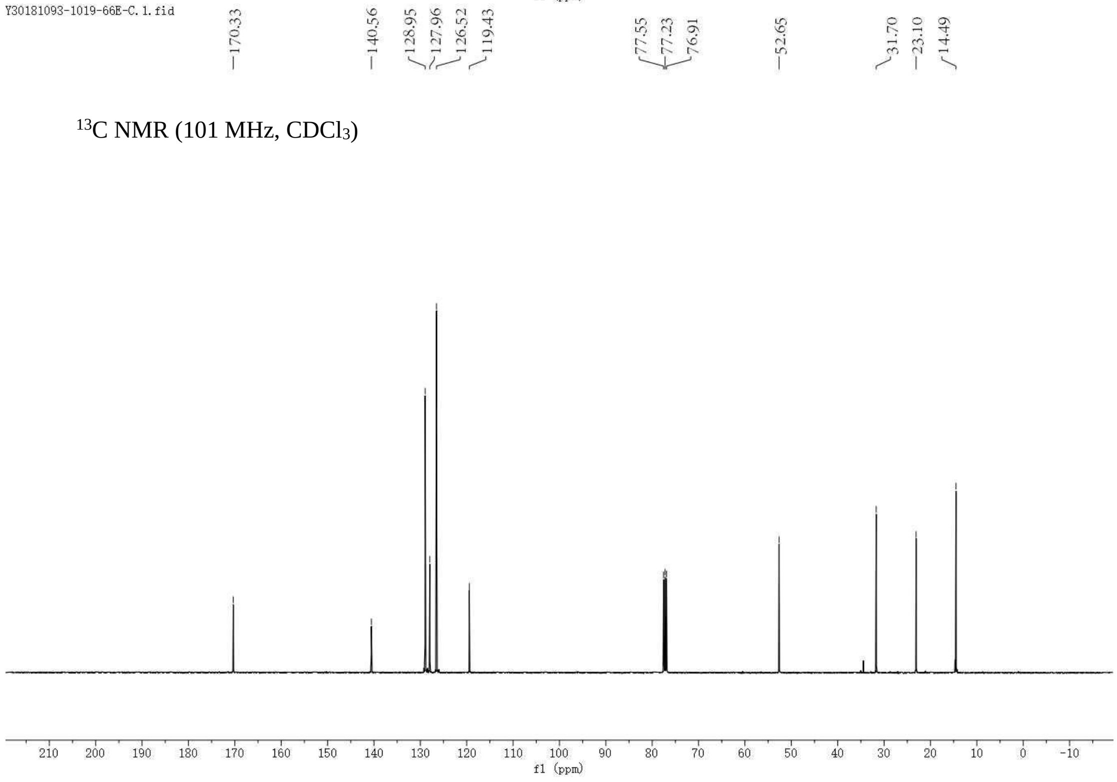

Supplementary Figure 37. ^{1}H NMR and ^{13}C NMR spectra of compound 30.

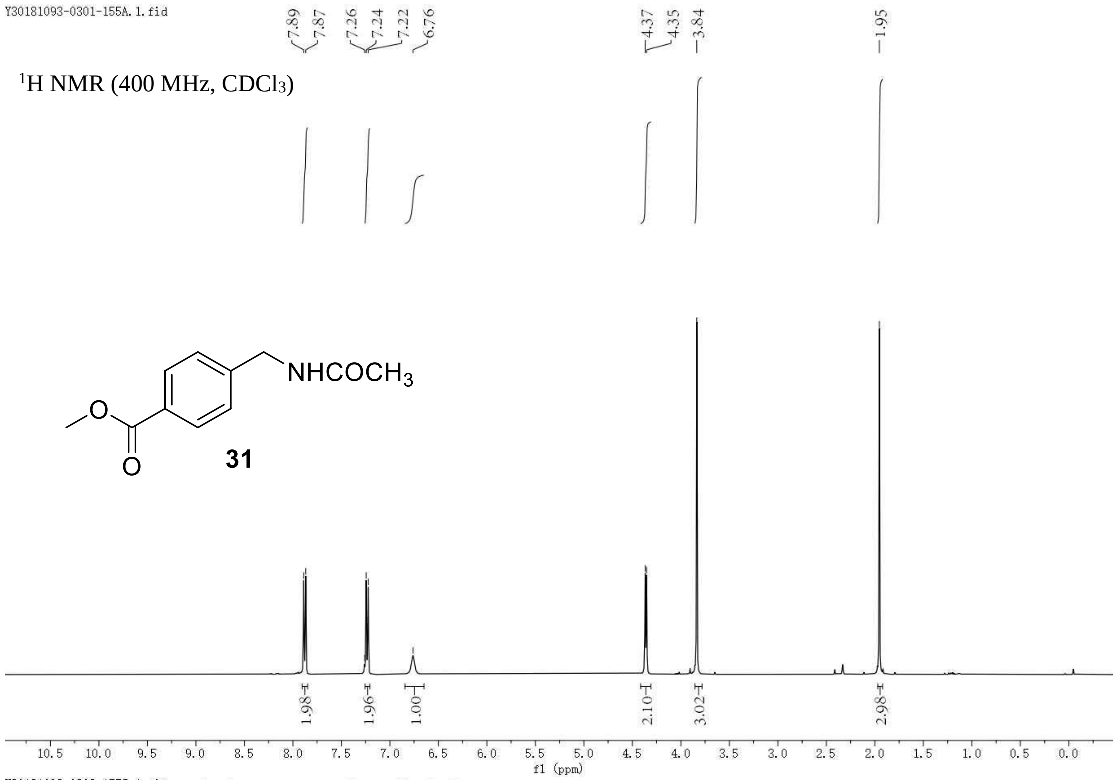

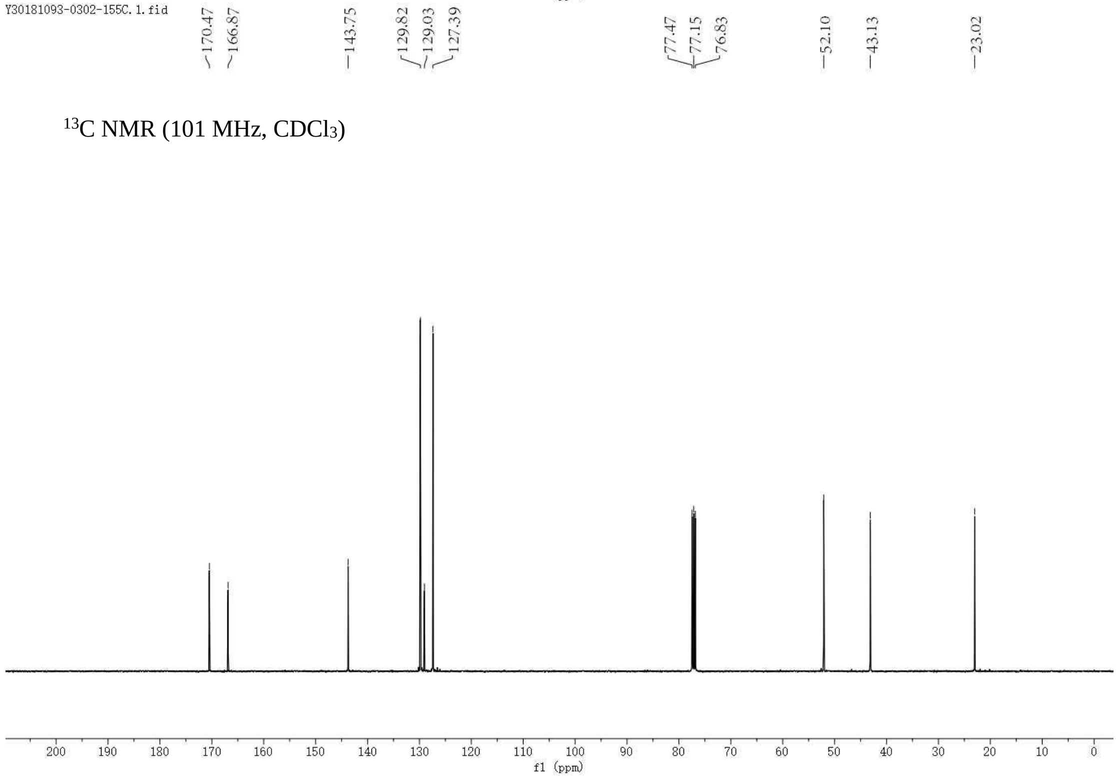

Supplementary Figure 38. ^{1}H NMR and ^{13}C NMR spectra of compound 31.

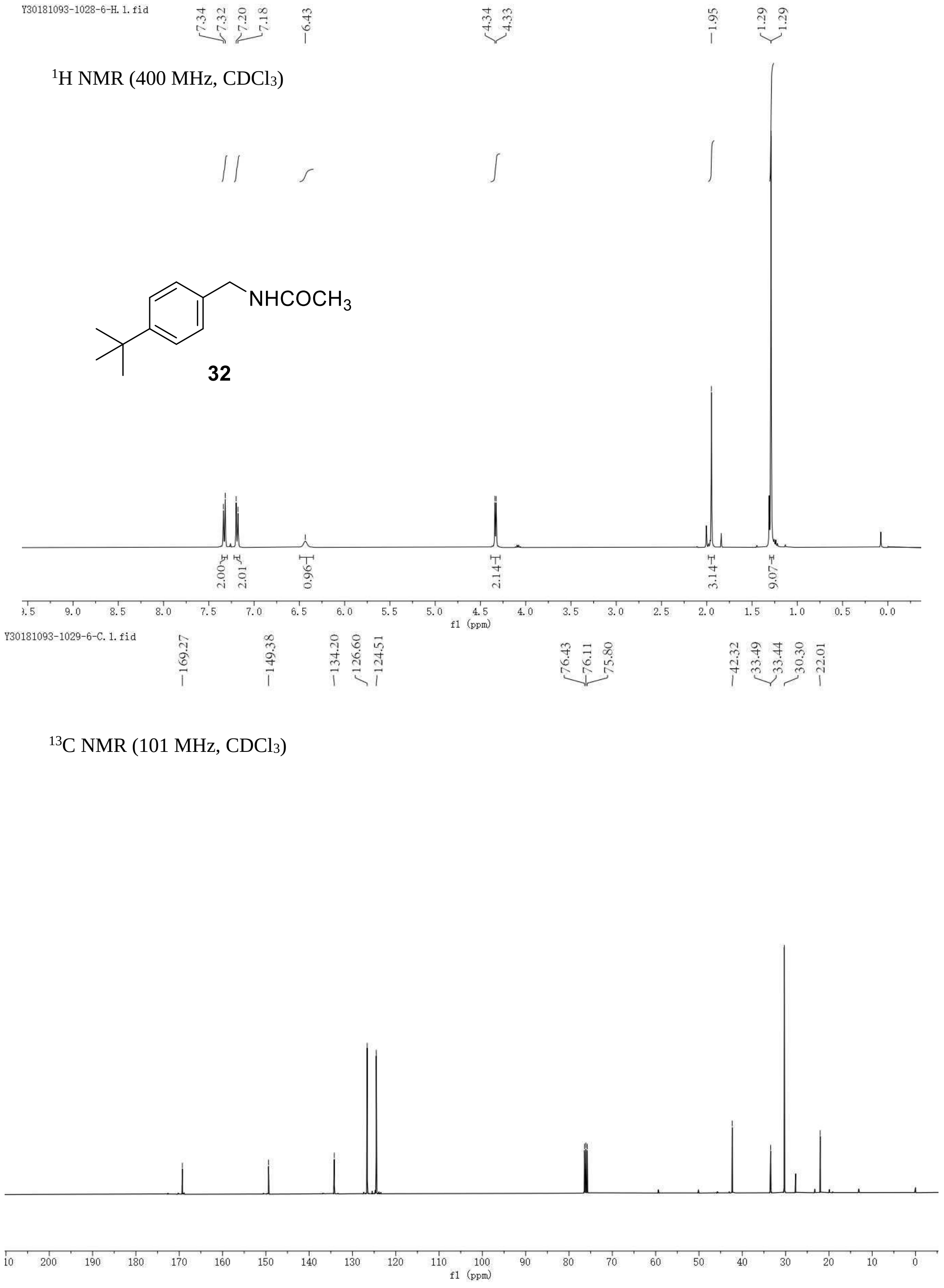

Supplementary Figure 39. ^{1}H NMR and ^{13}C NMR spectra of compound 32.

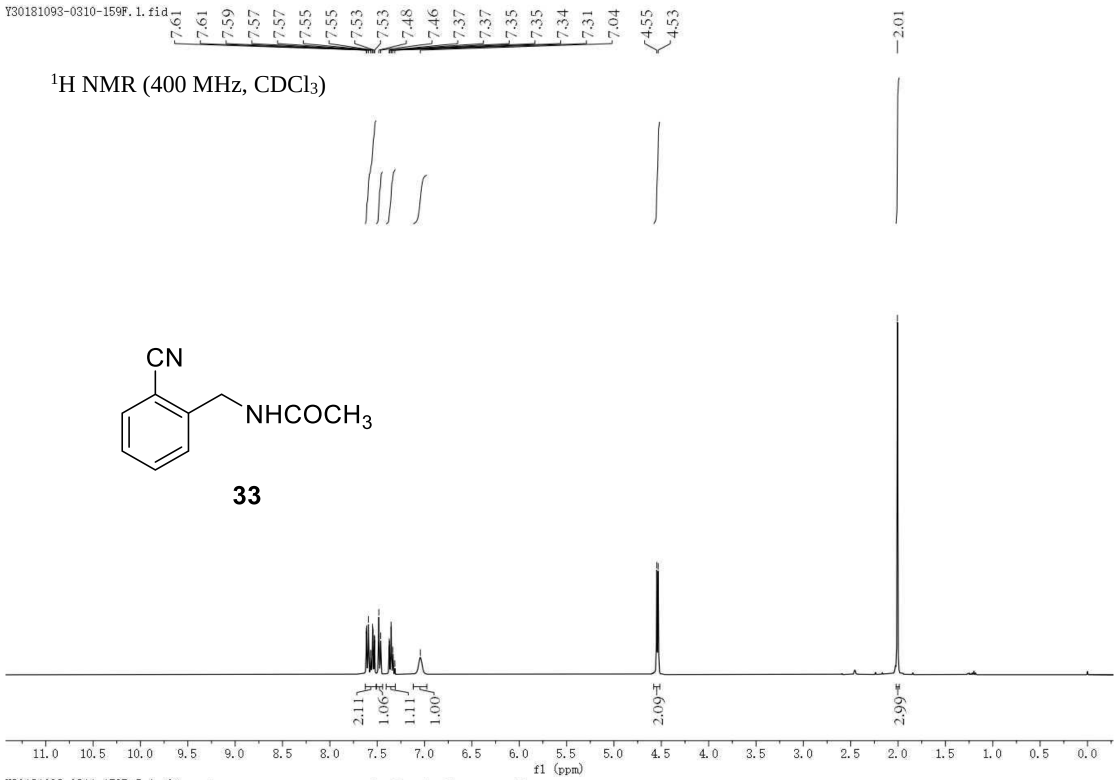

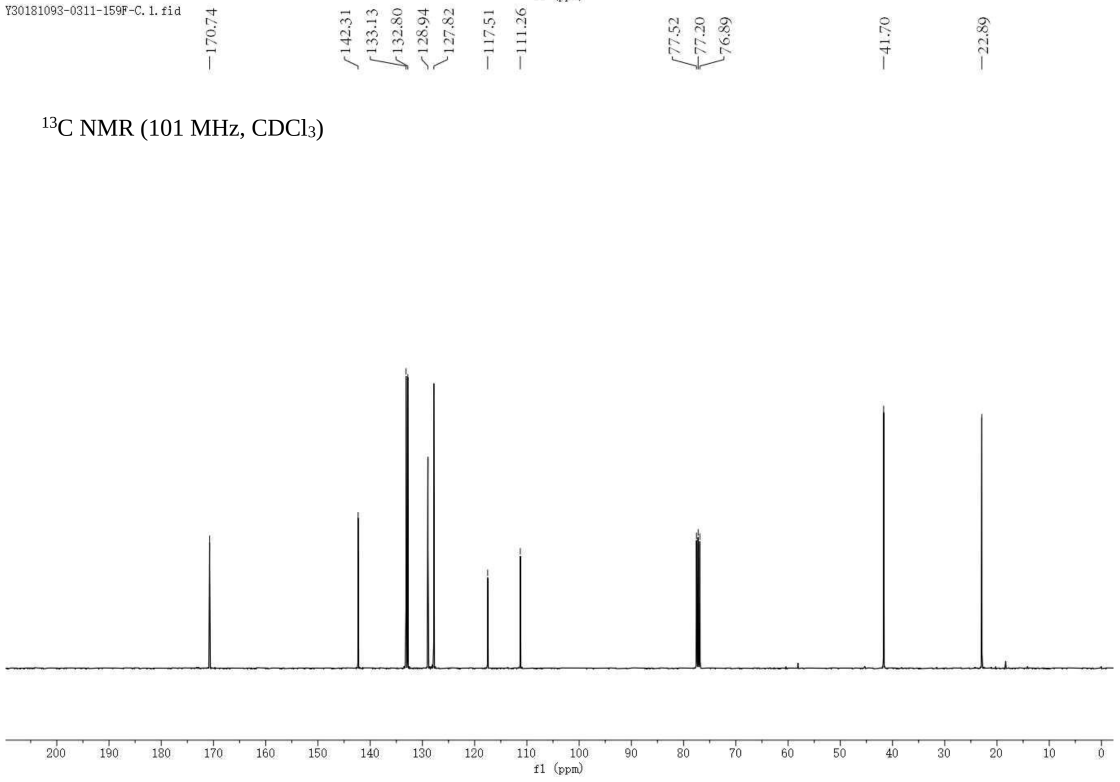

Supplementary Figure 40. ^{1}H NMR and ^{13}C NMR spectra of compound 33.

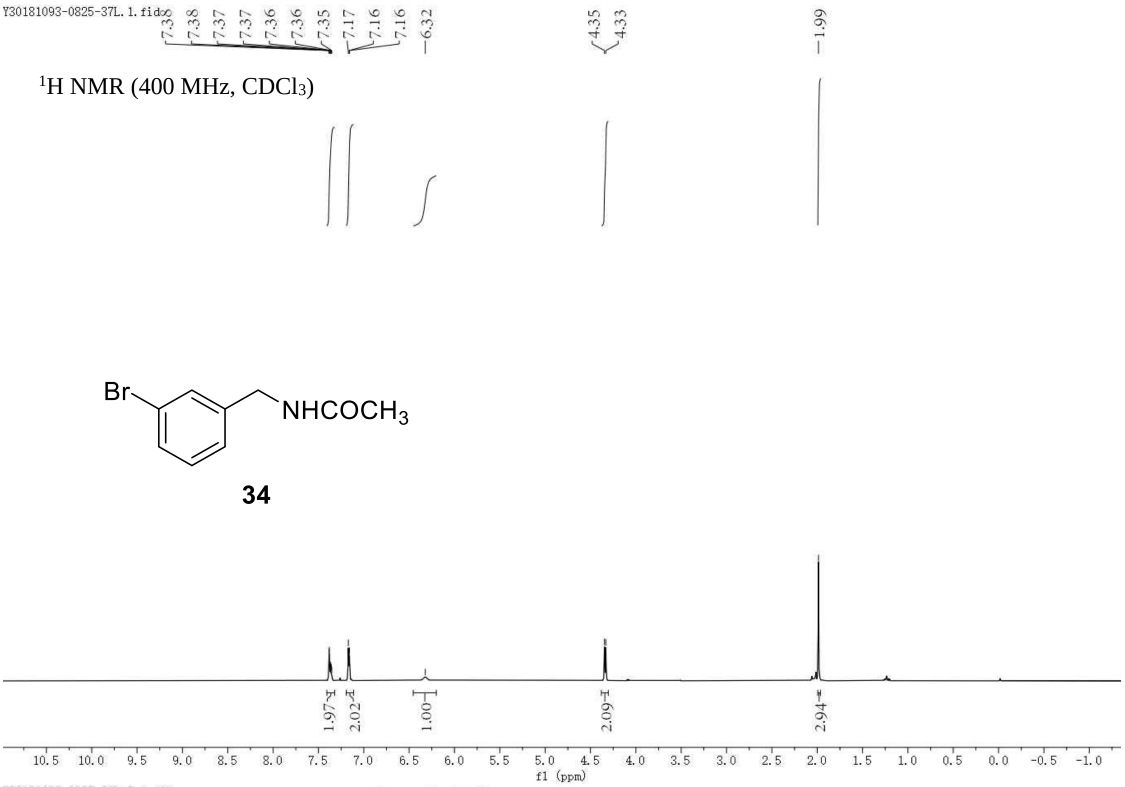

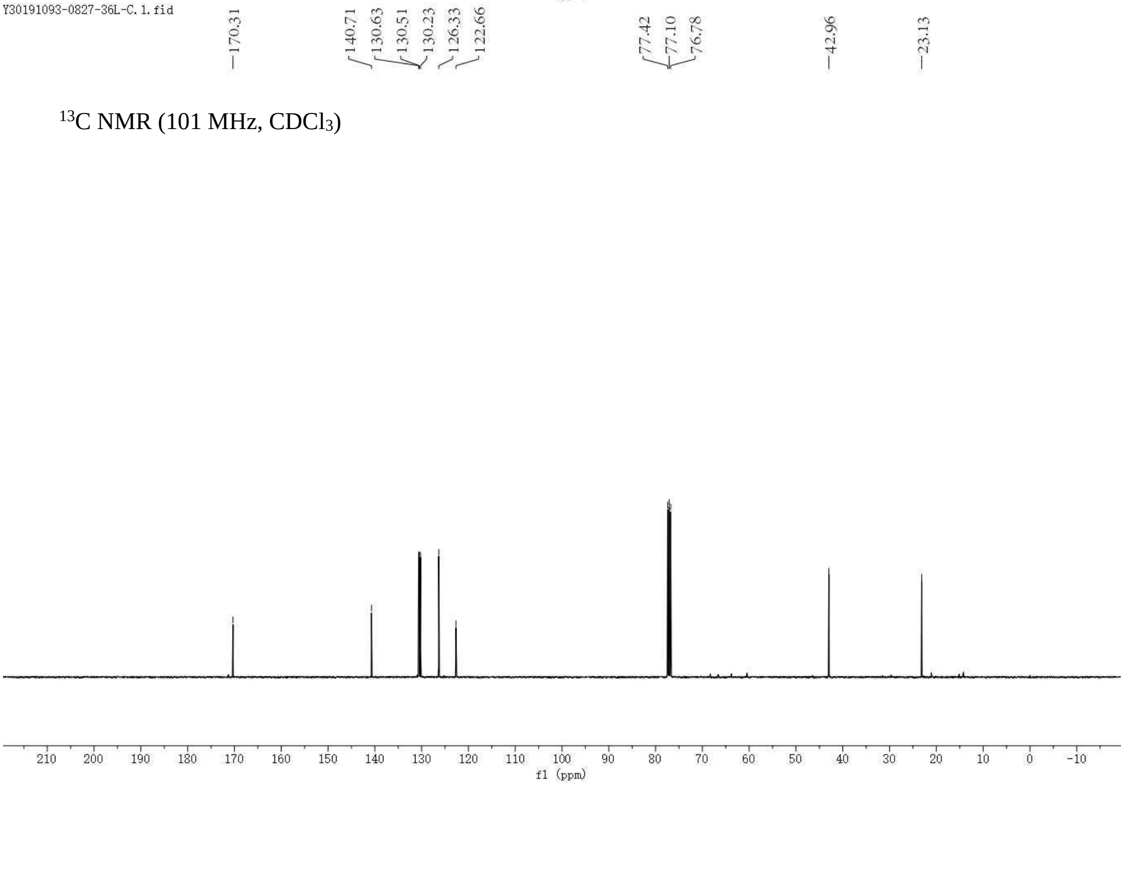

Supplementary Figure 41. ^{1}H NMR and ^{13}C NMR spectra of compound 34.

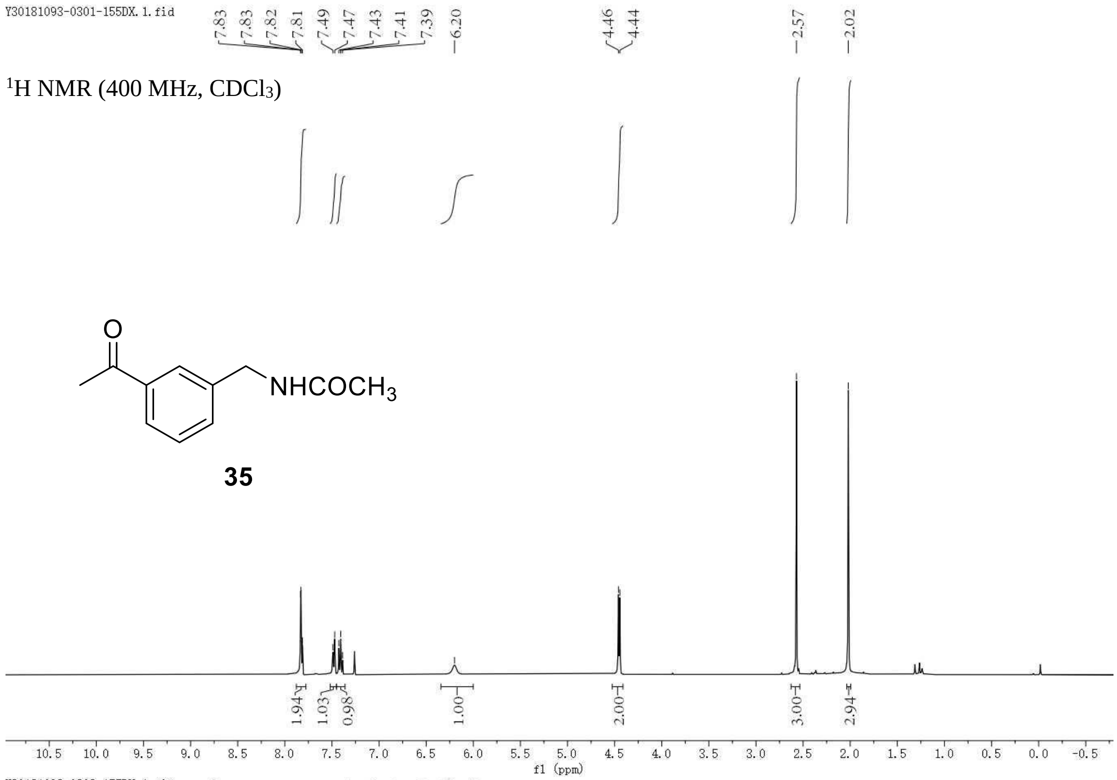

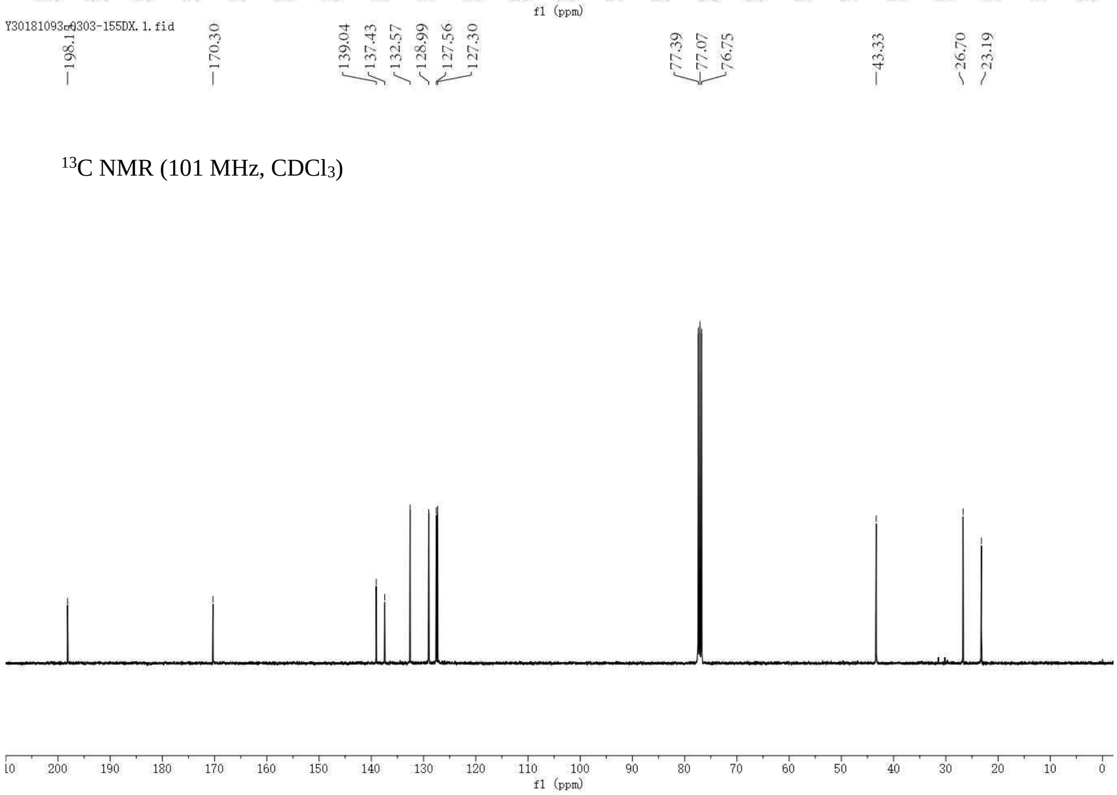

Supplementary Figure 42. ^{1}H NMR and ^{13}C NMR spectra of compound 35.

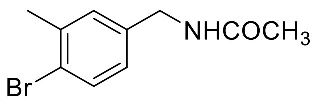

^{1}H NMR (400 MHz, $CDCl_3$)

36

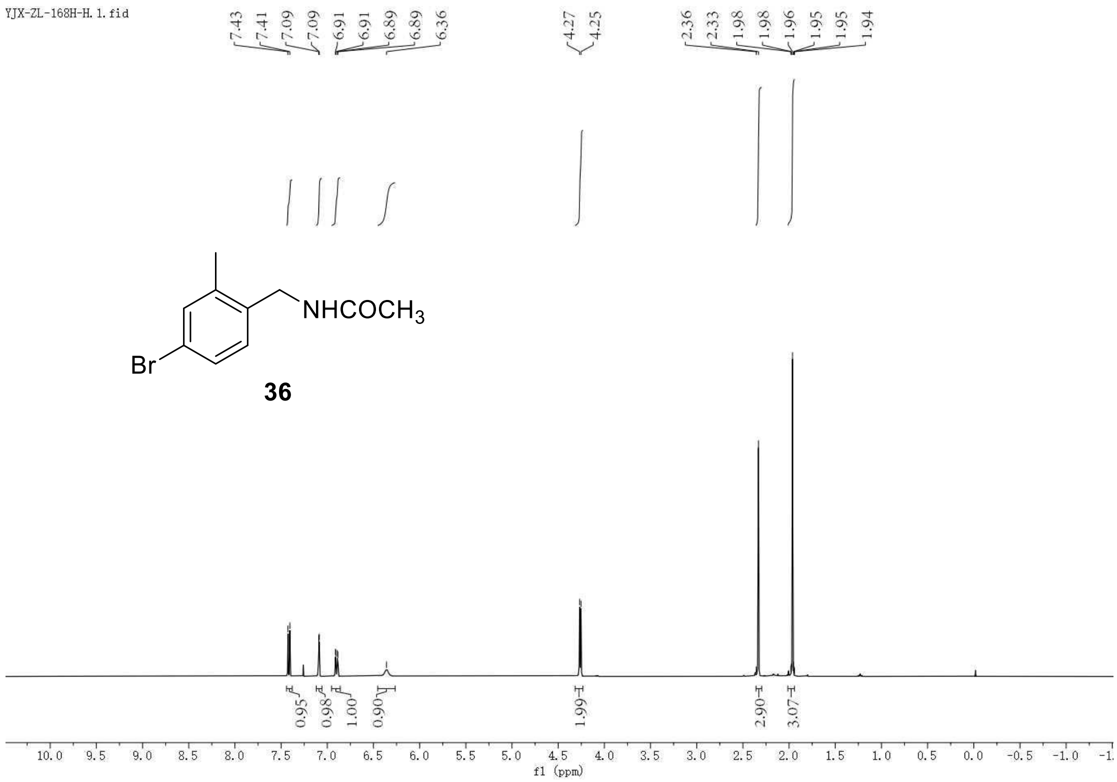

^{13}C NMR (101 MHz, $CDCl_3$)

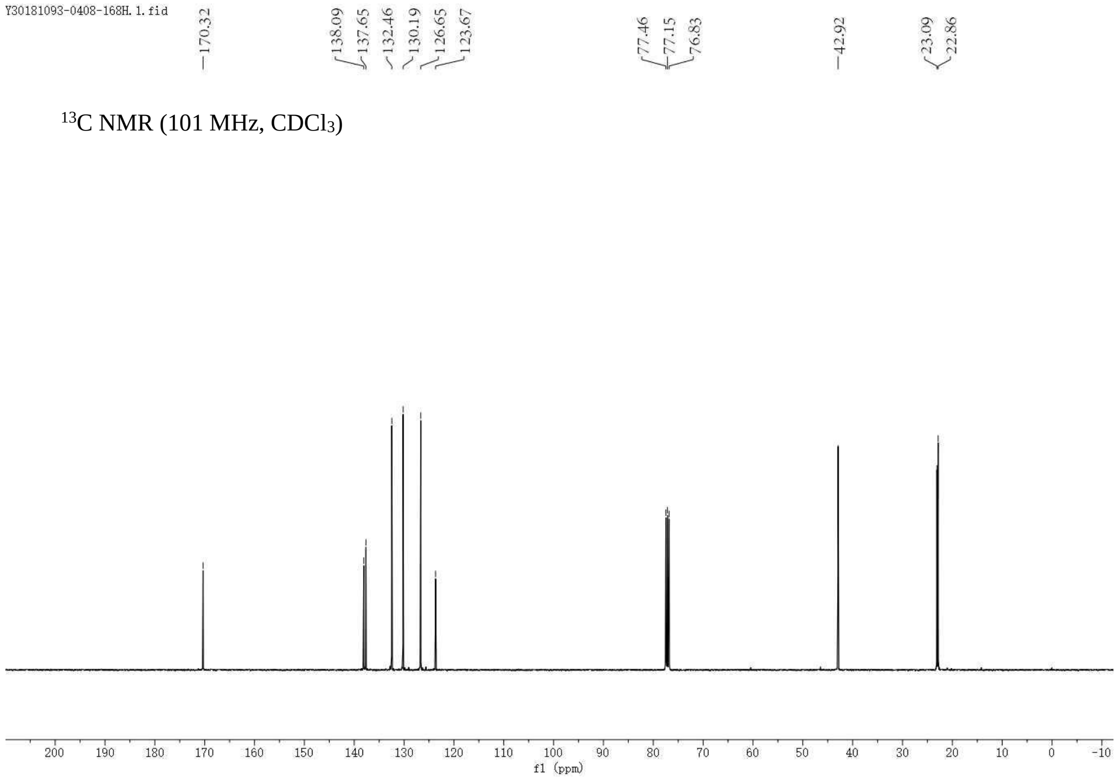

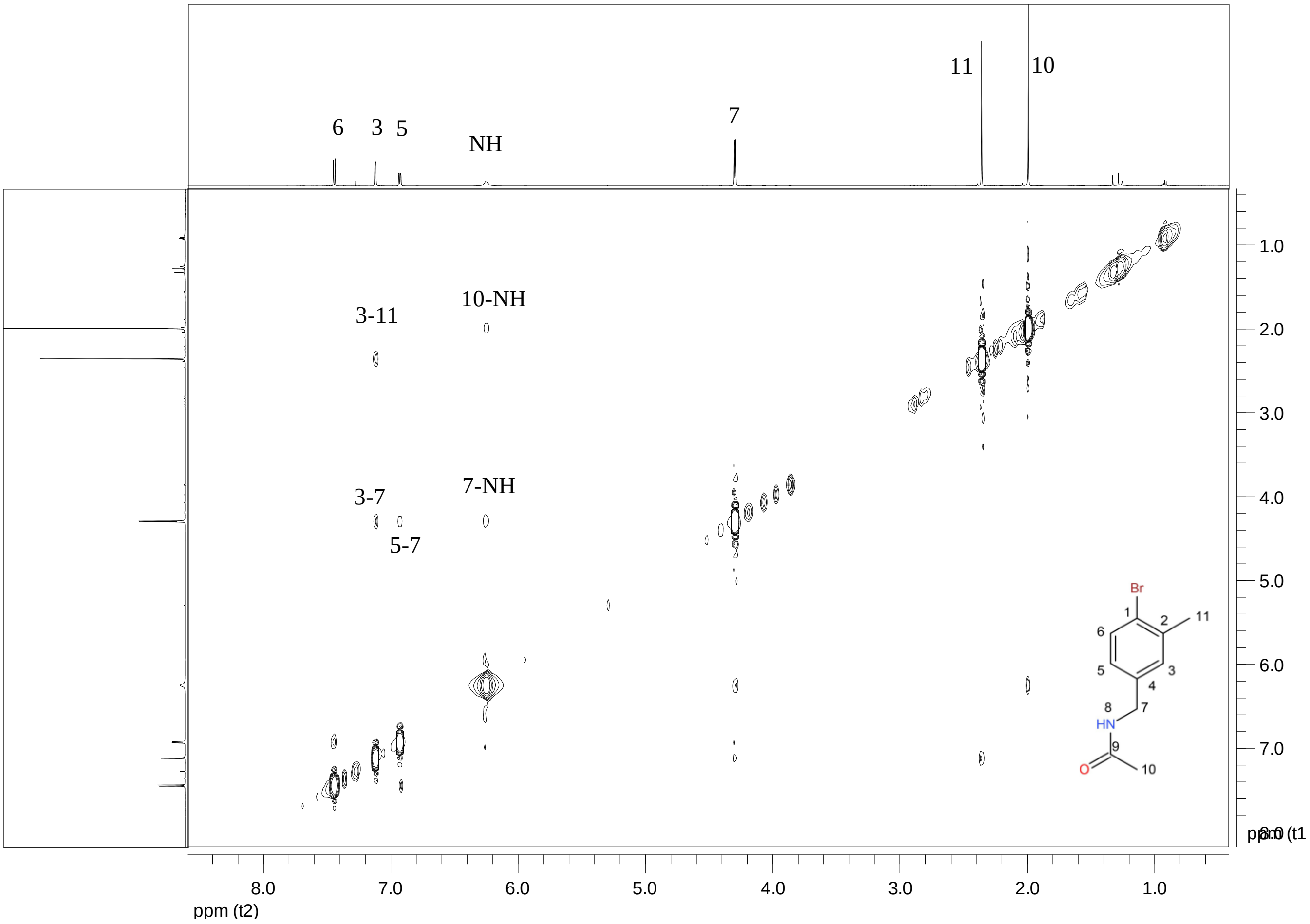

Supplementary Figure 43. ^{1}H NMR, ^{13}C NMR and 2D NOESY spectra of compound 36.

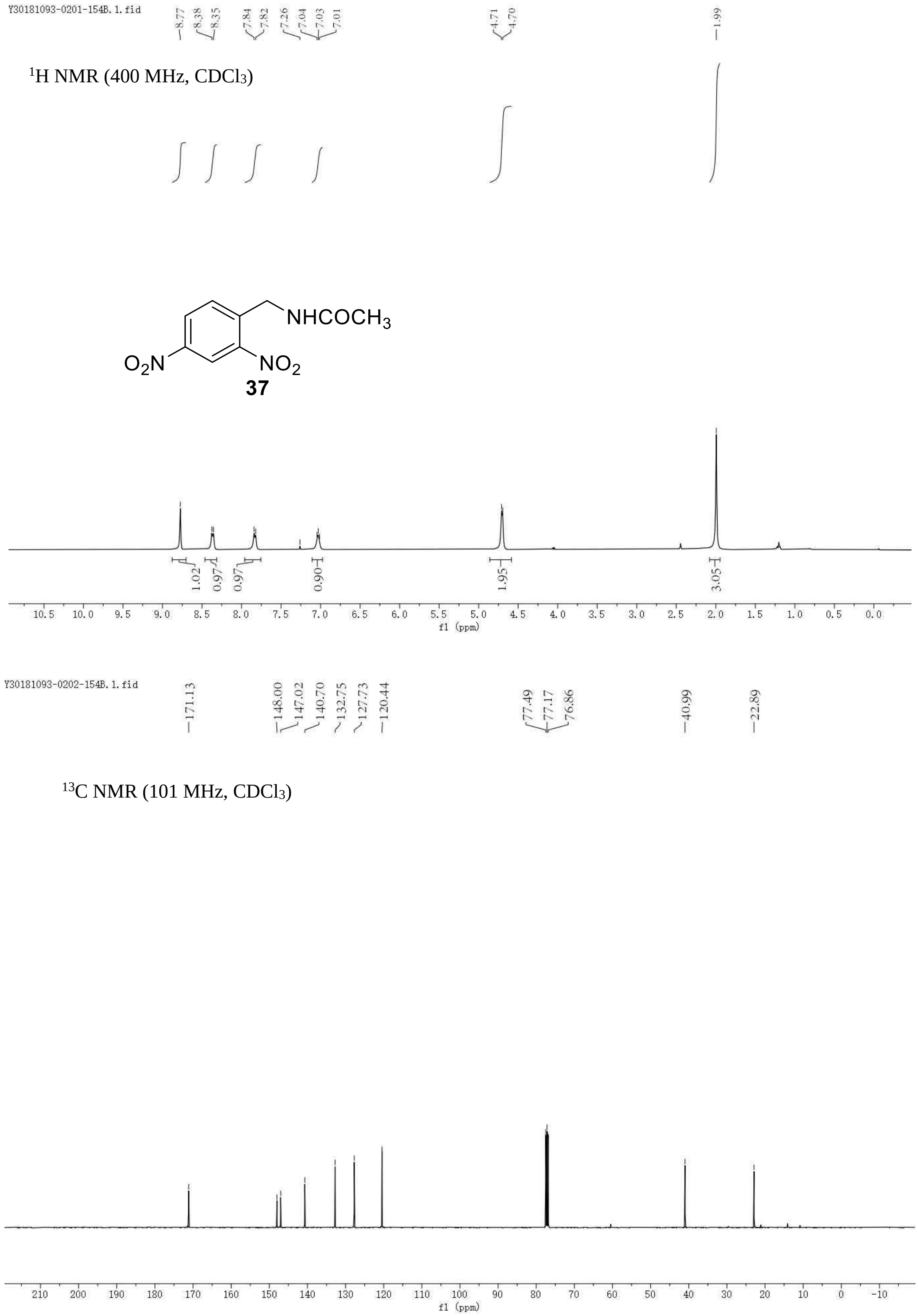

Supplementary Figure 44. ^{1}H NMR and ^{13}C NMR spectra of compound 37.

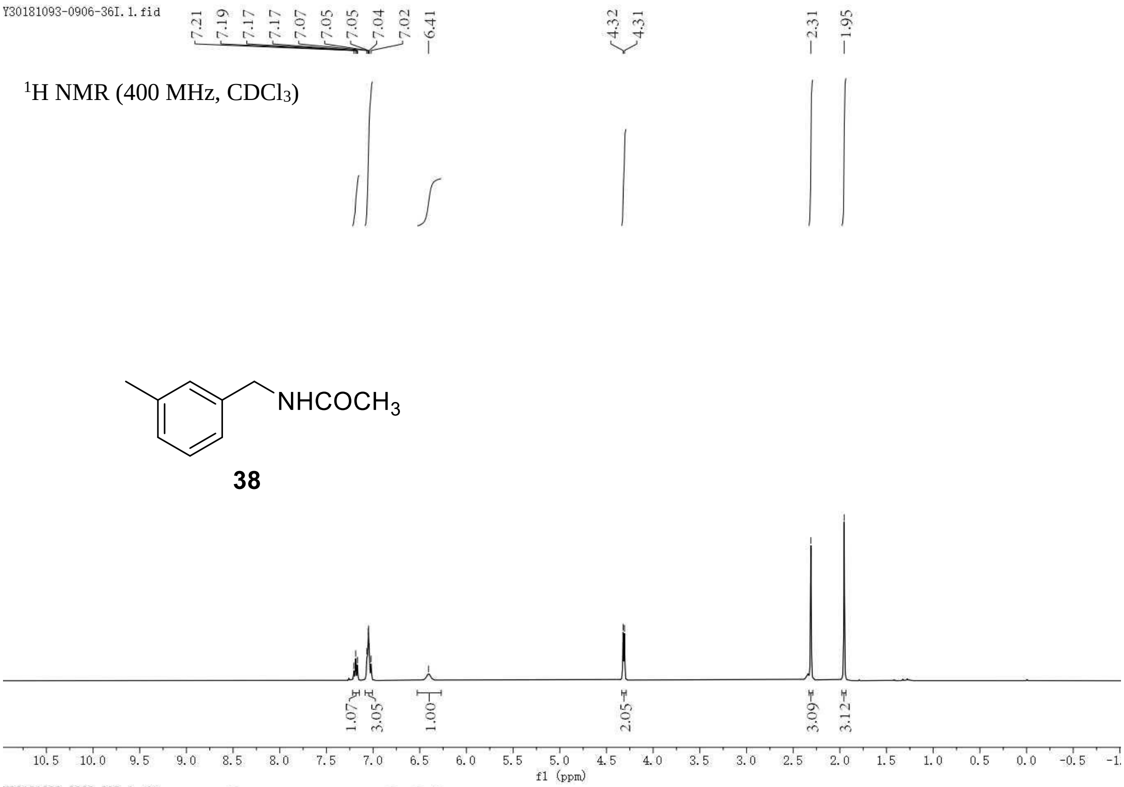

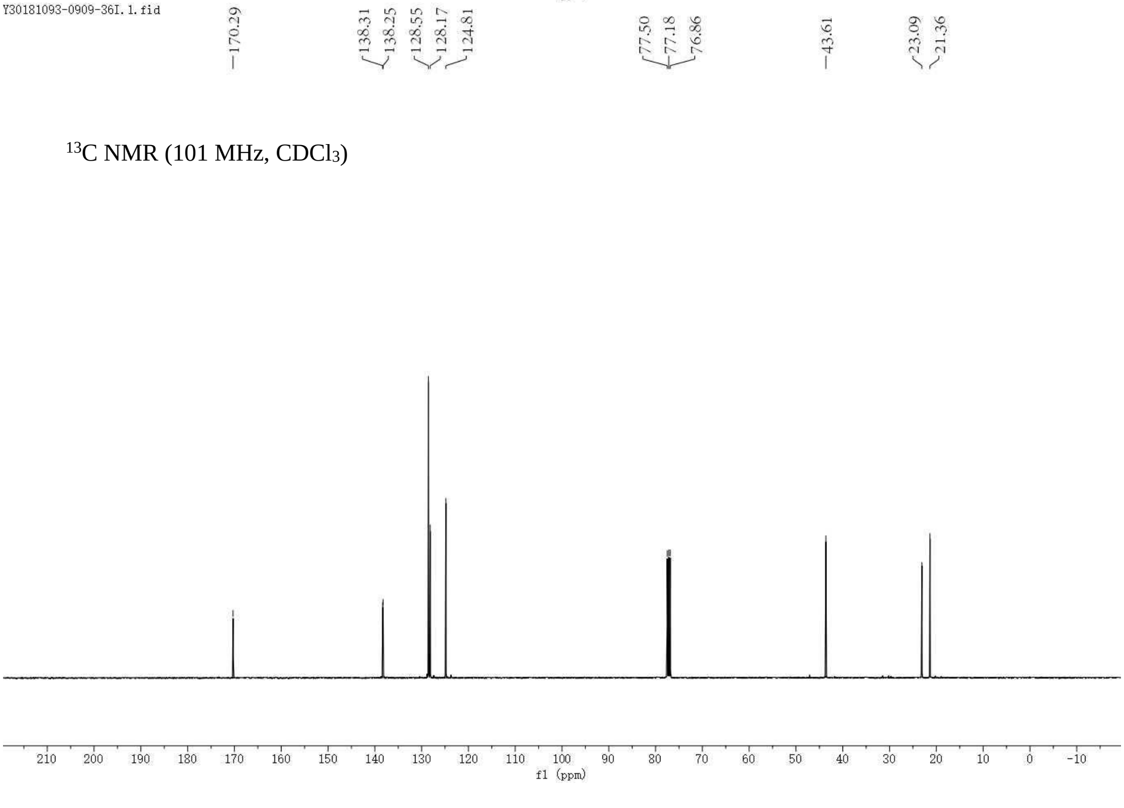

Supplementary Figure 45. ^{1}H NMR and ^{13}C NMR spectra of compound 38.

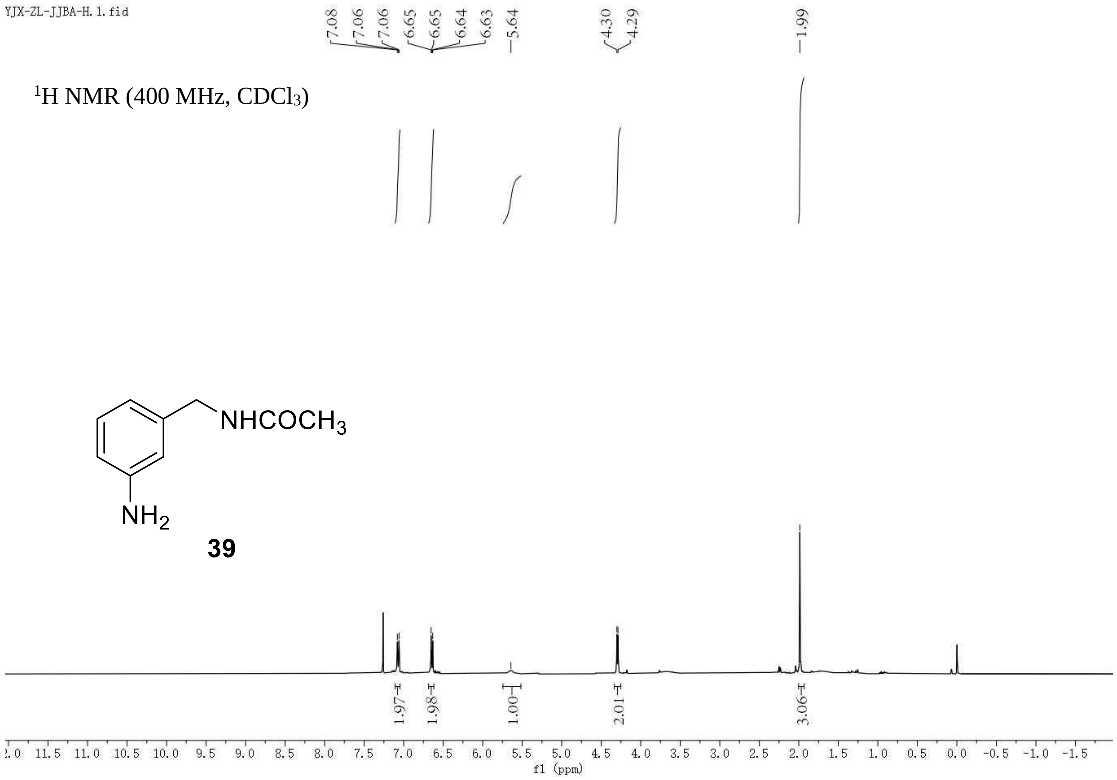

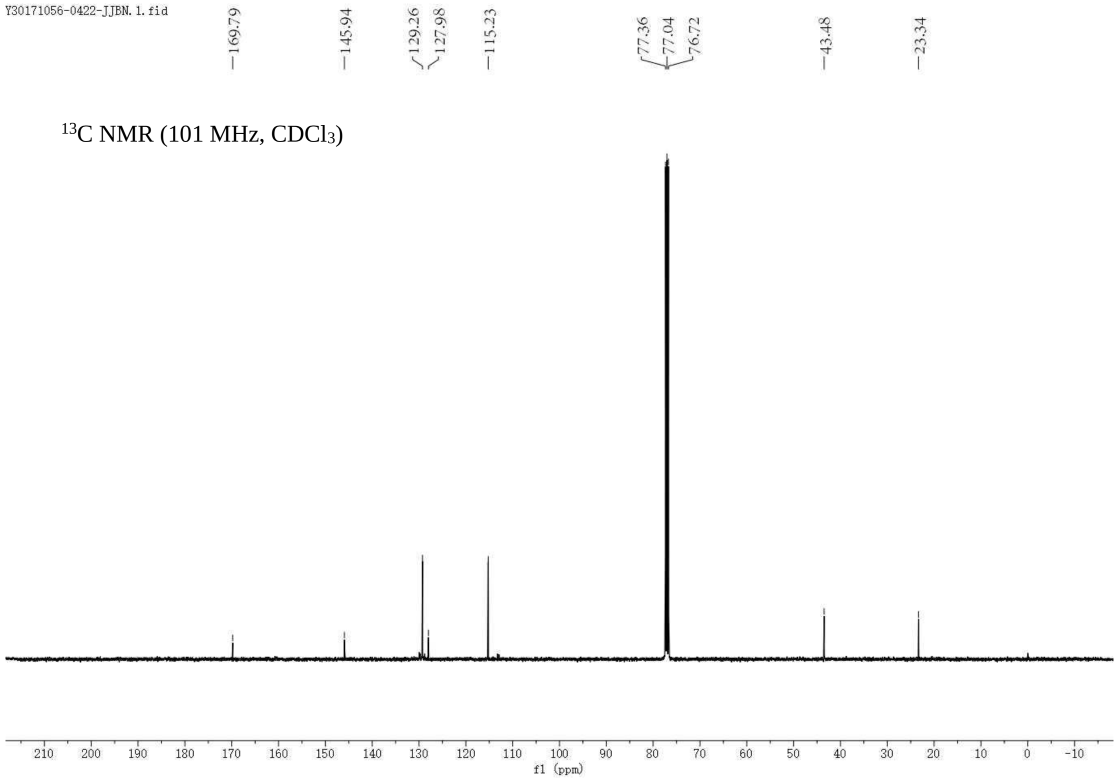

Supplementary Figure 46. ^{1}H NMR and ^{13}C NMR spectra of compound 39.

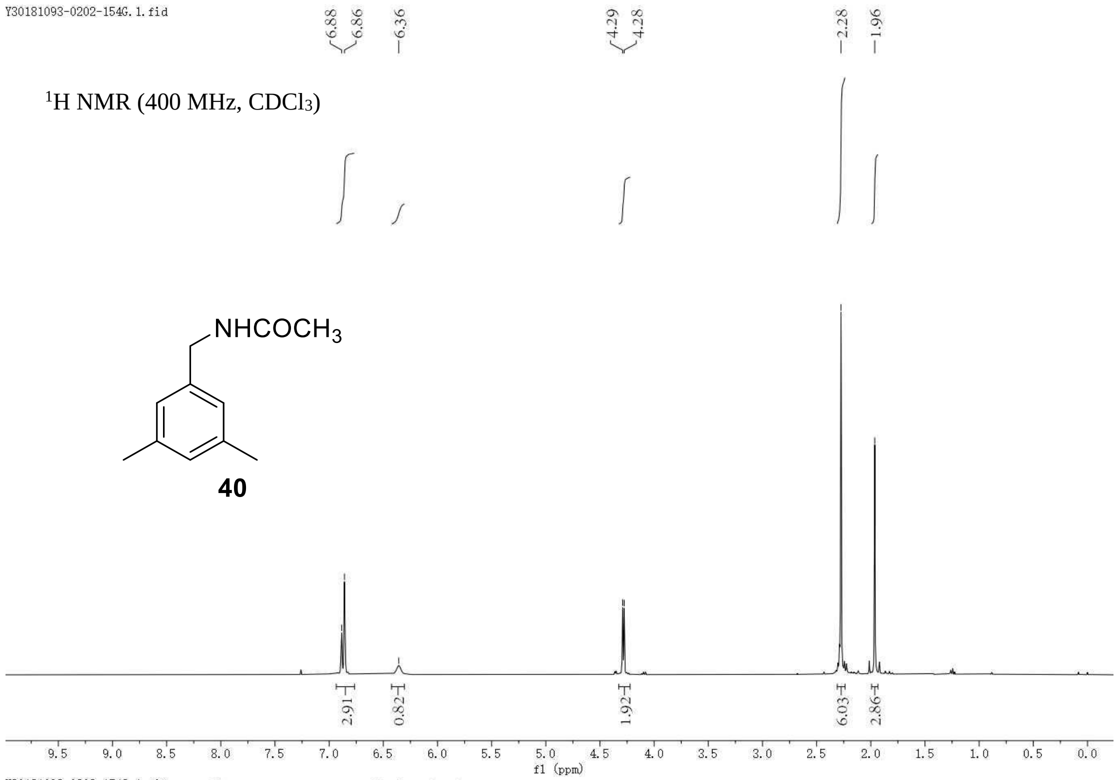

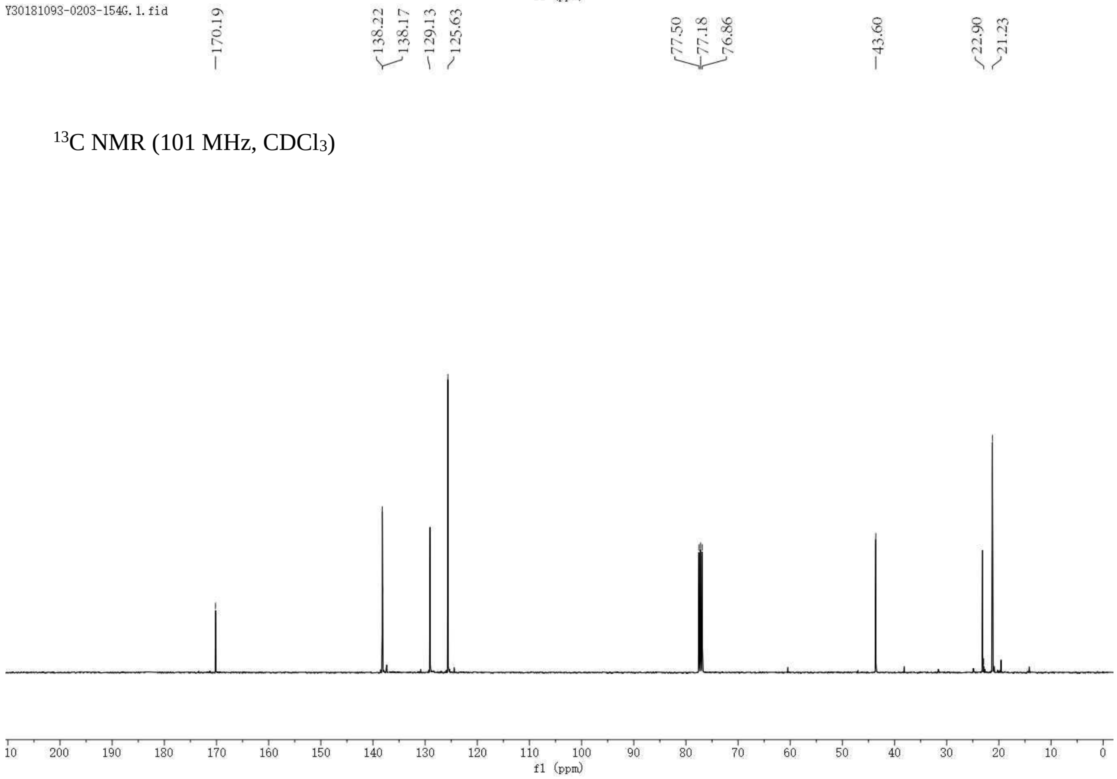

Supplementary Figure 47. ^{1}H NMR and ^{13}C NMR spectra of compound 40.

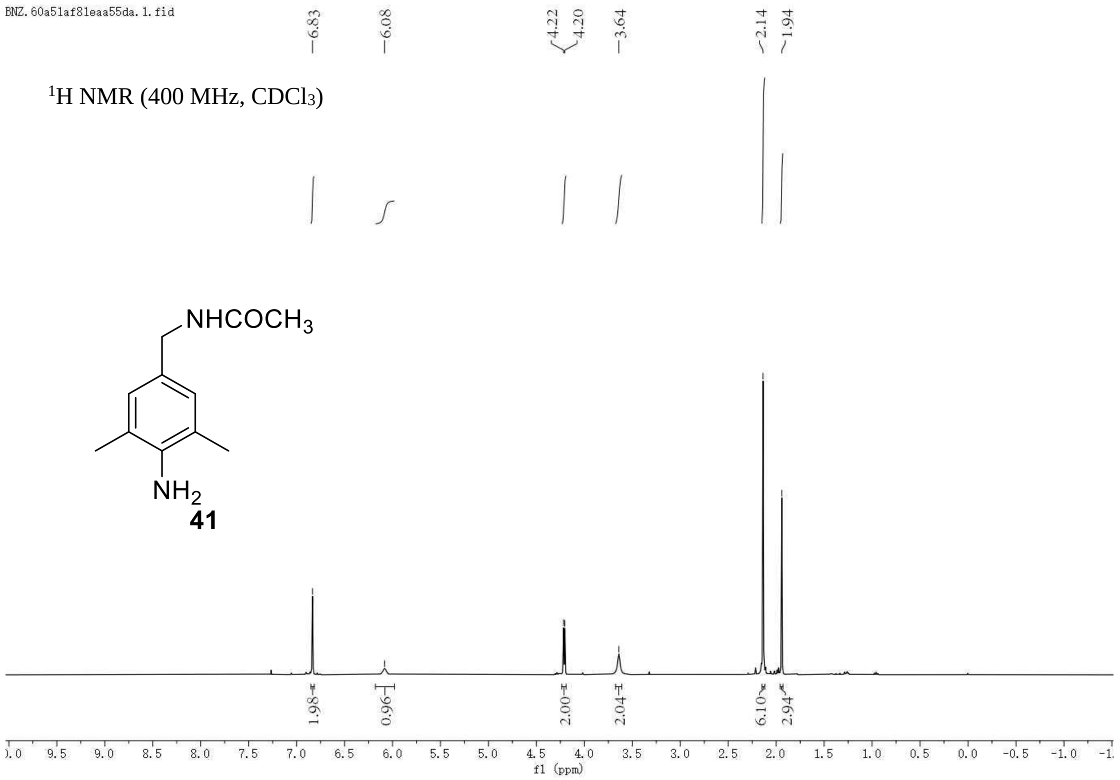

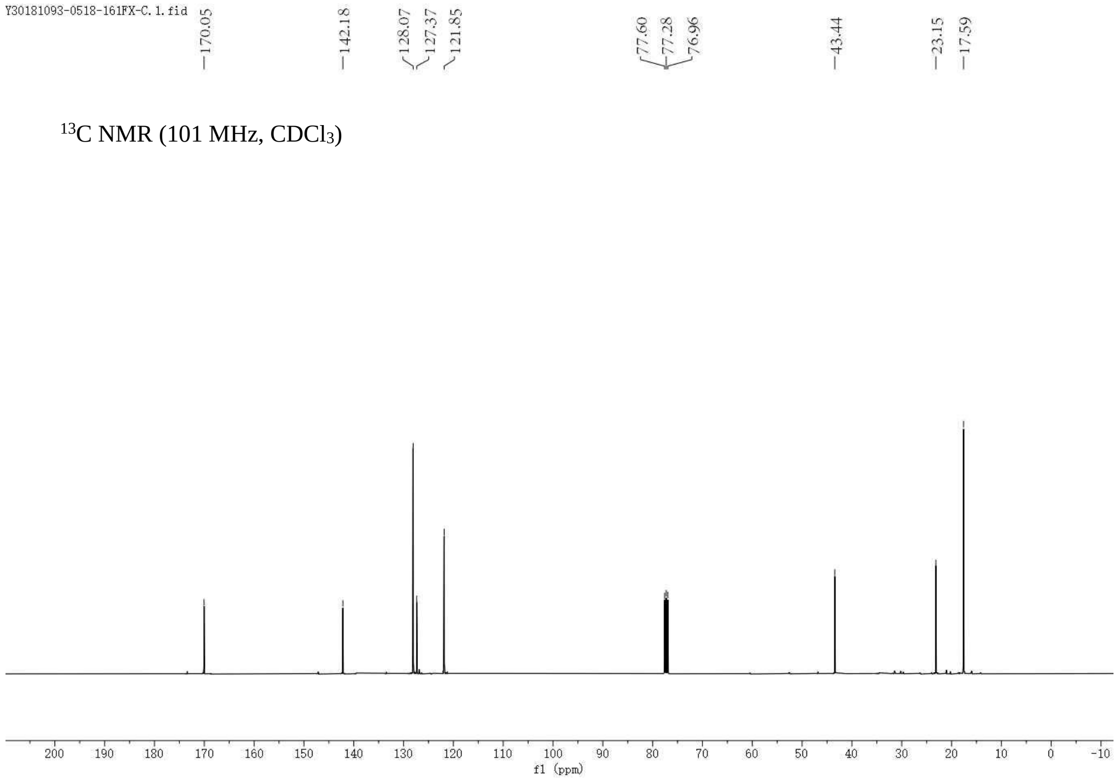

Supplementary Figure 48. ^{1}H NMR and ^{13}C NMR spectra of compound 41.

^{1}H NMR (400 MHz, $CDCl_3$)

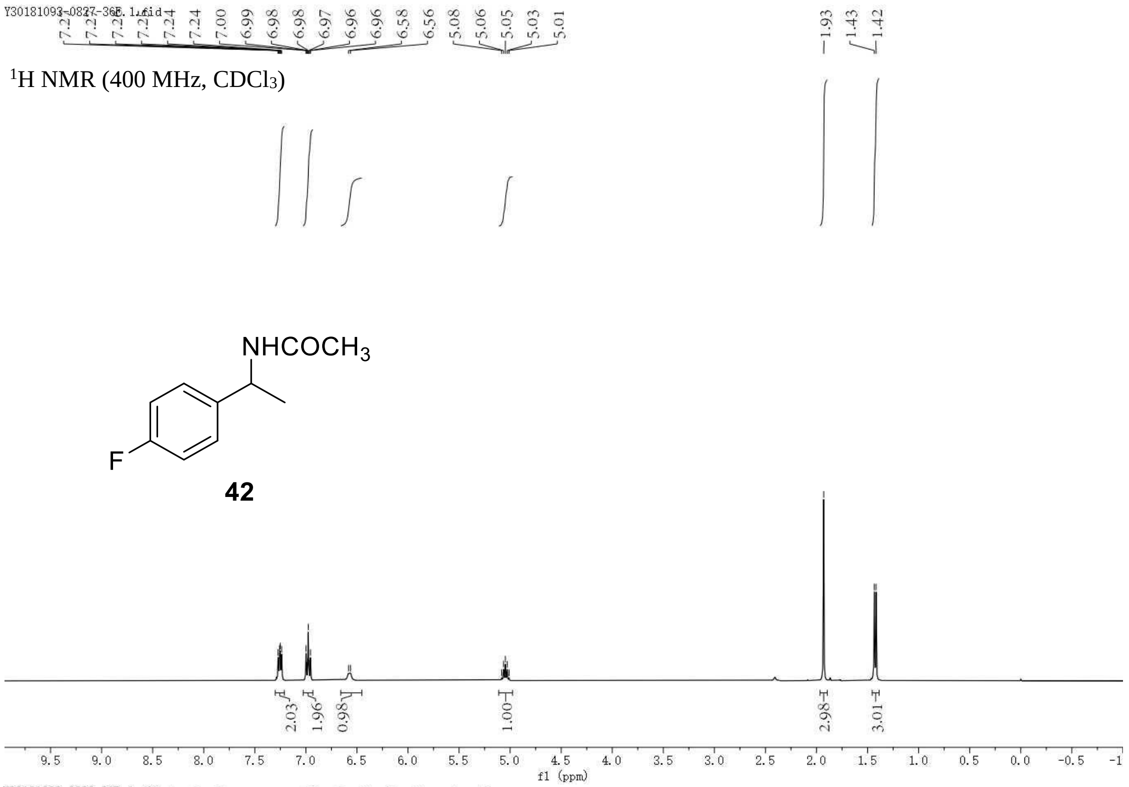

42

^{13}C NMR (101 MHz, $CDCl_3$)

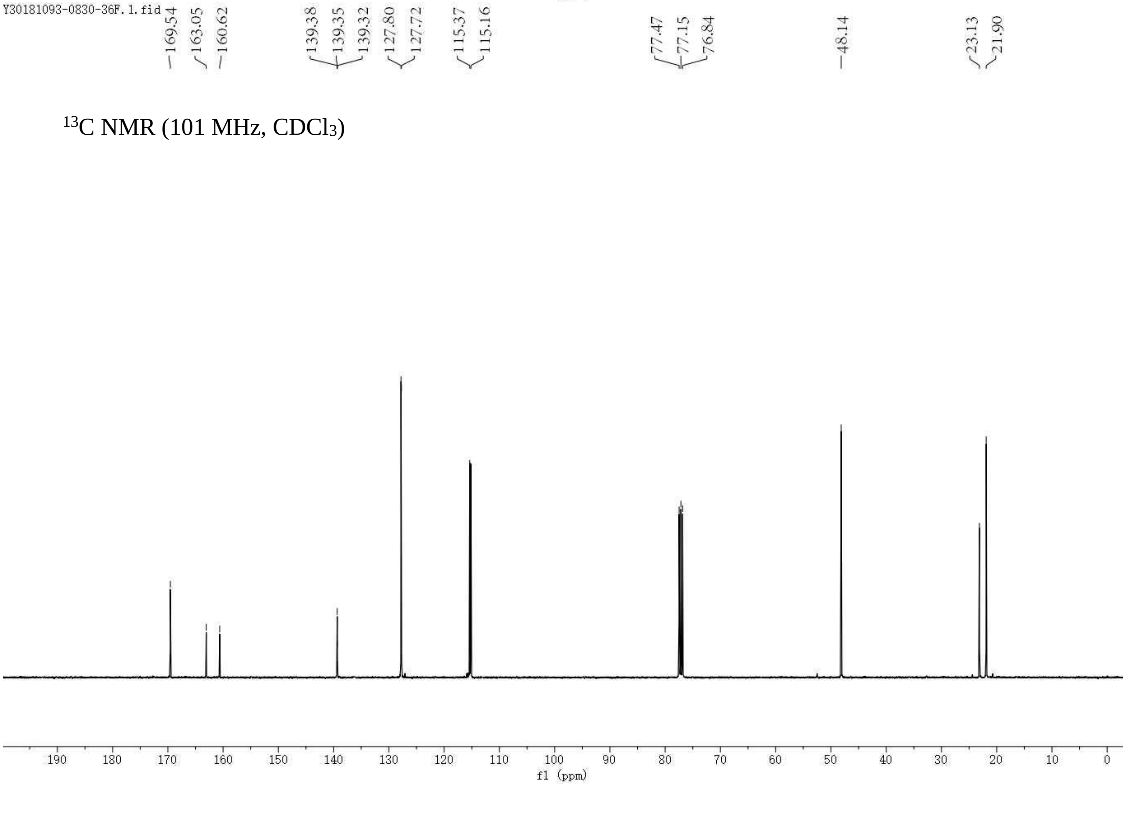

Y30181093-0114-FP.1.fid

^{19}F NMR (101 MHz, $CDCl_3$)

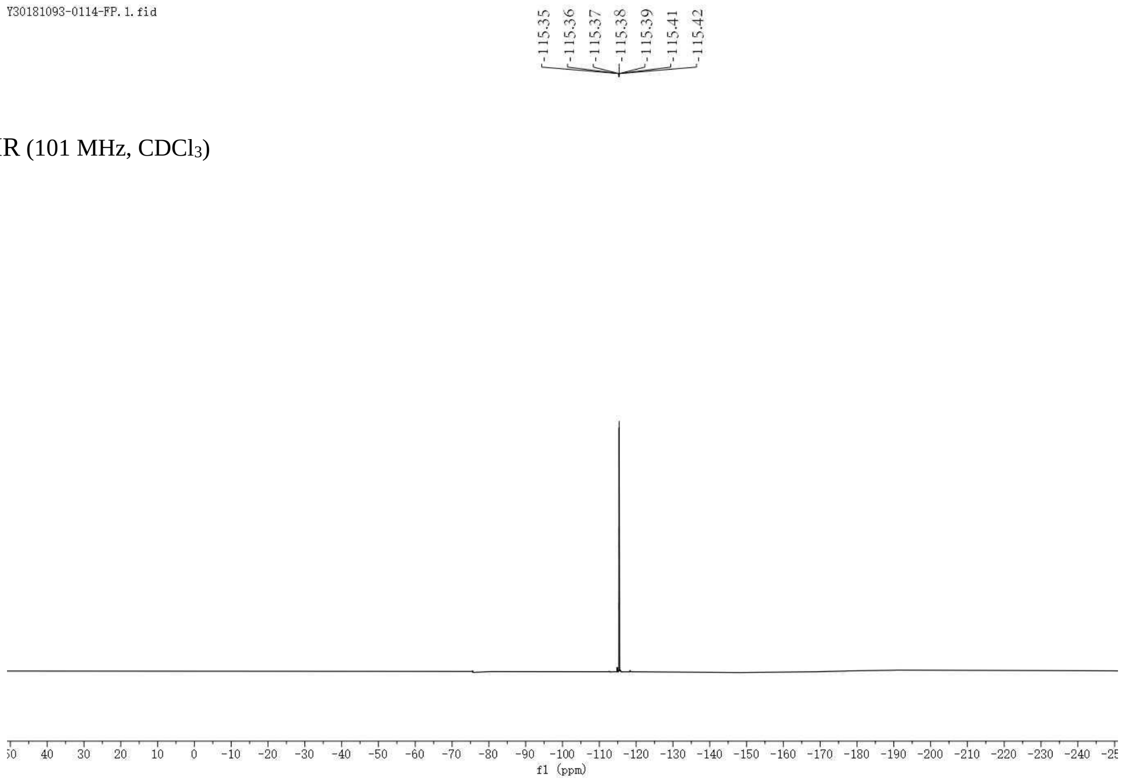

Supplementary Figure 49. ^{1}H NMR, ^{13}C NMR and ^{19}F NMR spectra of compound 42.

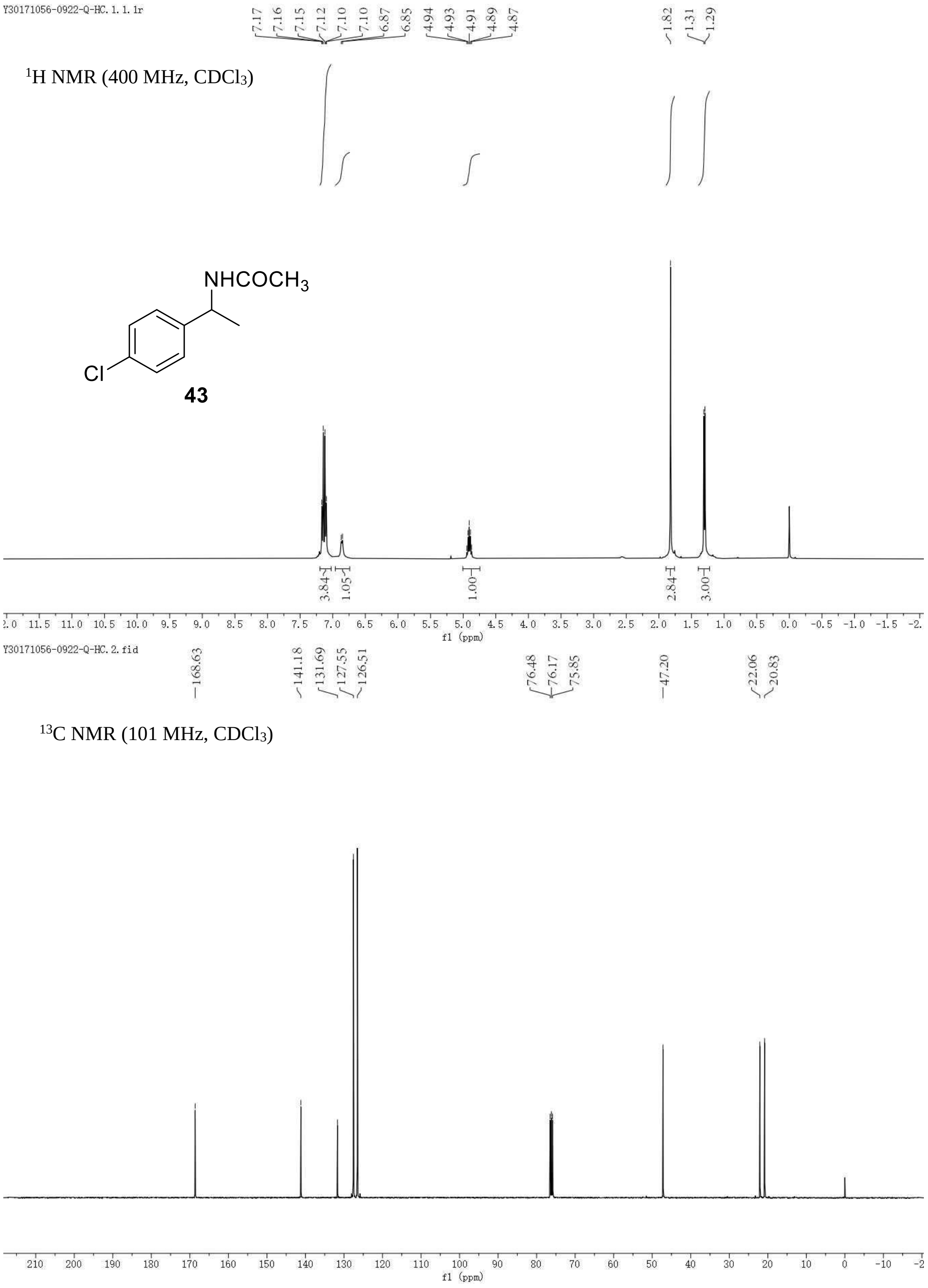

Supplementary Figure 50. ^{1}H NMR and ^{13}C NMR spectra of compound 43.

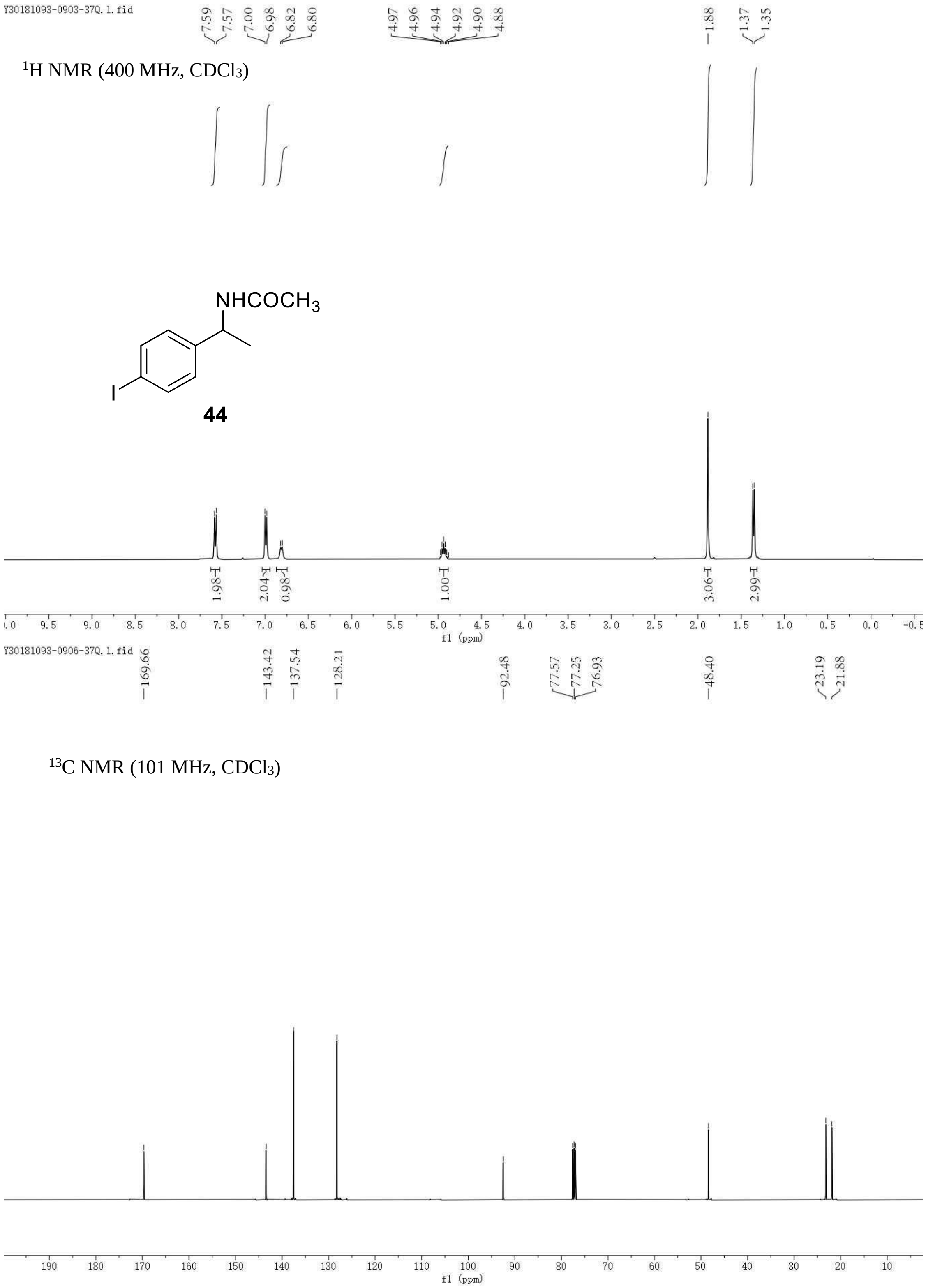

Supplementary Figure 51. ^{1}H NMR and ^{13}C NMR spectra of compound 44.

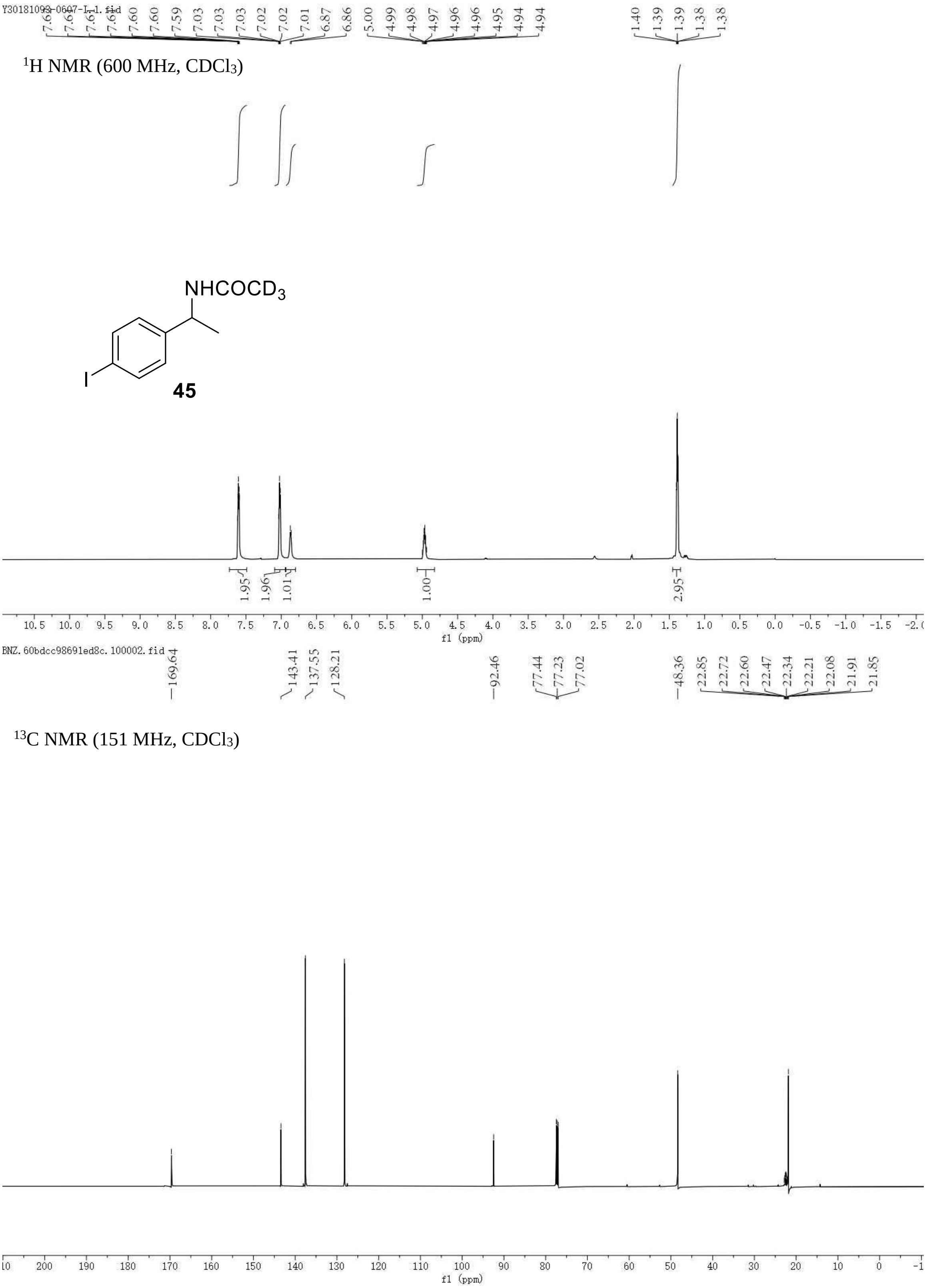

Supplementary Figure 52. ^{1}H NMR and ^{13}C NMR spectra of compound 45.

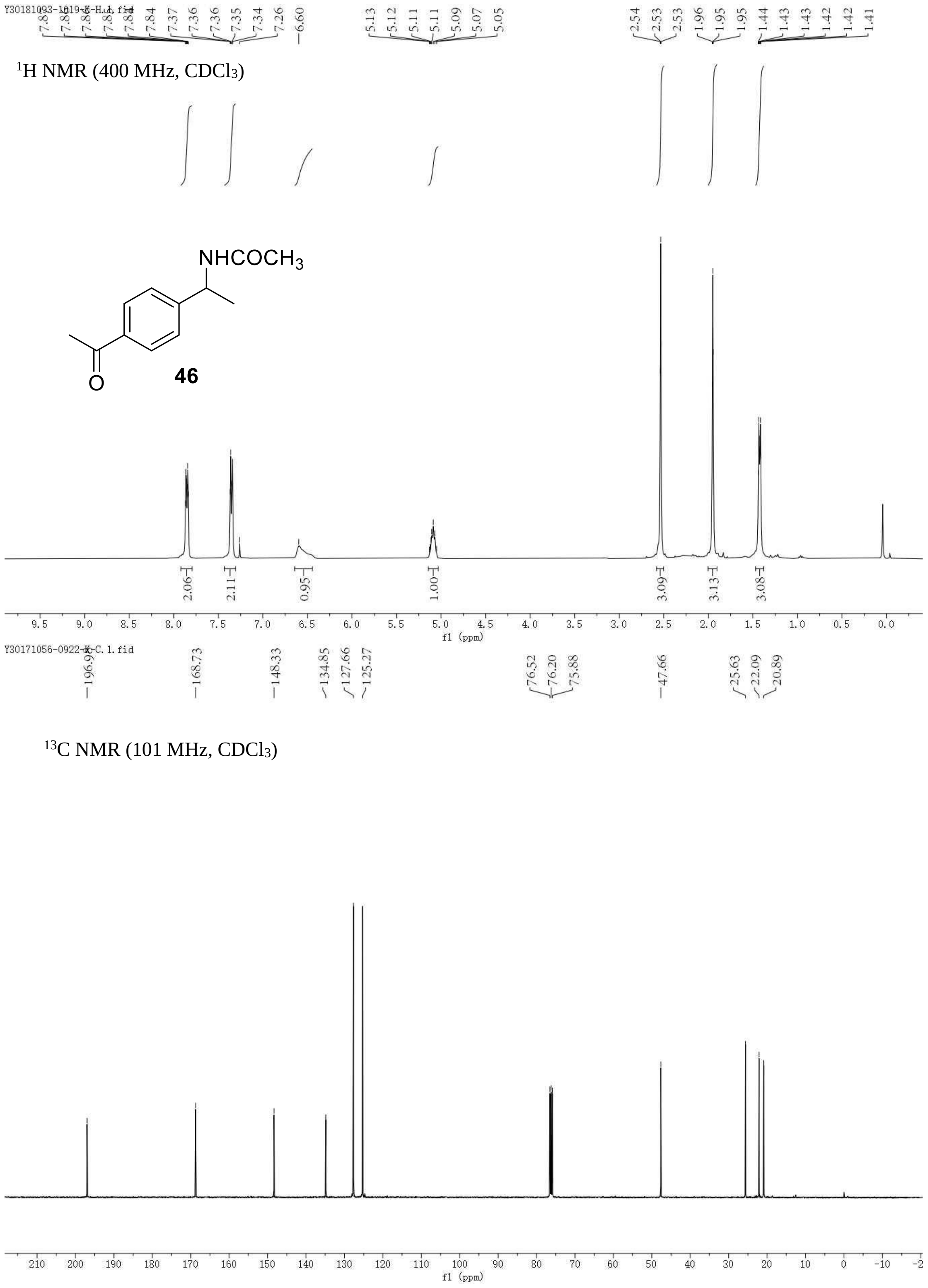

Supplementary Figure 53. ^{1}H NMR and ^{13}C NMR spectra of compound 46.

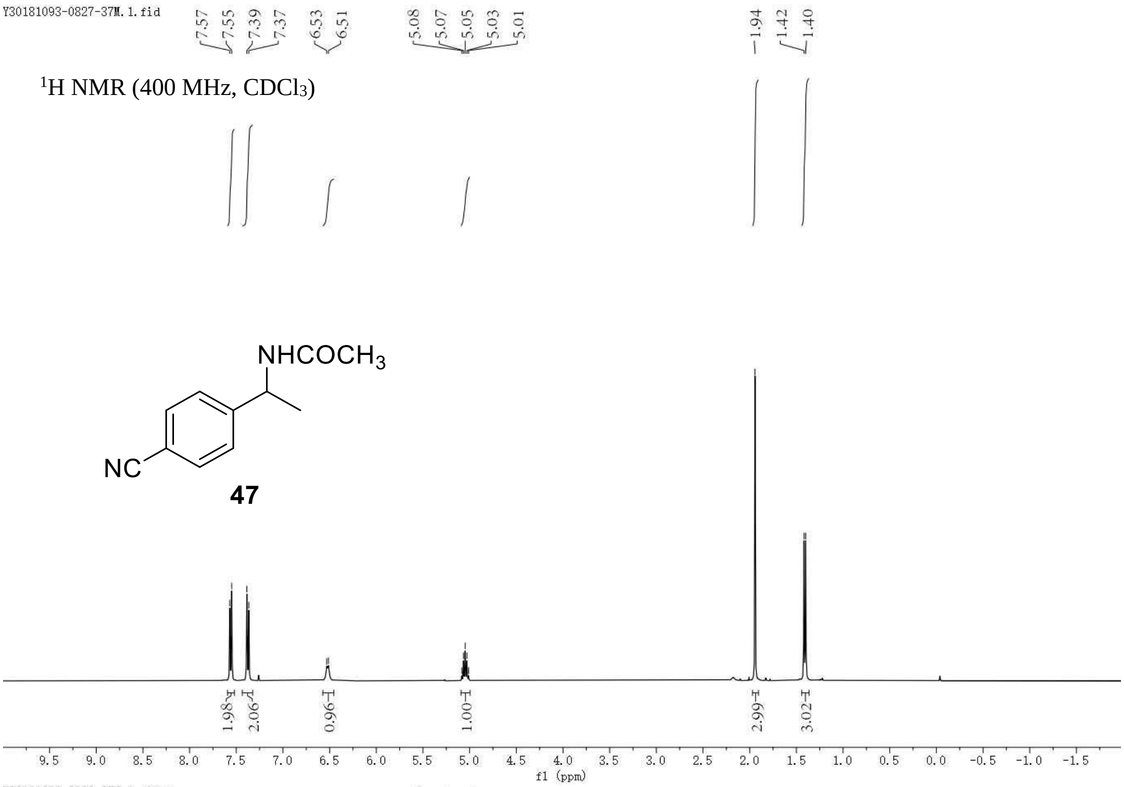

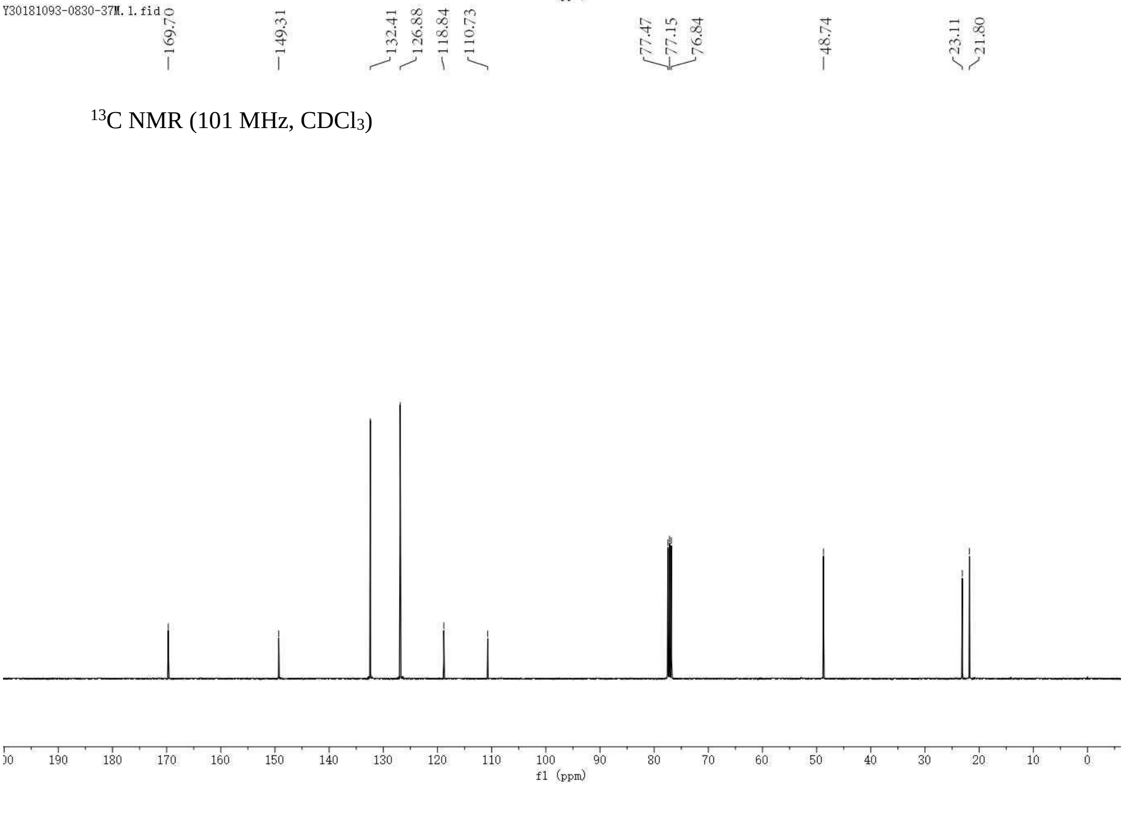

Supplementary Figure 54. ^{1}H NMR and ^{13}C NMR spectra of compound 47.

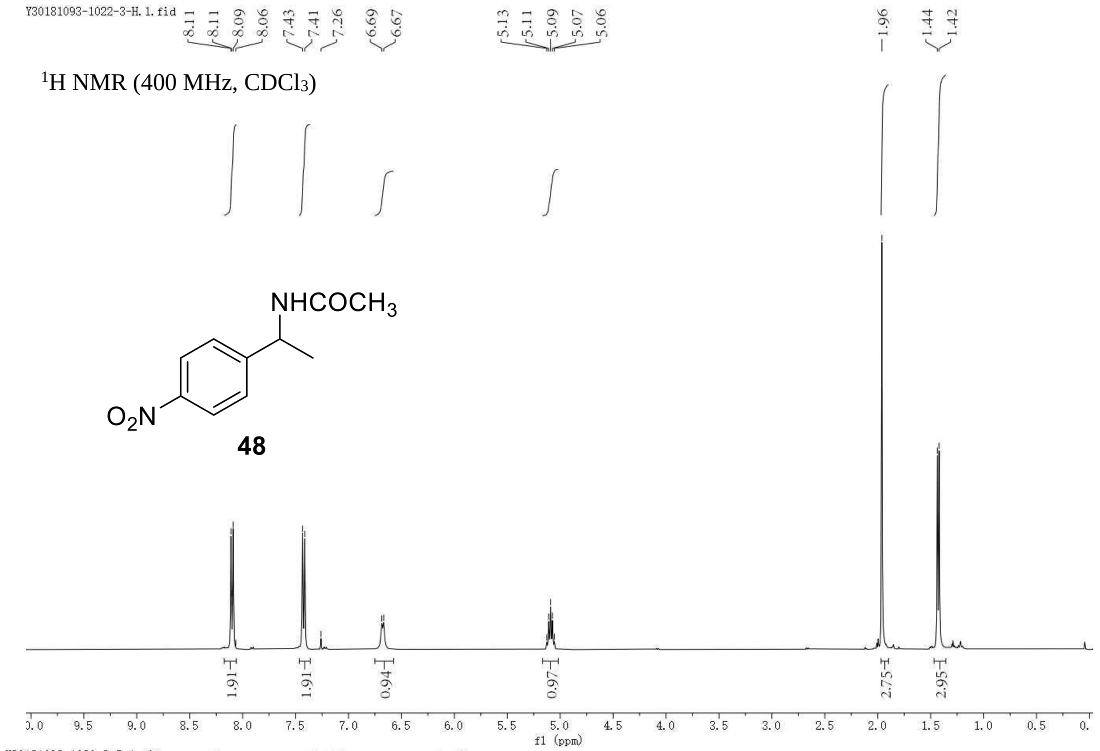

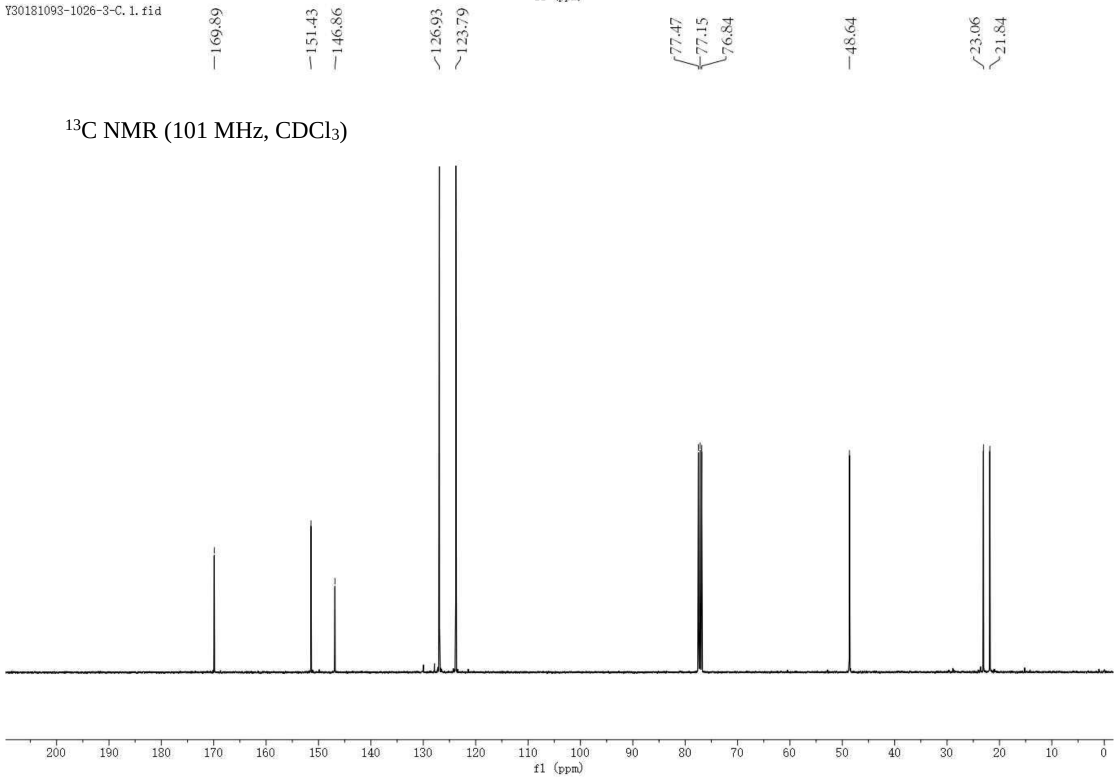

Supplementary Figure 55. ^{1}H NMR and ^{13}C NMR spectra of compound 48.

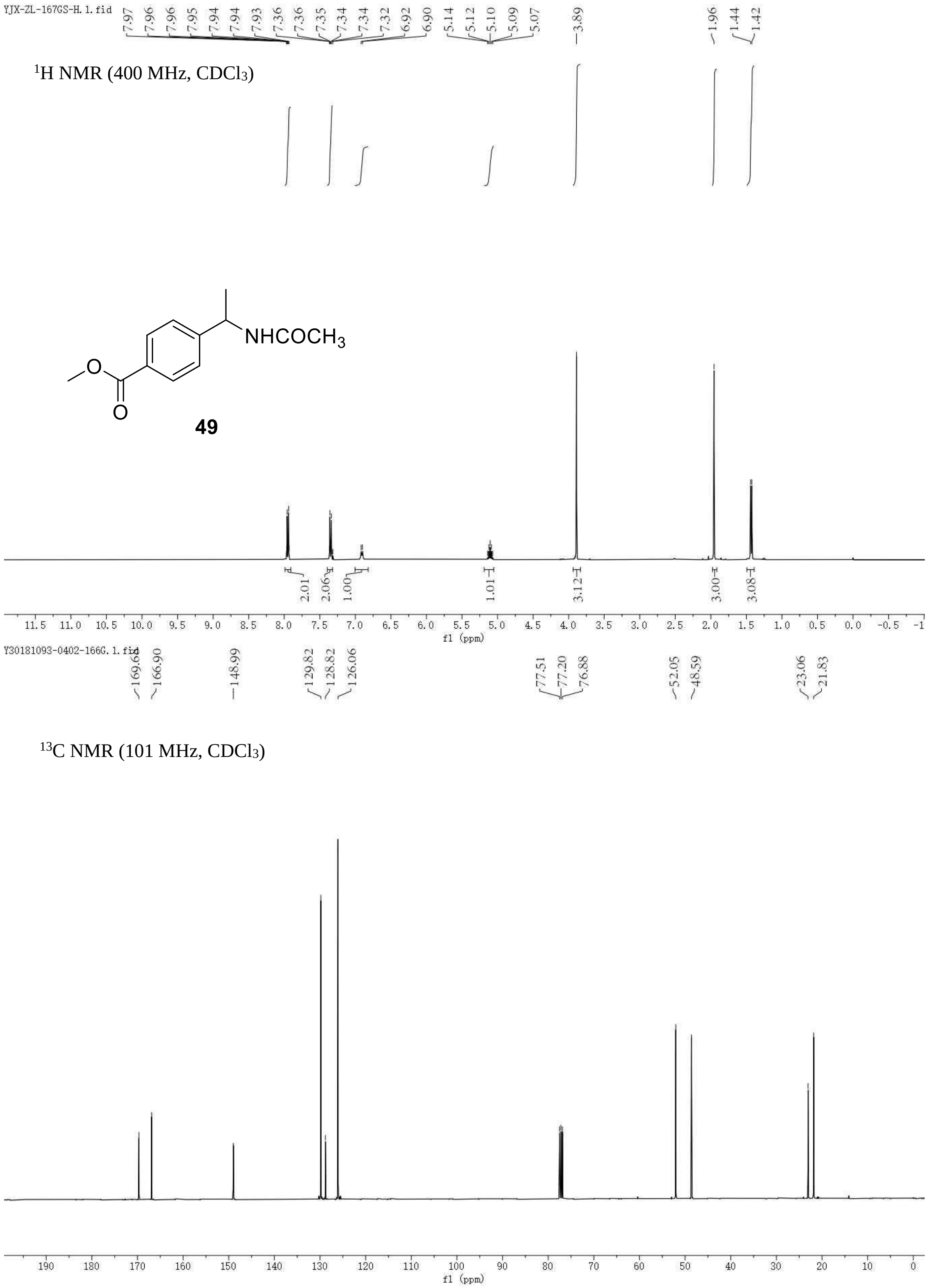

Supplementary Figure 56. ^{1}H NMR and ^{13}C NMR spectra of compound 49.

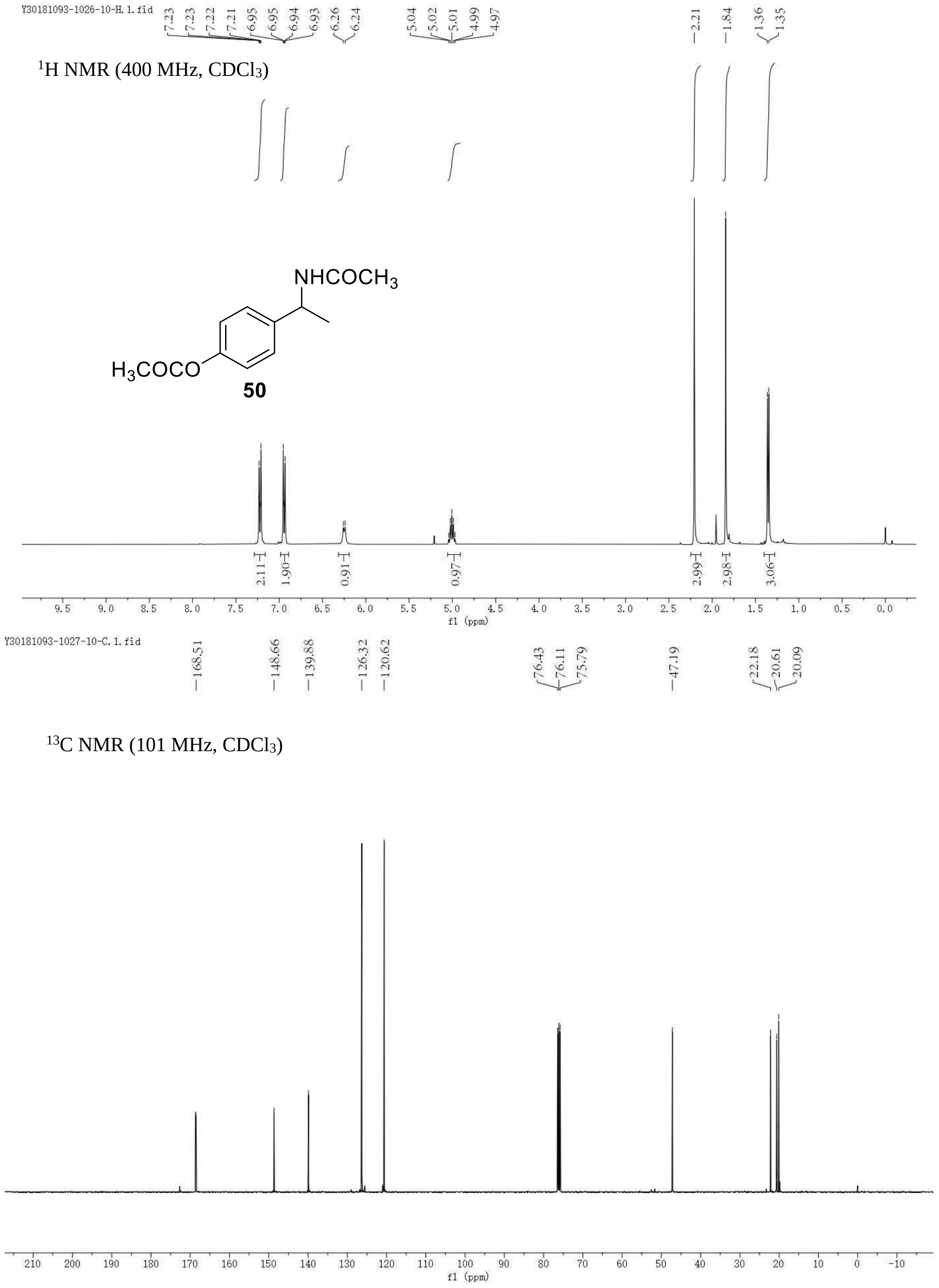

Supplementary Figure 57. ^{1}H NMR and ^{13}C NMR spectra of compound 50.

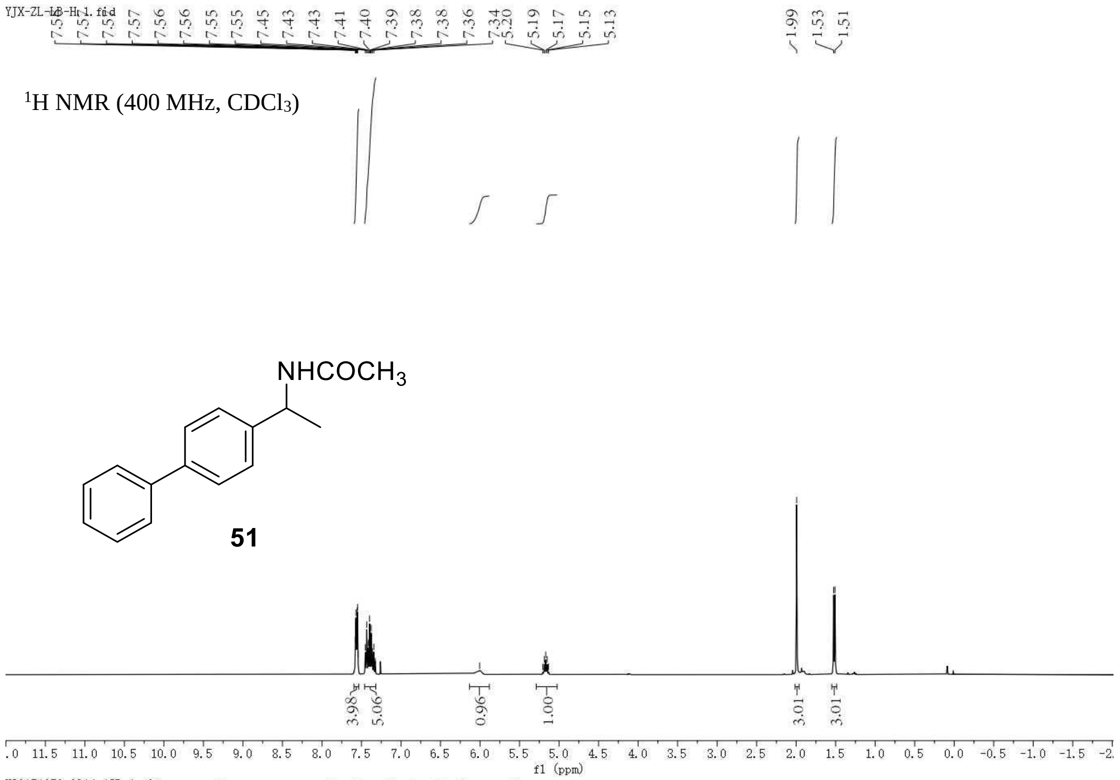

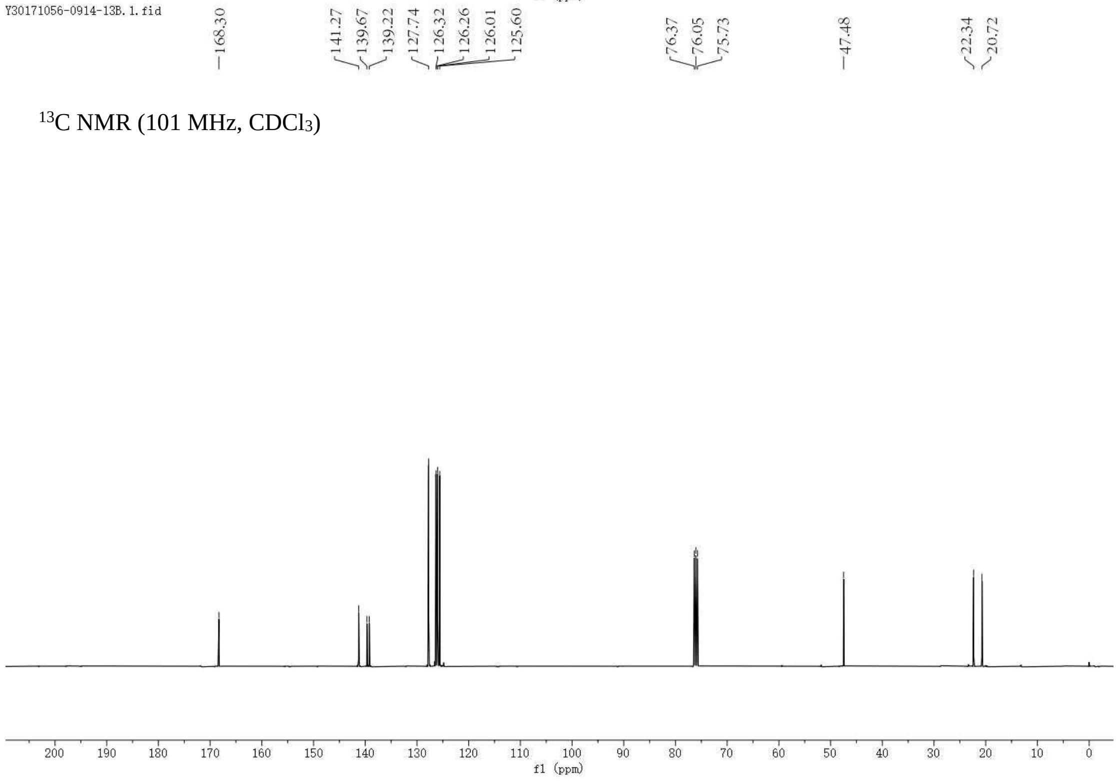

Supplementary Figure 58. ^{1}H NMR and ^{13}C NMR spectra of compound 51.

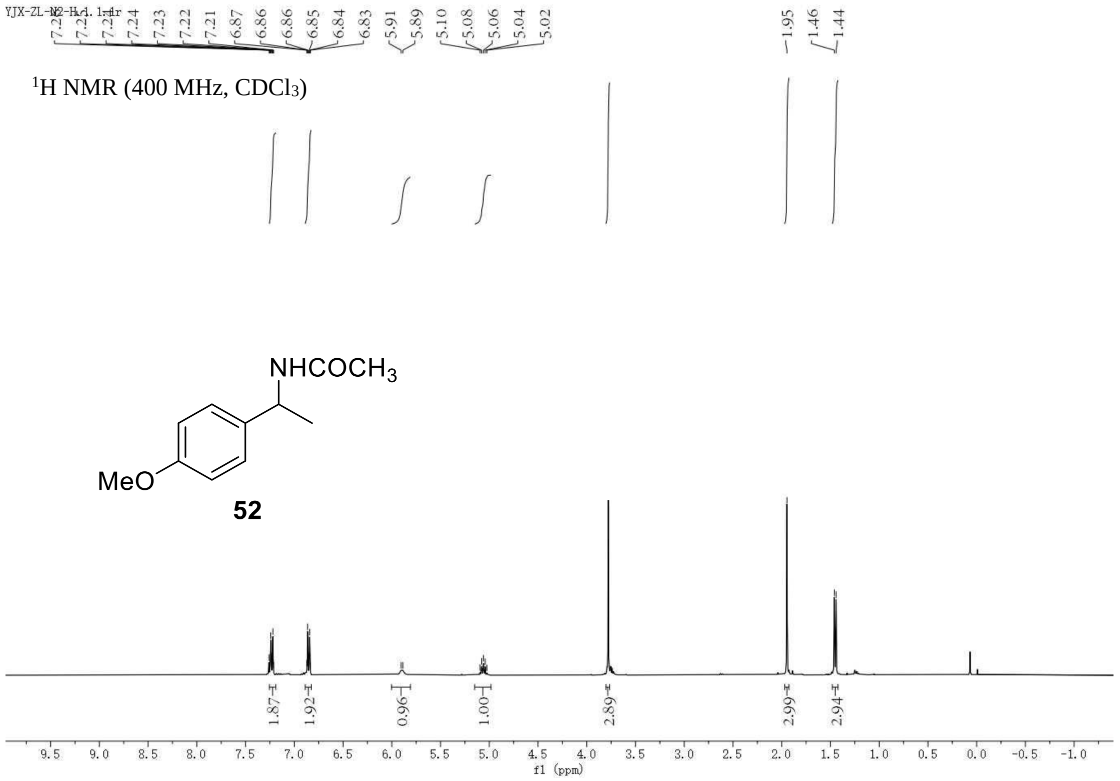

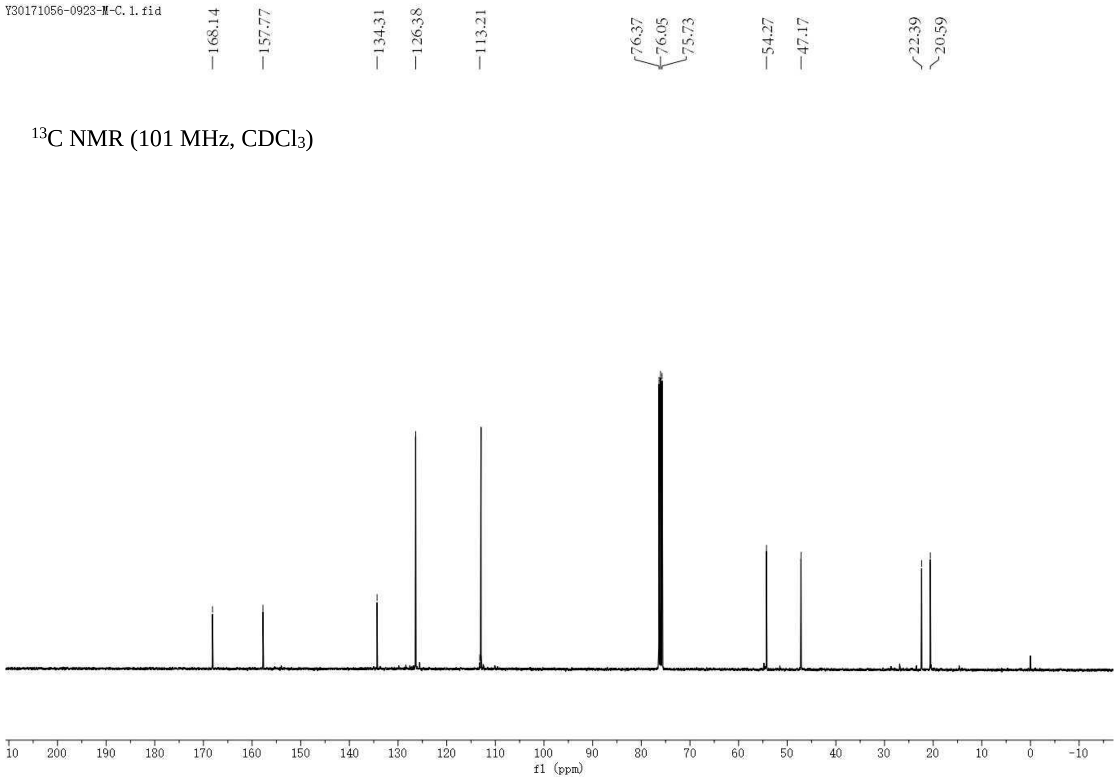

Supplementary Figure 59. ^{1}H NMR and ^{13}C NMR spectra of compound 52.

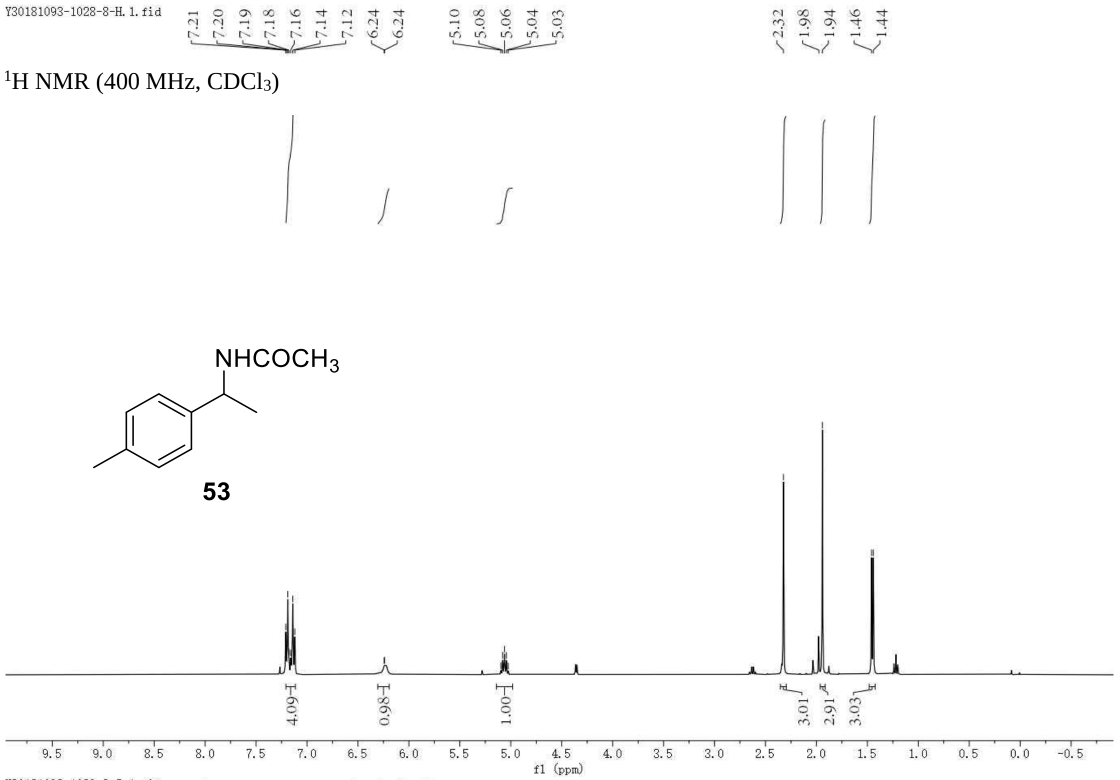

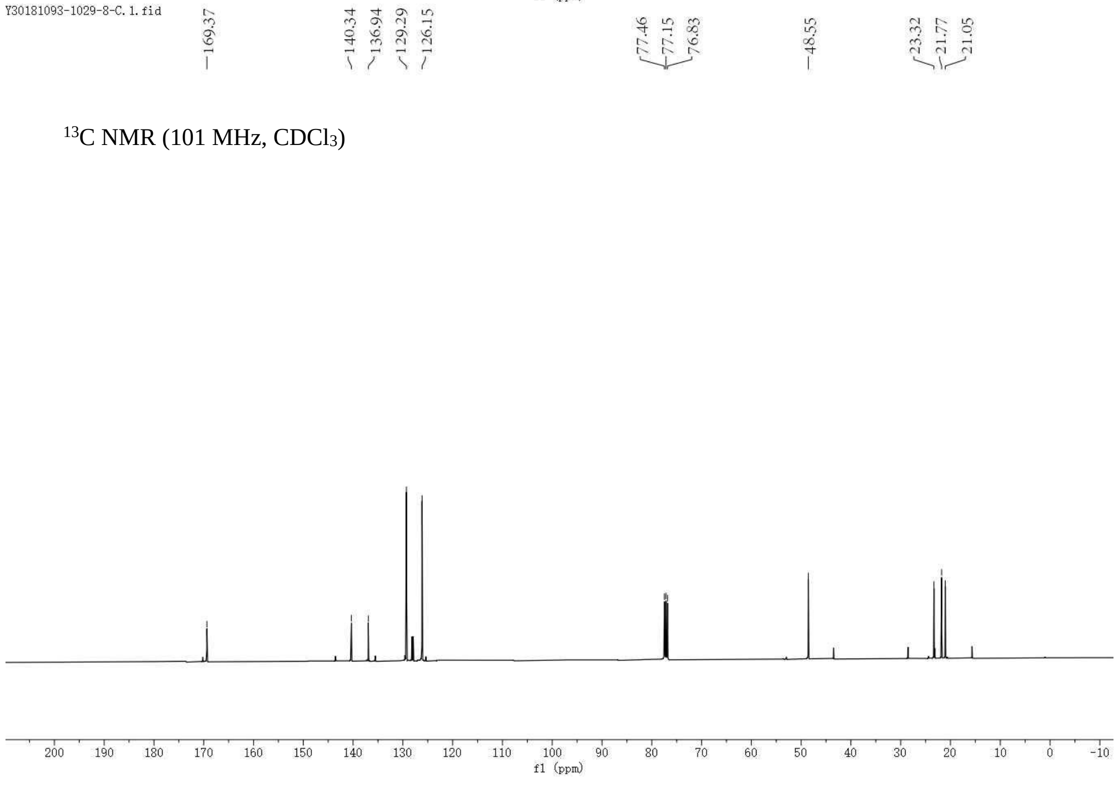

Supplementary Figure 60. ^{1}H NMR and ^{13}C NMR spectra of compound 53.

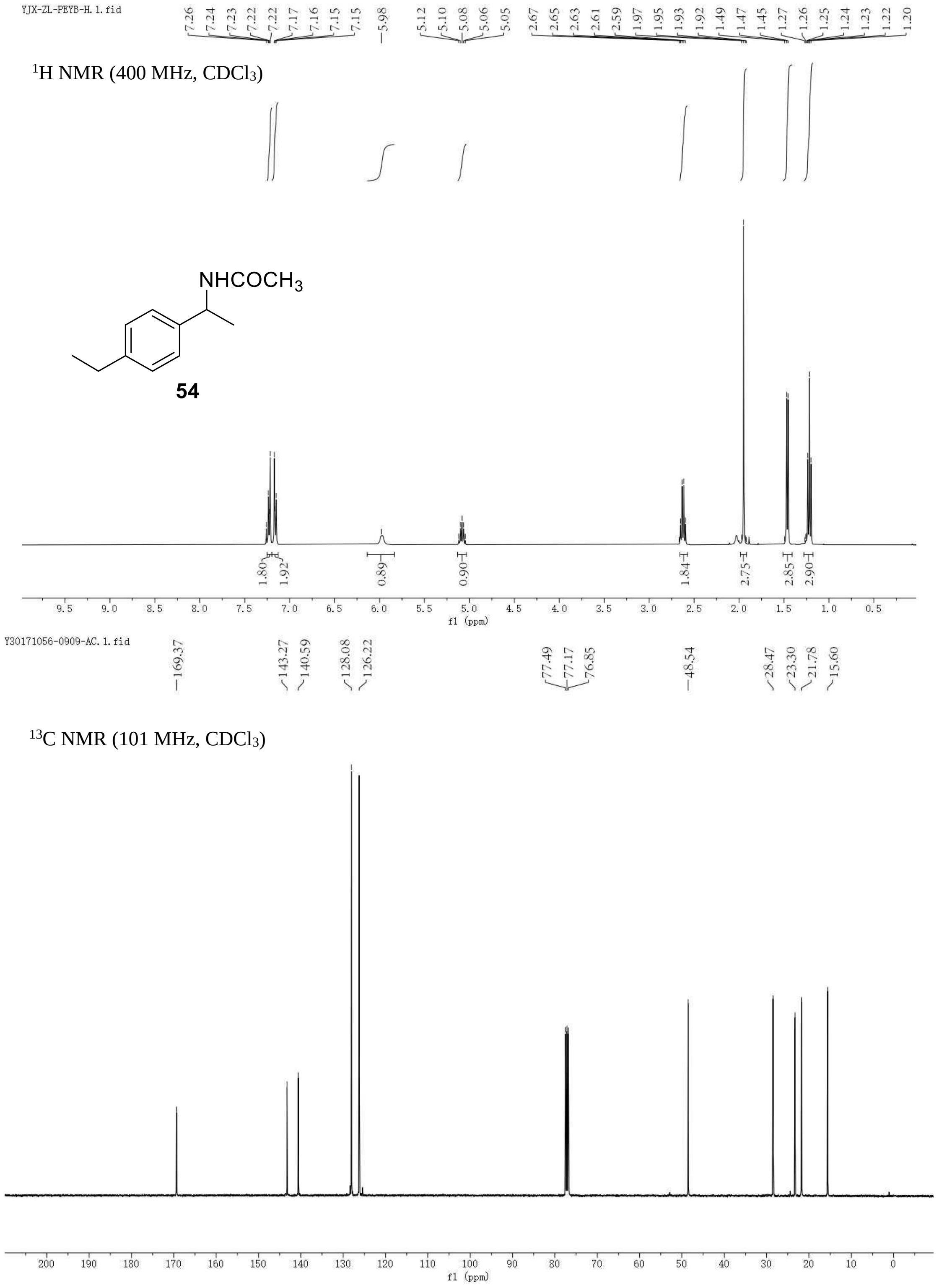

Supplementary Figure 61. ^{1}H NMR and ^{13}C NMR spectra of compound 54.

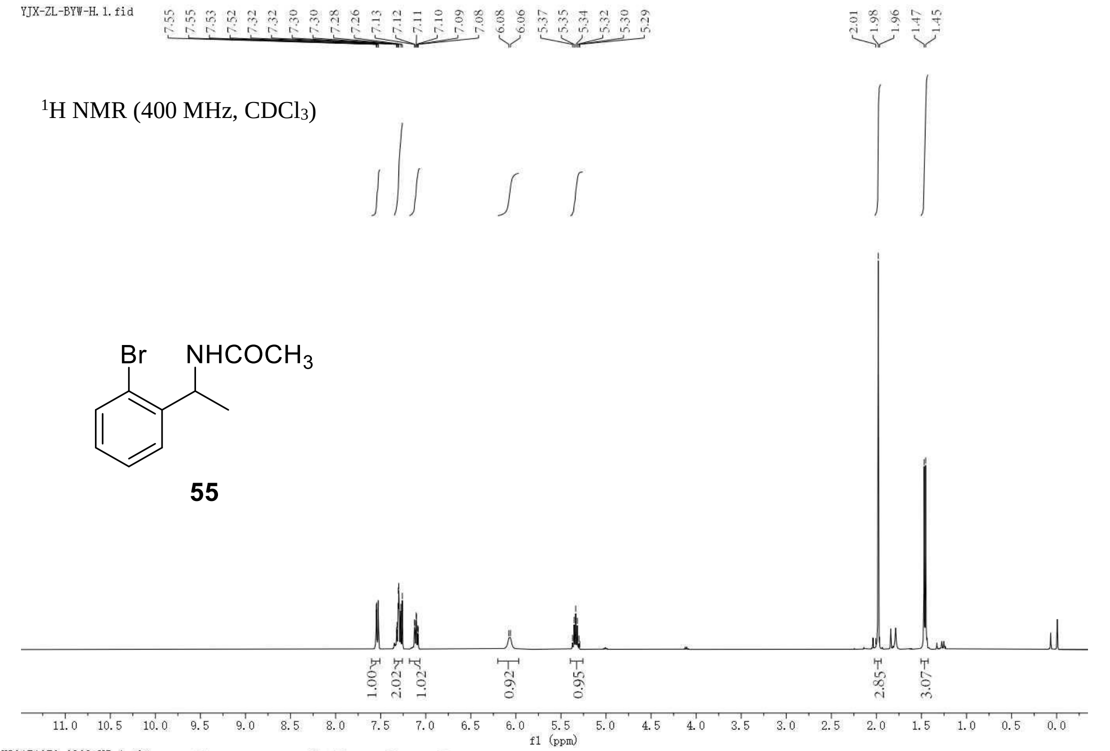

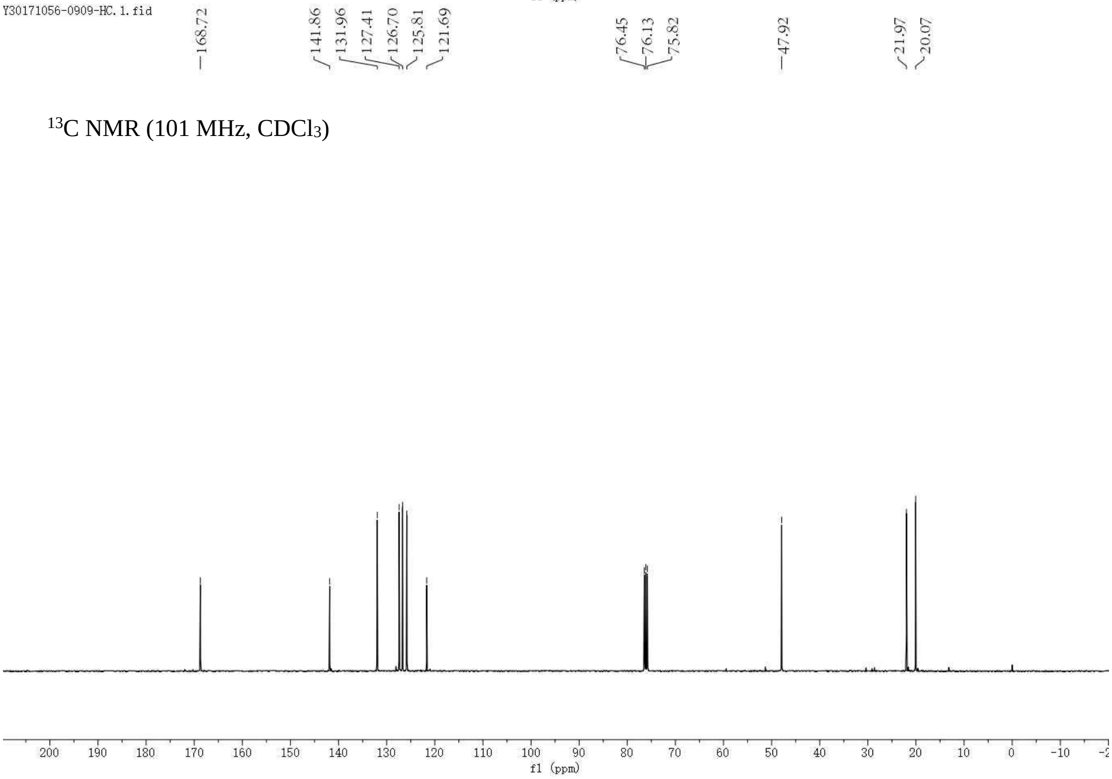

Supplementary Figure 62. ^{1}H NMR and ^{13}C NMR spectra of compound 55.

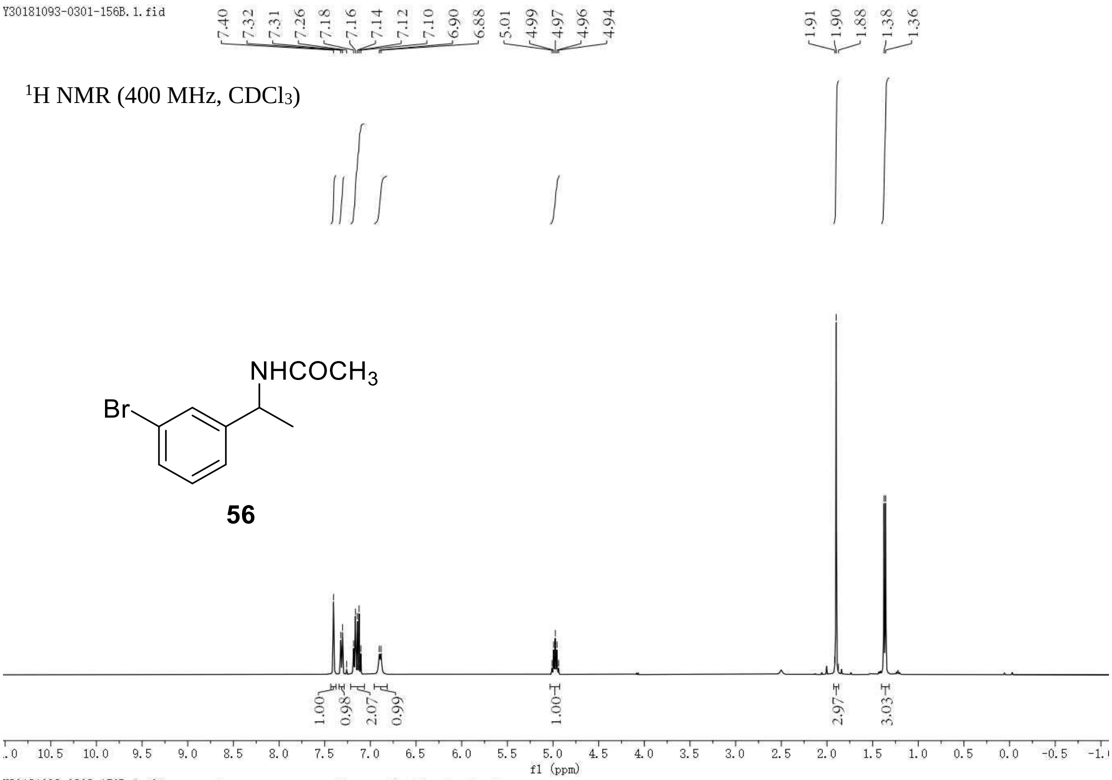

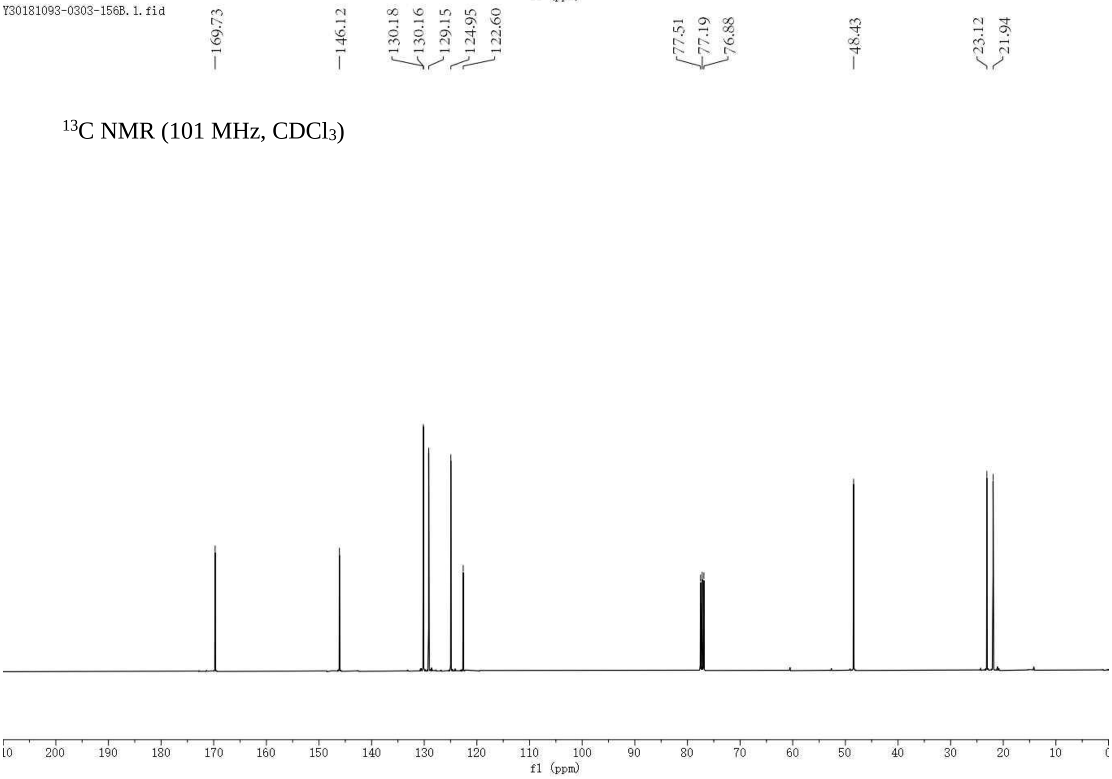

Supplementary Figure 63. ^{1}H NMR and ^{13}C NMR spectra of compound 56.

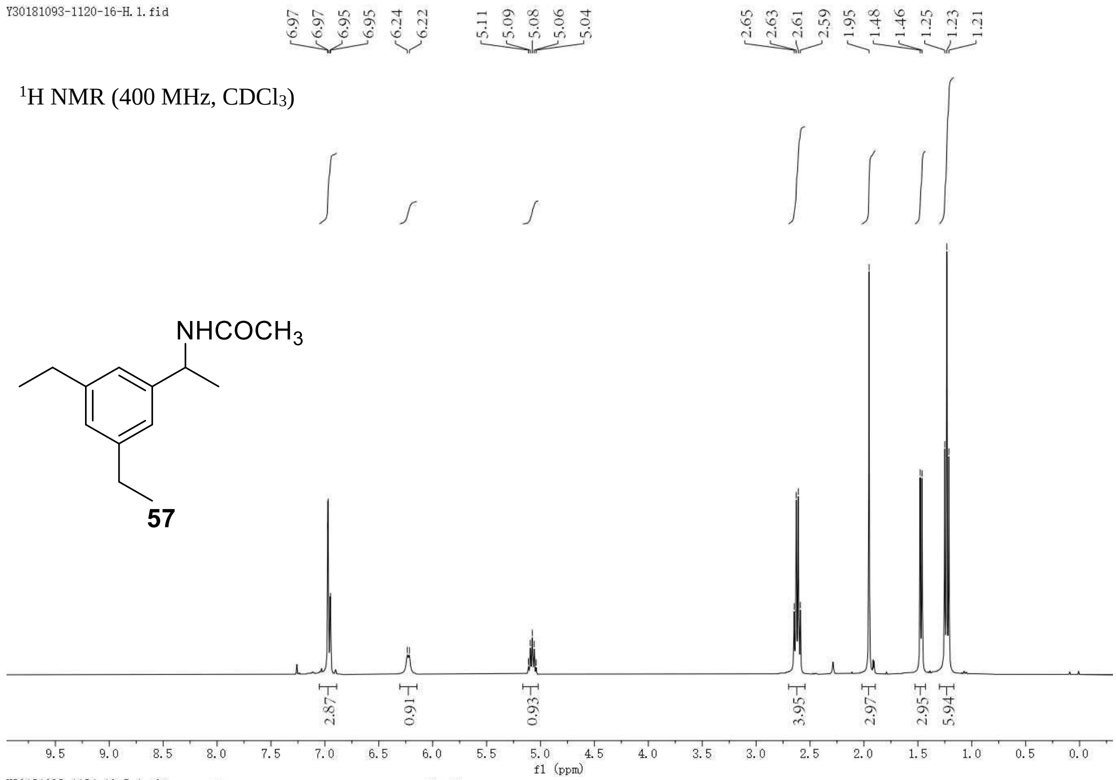

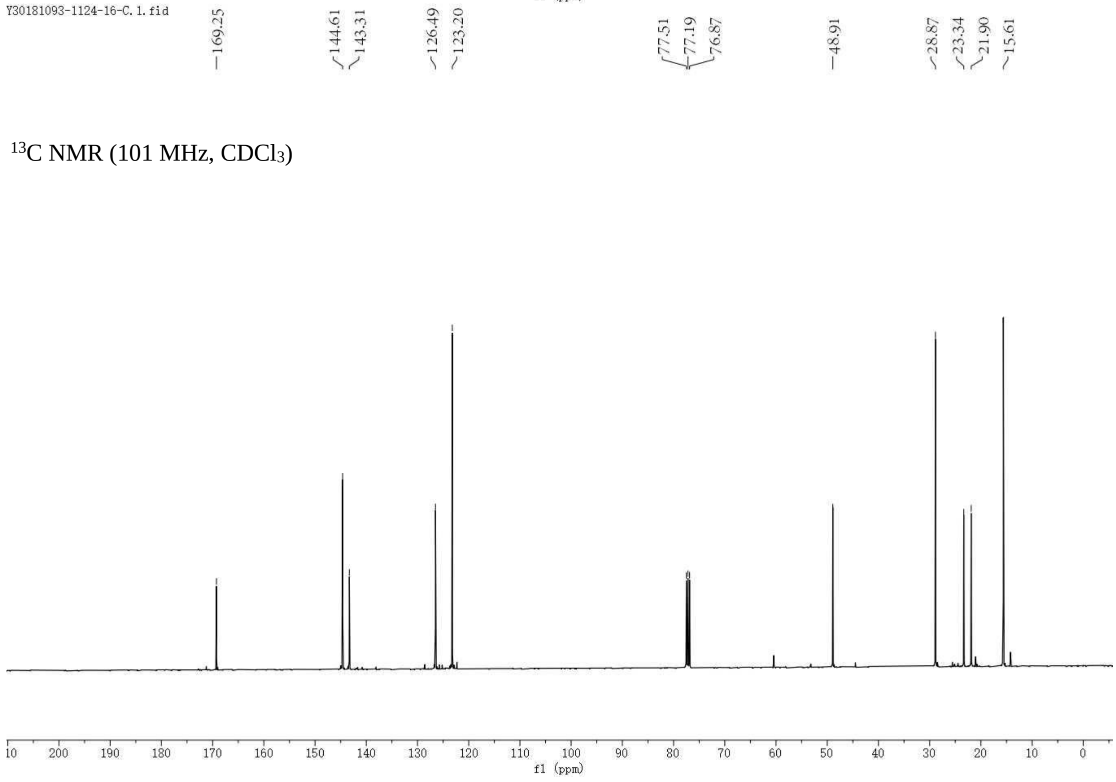

Supplementary Figure 64. ^{1}H NMR and ^{13}C NMR spectra of compound 57.

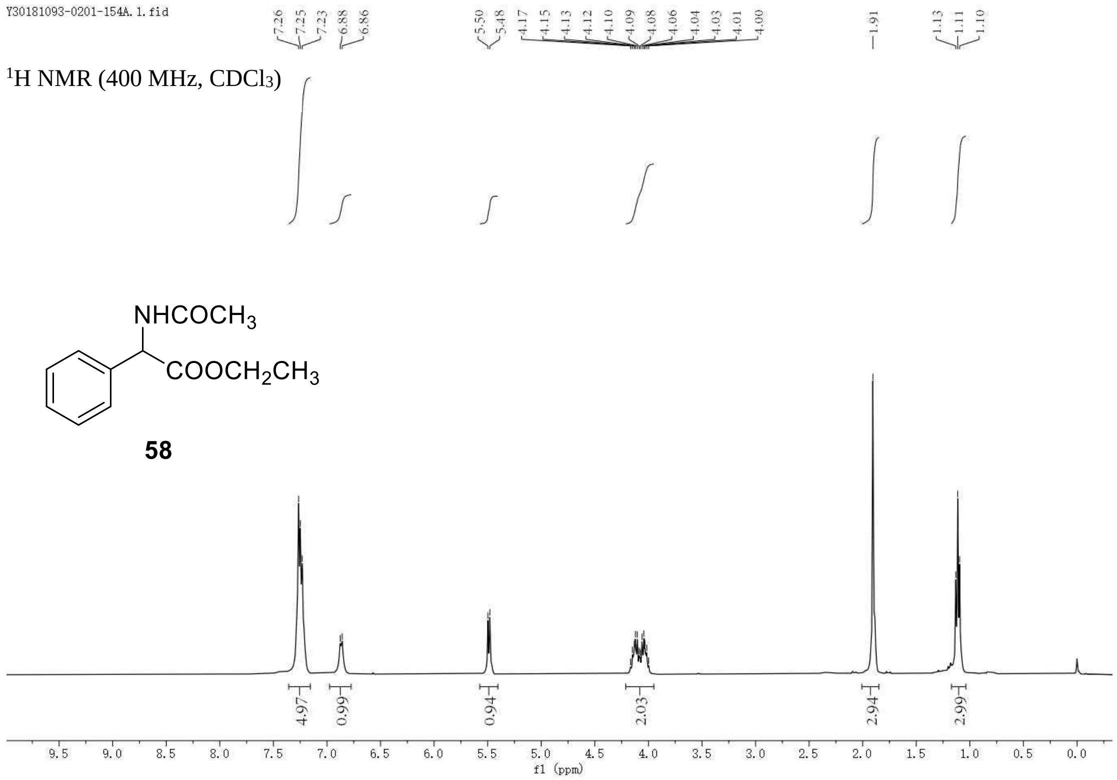

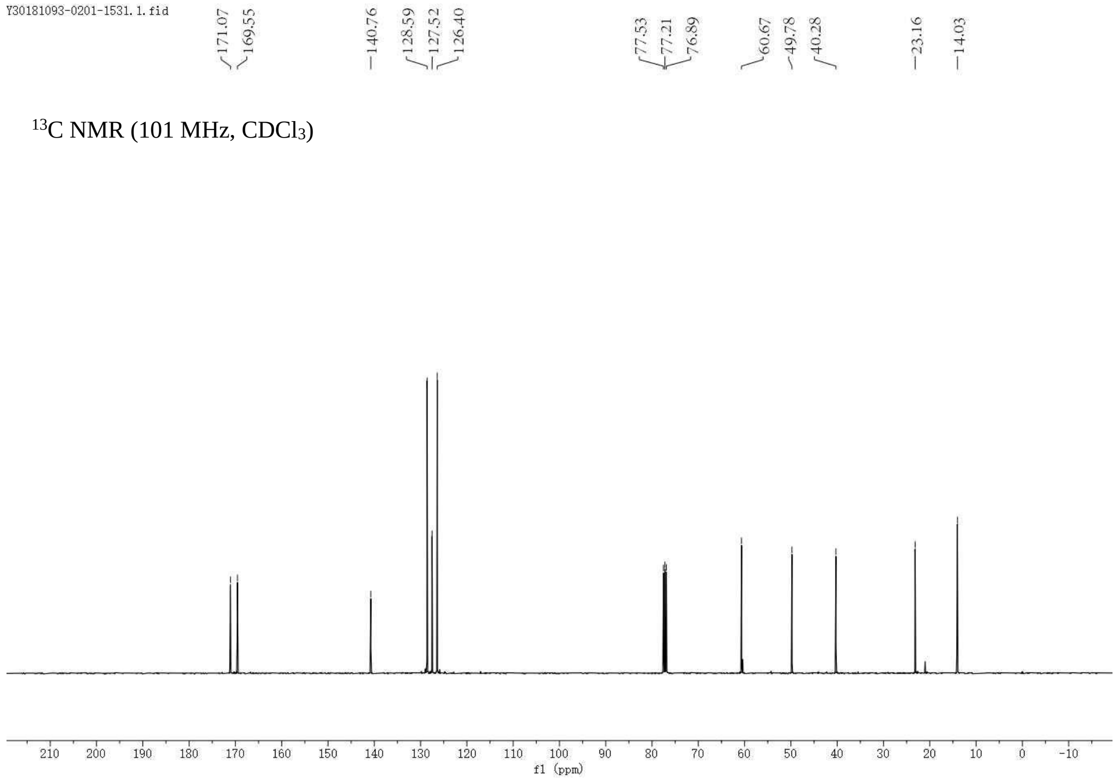

Supplementary Figure 65. ^{1}H NMR and ^{13}C NMR spectra of compound 58.

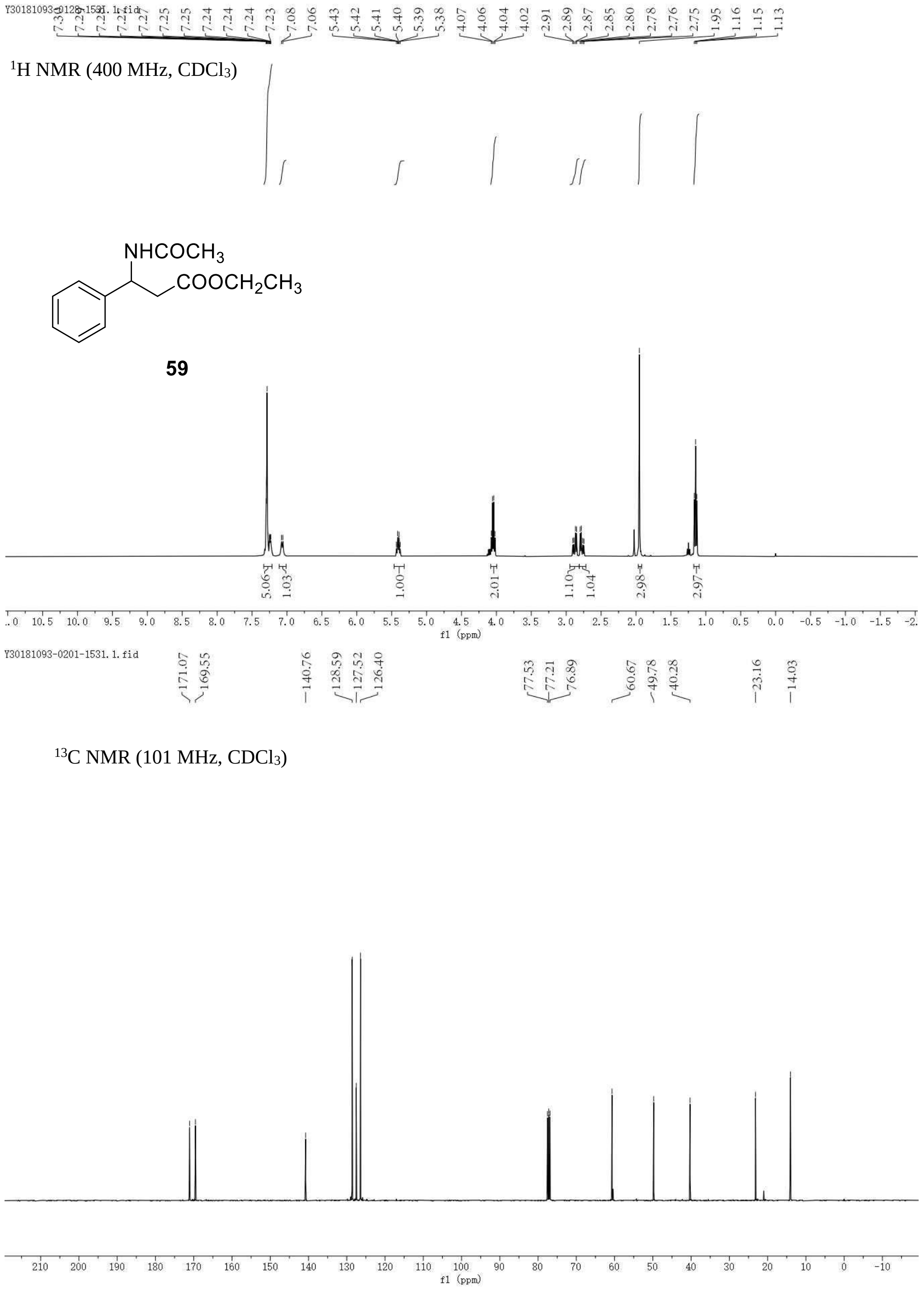

Supplementary Figure 66. ^{1}H NMR and ^{13}C NMR spectra of compound 59.

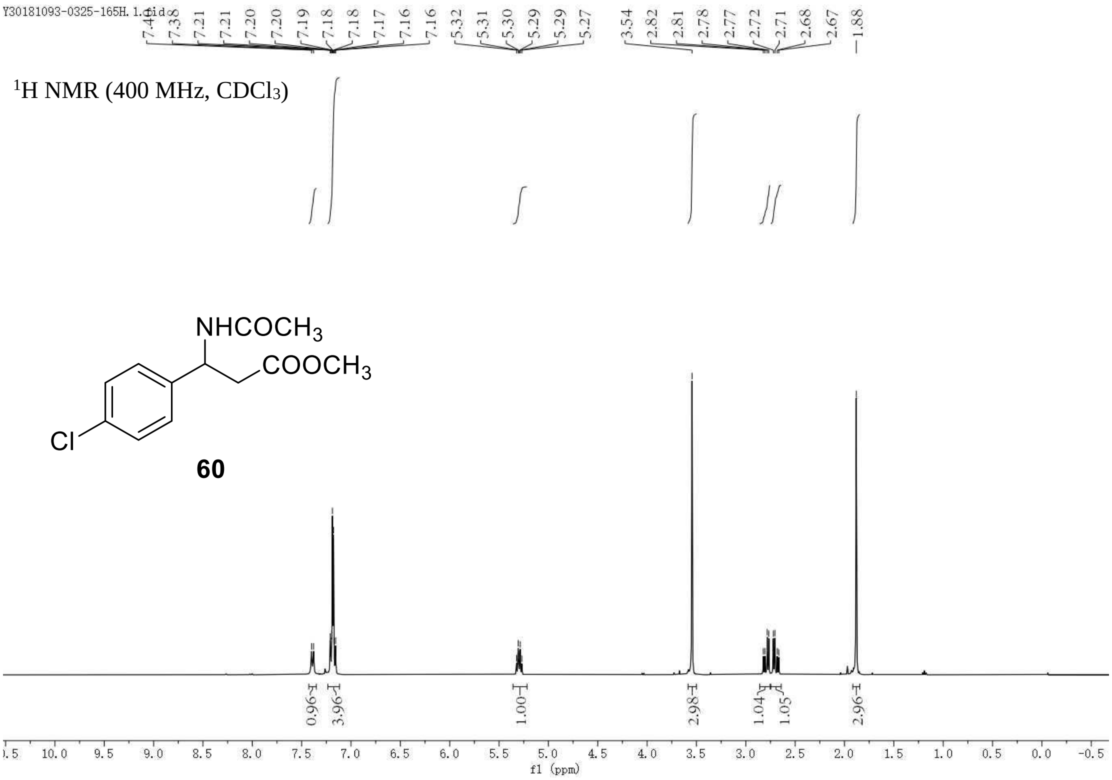

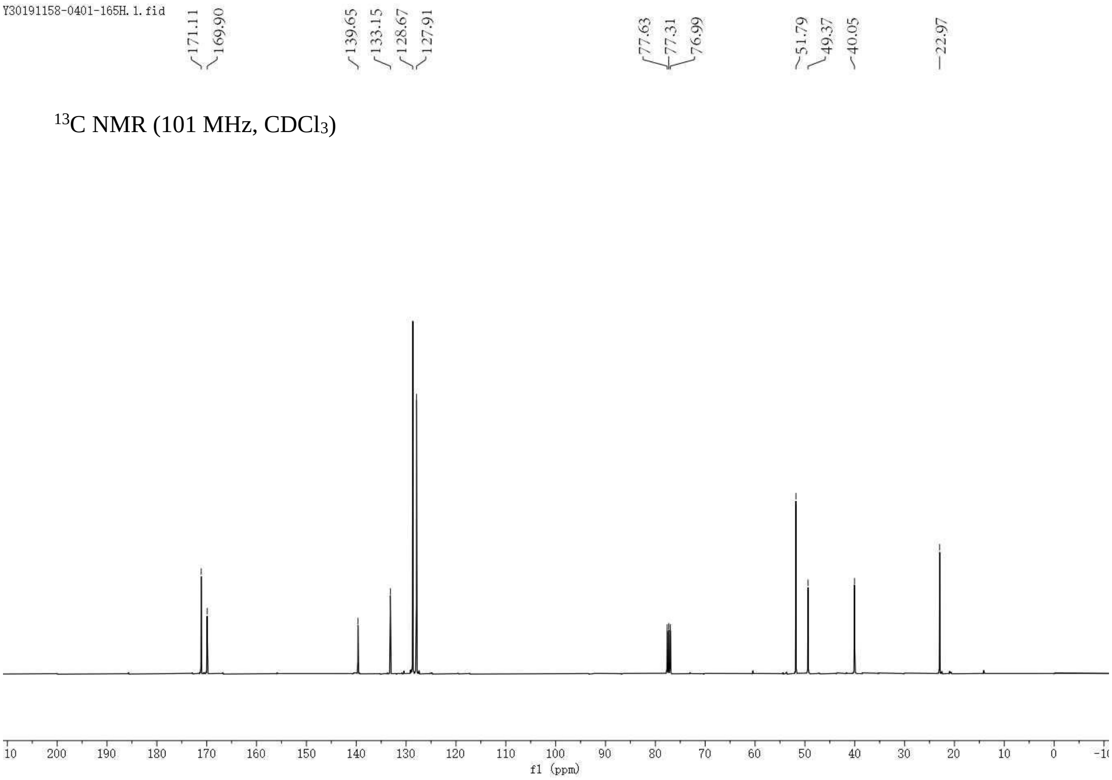

Supplementary Figure 67. ^{1}H NMR and ^{13}C NMR spectra of compound 60.

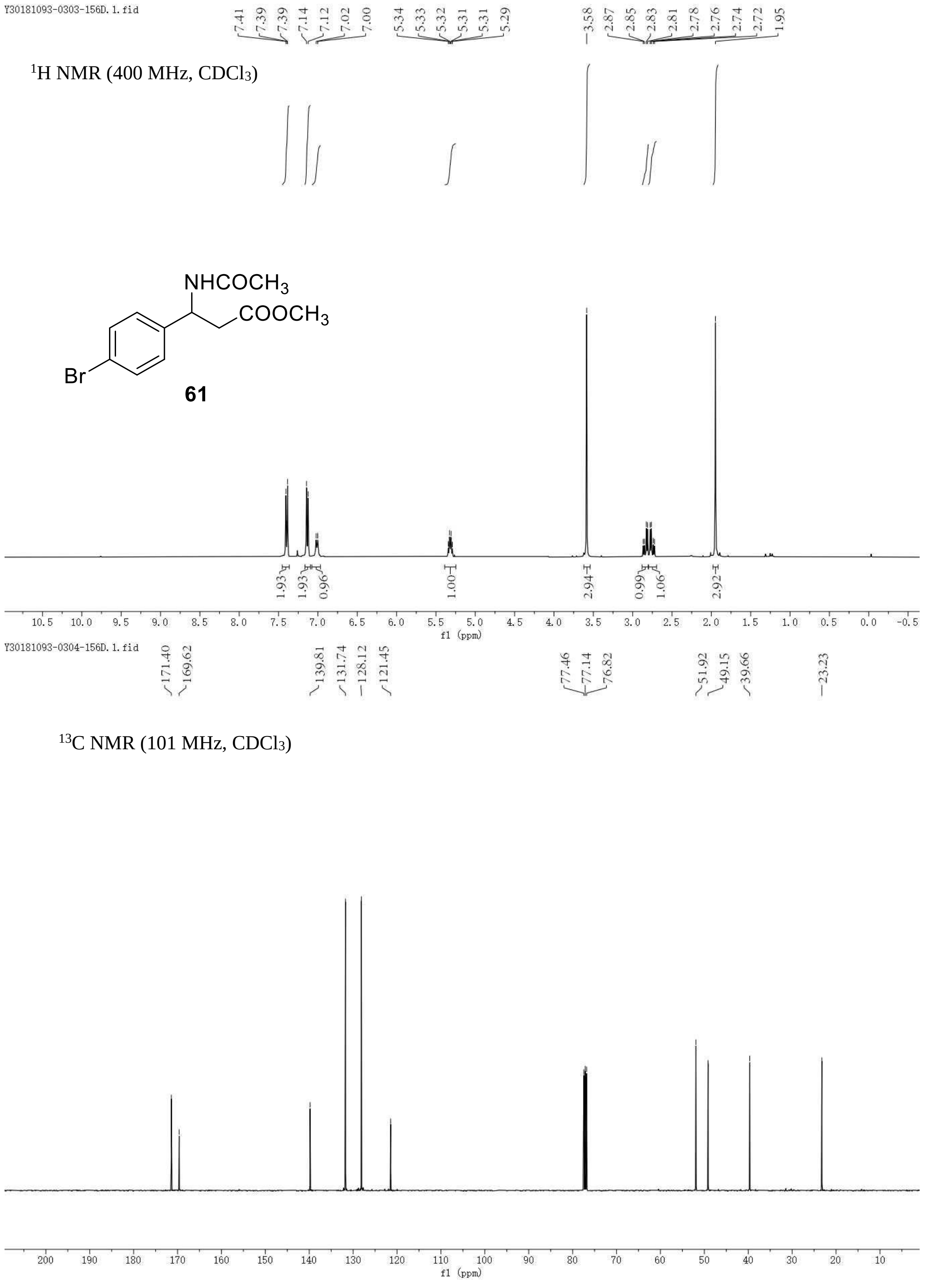

Supplementary Figure 68. ^{1}H NMR and ^{13}C NMR spectra of compound 61.

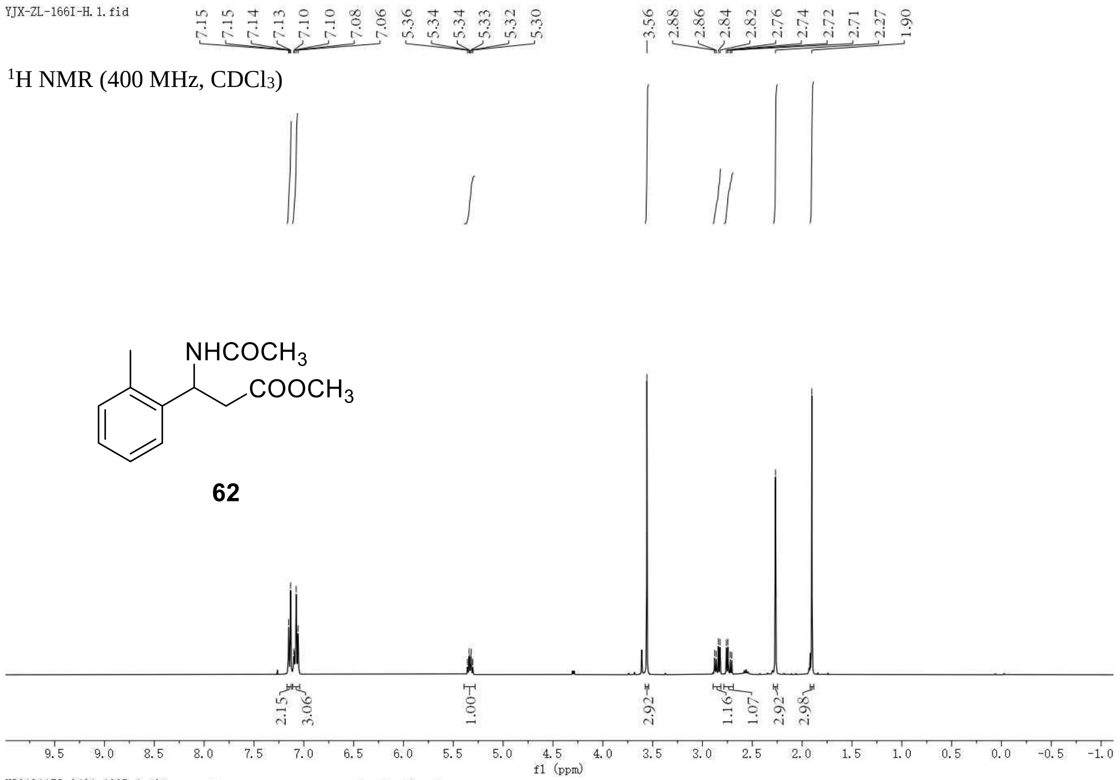

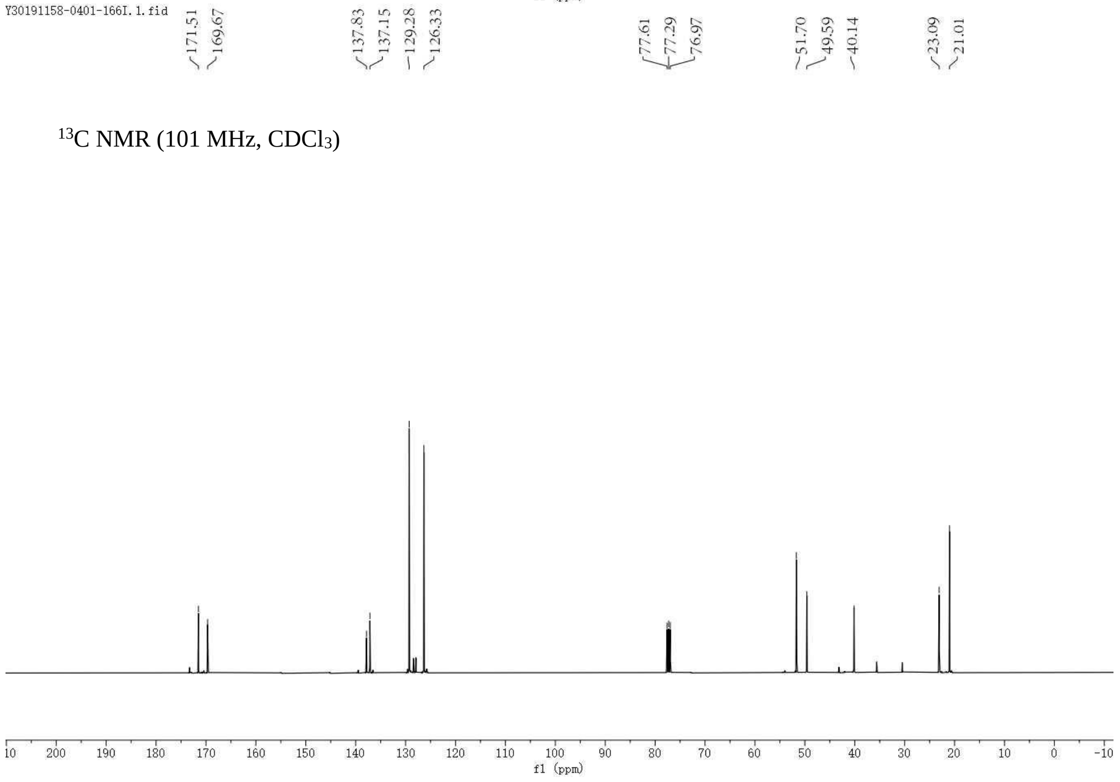

Supplementary Figure 69. ^{1}H NMR and ^{13}C NMR spectra of compound 62.

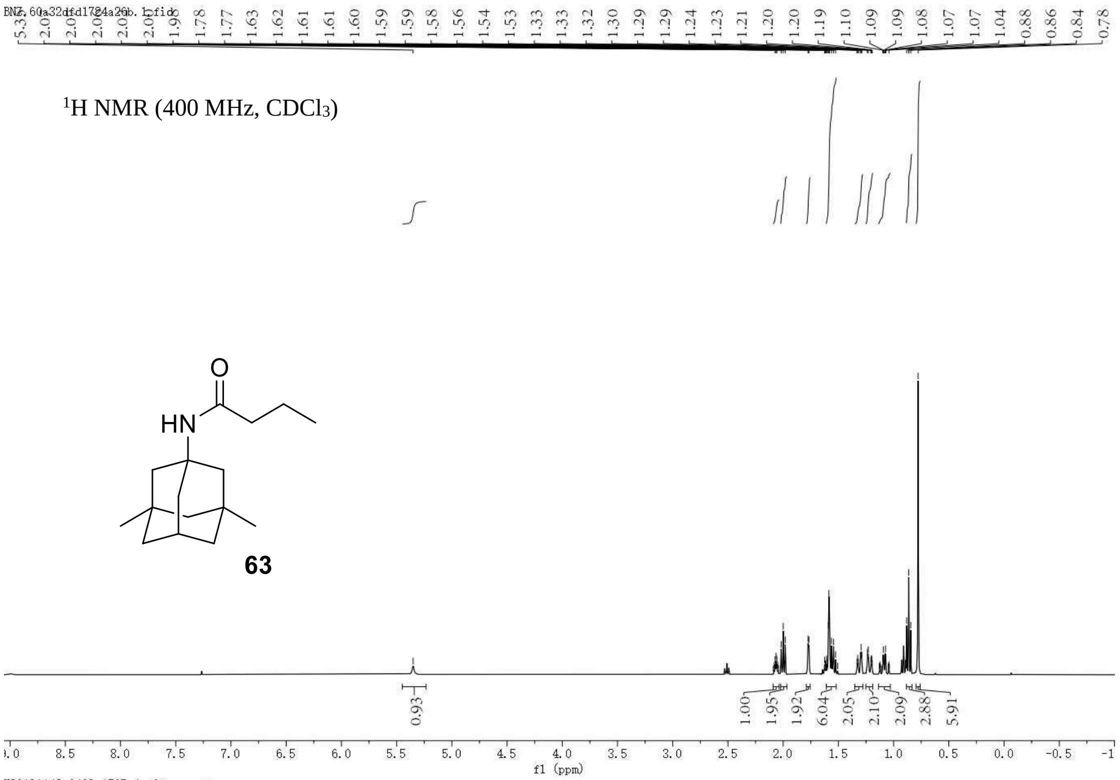

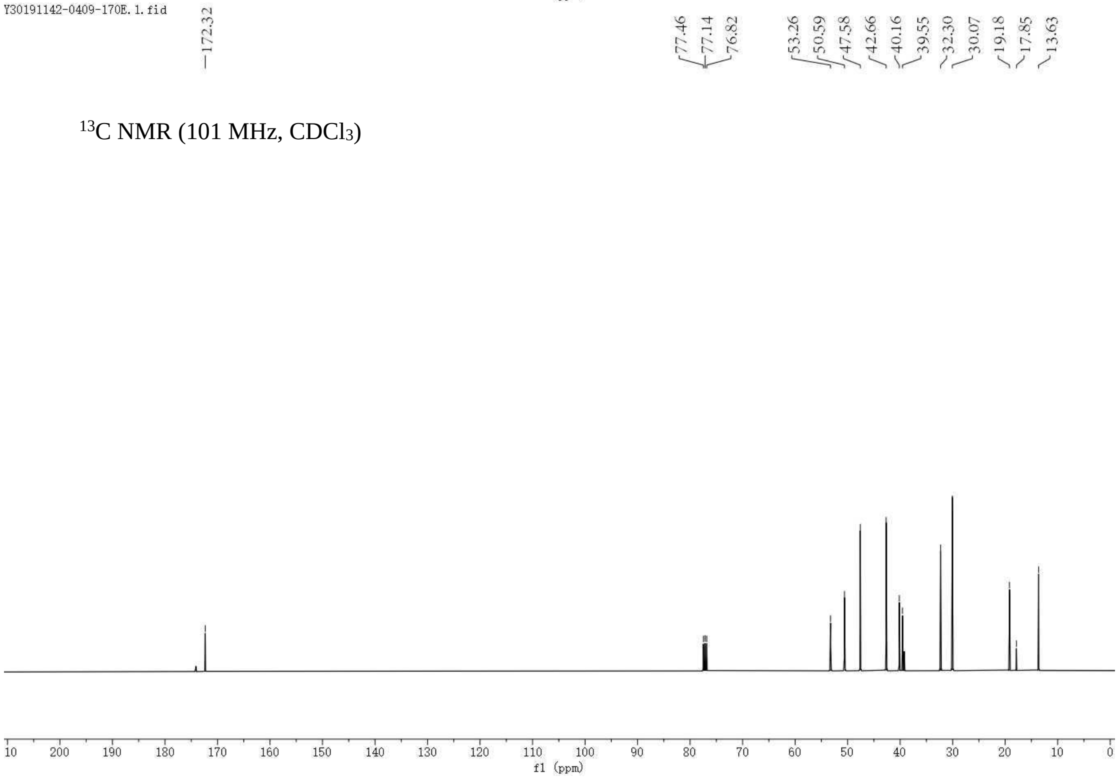

Supplementary Figure 70. ^{1}H NMR and ^{13}C NMR spectra of compound 63.

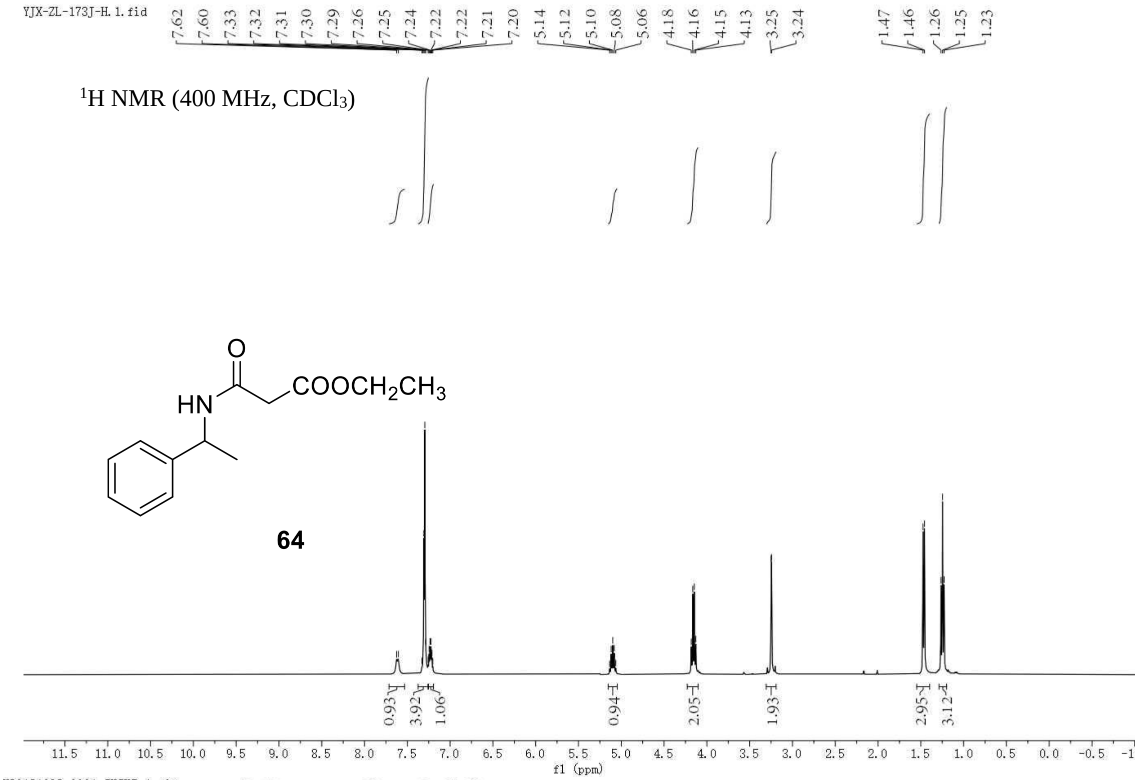

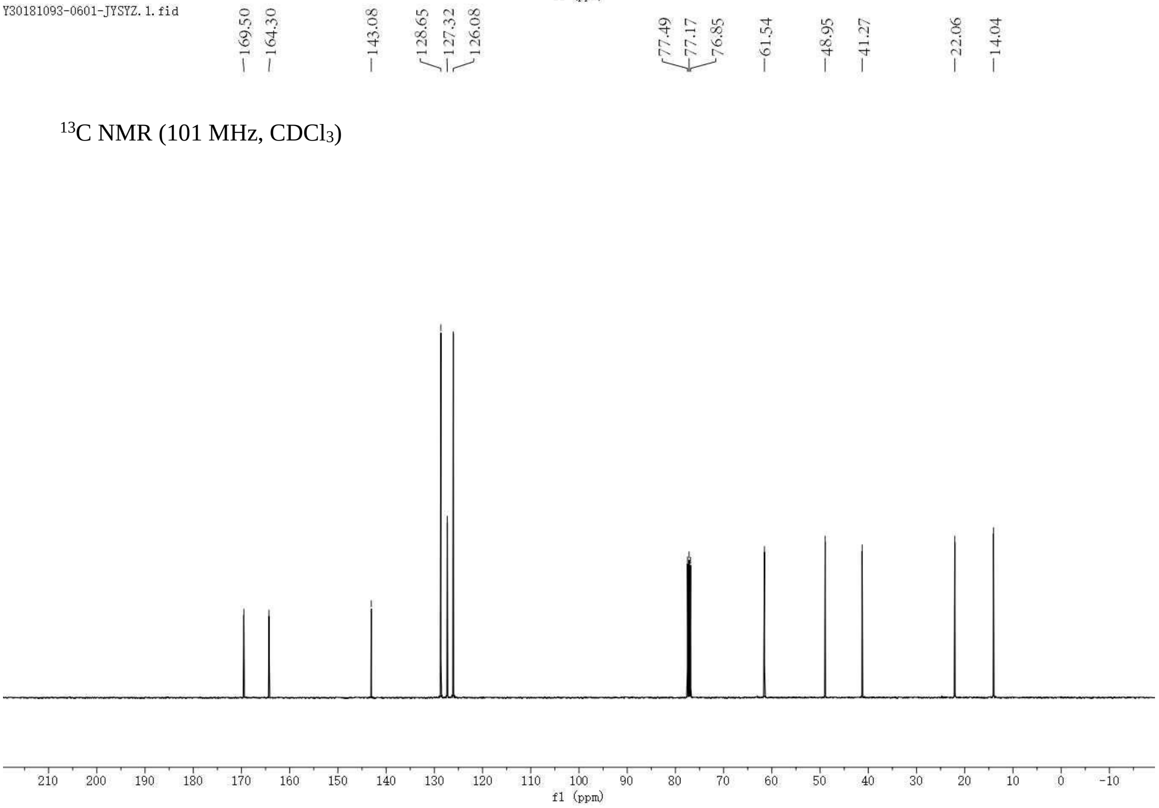

Supplementary Figure 71. ^{1}H NMR and ^{13}C NMR spectra of compound 64.

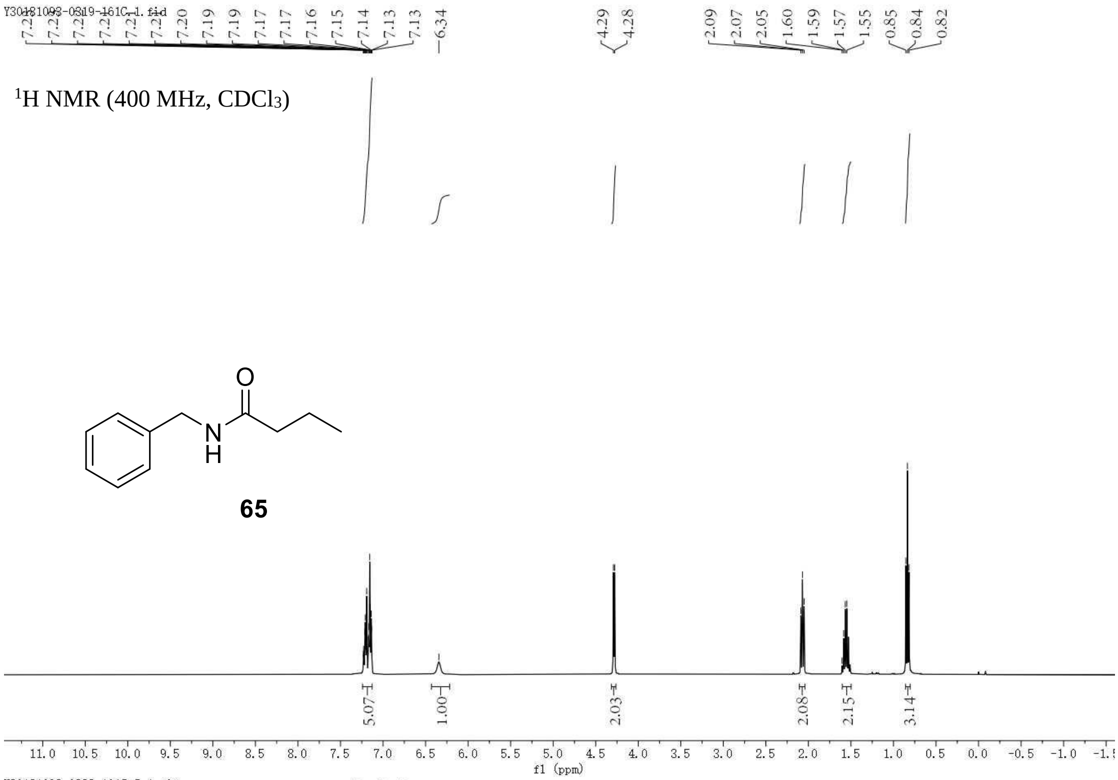

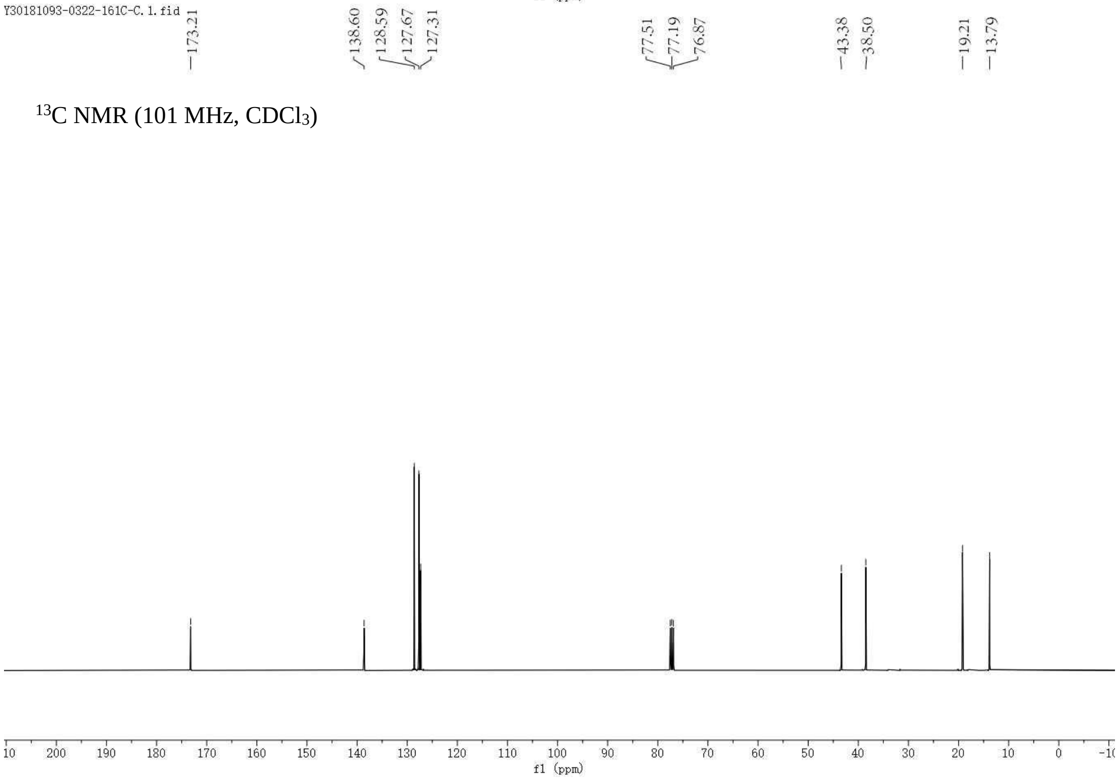

Supplementary Figure 72. ^{1}H NMR and ^{13}C NMR spectra of compound 65.

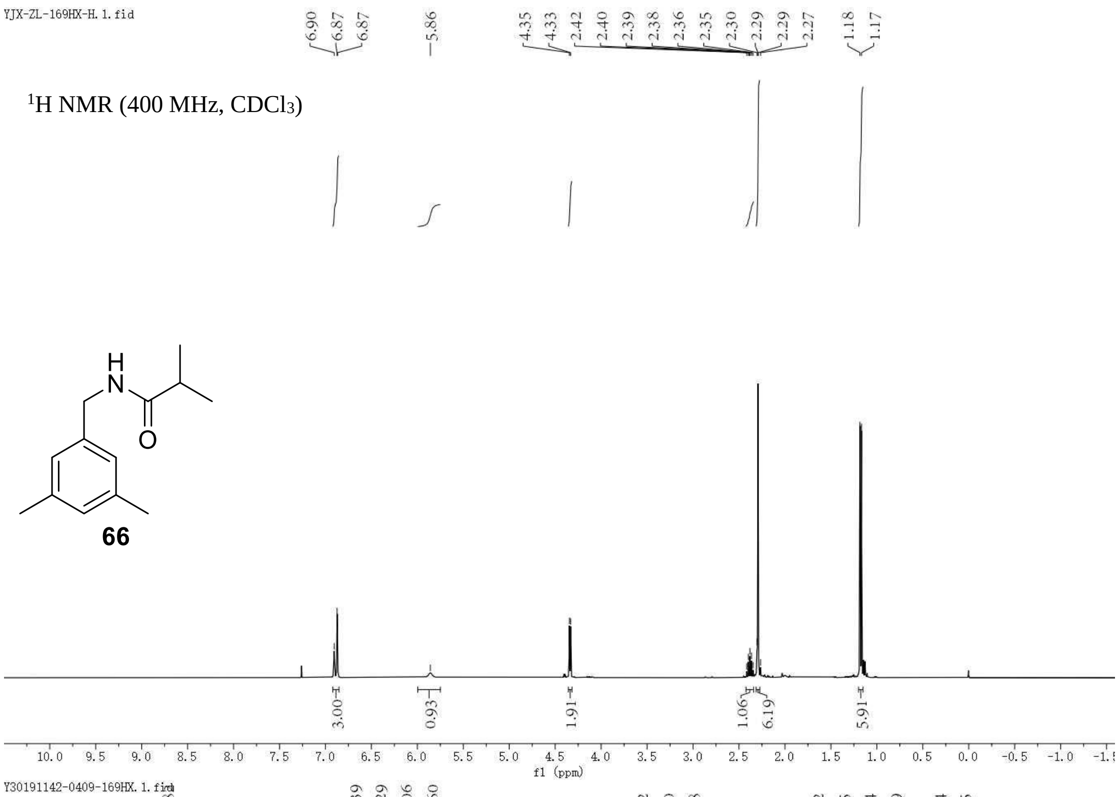

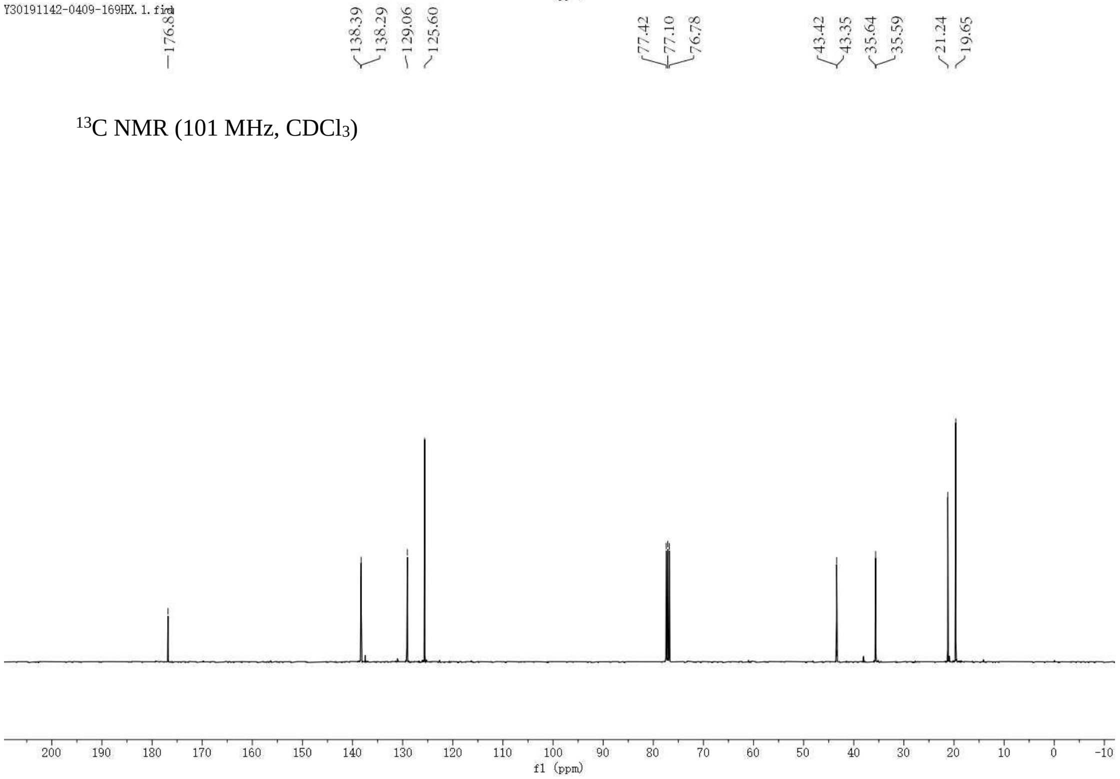

Supplementary Figure 73. ^{1}H NMR and ^{13}C NMR spectra of compound 66.

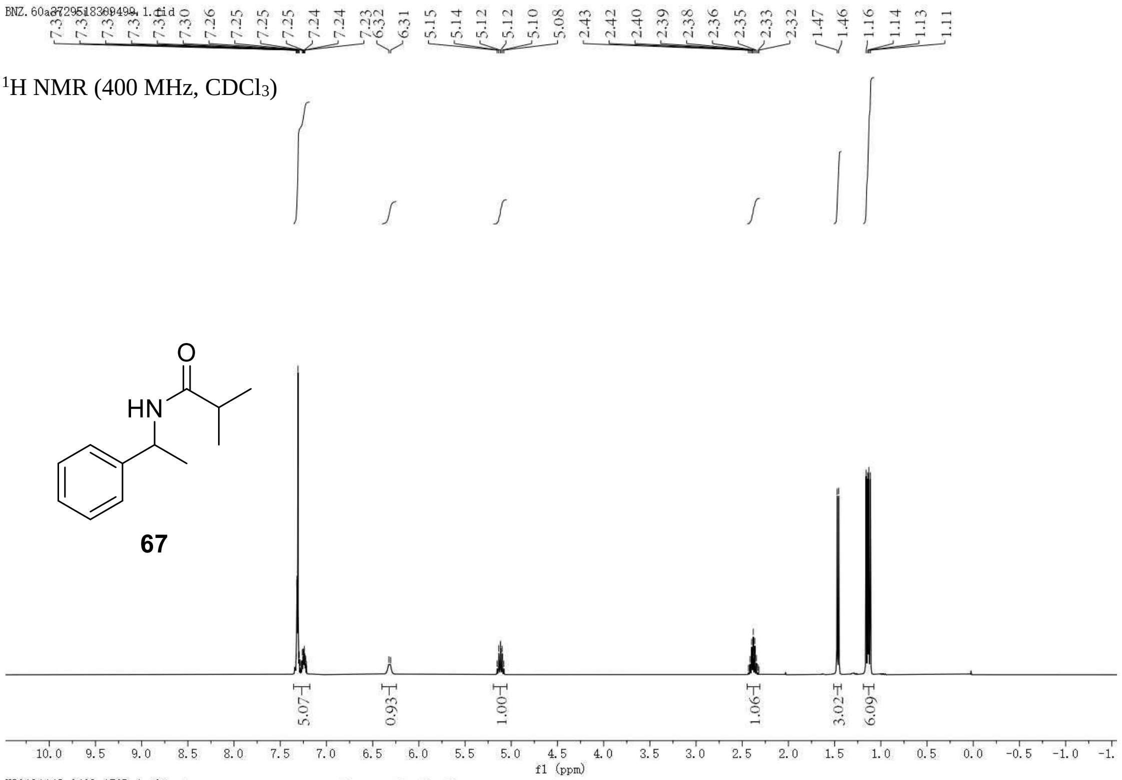

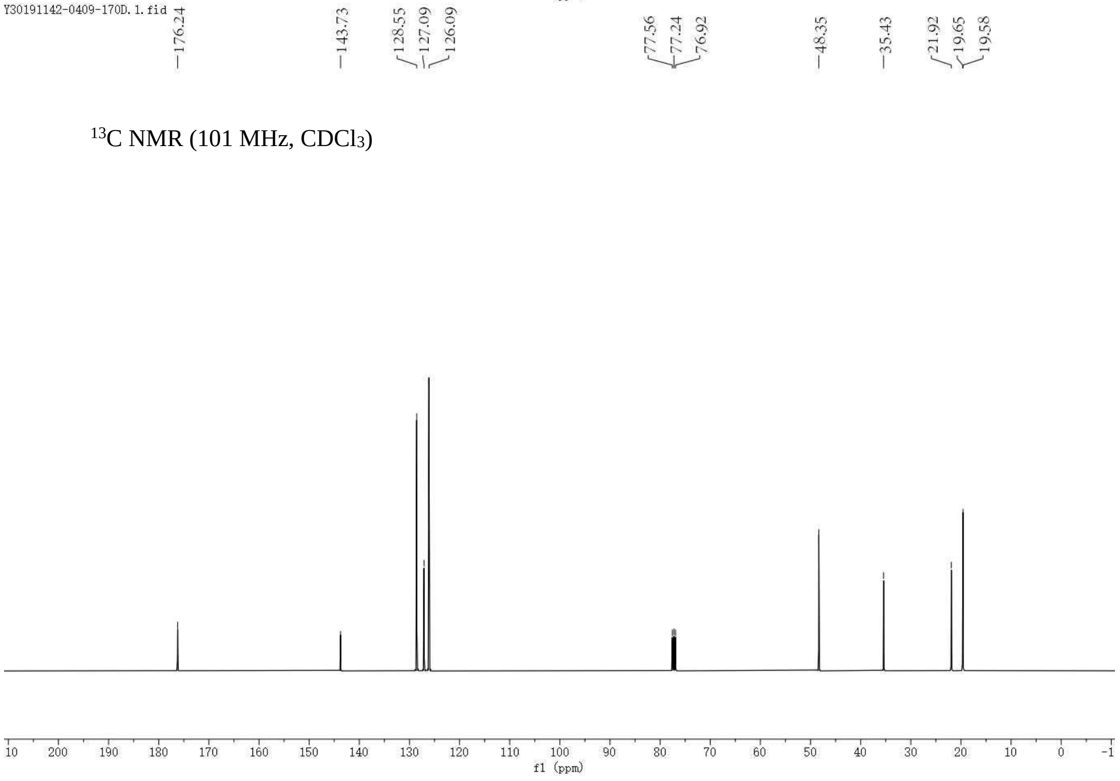

Supplementary Figure 74. ^{1}H NMR and ^{13}C NMR spectra of compound 67.

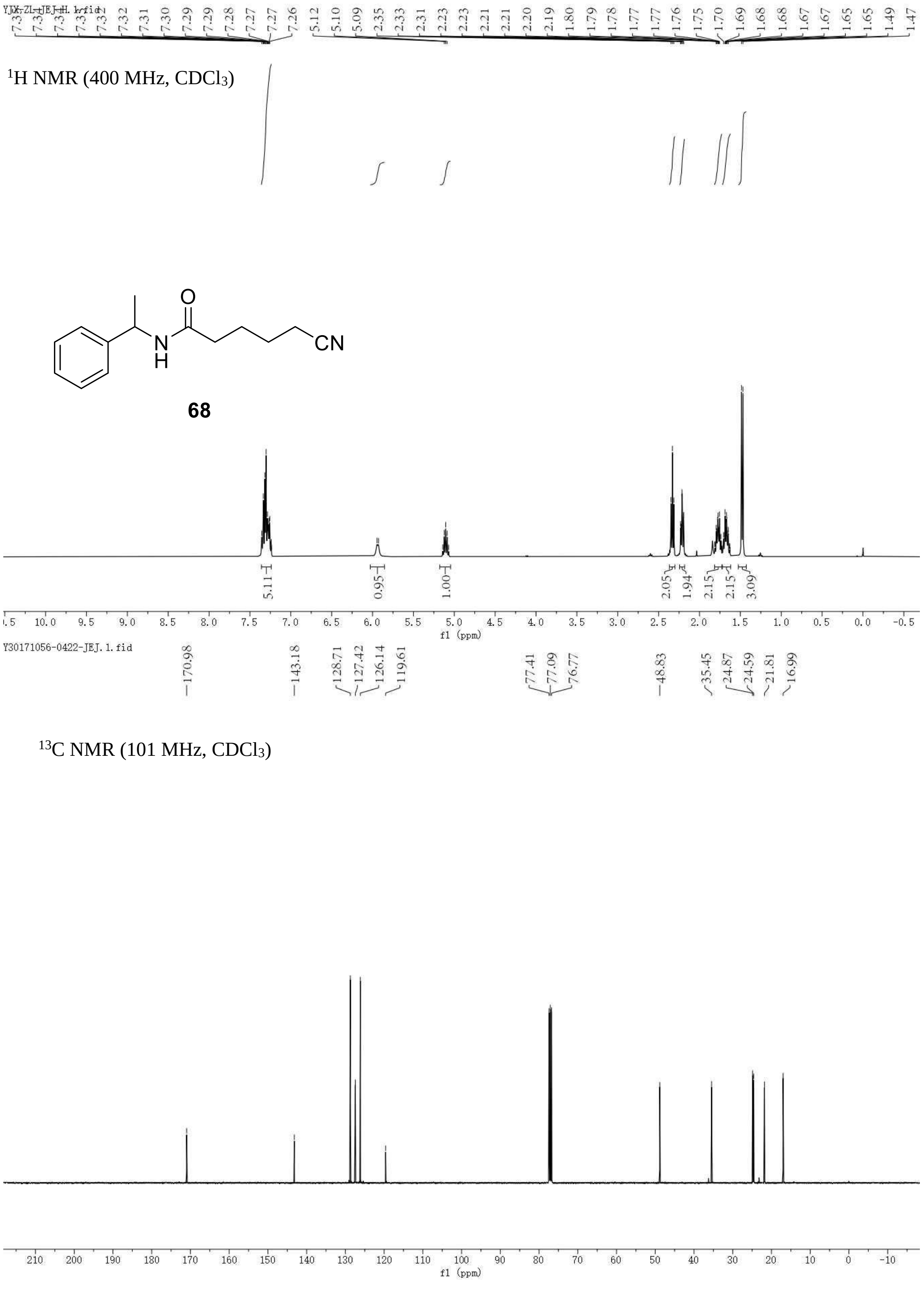

Supplementary Figure 75. ^{1}H NMR and ^{13}C NMR spectra of compound 68.

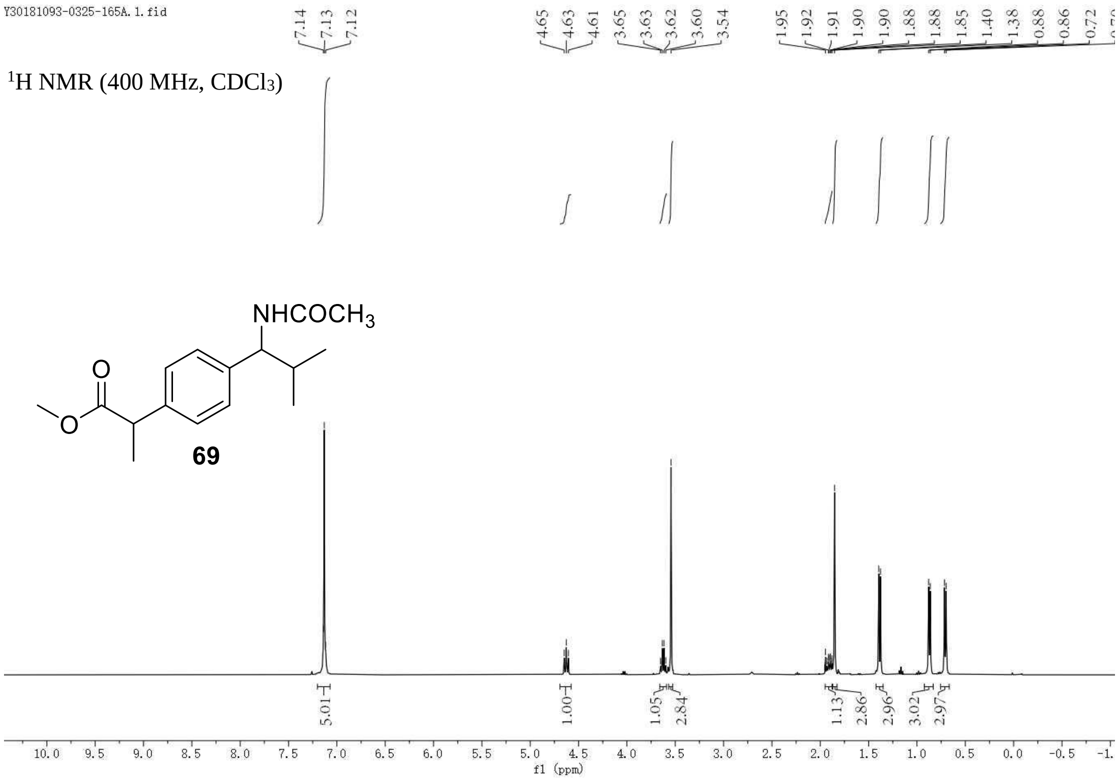

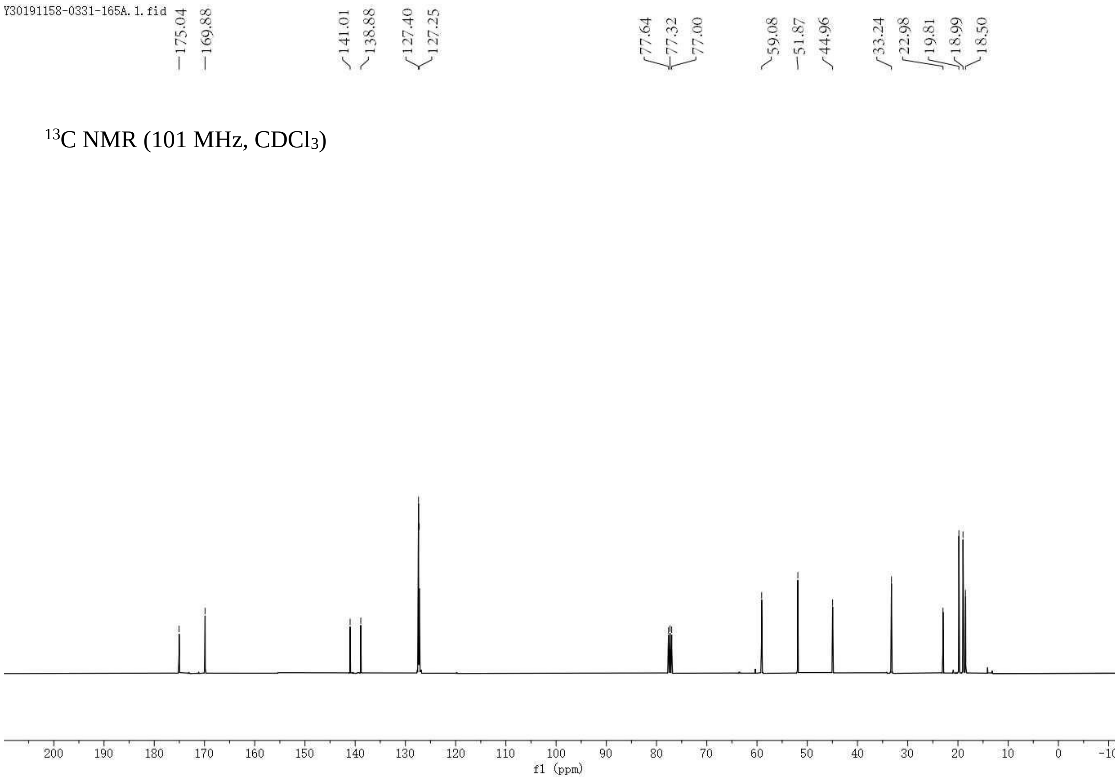

Supplementary Figure 76. ^{1}H NMR and ^{13}C NMR spectra of compound 69.

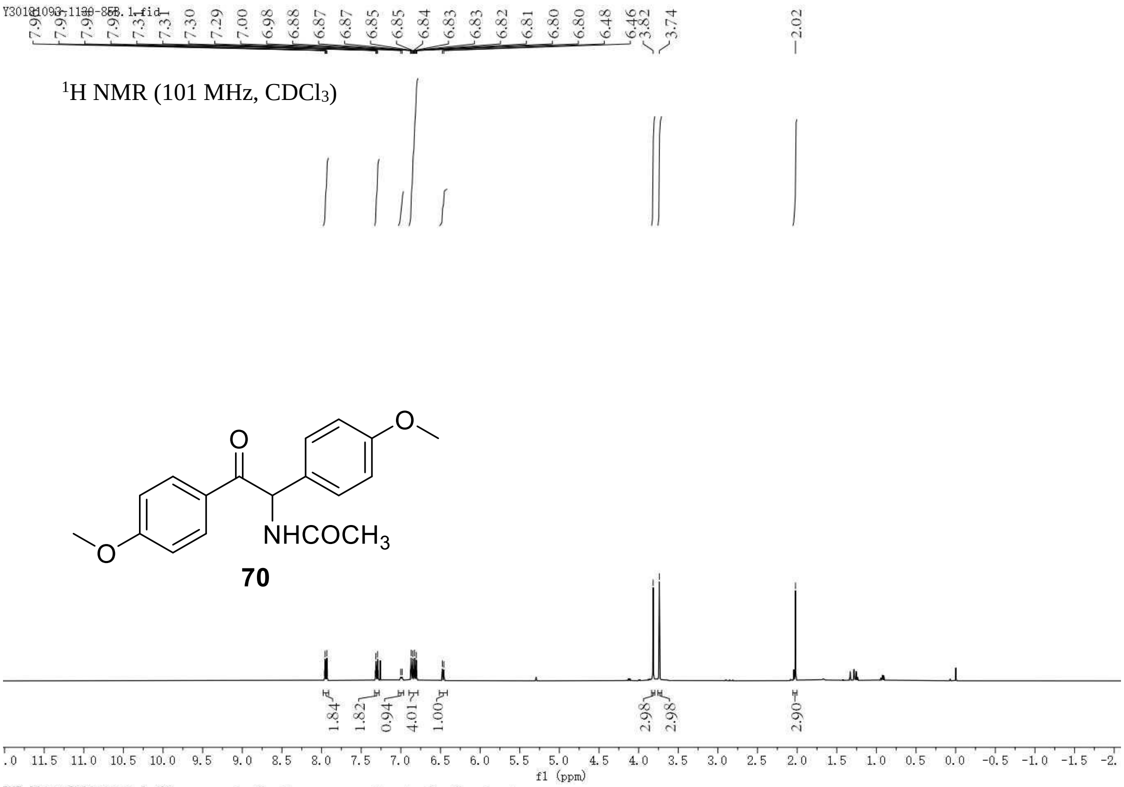

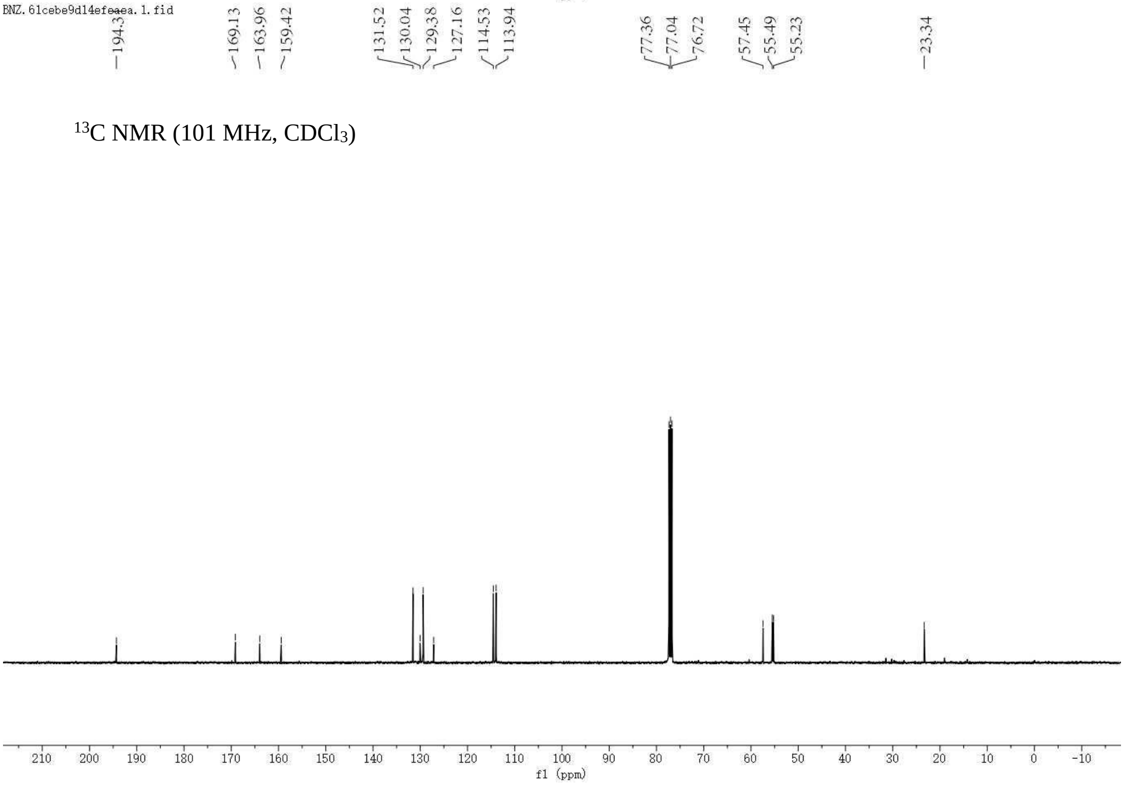

Supplementary Figure 77. ¹H NMR and ¹³C NMR spectra of compound 70.

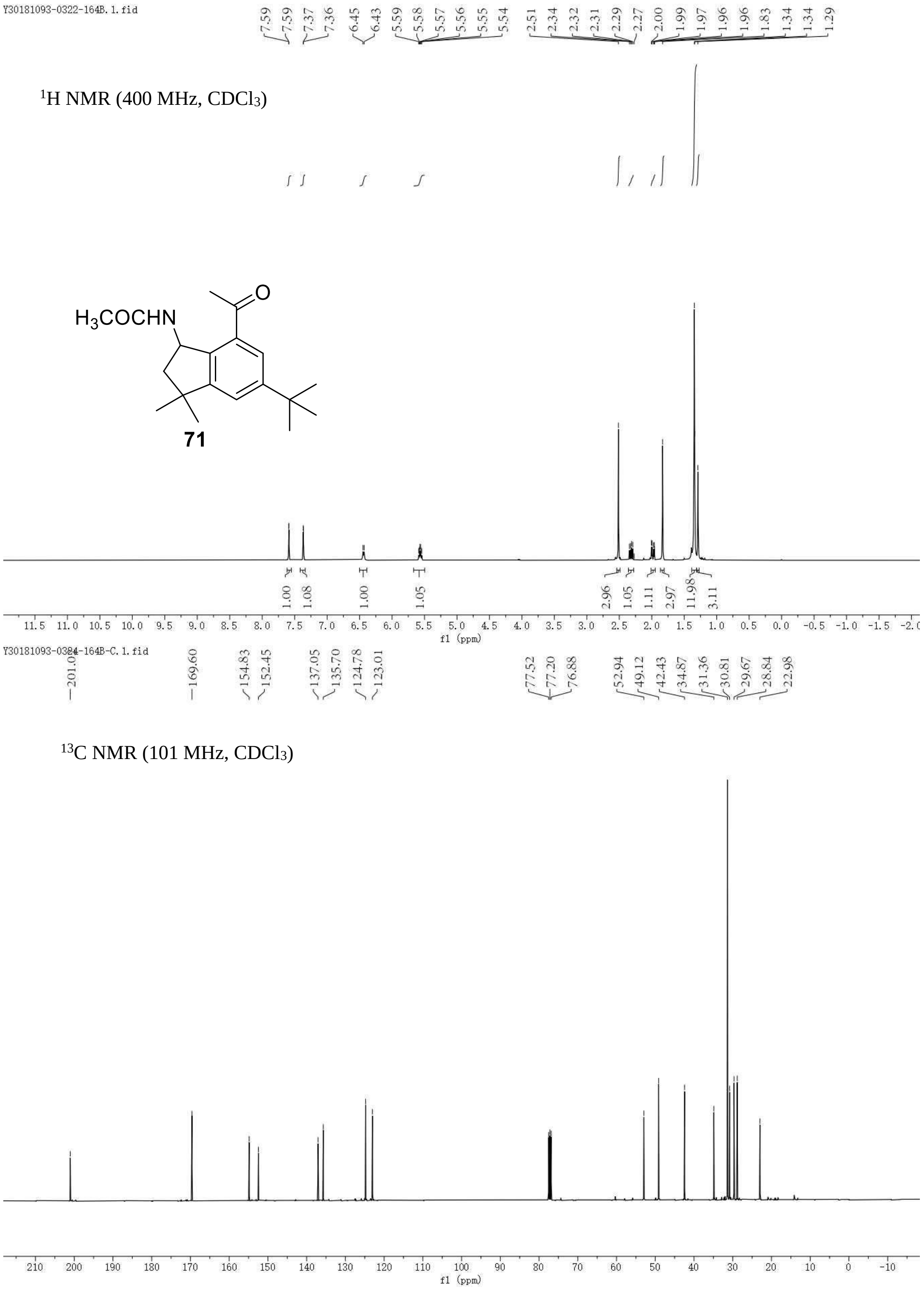

Supplementary Figure 78. ^{1}H NMR and ^{13}C NMR spectra of compound 71.

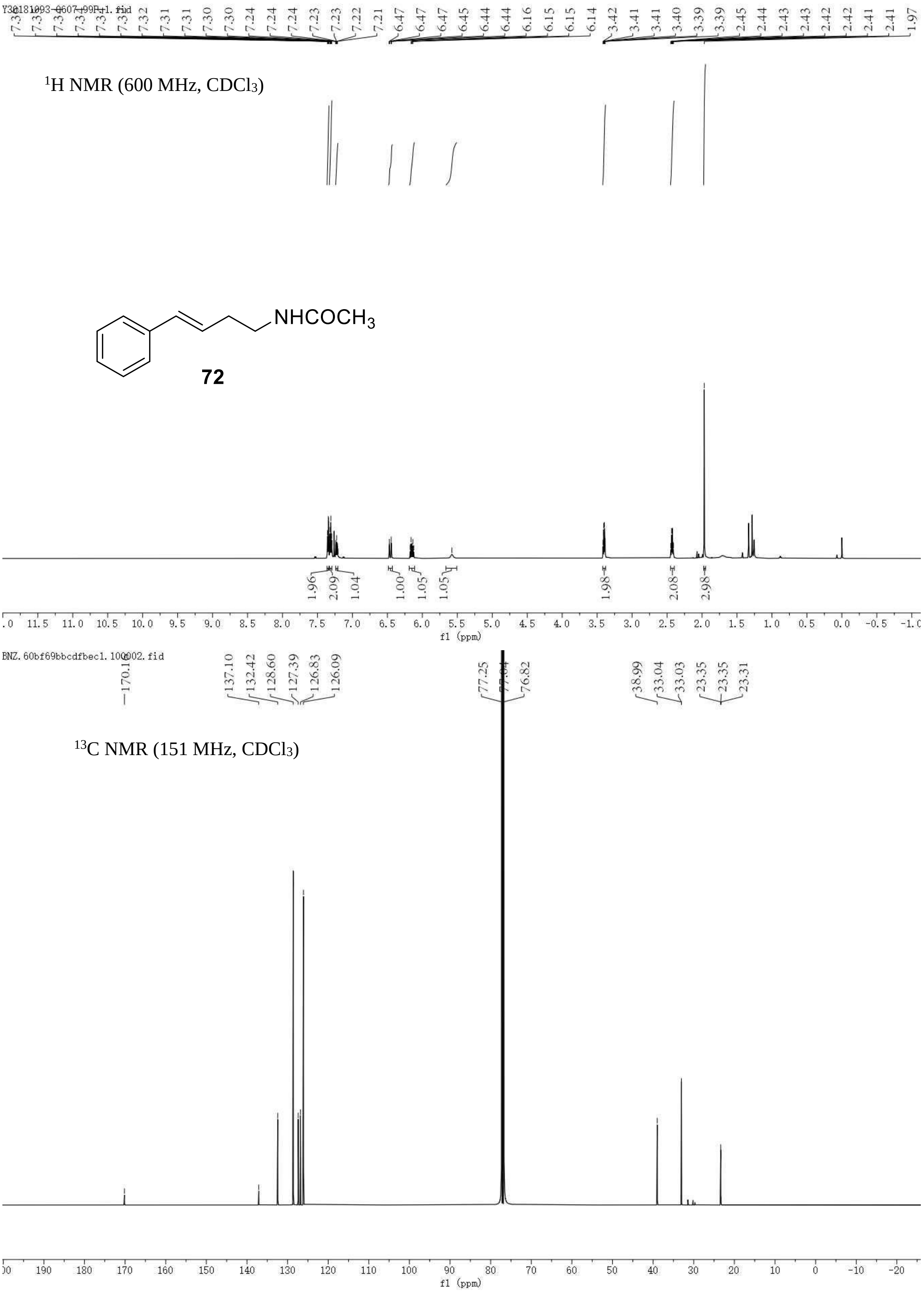

Supplementary Figure 79. ^{1}H NMR and ^{13}C NMR spectra of compound 72.

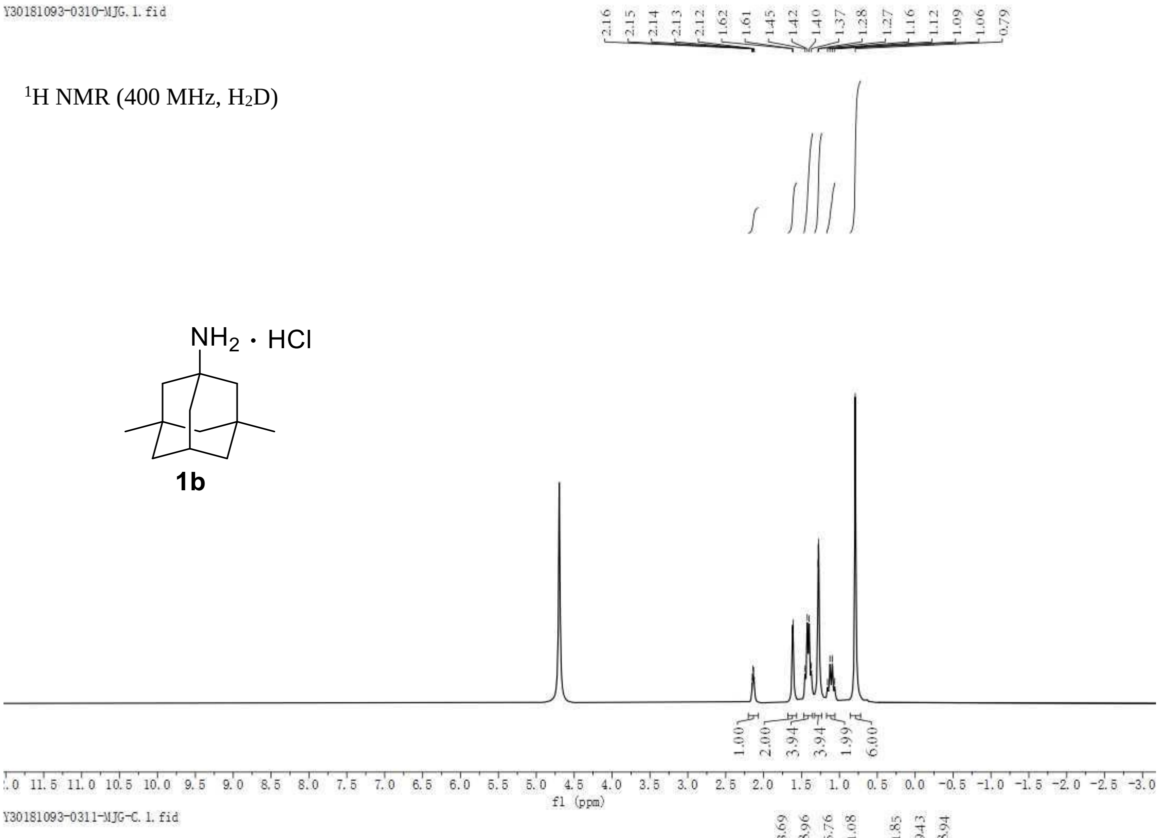

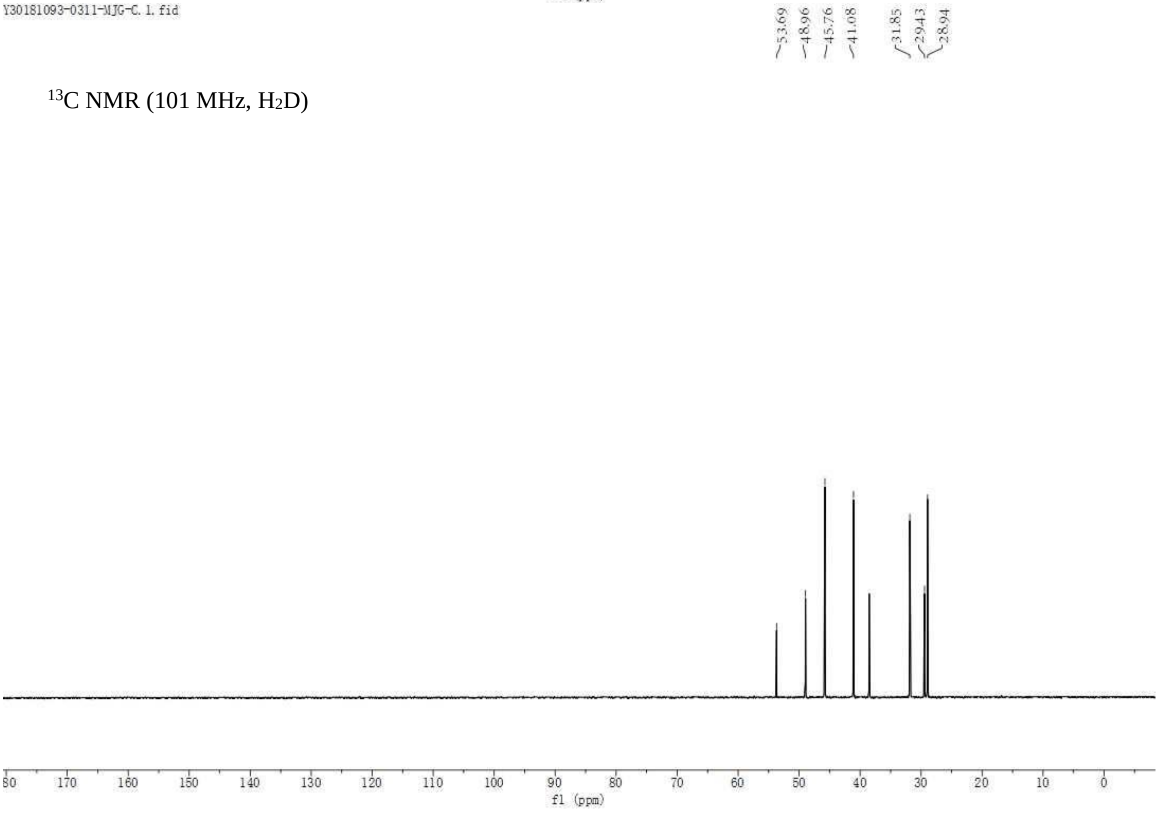

Supplementary Figure 80. ^{1}H NMR and ^{13}C NMR spectra of compound 1b.

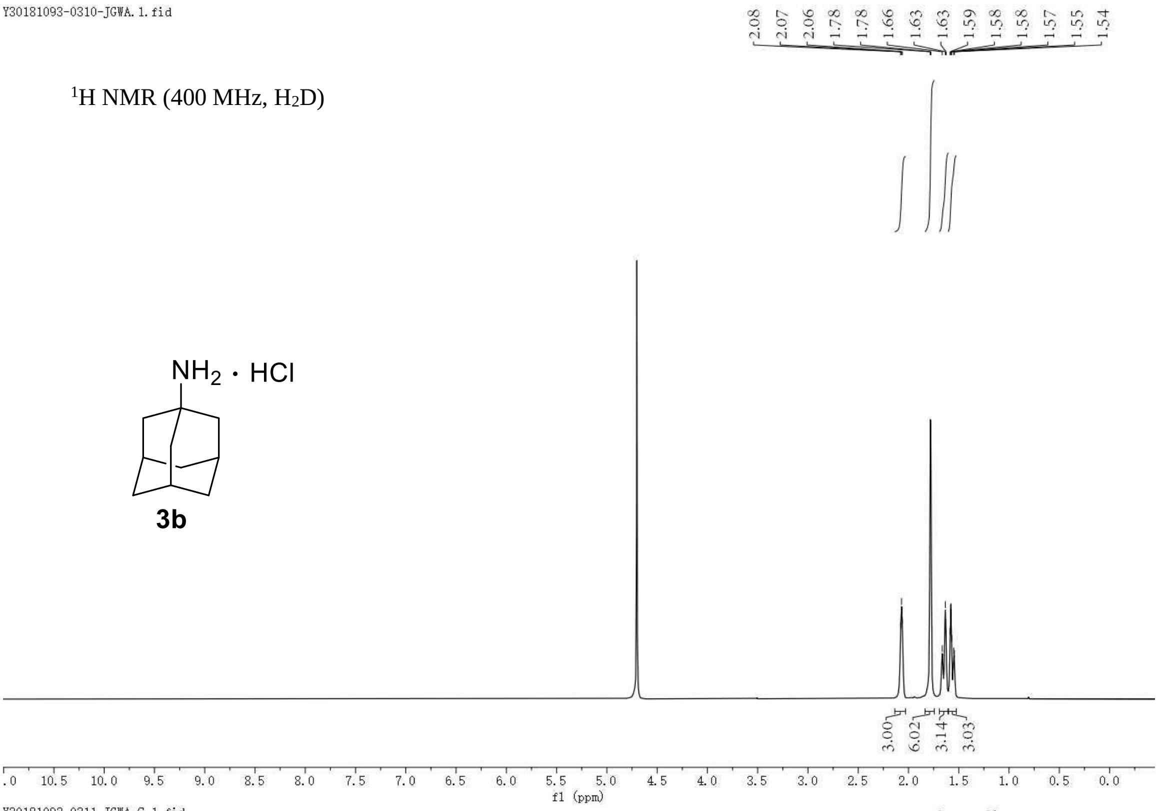

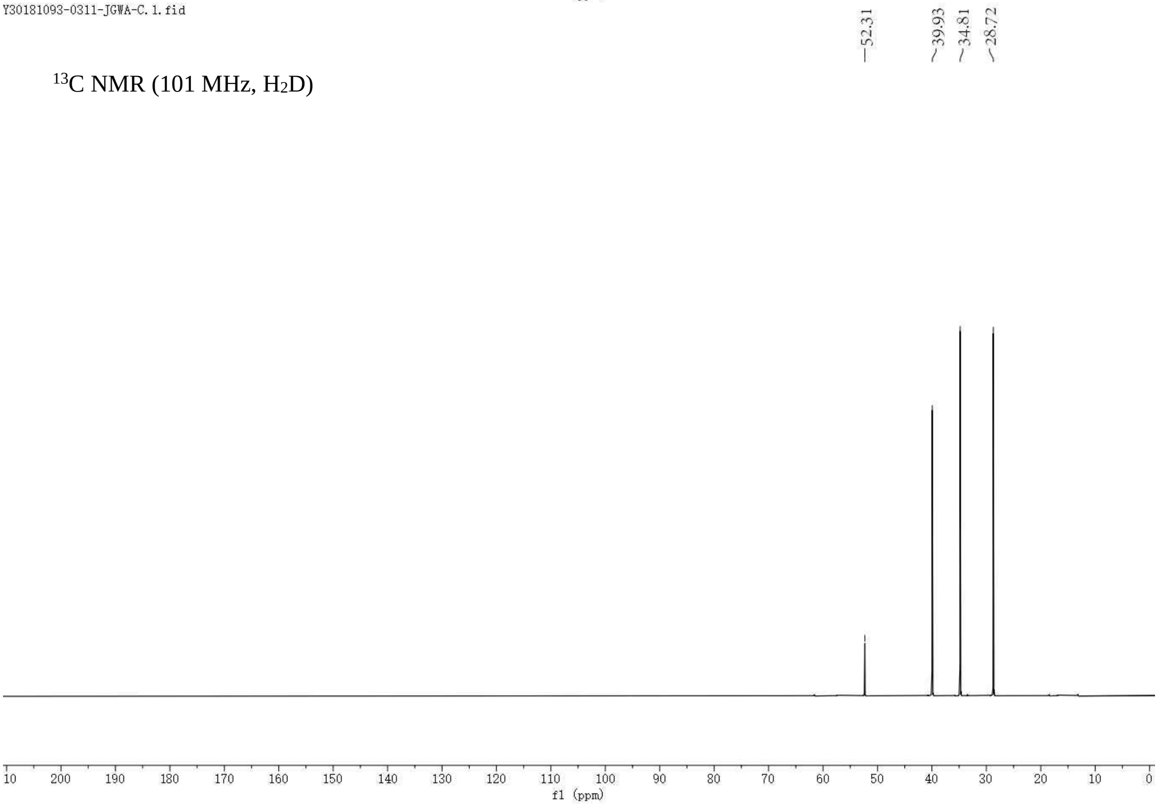

Supplementary Figure 81. ^{1}H NMR and ^{13}C NMR spectra of compound 3b.

^1H NMR (400 MHz, $CDCl_3$)

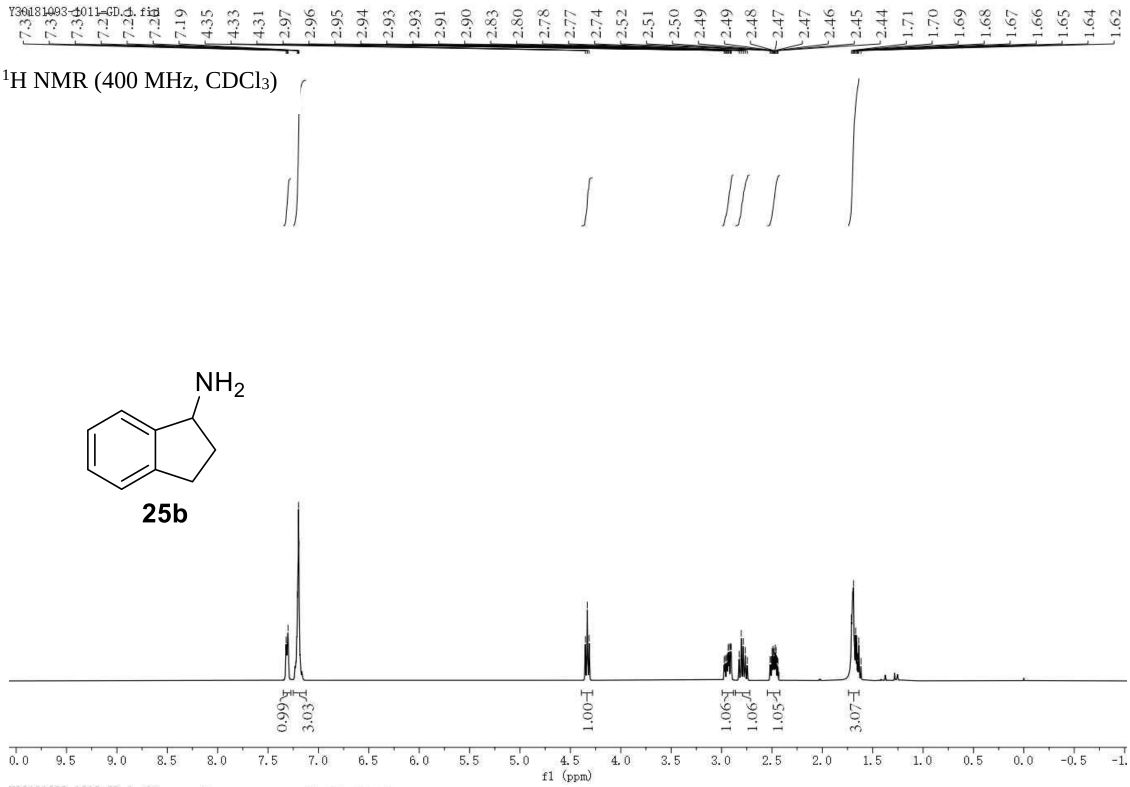

25b

^{13}C NMR (101 MHz, $CDCl_3$)

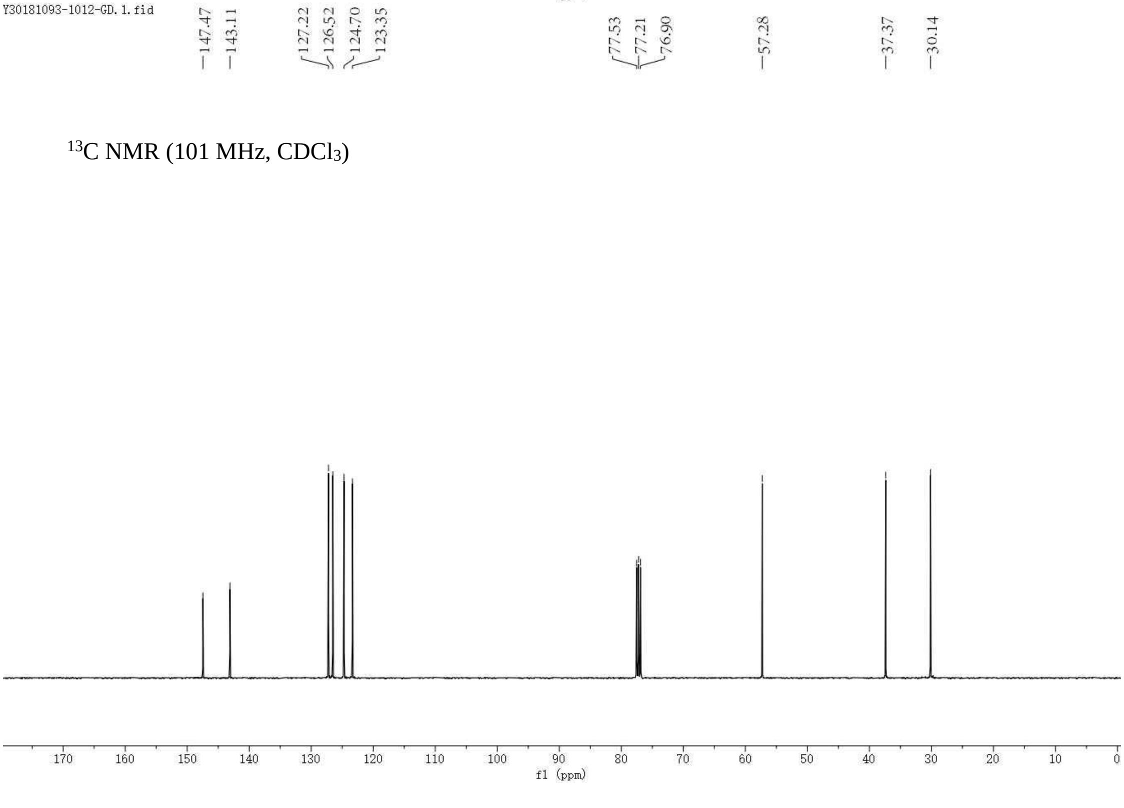

Supplementary Figure 82. ^1H NMR and ^{13}C NMR spectra of compound 25b.

3. Supplementary References

1. Madhra, M. K., Sharma, M. & Khanduri, C. H. New synthetic approach to memantine hydrochloride starting from 1,3-dimethyl-adamantane. *Org. Process Res. Dev*. **11**, 922-923 (2007).
2. Vu, D. B., Nguyen, T. V., Le, S. T. & Phan, C. D. An improved synthesis of amantadine hydrochloride. *Org. Process Res. Dev*. **21**, 1758-1760 (2017).
3. Pérez-Venegasa, M. & EusebioJuaristi, E. Mechanoenzymatic resolution of racemic chiral amines, a green technique for the synthesis of pharmaceutical building blocks. *Tetrahedron.* **74**, 6453-6458 (2018).
4. Mazzarella, D., Crisenza, G. E. M. & Melchiorre, P. Asymmetric photocatalytic C-H functionalization of toluene and derivatives. *J. Am. Chem. Soc.* **140**, 8439-8443 (2018).
5. Liang, C.-J., Huang, C.-F., Mohanty, N. & Kurakalva, R. M. A rapid spectrophotometric determination of persulfate anion in ISCO. *Chemosphere.* **73**, 1540-1543 (2008).
